# ﻿Revision of the genus *Agrostis* (Poaceae, Pooideae, Poeae) in Megamexico

**DOI:** 10.3897/phytokeys.230.105878

**Published:** 2023-08-11

**Authors:** J. Luis Vigosa-Mercado, Alfonso Delgado-Salinas, Leonardo O. Alvarado Cárdenas, Luis E. Eguiarte

**Affiliations:** 1 Posgrado en Ciencias Biológicas, Universidad Nacional Autónoma de México, Ciudad Universitaria, Av. Universidad 3000, Coyoacán, 04510, Cd. Mx., Mexico Universidad Nacional Autónoma de México Mexico City Mexico; 2 Departamento de Botánica, Instituto de Biología, Universidad Nacional Autónoma de México, Ciudad Universitaria, Av. Universidad 3000, Coyoacán, 04510, Cd. Mx., Mexico Universidad Nacional Autónoma de México Mexico City Mexico; 3 Departamento de Biología Comparada, Facultad de Ciencias, Universidad Nacional Autónoma de México, Ciudad Universitaria, Av. Universidad 3000, Coyoacán, 04510, Cd. Mx., Mexico Universidad Nacional Autónoma de México Mexico City Mexico; 4 Departamento de Ecología Evolutiva, Instituto de Ecología, Universidad Nacional Autónoma de México, Ciudad Universitaria, Av. Universidad 3000, Coyoacán, 04510, Cd. Mx., Mexico Universidad Nacional Autónoma de México Mexico City Mexico

**Keywords:** Anatomy, distribution, Gramineae, grasses, identification, morphology, nomenclature, taxonomy

## Abstract

*Agrostis* is one of the most diverse genera of the Poaceae, including ca. 198 species, principally distributed in cold and temperate regions of the world, but also found in the high mountains of the tropics. We present a revision based on morphoanatomical evidence, for the biogeographic region known as Megamexico 3 (i.e., Mexico including the desert areas of southern USA and the Central America territory, to northern Nicaragua). We include taxonomic descriptions and an identification key for the found taxa, maps with the known geographical distribution of the species, and figures with the morphoanatomical characteristics, elevation and phenology. *Agrostis* is represented in the study zone by 20 species, of which four are endemic and three are introduced. Most records of the genus are distributed in the mountains, above 1500 m a.s.l., in open areas of temperate forests, with conifers and *Quercus*. Specimens with spikelets occur year round, but most records occur during the wet season, in the months of July to October. We propose a preliminary conservation assessment for each species in the study zone, according to the International Union for Conservation of Nature categories: one with Deficient Data (DD), six as Endangered (EN), two as Vulnerable (VU), and 11 as Least Concern (LC).

## Abstract (es)

*Agrostis* es uno de los géneros más diversos de las Poaceae, con ca. 198 especies, principalmente distribuidas en regiones frías y templadas del mundo, aunque también se pueden encontrar en grandes elevaciones de los trópicos. En este trabajo se presenta una revisión basada en evidencia morfoanatómica para la región biogeográfica conocida como Megaméxico 3 (i.e., México incluyendo las áreas desérticas del sur de los EUA y el territorio de Centroamérica hasta el norte de Nicaragua). Se incluyen descripciones taxonómicas y una clave de identificación para los taxones encontrados, mapas con la distribución geográfica conocida de las especies, así como figuras con las características morfoanatómicas, elevación y fenología. *Agrostis* está representado en la zona de estudio por 20 especies, cuatro de las cuales son endémicas y tres son introducidas. La mayoría de los registros del género se distribuyen en regiones montañosas, por arriba de los 1500 de elevación, en áreas abiertas de bosques templados, con coníferas y *Quercus*. Es posible encontrar ejemplares con espiguillas durante todo el año, pero la mayoría de los registros ocurren durante la época lluviosa, durante los meses de julio a octubre. Se propone una evaluación preliminar de conservación para cada especie en la zona de estudio, de acuerdo con las categorías de la Union Internacional para la Conservación de la Naturaleza: una con Datos Deficientes (DD), seis como Amenzadas (EN), dos como Vulnerables (VU) y 11 de Preocupación Menor (LC).

## ﻿Introduction

The genus *Agrostis* L. includes ca. 198 species (Soreng et al. 2022), principally distributed in cold and temperate regions of the world, but also found in the high mountains of the tropics. The genus belongs to the subfamily Pooideae Benth., tribe Poeae R. Br., and subtribe Agrostidinae Fr. (Soreng et al. 2022). *Agrostis* species are characterized by the usually fragile habit of the plants, synflorescences of the panicle type, spikelets one-flowered, floret notably shorter than the glumes, usually 1/3–3/4 the length of the glumes, rarely longer, lemma with usually five nerves, palea often reduced or absent, and rachilla prolongation absent. Some of the species are forage plants of regular to excellent quality (Mejía-Saulés and Dávila 1992), while others are agricultural weeds, and some are excellent lawn grasses in cool climates (Harvey 2007).

The systematics of *Agrostis* has been considered as challenging, since the limits between this and other genera remain poorly understood, and there are several species complexes where the limits between taxa are difficult to establish due to morphological variation. Attempts at infrageneric classifications have been made in the past, based on the habit of the plants, presence of awns in the glumes and lemmas, and the presence of transversal thickenings in the outer walls of the lemma epidemal cells (called the “*Trichodium* net”), but some authors indicate that the established groups are unnatural (e.g. Clayton and Renvoize 1986). Several authors recognize at least two subgenera, on the basis of the absence (A.subg.Vilfa (Adans.) Rouy) or presence of paleas (A.subg.Agrostis). Peterson et al. (2020) provide a molecular phylogeny of some European species of *Agrostis* and allied genera, where two clades that correspond with these two groups are recovered. Studies of the genus that incorporate several lines of evidence are scarce, but some outstanding works are the ones of Björkman (1960), Carlbom (1967), Widén (1971) and Romero-García et al. (1988). Recent studies based on molecular evidence include some species of *Agrostis*, showing that the sampled species form a well-supported clade, including species of *Bromidium* Nees & Meyen, *Chaetopogon* Janch, *Lachnagrostis* Trin., and *Polypogon* Desf. (Saarela et al. 2010, 2017; Tkach et al. 2020; Orton et al. 2021). Despite the existence of previous studies, the systematic knowledge of *Agrostis* has been far from ideal for decades, urging a worldwide revision of the genus (e.g., McVaugh 1983; Pohl and Davidse 1994).

**Figure 1.**
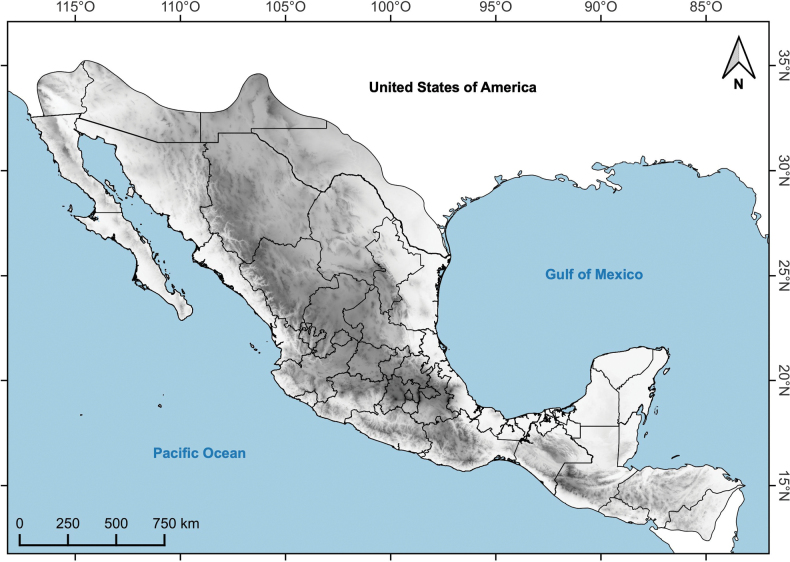
Area of Megamexico 3.

Mexico is considered as a mega-diverse country, since 10–12% of the known species across the globe inhabit its territory (Llorente and Ocegueda 2008). In this country, ca. 23,314 native species of vascular plants have been reported, and a little more than half are endemic, which places Mexico in second position for endemism, after South Africa (Villaseñor 2016). Rzedowski (1991), based on the geographic affinities and endemism of the phanerogamic flora of Mexico, proposed three phytogeographic areas, which extend beyond the political limits of the country as follows: 1) Megamexico 1, which includes the Sonoran, Chihuahuan and Tamaulipan desert areas of the southern USA; 2) Megamexico 2, which includes the Centroamerican territory up to northern Nicaragua; 3) Megamexico 3, which includes the latter two (Fig. 1). This region has been recognized as a biodiversity hotspot and unique in its complex geology, orography, and its climatic heterogeneity (Rzedowski 1991; Ferrusquía-Villafranca 1993; García 2004).

One of the first taxonomic studies of *Agrostis* in Megamexico 3 was the work of Fournier (1886), where several species that are still accepted were described. Another pioneering work was from Hitchcock (1905), who studied the taxonomy of North American species of *Agrostis*. Years later, the same author provided a synopsis of the Mexican species of the genus (Hitchcock 1913). As part of North American Flora, Hitchcock (1937) updated and expanded his 1905 work. Several decades later, Beetle et al. (1983) provided a taxonomic revision of the Mexican species, but this work contains some errors and omissions (see Vigosa-Mercado 2022b), and since its publication, several of the included species have been transferred to other genera and new records have been made.

Other relevant works for *Agrostis* in Megamexico 3 are the catalogs of Mexican Poaceae species by Espejo-Serna et al. (2000), Dávila et al. (2006, 2018), Villaseñor (2016) and Sánchez-Ken (2019). The genus has also been studied in state catalogs and regional floras, including the states of Chihuahua (Herrera-Arrieta and Peterson 2018), Coahuila (Valdés-Reyna 2015), Durango (Herrera-Arrieta 2001), Morelos (Sánchez-Ken and Cerros-Tlatilpa 2016), Oaxaca (Pacheco et al. 2012), and Zacatecas (Herrera-Arrieta et al. 2010), as well as the regions of the Bajío (Vigosa-Mercado and Ruiz-Sánchez 2020), Nueva Galicia (McVaugh 1983), Valley of Mexico (Acosta 2001) and the Valley of Tehuacán-Cuicatlán (Vigosa-Mercado 2017). Other works that partially cover Megamexico 3 are those of Harvey (2007), which is part of the Flora of North America North of Mexico, and Pohl and Davidse (1994), as part of the Flora Mesoamericana. In this study, we provide an updated review of the morphoanatomy, taxonomy, distribution, and conservation of *Agrostis* in Megamexico 3.

## ﻿Materials and methods

### ﻿Taxonomic study

Herbarium specimens of *Agrostis* and related genera from the following collections were examined on both physical and digital repositories: ASU, CHAPA, CIIDIR, ENCB, F, FCME, HUMO, IBUG, IEB, INEGI, MEXU, MO, NY, SD, TEX, UAMIZ, US and XAL (abbreviations according to Thiers 2022). Digitalized specimens of the following repositories were also examined: Consortium of California Herbaria (CCH2 Portal 2022), SEINet (2022), Texas Oklahoma Regional Consortium of Herbaria (TORCH 2022), and the Portal de Biodiversidad de Guatemala [Biodiversity Portal of Guatemala] (2022). Available protologues and type specimens were examined.

The analysis of the specimens consisted of: 1) verification of their correct identification, 2) correction of erroneous determinations, 3) updating of the names in synonymy, 4) identification of unnamed specimens, 5) taking measurements of vegetative and reproductive structures for descriptions, and 6) integration of information of the specimens in a database. The synonyms of some of the species are too extensive, whereby only the names with types collected in the study zone, or names widely used in the literature, are cited in the main text. A list of other heterotypic synonyms is provided in Suppl. material 1. Descriptions are based on the examined specimens from the study zone, but measurements reported in the literature from other regions (e.g., Harvey 2007), are included as comments, below the species descriptions. The descriptions of species with few herbarium specimens were complemented with information from the literature. Below the description of each species, one to three representative specimens per state are cited. A list of additional examined specimens is provided in Suppl. materials 2, 3.

### ﻿Anatomical and micromorphological study

Samples of mature leaves and florets were taken from herbarium specimens, with prior authorization, except for species of which no physical herbarium specimens were seen, as *A.idahoensis* Nash. For each species, a variable number of specimens was studied according to the availability of material. Specimens studied for leaf anatomy are marked with one asterisk (*), and specimens studied for floret micromorphology are marked with two asterisks (**) in the lists of examined specimens.

Leaf blade samples were rehydrated with hot water for 1–5 minutes. The abaxial epidermis was isolated by removing the adaxial epidermis and underlying tissues with a razor blade. Hand transversal sections were taken with a razor blade from the upper third of the leaf blades. The isolated epidermis and transversal sections were treated with 6% sodium hypochlorite solution to clarify and soften the tissues, then washed with water. Only the transversal sections were stained with 1% safranin, and washed again. Both kinds of preparations were mounted with glycerin gelatin. For the description of the anatomical characteristics, the terminology proposed by Ellis (1976, 1979) was used with some modifications.

Floret samples were mounted in aluminum sample holders with conductive carbon tape, exposing the abaxial surface of the lemma, and covered with a layer of gold. The observation and taking of digital photographs were carried out in a Hitachi SU1510 scanning electron microscope.

### ﻿Elevation and phenology histograms

Histograms of elevation and phenology were drawn in Microsoft Excel (Microsoft Corporation 2022), using the date of collection of the herbarium specimens, excluding duplicates. Phenology histograms represent both the presence of flowers and fruits, since spikelets in a synflorescence contain both kind of structures during the reproductive season. The Y axis in the graphs represents the proportion of the occurrences.

### ﻿Distribution maps and conservation assessment.

Maps of known geographic distribution of *Agrostis* species, are based on the herbarium specimen data. Herbarium specimens examined were georeferenced by using either Google Earth online or Mapa digital de Mexico V.6 [Digital Map of Mexico] (INEGI 2022). Maps were drawn using QGIS 3.22 (QGIS.org 2022). In the lists of examined specimens, coordinates georeferenced for the purposes of this work appear in square brackets. Records of distribution of *Agrostis* species in the study zone that we were unable to confirm, are discussed under each species description. These records were taken from several references (e.g., Villaseñor 2016; Dávila et al. 2018; Sánchez-Ken 2019), where no examined specimens are cited.

A preliminary conservation assessment in the study zone was proposed for each species, according to the International Union for Conservation of Nature categories and criteria B (IUCN 2012), as well as other sources of information, such as the size of populations indicated in the specimen labels, number of collections, and the presence of records in protected areas. The Extent of Occurrence (EOO), defined as the area contained within the shortest continuous imaginary boundary, which can be drawn to encompass all the known sites of present occurrence of a taxon, and the Area of Occupancy (AOO), defined as the area within the extent of occurrence which is occupied by a taxon (IUCN 2012), were calculated in km^2^, using the data from localities, in the R package ConR (Dauby 2020), assuming cells of 2 km per side.

### ﻿Species concept and delimitation

In this work, the cohesive species concept (Templeton 1989) was followed as an explanatory hypothesis to recognize species and infraspecific categories. This concept has a population genetics framework, but does not discard other cohesive factors to explain species or infraspecific taxa recognition, such as the expression of morphology (phenotypic variability constraints on individuals in the group) and habitat distinctiveness (geographic distribution and ecological constraints). Evolutionary processes act on these cohesive factors to maintain populations or generate new taxa. Species recognized here are characterized by a combination of characters, evaluated in most cases in a broad study of herbarium specimens and the individuals in the field, as well as the known distribution of the organism.

## ﻿Results

### ﻿Diversity, elevation, habitat, and phenology

The genus *Agrostis* is represented in Megamexico 3 by 20 species (Table 1), of which four are endemic and three are introduced. We include a list of 16 excluded names of *Agrostis*, previously reported in the study zone, at the end of the work. The found species are distributed mainly in the mountains of southwestern USA, Mexico and Central America, between 14–4,520 m a.s.l. (Fig. 9A). The mean elevation of the records is 2,544.5 m, and the median is 2,592 m. The species grow in a wide range of habitats, such as grasslands, shrublands, stream edges, and temperate forests. Most records of native species of *Agrostis* are distributed above 1,500 m a.s.l. (Fig. 9B), usually in open areas of temperate forests, with conifers and *Quercus*. Some species, such as *Agrostismicrophylla* (Fig. 9N), *A.pallens* (Fig. 9O), as well as the populations in the southwestern USA of *A.exarata* (Fig. 9H), *A.hyemalis* (Fig. 9K), *A.perennans* (Fig. 27A), and *A.scabra* (Fig. 27B), are distributed at lower elevations, often in drier environments. Introduced species are distributed between 651–3300 m a.s.l. (Fig. 9C) and seem to prefer disturbed habitats. They are found mainly in moist soils of ditches, marshy habitats, and stream edges. Specimens with flowers and fruits occur all year round, but most records are during the wet season (Fig. 10A), between the months of July to October. Records of native species of *Agrostis* show the same pattern (Fig. 10B), while specimens of introduced species are found during the months of June to December and no specimens of introduced species were found from January to May (Fig. 10C). Elevation, habitat, and phenology for each species are discussed under each species description.

**Table 1.** Distribution of the species of *Agrostis* in Megamexico 3 and conservation status.

Species	Distribution in the study zone	Elevation (m a.s.l.)	EOO (km^2^)	AOO (km^2^)	Conservation status
*A.bourgaei* E. Fourn.	Endemic of central Mexico	1800–3800	45,765	1,476	LC
*A.calderoniae* Acosta	Endemic of central Mexico	3500–3800	–	–	EN
*A.capillaris* L.	Introduced to Mexico and Honduras	2000–2830	–	–	LC
*A.elliottiana* Schult.	Southwestern USA (Arizona, New Mexico)	1189–1676	1,078	16	EN
*A.exarata* Trin.	Southern USA to central Mexico	350–2900	1,402,821	188	LC
*A.ghiesbreghtii* E. Fourn.	Endemic. Central Mexico to Guatemala	1110–3700	240,863	176	LC
*A.gigantea* Roth	Introduced to USA and Mexico	651–3300	–	–	LC
*A.hyemalis* (Walter) Britton, Sterns & Poggenb.	Southern USA to central Mexico	14–2710	1,491,700	248	LC
A.idahoensis Nash	Southwestern USA (Arizona, California)	3084–3121	–	–	DD
*A.laxissima* Swallen	Endemic. Southern Mexico to Guatemala	2250–3800	2,615	44	VU
*A.microphylla* Steud.	Baja California peninsula	37–315	897	12	EN
*A.pallens* Trin.	California to Baja California	40–1635	10,902	48	VU
*A.perennans* (Walter) Tuck.	Northern Mexico to Honduras	622–3847	992,915	700	LC
*A.scabra* Willd.	Southern USA to Guatemala	630–3500	2,076,712	712	LC
*A.stolonifera* L.	Introduced to Mexico	1800–2100	–	–	LC
*A.subpatens* Hitchc.	Southern Mexico to Guatemala	2900–3790	5,589	20	EN
*A.subrepens* (Hitchc.) Hitchc.	Mexico (Chihuahua)	2000–2168	74	12	EN
*A.tolucensis* Kunth	Northern Mexico to Guatemala	1330–4520	439,417	432	LC
*A.turrialbae* Mez	Central Mexico to Guatemala	1600–4240	183,426	84	LC
*A.variabilis* Rydb.	California to Baja California	2400–2639	1,621	16	EN

### ﻿Distribution

Distribution of most found species extend beyond the study zone, and there are several main patterns of the native species: 1) species widely distributed in the Americas (*A.hyemalis*, *A.perennans*, *A.scabra*); 2) species widely distributed in North America, with a southern distribution limit in southwestern USA (*A.elliottiana*, *A.idahoensis*); 3) species distributed from Alaska to central Mexico (*A.exarata*); 4) species distributed from Canada to Baja California peninsula (*A.microphylla*, *A.pallens*, *A.variabilis*); 5) species distributed from northern Mexico to South America (*A.tolucensis*); 6) species distributed from central or southern Mexico to Central America (*A.ghiesbreghtii*, *A.laxissima*, *A.subpatens*, *A.turrialbae*); and 7) species distributed in central Mexico (*A.bourgaei*, *A.calderoniae*). *Agrostissubrepens* is an outlier by its disjunct distribution in Chihuahua, Mexico, and South America. Table 1 shows the distribution of the species in Megamexico 3. Distribution for each species is discussed under each species description.

### ﻿Taxonomy

The two subgenera of *Agrostis* are represented in the study zone. The three introduced species belong to subgenus Vilfa (*A.capillaris*, *A.gigantea*, *A.stolonifera*), characterized by the stoloniferous or rhizomatous habit of the plants, spikelets with a usually well-developed palea, reaching (1/5–)1/3–3/4 of the lemma length, and epidermal cells of the lemmas without transversal thickenings. The remaining species belong to subgenus Agrostis, characterized by the usually caespitose habit of the plants, spikelets with usually reduced palea, less than 1/5 of the lemma length, and epidermal cells of the lemmas with transversal thickenings.

In the subgenus Agrostis, there are several informal groups of morphologically similar species, where the taxonomy is often complex, since the differences between the species are often subtle and intermediate forms are common. However, through a combination of characters, a reasonably good separation can be made. These groups are: 1) species with usually open panicles and usually awnless lemmas (*A.bourgaei*, *A.calderoniae*, *A.ghiesbreghtii*, *A.hyemalis*, *A.laxissima*, *A.idahoensis*, *A.perennans* sensu lato, *A.scabra*, *A.subrepens*, *A.turrialbae*); 2) species with usually narrow and dense panicles, and often acuminate to awned glumes (*A.exarata*, *A.microphylla*, this group is called the *A.exaratacomplex* by Carlbom (1967)); 3) species with mostly basal leaves, usually conduplicated to convolute leaf blades, and usually narrow and dense panicles (*A.subpatens*, *A.tolucensis*, *A.variabilis*). Particularly challenging is the taxonomy of *Agrostisperennans* sensu lato (see Sylvester et al. 2020a), where numerous entities have been identified under this name. There is still much work to do on the taxonomy of *Agrostis*, and molecular evidence may help in the systematics of the group (Vigosa-Mercado in prep.).

### ﻿Conservation status

We propose a preliminary conservation assessment for each species in the study zone (Table 1), according to the International Union for Conservation of Nature categories: one with Deficient Data (DD), six as Endangered (EN), two as Vulnerable (VU), and 11 as Least Concern (LC). Values of EOO and AOO for each species are presented at Table 1. The mean for the EOO is 459,760 km^2^, and for the AOO is 279 km^2^. Endangered species are reported as scarce, with few plants per population, and usually are known only from one or two localities in the study zone. Vulnerable species are known from a greater number of localities. Species assessed as Least Concern are widespread, and often are reported as abundant in the populations. Introduced species often are weedy and are included in this latter category. The conservation status for each species is discussed under each species description.

### ﻿Morphoanatomy

A general morphoanatomical characterization of the species found in the study zone is presented below. Description of the morphology and anatomy of each species is presented in the taxonomic treatment.

**Habit.** All the species of *Agrostis* in the study zone are herbaceous plants, usually perennial, but some annual species occur (*A.elliottiana*, *A.microphylla*). Most species are caespitose, with the culms growing densely in tufts, some species develop stolons (*A.stolonifera*, Fig. 33A; rarely *A.capillaris* and *A.gigantea*), rhizomes (*A.capillaris*, *A.capillaris*, *A.gigantea*, *A.tolucensis*, rarely *A.exarata*), or pseudostolons that have the appearance of rhizomes on herbarium specimens (*A.perennans* sensu lato, *A.subrepens*, Fig. 35B). Usually, the species in the study zone develop extravaginal tillers that are covered with cataphylls. While the type of tillering has been considered as a character of taxonomic importance (Philipson 1937; Widén 1971), this character is difficult to observe on herbarium specimens.

**Roots.** They are fibrous, and the presence of arbuscular mycorrhiza has been reported in *Agrostiscapillaris* (Gollotte et al. 2004).

**Culms.** They are unbranched, hollow, and usually erect, but sometimes are decumbent in the lower portion. The length of the culms is variable, from a few centimeters to 1.2 meters in some species (*A.gigantea*). The number of nodes is variable, from one to several.

**Leaves.** They are formed by the sheath, leaf blade and ligule. They could be basal (Fig. 38A), cauline (Fig. 24A), or both, as in most species of Megamexico 3.

**Sheaths.** They are tubular. Their margins are free and clasp the culms. In the species of the study zone, the sheaths range from shorter to longer than the internodes, and their abaxial surface are glabrous or scaberulous.

**Ligules.** The length and form of the ligules have been recognized as a valuable taxonomic character (Philipson 1937; Widén 1971; Romero-García et al. 1988). In the species of the study zone, the ligules are variable, but in most cases no significant differences were found between the species. The ligules range from longer or shorter than wide, their abaxial surfaces are usually scaberulous, and the apices are acute, erose or truncate. In several species of Megamexico 3, the old ligules are often lacerate (e.g., *A.bourgaei*, Fig. 3B).

**Leaf blades.** In the species of the study zone, leaf blades are filiform (e.g., *A.subpatens*, Fig. 34A) to linear (e.g., *A.perennans* sensu lato, Fig. 25A), and their consistency is usually chartaceous.

**Leaf blade abaxial epidermis.** Among the species in the study zone, the abaxial epidermis is quite homogeneous and thus its taxonomic value is poor, as previously noted by Romero-García et al. (1988).

**Figure 2.**
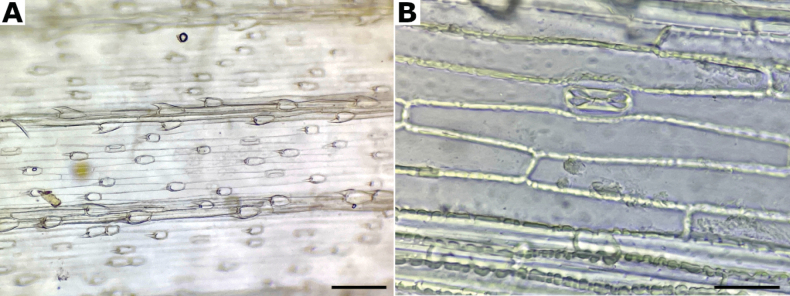
Abaxial epidermis of *Agrostis* species **A***A.bourgaei***B***A.exarata*. Scale bars: 100 μm (**A**); 50 μm (**B**).

The abaxial epidermis presents two differentiated regions, the intercostal and costal zones (Fig. 2A). The intercostal zone is made up of elongated cells of constant shape and size, their walls are straight, the sidewalls are angled outwards, and end walls are vertical or angled (Fig. 2B). In the intercostal zone there are one or two rows of stomata (Fig. 2A), with guard cells parallel-sided or low dome-shaped. No short cells were seen in the intercostal zone of the studied species. The costal zone presents elongated cells similar to the intercostal zone, but sometimes their walls are slightly thickened, with short cells (Fig. 2B), and silica bodies. Prickle hairs are present on both zones (Fig. 2A). They are medium sized, with a short and raised barb. No micro or macrohairs were seen in the abaxial epidermis of the studied species.

**Leaf blade transversal section anatomy.** The leaf blades of *Agrostis* species display a no-Kranz anatomy (C3 photosynthesis). The anatomical features of the leaf blade have been considered of taxonomic importance, particularly the patterns of sclerenchyma distribution (Romero-García et al. 1988). In the studied species, leaf blade anatomy is a useful character for distinguishing some species, but several species share similar attributes.

The leaf blade could be flat (Fig. 6A), convolute (15D), or v-shaped, (37A) in transversal section. Adaxial furrows of variable depth and width are present between all vascular bundles (Figs 6, 15, 22, 31, 37). Adaxial ribs of variable shape are present over all vascular bundles (Figs 6, 15, 22, 31, 37). No abaxial furrows and ribs were seen in the studied species.

A keel with several vascular bundles and parenchyma was found only in some individuals of *A.exarata* (Fig. 15A). There are one to three vascular bundles of second order between the bundles of first order, where all of them are at the same level, closer to the abaxial surface (Figs 6, 15, 22, 31, 37), and their shape is circular (Fig. 6B) to slightly elliptical (Fig. 31B). Sometimes there are third order vascular bundles, between the first and second order bundles, or near the leaf blade margins (Figs 6D, 22F). There are two sheaths surrounding the vascular bundles, an outer parenchymatous sheath that is interrupted abaxially (Fig. 15B), or sometimes also adaxially (Fig. 31E), and an inner sclerenchymatous complete sheath (Fig. 15J). Sclerenchyma is found associated with vascular bundles of first and second order, in abaxial or adaxial strands or girders (Figs 6, 15, 22, 31, 37). There are small caps of sclerenchyma in leaf margins (Fig. 22F). In some species, abaxial strands of sclerenchyma between the vascular bundles are found (Fig. 6E), and only in some individuals of *A.ghiesbreghtii* a hypodermal abaxial layer of sclerenchyma is detected (Fig. 15D, E).

The mesophyll is non-radiate, with regular small cells, isodiametric, and tightly packed (Fig. 6G). Colorless cells were found associated with the outer bundle sheath, only in some individuals of *A.exarata* (Fig. 15A). In all species, there are fan-shaped groups of small, bulliform cells in the base of adaxial furrows (Fig. 6E), but sometimes in dried specimens they could be collapsed. In all species, there are prickle hairs in adaxial and abaxial epidermis.

**Synflorescences.** They are made up of spikelets disposed in panicles, contracted (e.g., *A.microphylla*, Fig. 23A) to open (e.g., *A.perennans* sensu lato, Fig. 25A). The shape of the panicles could be linear (e.g., *A.variabilis*, Fig. 40A), or lanceolate (e.g., *A.exarata*, Fig. 14A) to ovate (e.g., *A.ghiesbreghtii*, 16A). The branches are disposed in verticils and could be appressed (e.g. *A.stolonifera*, Fig. 33A) to divergent (e.g., *A.scabra*, Fig. 29A), with or without spikelets from the base, or sometimes the spikelets are clustered at branch tips (e.g., *A.hyemalis*, Fig. 19C).

**Figure 3.**
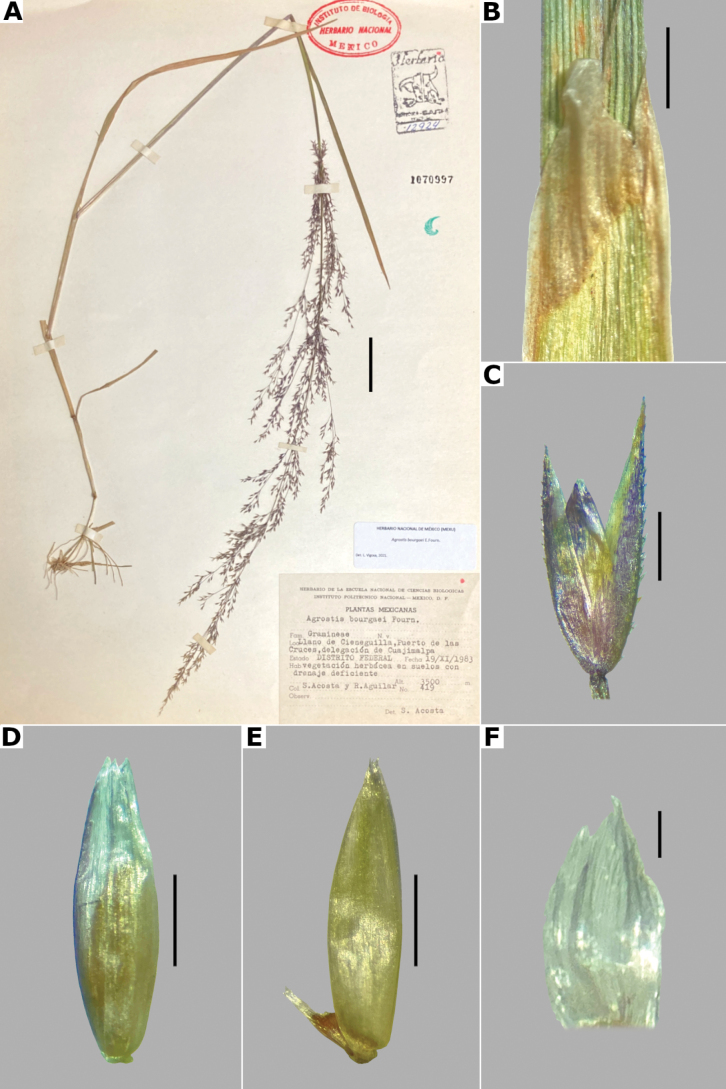
*Agrostisbourgaei***A** whole plant **B** ligular area **C** spikelet **D** floret, abaxial view, **E** floret, lateral view showing the lemma and the palea **F** palea. Based on Acosta 419 (MEXU). Scale bars: 3 cm (**A**); 2 mm (**B**); 0.5 mm (**C–E**); 0.1 mm (**F**).

**Spikelets.** They are small, no more than 4.5 mm long. They are made up of two basal bracts called glumes and a single floret (Fig. 3C). The florets consist of a lower bract called the lemma, an upper bract called the palea, and a bisexual flower (Fig. 3E). The base of the floret is slightly hardened (callus), usually with two tufts of hairs (Figs 5, 30).

**Glumes.** They are longer than the floret, equal to unequal in size between them and keeled on the back. Their apices are usually acute to acuminate (Fig. 14C), or sometimes awned (*A.microphylla*, Fig. 23C; sometimes *A.exarata*). Their consistency is membranous, with a single vein, and are scaberulous on the keel (Fig. 19D), and sometimes in the rest of the body. It has been considered that the glumes do not have characters of taxonomic value (Widén 1971).

**Figure 4.**
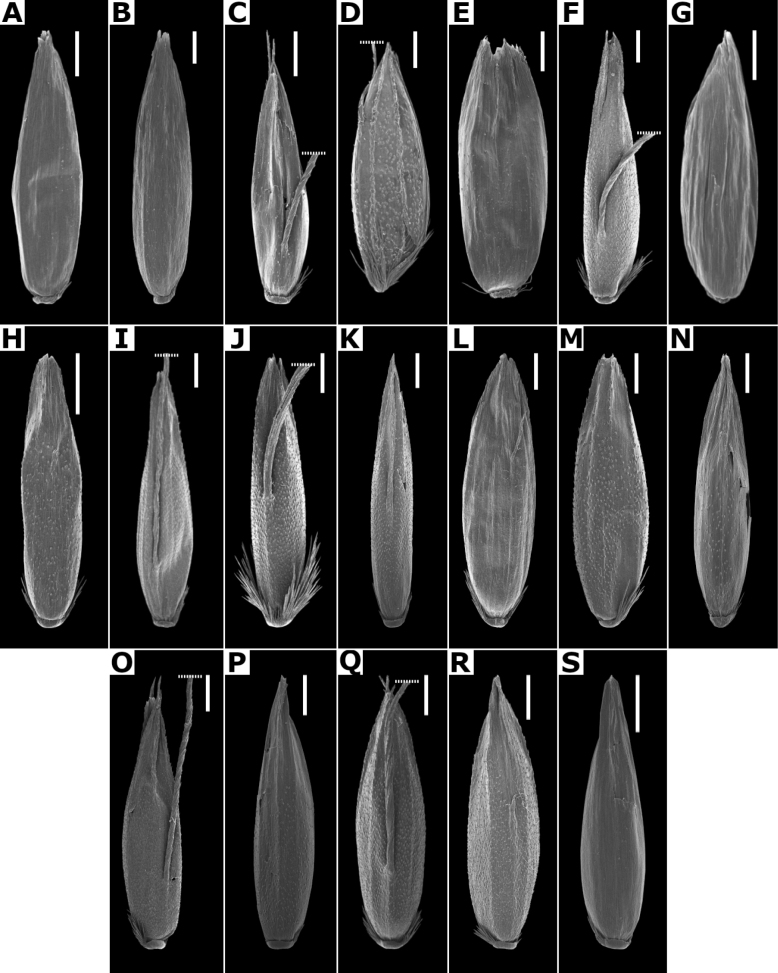
Florets of *Agrostis* species observed with SEM **A***A.bourgaei***B***A.calderoniae***C***A.capillaris***D***A.elliottiana***E***A.exarata***F***A.ghiesbreghtii***G***A.gigantea***H***A.hyemalis***I***A.laxissima***J***A.microphylla***K***A.pallens***L***A.perennans***M***A.scabra***N***A.stolonifera***O***A.subpatens***P***A.subrepens***Q***A.tolucensis***R***A.turrialbae***S***A.variabilis*. Scale bars: 0.3 mm.

**Lemmas.** They have several characters of taxonomic importance (Widén 1971; Romero-García et al. 1988). The lemmas are elliptical to oblong, rounded on the back, and their apices could be acute (e.g., *A.gigantea*, Fig. 4G), truncate to obtuse (e.g., *A.hyemalis*, 4H), toothed (e.g., *A.tolucensiss*, Fig. 4Q), or sometimes the lateral veins are shortly excurrent (e.g., *A.capillaris*, Fig. 4C). Their consistency is membranous, usually with five veins that are prominent on the back, or inconspicuous. They are often awned on the back, with the awn inserted near the base (e.g., *A.ghiesbreghtii*, Fig. 4F), or near the apex, and could be straight to geniculate (e.g., *A.laxissima*, Fig. 21D).

**Lemma micromorphology.** Transversal thickenings in the outer walls of the abaxial epidermis cells, especially the ones of the lower half portion, could form a pattern that has been called the *Trichodium* net. It has been noted that this character is useful to form broad groups, and it has been proposed that it is an important character for the infrageneric classification of the genus (Björkman 1960; Widén 1971; Romero-García et al. 1988).

Widén (1971) has recognized seven types of net, based on the width of the transversal thickenings. In the studied species of *Agrostis*, taxa with absent or reduced paleas have a conspicuous net, while the species with paleate spikelets usually lack, or have fragmentary nets (Figs 7, 32), as noted in previous studies (Björkman 1960; Widén 1971; Romero-García et al. 1988). In the species of *Agrostis* present in Megamexico 3, it has also been found that this character is useful for distinguishing some morphologically similar species that are often confused (e.g., *A.bourgaei* and *A.gigantea*).

**Paleas.** They are usually minute, membranous, veinless, and often are missing. In some species, the paleas are well-developed, reaching (1/5–)1/3–3 /4 of the lemma length, usually with two visible veins (e.g., *A.capillaris*, Fig. 12F).

**Flowers.** They consist of a perianth with two scales (lodicules), an androecium of three stamens (one in *A.elliottiana*), and a unilocular gynoecium with two styles and two plumose stigmas.

**Fruits.** They are of the caryopsis type, usually ellipsoidal, with the surface smooth, and have a punctiform hilum. The endosperm is liquid, soft, or solid. The embryo is small in relation to the fruit size.

### ﻿Taxonomic treatment

#### ﻿ Agrostis

Taxon classificationPlantaePoalesPoaceae

L., Sp. Pl. 1: 61. 1753, nom. et typ. cons.

F6522B12-AEEF-5BDB-B388-C30FFF4A3C72

= Vilfa Adans., Fam. Pl. 2: 495. 1763. Type: Agrostis.stolonifera L. (lectotype designated by Hitchcock (1920: 127)).
= Trichodium Michx., Fl. Bor.-Amer. 1: 41. 1803. Type: T.laxiflorum Michx. (lectotype designated by Hitchcock (1920: 127)).
= Agraulus P. Beauv., Ess. Agrostogr. 5, t. 4. 1812. Type: Agrostiscanina L. (lectotype designated by Hitchcock (1920: 127)).
= Decandolia Bastard, Essai Fl. Maine et Loire 15, 28. 1809, nom. illeg. superfl. Type: Agrostisstolonifera L. (lectotype designated by Hitchcock (1937: 515)).
= Notonema Raf., Bull. Bot. (Geneva) 1: 220. 1830. Type: Agrostisarachnoides Elliott (=A.elliotiana Schult.).
= Bromidium Nees & Meyen, Nov. Actorum Acad. Caes. Leop.-Carol. Nat. Cur. 19, suppl. 1: 154. 1843. Type: Agrostishygrometrica Nees (lectotype designted by Rúgolo 1982: 196)).
= Anomalotis Steud., Syn. Pl. Glumac. 1: 198. 1854. Type: A.quinqueseta Steud.
= Didymochaeta Steud., Syn. Pl. Glumac. 1: 185. 1854. Type: D.chilensis Steud. (lectotype designated by Clayton and Renvoize (1986: 134)).
= Chaetopogon Janch., Eur. Gatt. Farn. Bl.-Pfl. (ed. 2) 33. 1913. Type: not designated.
= Neoschischkinia Tzvelev, Bot. Zhurn. (Moscow & Leningrad) 53: 309. 1968. Type: Trichodiumelegans Thore.
= Linkagrostis Romero Garcia & C. Morales, Candollea 42(1): 383. 1987. Type: Agrostisjuressi Link.^*^

##### Type.

*Agrostiscanina* L. (lectotype designated by Widén (1971: 13), see also Philipson (1937), Jarvis (1992) and commentary below) .

##### Description.

Plants herbaceous, annuals or perennials, up to 1.2 m, caespitose, rhizomatous or stoloniferous, sometimes developing pseudoestolons. ***Tillers*** extravaginal and/or intravaginal. ***Culms*** decumbent to erect, usually slender, unbranched, internodes hollow. ***Leaves*** basal or cauline; sheaths open; auricles absent; ligules membranous, entire to lacerate, usually scabridulous dorsally; blades filiform to linear, flat, conduplicate, convolute, or involute. ***Synflorescence*** a panicle, usually terminal, contracted to open; branches usually in whorls. ***Spikelets*** up to 4.5 mm long, 1-flowered, pedicellate, laterally compressed; rachilla not prolonged beyond the base of the floret; disarticulation above the glumes; glumes as long as the spikelet, equal to unequal, keeled, membranous, usually 1-nerved, rarely awned; floret bisexual, shorter than the glumes, usually 1/3–3/4 the length of the glumes, rarely longer; callus rounded, glabrous to pubescent; lemmas rounded dorsally, apices entire, erose or toothed, membranous, (3)5-nerved, veins inconspicuous or prominent, unawned or awned dorsally, awn straight to geniculate; paleas often absent or diminute, sometimes well-developed and reaching (1/5–)1/3–3/4 of the lemma length; lodicules 2; anthers (1)3; ovary glabrous, styles 2, free to the base. ***Fruit*** a caryopsis; endosperm liquid, soft or solid. x= 7.

##### Commentaries.

The name *Agrostis* was first described in the work of Linnaeus, Genera Plantarum (Linnaeus 1737: 19), with a Latin diagnosis. According to the International Code of Nomenclature for Algae, Fungi and Plants (Art. 13.1) (Turland et al. 2018), the beginning of effective publication dates for generic names is 1^st^ May 1753, with the publication of Species Plantarum (Linnaeus 1753). In this latter work, Linnaeus described 12 names, classified in two groups: 1) Aristatae (*A.spica-venti* L., *A.miliacea* L., *A.arundinacea* L., *A.rubra* L., *A.canina* L., *A.paradoxa* L.); and 2) Muticae (*A.stolonifera* L., *A.capillaris* L., *A.alba* L., *A.minima* L., *A.virginica* L., *A.indica* L.). No type species was designated by Linnaeus.

Hitchcock (1905) designated *A.alba* as the type, but the original material of this name is a *Poa* L. species (Widén, 1971), and the adoption of this name as lectotype would result in *Agrostis* becoming a synonym of *Poa* (McNeill et al. 1987). Years later, Hitchcock (1920), proposed *A.stolonifera* as lectotype, but this name conflicts with the original description of the genus, since it mentions that the lemmas are awned (Linnaeus 1737), and the original description of *A.stolonifera* mentions that the lemmas are unawned (Linnaeus 1753).

Philipson (1937) was the first to propose as lectotype the name *A.canina*. Widén (1971) agreed with that choice, and formally designated this name, since it is the one that keeps better the usage of the generic name. Jarvis (1992) proposed the conservation of the name *Agrostis*, with a conserved type.

The species of this genus are often confused with other genera found in the study zone, with one-flowered spikelets, such as *Lachnagrostis* Trin., *Muhlenbergia* Scherb., *Podagrostis* (Griseb.) Scribn. & Merr., *Polypogon* Desf., *Sporobolus* R. Br., and some species of *Peyritschia* E. Fourn., but it is distinguished from them by the following combination of characters: spikelets disarticulating above the glumes, florets shorter than the glumes, usually 1/3–3/4 the length of the glumes, rarely longer, lemmas with usually five veins, dorsal awns often present, palea often reduced or absent (sometimes reaching up to 3/4 of the lemma length), and rachilla not prolonged. See the work of Sylvester et al. (2020b) for a key to differentiate *Agrostis* from morphologically similar genera.

### ﻿Identification key for the species of *Agrostis* of Megamexico 3

**Table:** 

1	Paleas present, 0.5 mm or longer ((1/5–)1/3–3/4 of the lemma length)	**2**
–	Paleas absent or diminute, up to 0.2(–0.4) mm long (usually less than 1/5 of the lemma length)	**8**
2(1)	Panicles 0.4–3 cm wide, contracted, dense, often spiciform, branches appressed to ascending	**3**
–	Panicles (2–)3.5–20 cm wide, open, dense to lax, not spiciform, branches ascending to spreading	**4**
3(2)	Plants caespitose, rarely shortly rhizomatous; paleas up to 0.8 mm long, veinless; anthers 0.3–0.7 mm long	***A.exarata* Trin**.
–	Plants stoloniferous; paleas 0.7–1.4 mm long, 2-veined; anthers 0.9–1.5 mm long	***A.stolonifera* L.**
4(2)	Plants caespitose	**5**
–	Plants rhizomatous or stoloniferous	**6**
5(4)	Spikelets 2–2.7 mm long; lemmas 1.5–1.8 mm long, anthers (0.3–)0.5–0.7 mm long; leaves basal and cauline, blades flat	***A.bourgaei* E. Fourn.**
–	Spikelets 2.5–3.5 mm long; lemmas 2–2.5 mm long; anthers 0.7–1 mm long; leaves mostly basal, conduplicate to convolute	***A.calderoniae* Acosta**
6(4)	Ligules of the basal blades 0.2–1.5 mm long, the upper ones up to 2 mm long, usually shorter than wide, rarely longer than wide	***A.capillaris* L.**
–	Ligules 1–7 mm long, longer than wide	**7**
7(6)	Plants rhizomatous, rarely stoloniferous; culms up to 1.2 m long; panicles (9–)13–40 cm long, 4–16 cm wide, open; blades 1–8 mm wide, usually at least some blades larger than 5 mm wide	***A.gigantea* Roth**
–	Plants stoloniferous, stolons usually long; culms up to 0.6 m long; panicles 4–20 cm long, 0.5–3 cm wide, open at anthesis, becoming contracted after flowering; blades (1–)2–6 mm wide	***A.stolonifera* L.**
8(1)	Panicles 0.2–3 cm wide, contracted, usually dense, spiciform, branches appressed to ascending	**9**
–	Panicles 0.5–30 cm wide, usually open and lax, if narrow never spiciform, branches ascending to spreading	**15**
9(8)	Plants annual; glumes with apices long acuminate or awned; lemma awned, awn 3.5–6 mm long	***A.microphylla* Trin.**
–	Plants perennial; glumes usually with apices acute or shortly acuminate (sometimes awned in *A.exarata*); lemmas unawned or with an awn up to 3.5 mm long	**10**
10(9)	Plants rhizomatous, rhizomes up to 10 cm long; leaves mostly cauline; plants known in the study zone from inland San Diego and Baja California	***A.pallens* Trin.**
–	Plants caespitose, or shortly rhizomatous, rhizomes if present inconspicuous, or up to 2 cm long; leaves mostly basal or basal and cauline	**11**
11(10)	Leaf blades 0.5–4(–8) mm wide, linear, flat	**12**
–	Leaf blades 0.4–1 mm wide, filiform, conduplicate or convolute (flat when young in *A.variabilis*)	**13**
12(10)	Lemmas unawned or awned about mid-length, awn up to 3 mm long; paleas absent or 0.3–0.8 mm long	***A.exarata* Trin**.
–	Lemmas usually awned near the base, sometimes above mid-length, rarely awnless, awn 1.5–3.5 mm long; paleas absent	***A.tolucensis* Kunth**
13(10)	Lemmas with apices entire, acute, usually unawned, rarely awned above mid-length, awn up to 1 mm long, straight, not reaching the lemma apices; plants known in the study zone from Baja California	***A.variabilis* Rydb.**
–	Lemmas with apices toothed, usually awned near the base (sometimes above mid-length or rarely awnless in *A.tolucensis*), awn 1.5–3.5 mm long, geniculate, reaching the lemma apices	**14**
14(13)	Panicles somewhat lax; most pedicels longer than the spikelets; plants known in the study zone from Chiapas and Guatemala	***A.subpatens* Hitchc.**
–	Panicles dense; most pedicels shorter than the spikelets	***A.tolucensis* Kunth**
15(8)	Plants rhizomatous or developing conspicuous pseudostolons	**16**
–	Plants caespitose (pseudostolons sometimes present in *A.perennans* sensu lato)	**17**
16(15)	Panicles 0.4–3 cm wide, open to contracted, branches appressed to ascending; leaf blades 1–4 mm wide; plants known in the study zone from inland San Diego and Baja California	***A.pallens* Trin.**
–	Panicles 5–10 cm wide, open, branches spreading; leaf blades 1–1.5 mm wide; plants known in the study zone from Chihuahua	***A.subrepens* (Hitchc.) Hitchc.**
17(15)	Lemmas awned, awn 2–10 mm long, inserted near the base (inserted in the upper third in *A.elliottiana*)	**18**
–	Lemmas unawned, rarely with an awn up to 2.5 mm long, inserted above mid-length	**21**
18(17)	Plants annual; awn of the lemma 3–10 mm long, inserted in the upper third, flexuous; anther 1	***A.elliottiana* Schult.**
–	Plants perennial; awn of the lemma 2.3–5 mm long, inserted near the base, geniculate; anthers 3	**19**
19(18)	Culms with 1–2 nodes; leaves mostly basal, blades 0.4–0.8 mm wide, filiform	***A.subpatens* Hitchc.**
–	Culms with 2–6 nodes; leaves basal and cauline, blades 1–4(–5) mm wide, linear	**20**
20(19)	Leaf blades stiff, convolute, the upper ones sometimes flat; spikelets (2.5–)3–4 mm long	***A.ghiesbreghtii* E. Fourn.**
–	Leaf blades lax, flat; spikelets 1.7–3 mm long	***A.laxissima* Swallen**
21(17)	Leaves basal and cauline (the basal ones often drying before anthesis in mature individuals of *A.perennans* sensu lato)	**22**
–	Leaves mostly basal	**24**
22(21)	Branches of the panicle rebranching about mid-length; spikelets not clustered; leaf blades 1–6 mm wide, usually at least some blades larger than 2 mm wide	***A.perennans* (Walter) Tuck. sensu lato**
–	Branches of the panicle rebranching in the upper third; spikelets clustered at the branch tips; leaf blades (0.3–)0.5–2(–3) mm wide	**23**
23(22)	Spikelets 1–2(–2.5) mm long; anthers 0.2–0.5 mm long	***A.hyemalis* (Walter) Britton, Sterns & Poggenb.**
–	Spikelets 2–3(–3.4) mm long, less clustered at the branch tips; anthers 0.5–1.4 mm long	***A.scabra* Willd.**
24(21)	Panicles (4–)8–30 cm long, (2.2–)4–20(–26) cm wide, branches rebranching in the upper third; spikelets 2–3(–3.4) mm long, usually somewhat clustered at the branch tips; leaf blades 2–14 cm long	***A.scabra* Willd.**
–	Panicles 3–13 cm long, 1–6(–8) cm wide, branches rebranching about or slightly above mid-length; spikelets 1.5–2.5 mm long, not clustered; leaf blades 1–9 cm long	**25**
25(24)	Leaf blades 0.5–2 mm wide, linear, flat, becoming involute when drying; plants known in the study zone from southern Arizona and California	***A.idahoensis* Nash**
–	Leaf blades 0.2–0.5 mm wide, filiform, conduplicate to involute, rarely flat in the upper leaves; plants known in the study zone from central Mexico to Guatemala	***A.turrialbae* Mez**

#### ﻿1. Agrostis bourgaei

Taxon classificationPlantaePoalesPoaceae

E. Fourn., Mexic. Pl. 2: 95. 1886.

E4E4858D-6CF6-57DD-999B-4F76FE976364

Figs 3 , 4A , 5A

Agrostis bourgaei E. Fourn. ex Hemsl., Biol. Cent.-Amer., Bot. 3: 550. 1885, nom. nud.
= Agrostis thyrsigera Mez, Repert. Spec. Nov. Regni Veg. 17(19): 301. 1921. Type: Mexico. State of México: wet banks, Sierra de las Cruces, 12 Aug 1893, C.G. Pringle 4485 (lectotype, designated by Vigosa-Mercado (2022a: 1): BR (BR0000006863616 [image!]); isolectoypes: BR (BR0000006864217 [image!]), K (K000308369 [image!]), KFTA (KFTA0002213 [image!]), MSC (MSC0129859 [image!]), NDG (NDG07467 [image!]), NY (NY00688876 [image!], NY00688877 [image!]), S (S12-16472 [image!]), W (W18940003052 [image!])).

##### Type.

Mexico. Mexico City: pedrégal près Tizapan, vallée de Mexico, 2 Aug 1865, E. Bourgeau 682 (holotype: P (P00740531 [image!]); isotype: US [fragm. ex P] (US00156379)).

##### Description.

***Plants*** perennial, caespitose. ***Tillers*** extravaginal, with cataphylls. ***Culms*** 0.1–1.2 m long, erect, sometimes shortly decumbent at the base, nodes 2–4, glabrous, internodes glabrous, or sometimes scaberulous below the nodes and panicle. ***Leaves*** basal and cauline; sheaths 3–18 cm long, usually shorter than the internodes, glabrous or scaberulous; ligules 1–7 mm long, longer than wide, dorsally scaberulous, apices acute, often lacerate; blades 3–15(–30) cm long, (0.5–)1–6 mm wide, linear, flat, scaberulous on both surfaces. ***Panicles*** (2–)9–25(–30) cm long, (0.8–)3–7(–10) cm wide, open, lax, lanceolate, usually long-exserted from the upper sheaths; branches ascending to spreading, rebranching from about or above mid-length, scaberulous, without spikelets near their base, inferior branches (1–)2–10 cm long; pedicels 0.5–3 mm long, ascending to spreading, scaberulous. ***Spikelets*** 2–2.7 mm long, usually purplish; glumes subequal to equal, lanceolate, apices shortly acuminate, 1-veined, scaberulous on the keel, lower glume 2–2.7 mm long, upper glume 1.8–2.5 mm long; callus puberulous, with 2 bunches of short trichomes, often inconspicuous; lemmas 1.5–1.8 mm long, elliptic, apices entire, acute or toothed, 5-nerved, veins inconspicuous, unawned; paleas present, (0.4–)0.5–0.7(–1) mm long, veinless, glabrous; anthers 3, (0.3–)0.5–0.7 mm long. ***Caryopsis*** 1.2–1.5 mm long, ellipsoid; endosperm soft. 2n= unknown.

**Figure 5.**
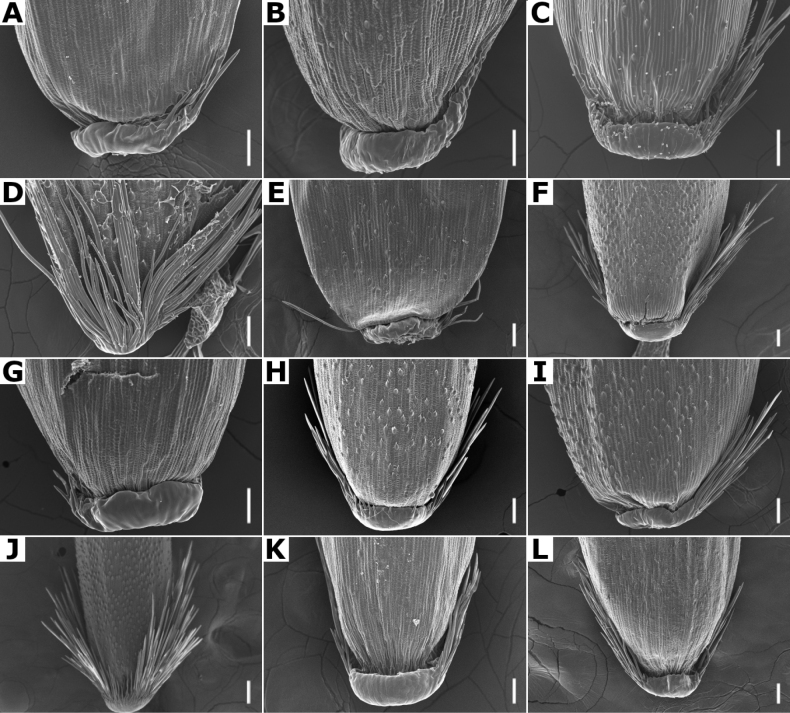
Calluses of *Agrostis* species observed with SEM **A***A.bourgaei***B***A.calderoniae***C***A.capillaris***D***A.elliottiana***E***A.exarata***F***A.ghiesbreghtii***G***A.gigantea***H***A.hyemalis***I***A.laxissima***J***A.microphylla***K***A.pallens***L***A.perennans*. Scale bars: 50 μm.

##### Anatomy and micromorphology.

**Figure 6.**
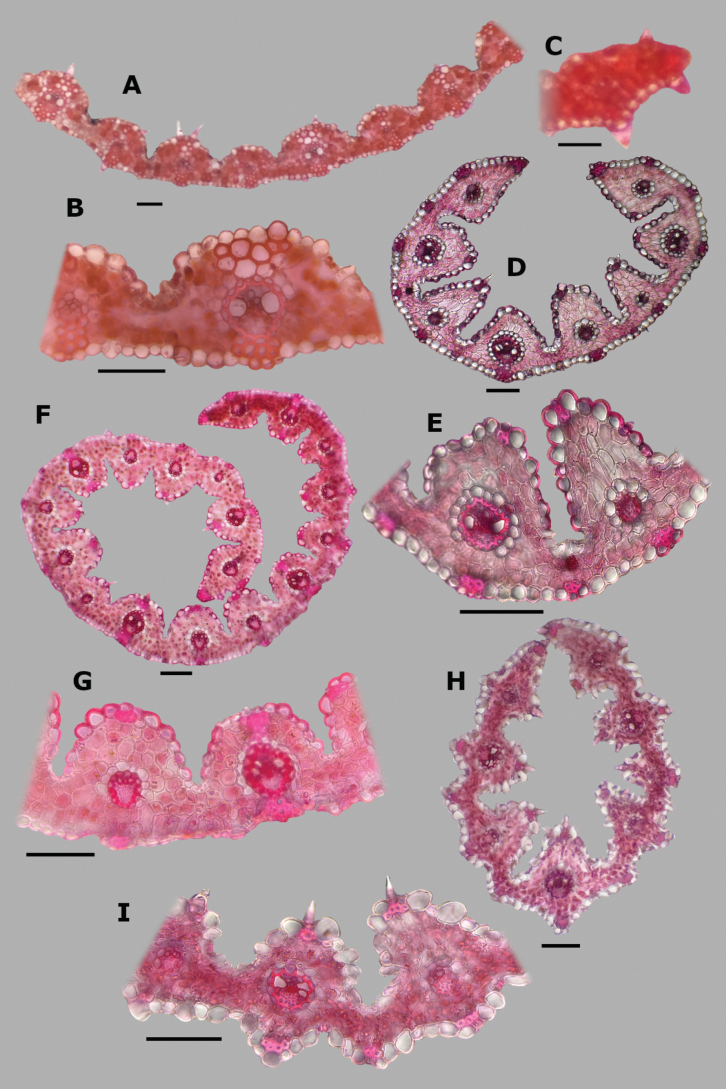
Leaf blade anatomy in transversal section of *Agrostis* species, in general view, and details of lateral bundles. **A**–**C***A.bourgaei***D**–**E***A.calderoniae***F**–**G***A.capillaris***H**–**I***A.elliottiana*. Scale bars: 0.1 mm.

**Figure 7.**
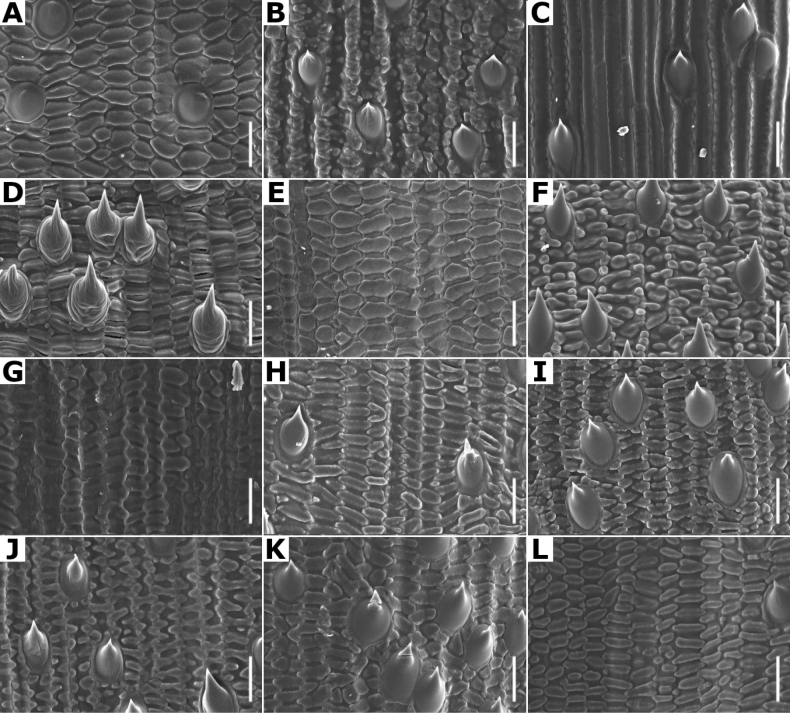
Lemma surface of *Agrostis* observed with SEM **A***A.bourgaei***B***A.calderoniae***C***A.capillaris***D***A.elliottiana***E***A.exarata***F***A.ghiesbreghtii***G***A.gigantea***H***A.hyemalis***I***A.laxissima***J***A.microphylla***K***A.pallens***L***A.perennans*. Scale bars: 15 μm.

Leaf blades flat in transversal section; adaxial furrows medium-sized, wide; adaxial ribs rounded; keel absent; first order bundles circular in outline, sheath interrupted adaxially and abaxially, abaxial and adaxial sclerenchyma in girders, narrowing towards the bundle; second order bundles circular in outline, sheath interrupted abaxially, abaxial sclerenchyma in girders, narrowing towards the bundle, adaxial sclerenchyma in strands or t-shaped girders; intercostal sclerenchyma absent; leaf margins with well-developed sclerenchyma caps, rounded; colorless cells absent (Fig. 6A–C). Lemmas with transversal thickenings polygonal, wider than the unthickened portions of the wall; prickle hairs scarce (Fig. 7A).

##### Distribution and habitat.

**Figure 8.**
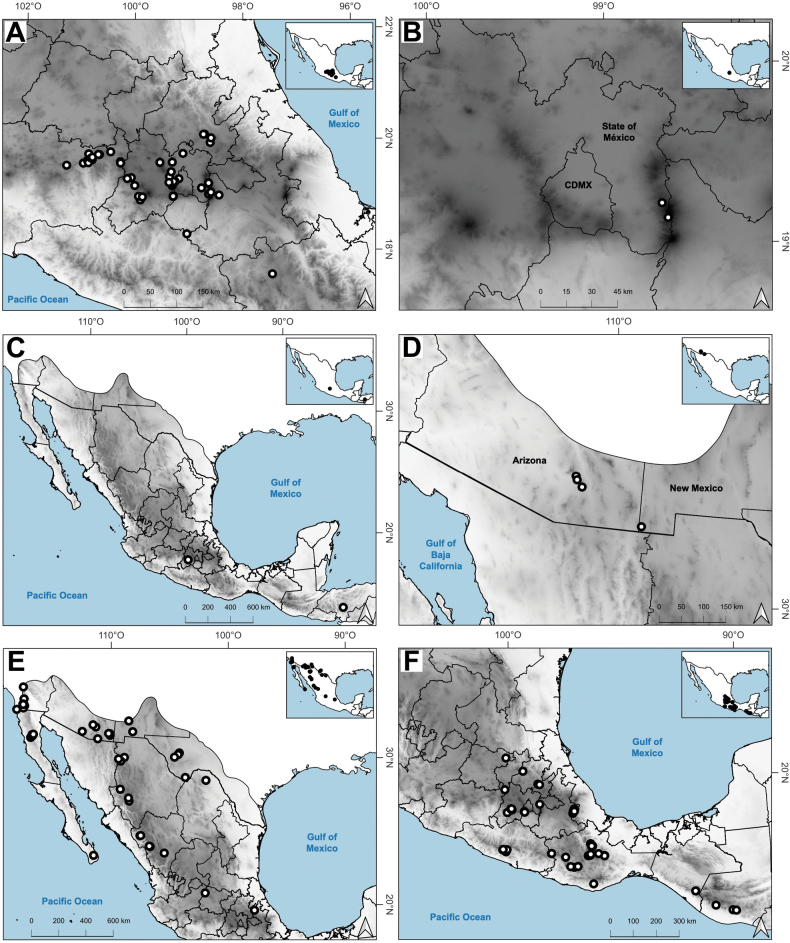
Map of known geographic distribution of *Agrostis* species, based on herbarium specimen data **A***A.bourgaei***B***A.calderoniae***C***A.capillaris***D***A.elliottiana***E***A.exarata***F***A.ghiesbreghtii*.

**Figure 9.**
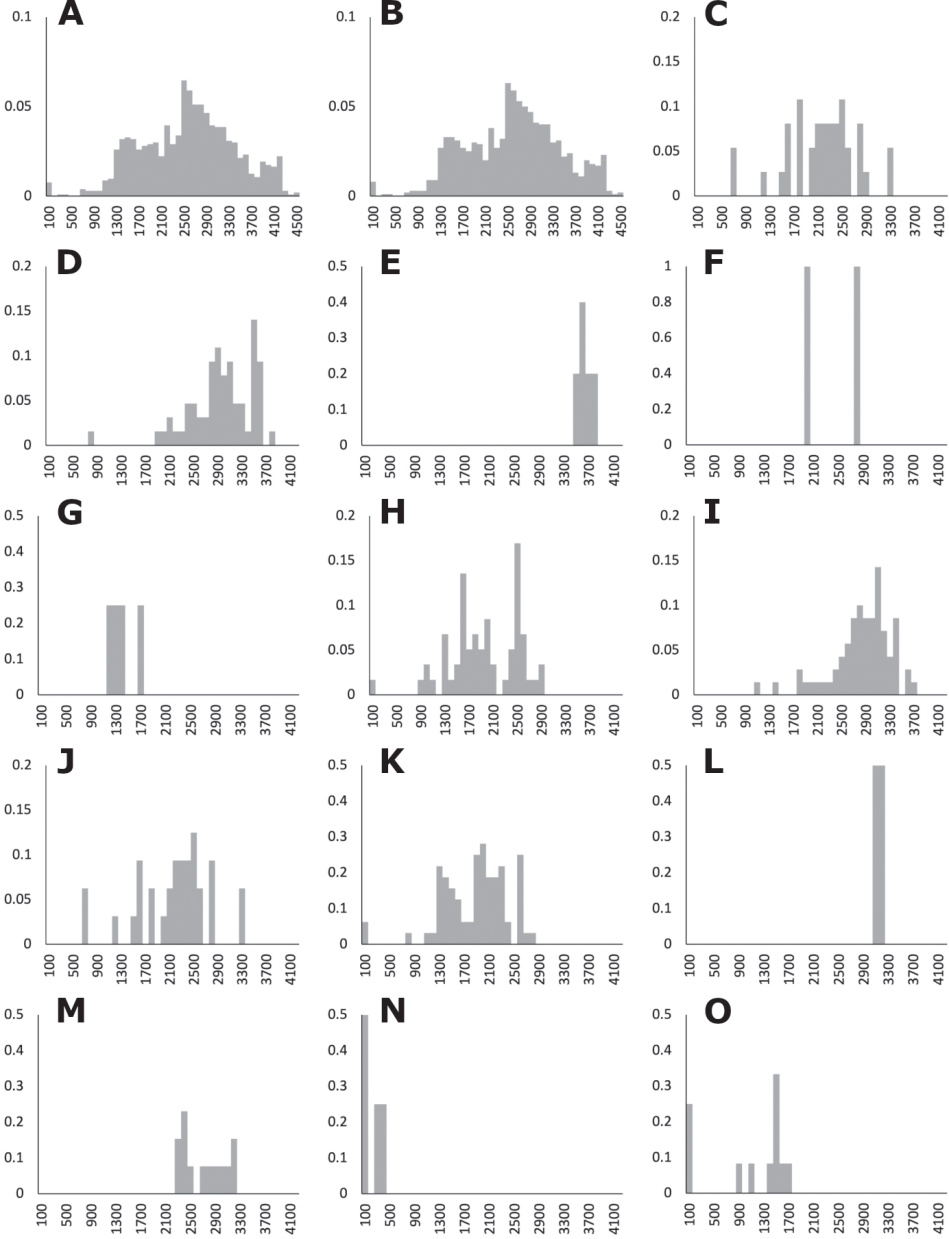
Elevation histograms of *Agrostis* species **A** all records **B** native species **C** introduced species **D***A.bourgaei***E***A.calderoniae***F***A.capillaris***G***A.elliottiana***H***A.exarata***I***A.ghiesbreghtii***J***A.gigantea***K***A.hyemalis***L***A.idahoensis***M***A.laxissima***N***A.microphylla***O***A.pallens*.

Endemic. Herbarium specimens of *A.bourgaei* have been collected in Mexico City and the Mexican states of Hidalgo, México, Michoacán, Morelos, Puebla, and Oaxaca (Fig. 8A). The species has also been reported for Durango, Guanajuato, Querétaro, Tlaxcala, and Veracruz (Villaseñor 2016; Dávila et al. 2018; Sánchez-Ken 2019), but no specimens from these states were seen. *Agrostisbourgaei* is mainly found on edges of streams and moist soils, in open areas of conifer forests of the Trans-Mexican Volcanic Belt, between 1800–3800 m a.s.l., with some outliers in Morelos found lower, at 725 m a.s.l. (Fig. 9D).

##### Phenology.

**Figure 10.**
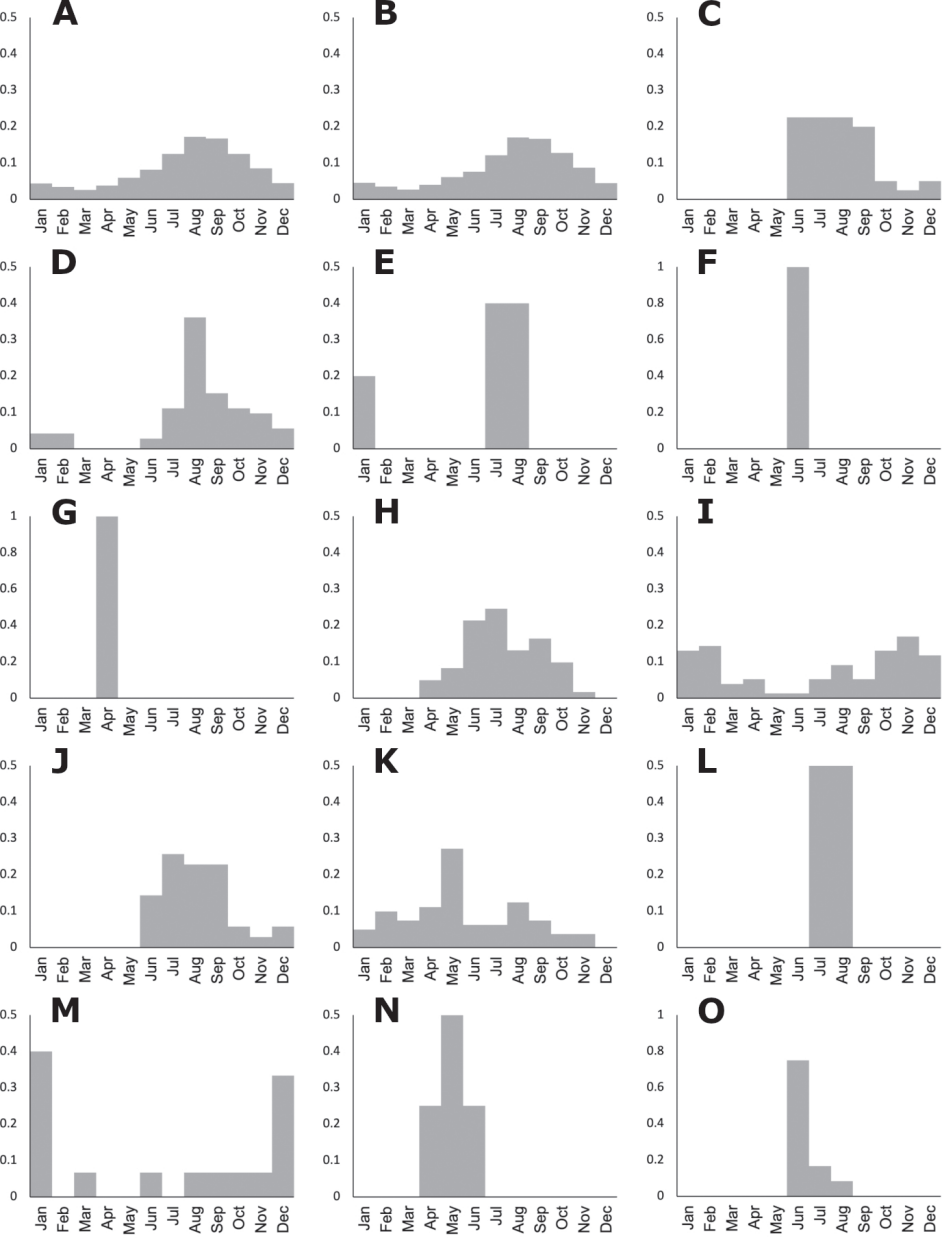
Phenology histograms of *Agrostis* species **A** all records **B** native species **C** introduced species **D***A.bourgaei***E***A.calderoniae***F***A.capillaris***G***A.elliottiana***H***A.exarata***I***A.ghiesbreghtii***J***A.gigantea***K***A.hyemalis***L***A.idahoensis***M***A.laxissima***N***A.microphylla***O***A.pallens*.

Specimens with spikelets have been collected from June to February, but most of the records are from August (Fig. 10D).

##### Commentaries.

*Agrostisbourgaei* is similar to *A.gigantea*, and both are often confused. They share very similar leaf anatomy and paleate spikelets, but *A.bourgaei* is distinguished from the latter in the much more fragile aspect of the plants, caespitose habit, lateral branches of the panicle without spikelets near their base, lemmas with prickle hairs, transversal thickenings, and shorter paleas of up to 0.7(–1) mm long (vs. robust plants, usually rhizomatous habit, often some inferior branches with spikelets near their base, lemmas glabrous, without transversal thickenings, paleas up to 1.2 mm long in *A.gigantea*). The specimen García-Mendoza 1116 (MEXU) from Oaxaca lacks vegetative parts that may allow a more acceptable identification, but is included tentatively in this species for its paleate spikelets.

##### Conservation status.

*Agrostisbourgaei* is an abundant and widespread species in Central Mexico. It is represented by 61 collections, with several populations occurring in 13 protected areas. The EOO is 45,765 km^2^ and the AOO is 1,476 km^2^. Following the IUCN criteria, the preliminary assessment category is Least Concern (LC).

##### Representative specimens examined.

**Mexico. Hidalgo**: **Municipio Epazoyucan**, Peñas Largas, [20.1°N, 98.6°W], 2850 m alt., 22 Dec 1983 J. Rzedowski 38373a (IEB, MEXU, XAL). **México: Municipio Lerma**, 5 km al W de La Marquesa, en la desviación a Salazar, [19.304403°N, 99.39047°W], 2800 m alt., 1 Aug 1981, R. Guzmán 3966 (IBUG, MEXU). **Municipio Ocuilan**, Laguna de Zempoala, [19.05034984°N, 99.31696647°W], 2800 m alt., 1 Aug 1987, J. Castañeda 327 (MEXU [*]). **Mexico City: Alcaldía Cuajimalpa**, Llano de la Cieneguilla, Puerto de las Cruces, [19.24694444°N, 99.33444444°W], 3500 m alt., 19 Sep 1983, S. Acosta and R. Aguilar 417 (CIIDIR, IEB, MEXU), 418 (CIIDIR, IEB, MEXU [*,**]), 419 (CIIDIR, IBUG, MEXU), 420 (CIIDIR, IBUG, IEB, MEXU); Parque Nacional Desierto de los Leones, 2 km a pie de La Venta por la brecha que va a Santa Rosa, 19.321112°N, 99.306817°W, 2881 m alt., 22 Aug 2021, L. Vigosa and A. Mercado 112 (MEXU [*,**]). **Michoacán: Municipio Angangueo**, Estación Chincua, Reserva de la Biósfera Mariposa Monarca, [19.65618889°N, 100.2717278°W], 3030 m alt., 5 Aug 2000, M.G. Cornejo et al. 67 (IEB, MEXU [*]). **Municipio Maravatío**, km 6 carretera Maravatío-Ciudad Hidalgo al S del poblado de Casa Blanca, [19.85705°N, 100.4516389°W], 2000 m alt., J.E. Morales and A. Pastor 56 (IEB, MEXU [**]). **Morelos: Municipio Huitzilac**, Lagunas de Zempoala, [19.05320158°N, 99.31276612°W, 2800 m alt.], 19 Sep 1938, E. Lyonnet 2518 (FCME [*], MEXU, UAMIZ, US); **Municipio Tlaquiltenango**, Huaxtla 18.37444444°N, 99.07222222°W, 725 m alt., 23 Feb 2015, G. Rendón et al. s.n. (HUMO). **Oaxaca: Municipio San Andrés Lagunas**, Laguna Grande, 1 km al N de San Isidro Lagunas, [17.62219847°N, 97.5414394°W], 2200 m alt., 5 Aug 1982, A. García-Mendoza 1116 (MEXU). **Puebla: Municipio San Nicolás de los Ranchos**, Paso de Cortés, 3 km al S de la carretera a Amecameca, sobre la brecha al Volcán Iztaccíhuatl, 13 km al E de Amecameca, [19.12°N, 98.63°W], 3760 m alt., 1 Nov 1976, S.D. Koch 76237 (CHAPA, US). See Suppl. materials 2, 3 for additional examined specimens.

#### ﻿2. Agrostis calderoniae

Taxon classificationPlantaePoalesPoaceae

Acosta, Phytologia 62(6): 449, Fig. 1. 1987.

2A70AC98-CBF6-5E94-B1DC-4FF42BD7E9DE

Figs 4B , 5B , 11

##### Type.

Mexico. State of México: Municipio Tlalmanalco, La Ciénega, región de Peñas Cuatas, ladera NW del Iztaccíhuatl, 3600 m alt., 19 Aug 1984, S. Acosta 687 (holotype: ENCB! (ENCB003243 [image!]); isotypes: MEXU! (MEXU00436130 [image!]), CHAPA! (CHAPA0000037 [image!]), IEB, TEX).

##### Description.

***Plants*** perennial, caespitose. ***Tillers*** extravaginal, with cataphylls. ***Culms*** 10–40 cm long, erect, nodes 1–2, glabrous, internodes glabrous. ***Leaves*** mostly basal; sheaths 3–10 cm long, usually longer than the internodes, glabrous; ligules 1.5–4 mm long, longer than wide, glabrous, apices acute, often lacerate; blades (1–)2–10 cm long, up to 2 mm wide, linear, conduplicate to convolute, scaberulous on both surfaces. ***Panicles*** 5–13 cm long, 2–10 cm wide, open, lax, lanceolate to ovate, long-exserted from the upper sheaths; branches ascending to spreading, rebranching about or above mid-length, scaberulous, without spikelets near their base, inferior branches 2–7 cm long; pedicels 1–3 mm long, ascending to spreading, scaberulous. ***Spikelets*** 2.5–3.5 mm long, purplish; glumes subequal to equal, lanceolate, apices shortly acuminate, 1-veined, scaberulous on the keel, lower glume 2.5–3.5 mm long, upper glume 2.2–3.5 mm long; callus glabrous; lemmas 2–2.5 mm long, elliptic, apices irregularly toothed, 5-nerved, veins inconspicuous, unawned; paleas present, 0.5–1 mm long, veinless, glabrous; anthers 3, 0.7–1 mm long. ***Caryopsis*** not seen. 2n= unknown.

**Figure 11.**
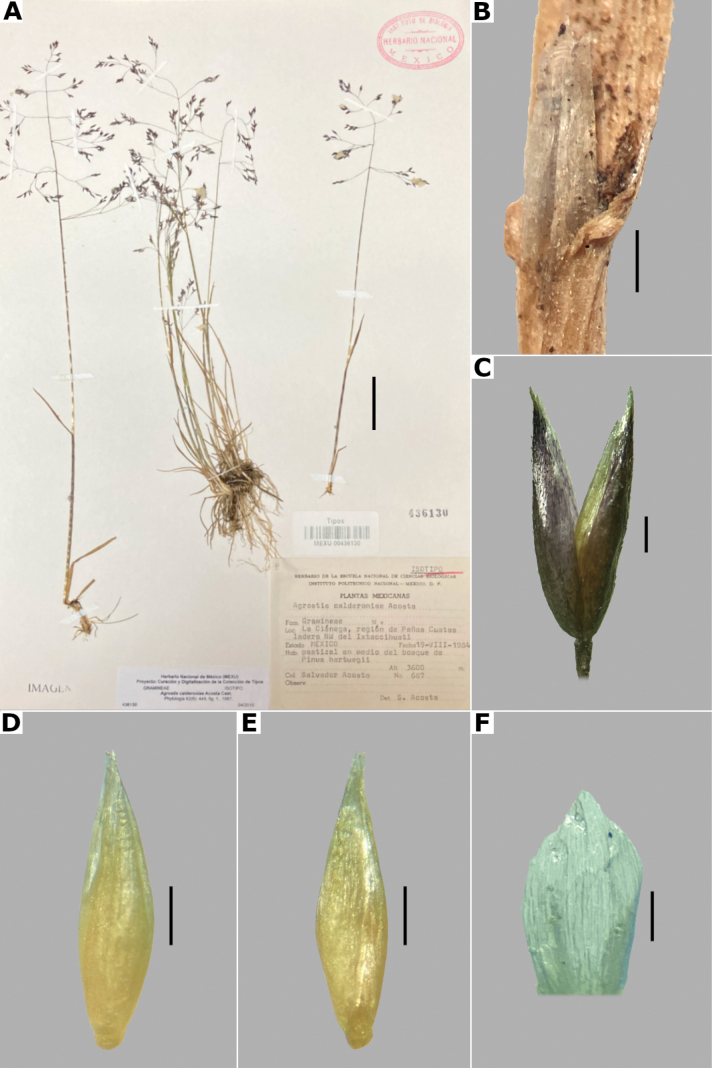
*Agrostiscalderoniae***A** whole plant **B** ligular area **C** spikelet **D** floret, abaxial view **E** floret, adaxial view **F** palea. A based on Acosta 687 (isotype, MEXU), B–F based on Acosta 692 (IEB). Scale bars: 3 cm (**A**); 1 mm (**B**); 0.5 mm (**C–E**); 0.2 mm (**F**).

##### Anatomy and micromorphology.

Leaf blades convolute or v-shaped in transversal section; adaxial furrows deep, narrow; adaxial ribs rounded to triangular; keel absent; first order bundles circular in outline, sheath interrupted abaxially, abaxial sclerenchyma in strands or girders in the central bundle, narrowing towards the bundle, adaxial sclerenchyma in strands; second order bundles circular in outline, sheath not interrupted, abaxial and adaxial sclerenchyma; intercostal sclerenchyma present, abaxial; leaf margins with well-developed sclerenchyma caps, rounded; colorless cells absent (Fig. 6D, E). Lemmas with transversal thickenings irregular, wider than the unthickened portions of the wall; prickle hairs abundant (Fig. 7B).

##### Distribution and habitat.

Endemic. *Agrostiscalderoniae* has only been collected in the western slope of the Iztaccíhuatl volcano, in the state of México (Fig. 8B). This species grows in moist soils near the edge of streams, in open areas of temperate forests with *Pinus*, between 3500–3800 m a.s.l. (Fig. 9E).

##### Phenology.

Specimens with spikelets have been collected in January, July, and August (Fig. 10E).

##### Commentaries.

This species is similar to *A.bourgaei*, in the caespitose habit and paleate spikelets, but it is distinguished in the mostly basal leaves, leaf blades conduplicate or convolute, with deep adaxial furrows, abaxial intercostal sclerenchyma, longer spikelets of 2.5–3.5 mm long, and lemmas with transversal thickenings of irregular shape (vs. basal and cauline leaves, leaf blades flat, with medium-sized adaxial furrows, without intercostal sclerenchyma, spikelets of 2–2.7 mm long, lemmas with polygonal transversal thickenings in *A.bourgaei*).

##### Conservation status.

*Agrostiscalderoniae* only has been collected in two localities of the Iztaccíhuatl-Popocatéptl National Park, where it is reported as scarce in the labels of the herbarium specimens. The EOO and AOO cannot be calculated, but by the scarcity and size of the populations of this species, the category of Endangered (EN) is suggested.

##### Specimens examined.

**Mexico. México: Municipio Amecameca**, vertiente SW del Iztaccíhuatl, 4 km al N de la estación retransmisora de TV, [19.14308652°N, 98.64774789°W], 3800 alt., 15 Jul 1965, J. Rzedowski 20161 (ENCB). **Municipio Tlalmanalco**, vertiente NW del Iztaccíhuatl, en la región de Peñas Cuatas, La Ciénega, [19.225278°N, 98.680833°W], 3650 m alt., 6 Jan 1966, J. Rzedowski 21798 (ENCB); La Ciénega, región de la cabeza del Iztacccíhuatl, [19.225278°N, 98.680833°W], 19 Aug 1984, S. Acosta 692 (IEB [*,**]); 3600 m alt., 18 Jul 1982, J. Rzedowski 37855 (ENCB).

#### ﻿3. Agrostis capillaris

Taxon classificationPlantaePoalesPoaceae

L., Sp. Pl. 1: 62. 1753.

0E5286B6-E7B7-5EF8-9D55-489B6614600B

Figs 4C , 5C , 12

= Agrostis capillaris Huds., Fl. Angl. 27. 1762, nom. illeg. hom., non L., 1753. Agrostistenuis Sibth., Fl. Oxon. 36. 1794. AgrostisalbaL.var.tenuis (Sibth.) Fiori, Nuov. Fl. Italia 1: 97. 1923. Type: England. Habitat in pratis et pascuis ubique (not located).
Agrostis polymorpha Huds. var. capillaris (L.) Huds., Fl. Angl. 1: 31. 1778.
Vilfa capillaris (L.) P. Beauv., Ess. Agrostogr. 147. 1812.
Trichodium capillaris (L.) Roth, Nov. Pl. Sp. 41. 1821.^*^

##### Type.

Herb. A. Van Royen s.n. (lectotype, designated by Widén (1971: 65): L [left hand specimen] (L (L0052645 [image!])).

##### Description.

***Plants*** perennial, short rhizomatous, rarely stoloniferous. ***Tillers*** extravaginal, with cataphylls. ***Rhizomes*** up to 5 cm long. ***Culms*** 10–80 cm long, erect, decumbent at the base, nodes up to 7, glabrous, internodes glabrous. ***Leaves*** basal and cauline; sheaths 1–13 cm long, longer or shorter than the internodes, glabrous; ligules of the basal blades 0.2–1.5 mm long, the upper ones up to 2 mm long, usually shorter than wide, rarely longer than wide, dorsally scaberulous, apices rounded to truncate, erose, sometimes lacerate; blades 3–15 cm long, 1–5 mm wide, linear, flat, sometimes becoming convolute when drying, scaberulous on both surfaces. ***Panicles*** (3–)10–20 cm long, (1–)2–12 cm wide, open, lax, oblong to ovate, usually long-exserted from the upper sheaths; branches ascending to spreading, rebranching about or above mid-length, scaberulous, without spikelets near their base, inferior branches up to 7 cm long; pedicels 0.4–3.3 mm long, usually spreading, scaberulous. ***Spikelets*** (1.7–)2–3(–3.5) mm long, greenish to purplish; glumes subequal, lanceolate, apices shortly acuminate, 1-veined, scaberulous on the keel, lower glume 2.5–3.5 mm long, upper glume 2.2–3.3 mm long; callus glabrous or with a few trichomes, inconspicuous; lemmas 1–2.5 mm long, elliptic, apices entire, acute to obtuse, sometimes truncate, 3(5)-veined, veins usually prominent, sometimes two veins excurrent ca. 0.5 mm, unawned, rarely awned near mid-length, awn up to 2 mm, geniculate or straight; paleas present, 0.6–1.2(–1.4) mm long, 2-veined, glabrous; anthers 3, 0.8–1.4 mm long. ***Caryopsis*** 0.8–1.5, elliptic; endosperm solid. 2n= 28 (Harvey 2007).

**Figure 12.**
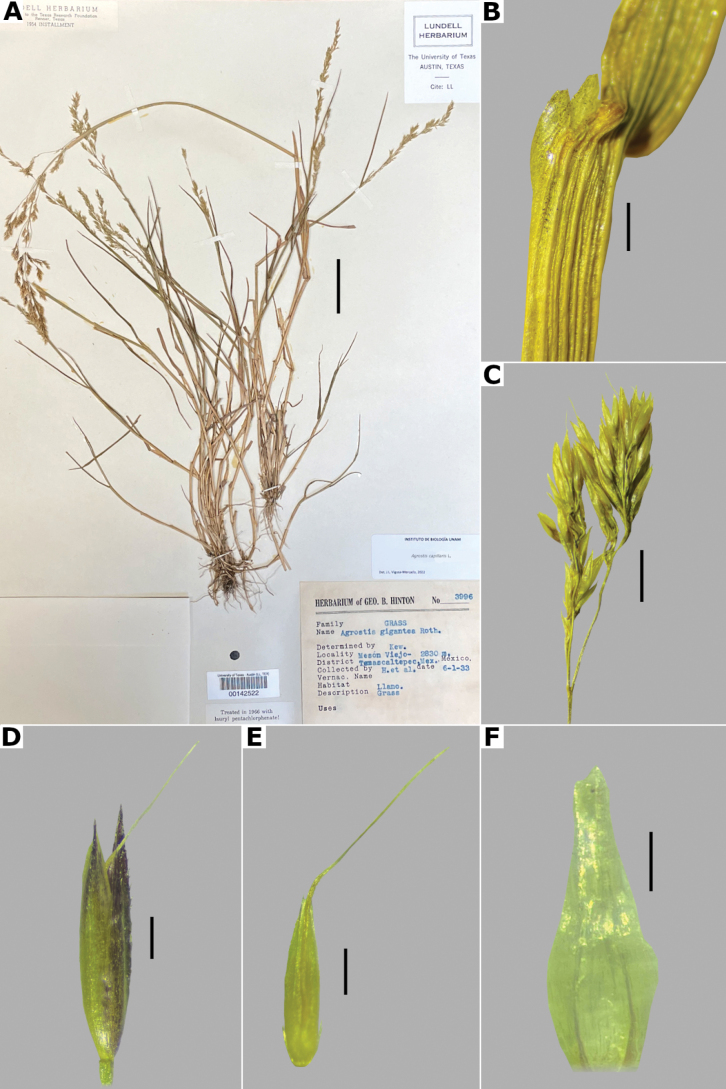
*Agrostiscapillaris***A** whole plant **B** ligular area **C** branch of the panicle **D** spikelet **E** floret, abaxial view **F** palea. Based on Hinton 3996 (TEX). Scale bars: 3 cm (**A**); 1 mm (**B**); 4 mm (**C**); 0.5 mm (**D, E**); 0.2 mm (**F**).

##### Anatomy and micromorphology.

Leaf blades flat to convolute in transversal section; adaxial furrows medium-sized, narrow; adaxial ribs rounded to triangular; keel absent; first order bundles circular to slightly elliptical in outline, sheath interrupted abaxially or also adaxially, abaxial and adaxial sclerenchyma in girders, narrowing towards the bundle; second order bundles circular in outline, sheath interrupted abaxially, abaxial and adaxial sclerenchyma; intercostal sclerenchyma absent; leaf margins with well-developed sclerenchyma caps, rounded; colorless cells absent (Fig. 6F, G). Lemmas without transversal thickenings; prickle hairs abundant (Fig. 7C).

##### Distribution and habitat.

Introduced. *Agrostiscapillaris* is native to Europe. This species has been collected in Francisco Morazán, Honduras, and in the state of México, Mexico (Fig. 8C). It grows in cloud forests and *Pinus* forests, at 2000–2830 m a.s.l. (Fig. 9F).

##### Phenology.

Specimens with spikelets have been collected in June (Fig. 10F).

##### Commentaries.

This species is often confused with *A.castellana* (see notes under excluded species). Some individuals of *A.capillaris* can develop ligules of the flowering culms longer than wide.

##### Conservation status.

Since *Agrostiscapillaris* is an introduced species in the study zone, its conservation status is considered as Least Concern (LC).

##### Specimens examined.

**Honduras. Francisco Morazán: Distrito Central**, bosque denso, húmedo y nebuloso de Montaña La Tigra, al SO de San Juancito, [14.2°N, 87.1°W], 2000 m alt., 11 Jun 1963, A. Molina 12706 (MICH, US). **MEXICO. México: Municipio Temascaltepec**, Mesón Viejo, [19.16666667°N, 99.88333333°W], 2830 m alt., 1 Jun 1933, G.B. Hinton 3996 (MO, TEX [*, **], US).

#### ﻿4. Agrostis elliottiana

Taxon classificationPlantaePoalesPoaceae

Schult., Mant. 2: 202. 1824.

3EE073AE-05EB-5C28-A386-FC843953256C

Figs 4D , 5D , 13

Agrostis arachnoides Elliott, Sketch Bot. S. Carolina 1(2): 134. 1816, nom. illeg. hom., non Poir., 1810. Type: USA. South Carolina near Orangeburgh, I.S. Bennet s.n. (holotype: CHARL (CHARL-BY3758 [image!])).
Notonema arachnoides Raf., Neogenyton 4. 1825.
= Agrostis exigua Thurb., Bot. California 2: 275. 1880. Type: USA. California: foothills of the Sierras, H.N. Bolander s.*n.* (holotype: GH (GH00022956 [image!]); isotypes: MO (MO-123096 [image!]), US (US00156423[image!])).
Notonema arachnoides Raf. ex B.D. Jacks., Index Kew. 2: 319. 1894, nom. inval., pro syn.
= Agrostis elliottiana Schult. fo. molesta Shinners, Rhodora 56(662): 28. 1954. Type: USA. Texas: Wood County, sandy upland pine woods 2.7 miles east of Mineaola, 23 Apr 1953, L.H. Shinners 14372 (holotype: BRIT (BRIT408703 [image!])).

##### Type.

Based on *Agrostisarachnoides* Elliott.

##### Description.

***Plants*** annual, caespitose. ***Tillers*** absent. ***Culms*** 5–45 cm long, erect, sometimes geniculate at the base, nodes (3–)4–9, glabrous, internodes glabrous. ***Leaves*** basal and cauline; sheaths 1–5 cm long, longer or shorter than the internodes, glabrous or scaberulous; ligules (0.7–)1.5–3.5(–5) mm long, longer than wide, dorsally scaberulous, apices rounded to truncate, lacerate; blades 0.5–4(–7) cm long, 0.5–1 mm wide, linear, flat, sometimes becoming conduplicated, scaberulous on both surfaces. ***Panicles*** (3–)5–20 cm long, (0.5–)2–12 cm wide, open, lax, linear to ovate, usually exserted from the upper sheaths, the whole panicle detaching after maturity; branches ascending to spreading, rebranching about or above mid-length, scaberulous, without spikelets near their base, inferior branches 2–8 cm long; pedicels 0.3–7.5 mm long, ascending to spreading, scaberulous. ***Spikelets*** 1.5–2.2 mm long, purplish; glumes equal to subequal, lanceolate, apices acute, 1-veined, scaberulous on the keel, sometimes margins distally scaberulous, lower glume 1.5–2.2 mm long, upper glume 1.3–2 mm long; callus pubescent, with 2 bunches of trichomes; lemmas 1–2 mm long, elliptic, apices entire, acute or toothed, 5-nerved, veins prominent, awned in the upper third, awn 3–10 mm, flexuous, deciduous; paleas absent, or up to 0.2 mm long, veinless, glabrous; anther 1, 0.1–0.2 mm long. ***Caryopsis*** 1–1.4 mm long, elliptic; endosperm liquid. 2n= 28 (Harvey 2007).

**Figure 13.**
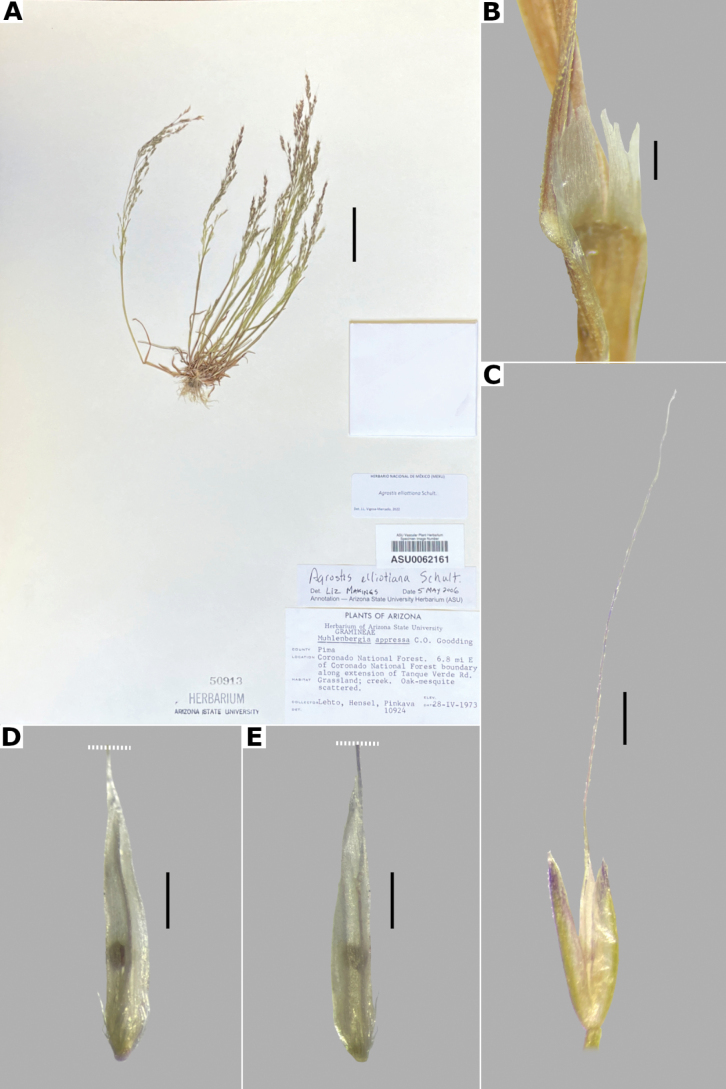
*Agrostiselliottiana***A** whole plant **B** ligular area **C** spikelet **D** floret, abaxial view **F** floret, adaxial view. Based on Lehto et al. 10924 (ASU). Scale bars: 3 cm (**A**); 5 mm (**B**); 0.5 mm (**C**); 0.3 mm (**D, E**).

##### Anatomy and micromorphology.

Leaf blades flat to v-shaped in transversal section; adaxial furrows deep, narrow; adaxial ribs triangular; keel absent; first order bundles circular in outline, sheath interrupted abaxially, abaxial and adaxial sclerenchyma in strands; second order bundles circular in outline, sheath interrupted abaxially, abaxial and adaxial sclerenchyma in strands; intercostal sclerenchyma present, abaxial; leaf margins with well-developed sclerenchyma caps, rounded; colorless cells absent (Fig. 6H, I). Lemmas with transversal thickenings oblong, wider than the unthickened portions of the wall; prickle hairs abundant (Fig. 7D).

##### Distribution and habitat.

*Agrostiselliottiana* is native to North America, distributed on the East Coast of USA, from Pennsylvania to Florida, and from northern California to Texas (Harvey 2007). Herbarium specimens from the study zone of this species have been collected in southern Arizona and New Mexico, USA (Fig. 8D). *Agrostiselliottiana* has also been reported from the Mexican state of Yucatán (Beetle et al. 1983), but herbarium specimens associated with this record have not been found. This species grows in the edges of streams, in the middle of scrublands, with *Arctostaphylos* and *Juniperus*, between 1189–1676 m a.s.l. (Fig. 9G). There are more records of this species in the study zone, on databases (GBIF 2023a), but not all of them have images, and thus we were unable to confirm their identity.

##### Phenology.

Specimens with spikelets have been collected in April (Fig. 10G).

##### Commentaries.

*Agrostiselliottiana* is often confused with *A.hyemalis* but differs from it in the annual habit of the plants, leaf blades sometimes conduplicated, with triangular adaxial ribs and abaxial intercostal sclerenchyma, spikelets not clustered, lemmas with a flexuous awn, and only one anther (vs. perennial plants, leaf blades flat, with rounded abaxial ribs, without intercostal sclerenchyma, spikelets clustered at branch tips, lemmas unawned, three anthers in *A.hyemalis*).

##### Conservation status.

*Agrostiselliottiana* apparently is a rare species in the study zone. It is only represented by four collections, occurring in the Coronado National Forest. The EOO is 1,078 km^2^ and the AOO is 16 km^2^. Following the IUCN criteria, the preliminary assessment category is Endangered (EN).

##### Specimens examined.

**USA. Arizona: Pima County**, Arizona National Scenic Trail, Santa Catalina Mountains, Coronado National Forest, along 4WD road west of Bellota Ranch, 32.324417°N, 110.6547°W, 1189 m alt., 28 Apr 2005, W.C. Hodgson et al. 20220 (DES); Coronado National Forest, 6.8 mi E of Coronado National Forest boundary, along extension of Tanque Verde Road, [32.2516°N, 110.62°W], 1300 m alt., 28 Apr 1973, E. Lehto et al. 10924 (ASU [*, **]); E edge of Rincon Mountains, 4 km N of Pima-Cochise County line along USFS-35 (Mescal Road), [32.116667°N, 110.483333°W], 1330 m alt., 16 Apr 1986, J.R. Reeder 842 (MICH). **New Mexico: Hidalgo County**, Peloncillo Mountains, Cloverdale Creek Canyon about 1.3 road mi NW of the Pendleton Ranch House, [31.448973°N, 108.986951°W], 1676 m alt., 20 Apr 1986, R.D. Worthington 14015 (COLO, DES, NY, UNM).

#### ﻿5. Agrostis exarata

Taxon classificationPlantaePoalesPoaceae

Trin., Gram. Unifl. Sesquifl. 207. 1824.

9B2C37B1-2308-52BD-A2EE-3193095F7375

Figs 4E , 5E , 14

= Polypogon monspeliensis (L.) Desf. var. monolepis Torr., Pacif. Railr. Rep. 5(2): 366. 1858. AgrostisexarataTrin.var.monolepis (Torr.) Hitchc., Amer. J. Bot. 21(3): 136. 1934. AgrostisamplaHitchc.fo.monolepis (Torr.) Beetle, Bull. Torrey Bot. Club 72: 544. 1945. Type. USA. California: Pose Creek, Walkers Pass, Aug 1853, Blake s.n. (holotype: NY (NY431434 [image!]); isotype: US [fragm. ex NY] (US04019109 [image!]).
= Agrostis durangensis Mez, Repert. Spec. Nov. Regni Veg. 17(19–30): 301. 1921. Type: MEXICO. Durango: collected at the city of Durango and vicinity, 1896, E. Palmer 190 (lectotype, designated by Vigosa-Mercado (2022a: 1): MO (MO-128909 [image!]; isolectotypes F (F0046563F [image!]), FCME! [fragm. ex US], GH (GH00022993 [image!]), NY (NY00327644 [image!]), US (US00151844 [image!], US00151845 [image!], US00151846 [image!], US00156413 [image!], US00486604 [image!])).^*^

##### Type.

USA. Alaska: Unalaska, 1829, J.F. Eschscholtz s.n. (holotype: LE (LE00009316 [image!]); isotype: LE).

##### Description.

***Plants*** perennial, caespitose, rarely shortly rhizomatous. ***Tillers*** extravaginal, with cataphylls. ***Culms*** 10–90 cm long, erect or decumbent at the base, nodes 2–4, glabrous, internodes glabrous. ***Leaves*** basal and cauline; sheaths 3–10 cm long, usually shorter than the internodes, glabrous; ligules 2.5–5 mm long, longer than wide, dorsally scaberulous, apices acute, often lacerate; blades 3–15 cm long, 1.5–4(–8) mm wide, linear, flat, scaberulous on both surfaces. ***Panicles*** (2–)4–20(–30) cm long, 0.4–2(–3) cm wide, contracted, usually dense and spiciform, lanceolate to oblong, sometimes interrupted at the base, sometimes partially included in the upper sheaths; branches appressed to ascending, rebranching about or below mid-length, scaberulous, usually with spikelets almost to the base, inferior branches 1–5 cm long; pedicels 0.5–3 mm long, appressed, scaberulous. ***Spikelets*** 2–2.5 mm long, greenish to stramineous; glumes subequal to unequal, lanceolate, apices acute to shortly acuminate, 1-veined, scaberulous on the keel, sometimes also on the body, sometimes awned, awn ca. 1 mm long, lower glume 2–2.5 mm long, upper glume 1.5–2.3 mm long; callus glabrous or with 2 bunches of few, short trichomes, often inconspicuous; lemmas 1.2–2 mm long, elliptic, apices acute or shortly toothed, 5-nerved, veins prominent distally or inconspicuous throughout, usually unawned, sometimes awned about mid-length, awn up to 3 mm long, geniculate or straight; paleas absent or 0.3–0.8 mm long, veinless, glabrous; anthers 3, 0.3–0.7 mm long. ***Caryopsis*** 1–1.5 mm long, elliptic; endosperm soft or solid. 2n= 28, 42, 56 (Harvey 2007).

**Figure 14.**
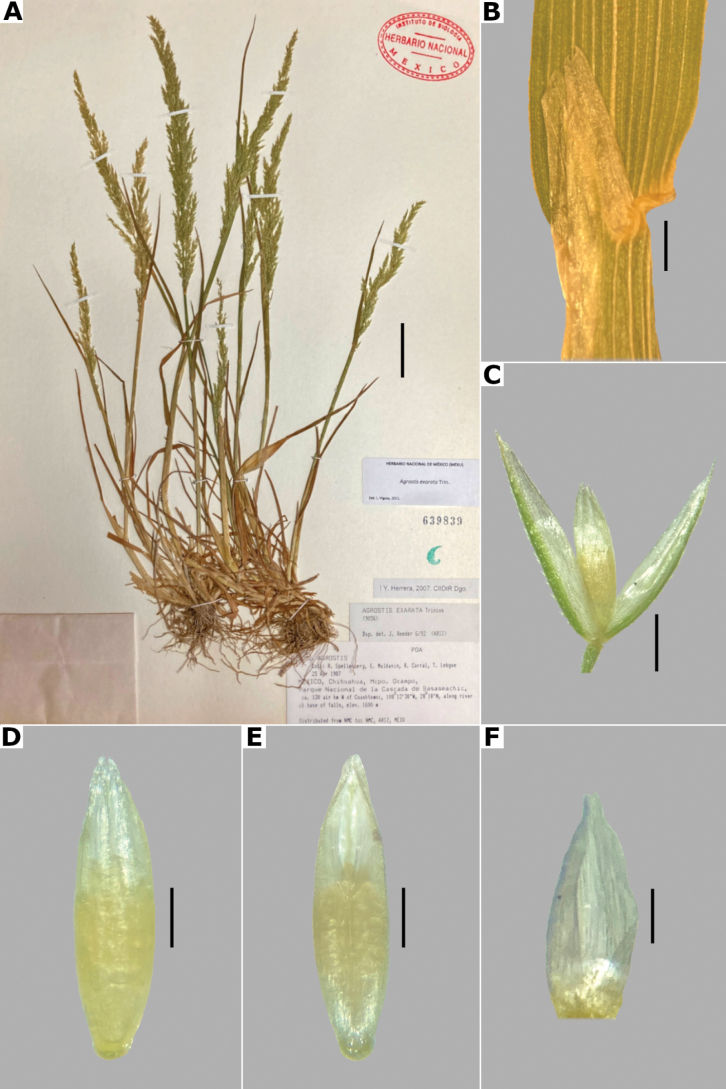
*Agrostisexarata***A** whole plant **B** ligular area **C** spikelet **D** floret, abaxial view, **E** floret, adaxial view **F** palea. Based on Spellenberg et al. 9056 (MEXU). Scale bars: 3 cm (**A**); 1 mm (**B**); 0.5 mm (**C**); 0.3 mm (**D, E**); 0.2 mm (**F**).

##### Anatomy and micromorphology.

**Figure 15.**
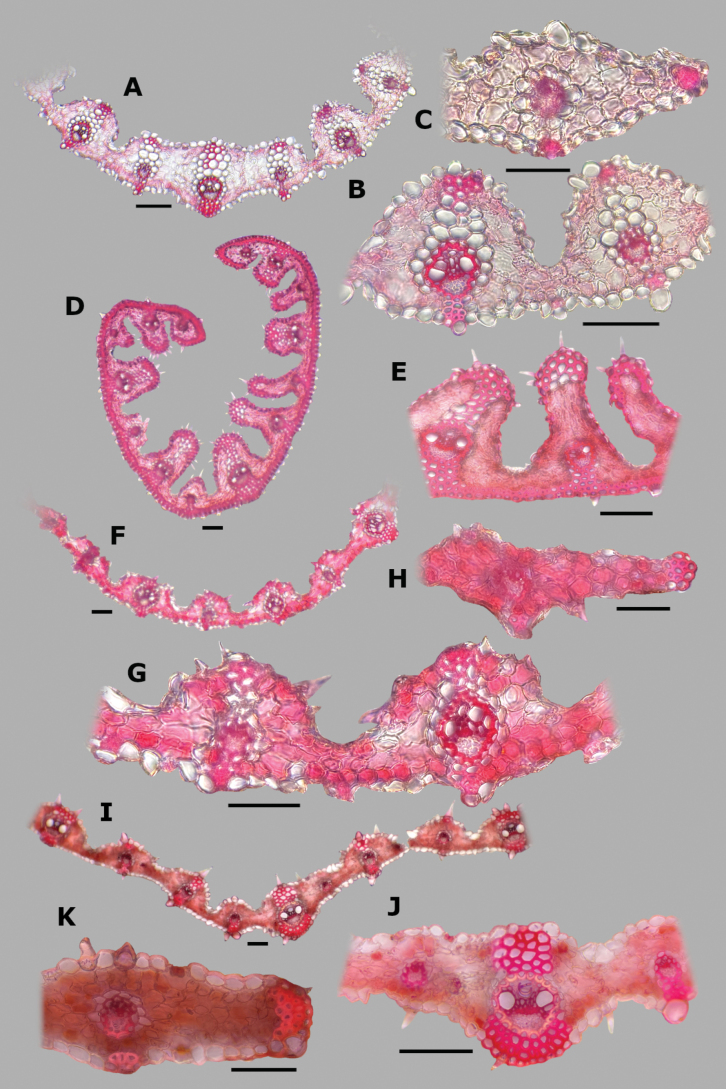
Leaf blade anatomy in transversal section of *Agrostis* species, in general view, and details of lateral bundles **A**–**C***A.exarata***D**–**E***A.ghiesbreghtii***F**–**H***A.gigantea***I**–**K***A.hyemalis*. Scale bars: 0.1 mm.

Leaf blades flat in transversal section; adaxial furrows deep, narrow; adaxial ribs rounded; keel sometimes present, with three vascular bundles; first order bundles circular in outline, sheath interrupted abaxially, abaxial and adaxial sclerenchyma in girders, narrowing towards the bundle; second order bundles circular in outline, sheath interrupted abaxially, abaxial sclerenchyma in girders, narrowing towards the bundle, adaxial sclerenchyma in strands; intercostal sclerenchyma absent; leaf margins with well-developed sclerenchyma caps, rounded; colorless cells present, associated with first and second order vascular bundles (Fig. 15A–C). Lemmas with transversal thickenings polygonal, wider than the unthickened portions of the wall; prickle hairs absent or scarce (Fig. 7E).

##### Distribution and habitat.

*Agrostisexarata* is distributed from Alaska to central Mexico, and is also found in Kamchatka and the Kuril Islands (Harvey 2007). In the study zone, this species has been collected in the USA states of Arizona, California, New Mexico and Texas, and in the Mexican states of Baja California, Baja California Sur, Chihuahua, Coahuila, Durango, Guanajuato, Hidalgo and Sonora (Fig. 8E). It has also been reported from Mexico City and the Mexican states of México and Puebla (Villaseñor 2016; Sánchez-Ken 2019), but no specimens from these states were seen. It grows in moist soils of stream edges and lake margins, in open areas of temperate forests with *Pinus* or *Quercus*, and shrublands, between 350–2900 m a.s.l. (Fig. 9H). The USA and Baja California populations of this species grow in lower elevations than the southern ones.

##### Phenology.

Specimens with spikelets have been collected from April to November (Fig. 10H).

##### Commentaries.

For other regions it has been reported that the panicles can reach 4 cm wide, and paleas up to 0.5 mm long (Harvey 2007).

*Agrostisexarata* is a variable species. Several specific names or infraspecific taxa have been proposed, but none of them are recognized in this work. Mexican populations of this species have been called *A.durangensis*, and are characterized by the unawned lemmas, and the presence of a short, veinless palea, but these characters overlap with populations of other regions. *Agrostisexarata* is often confused with several *Polypogon* species, which are often sympatric, but differs from them in the spikelets disarticulating above the glumes (vs. disarticulation below the glumes, with a pedicel fragment). Mexican populations of *A.exarata* are sometimes confused with some individuals of *A.tolucensis* with wide leaf blades, but differ from it in the spikelets often with a palea, and lemma usually unawned (vs. spikelets without a palea, lemma awned in *A.tolucensis*). This species is also confused with *A.blasdalei* and *A.densiflora* (see the notes in excluded species).

##### Conservation status.

*Agrostisexarata* is a widespread species in the study zone. It is represented by 61 collections, with several populations occurring in 13 protected areas. The EOO is 1,402,821 km^2^ and the AOO is 188 km^2^. Following the IUCN criteria, the preliminary assessment category is Least Concern (LC).

##### Representative specimens examined.

**Mexico. Baja California. Municipio Ensenada**, Sierra San Pedro Mártir, head of arroyo Copal, steep canyon on E sidelong crest of range, 1.5 km N of cerro Observatorio, [31.04418346°N, 115.463326°W, 2800 m alt.], 16 Jul 1988, S. Boyd and T. Ross 2544 (F, MEXU [*], NY). **Baja California Sur: Municipio Comondú**, along small dry streambed, on open rolling ridge tops, La Chuparosa, [26.22975°N, 111.97158°W, 350 m alt.], 12 Apr 1955, A. Carter and R.S. Ferris s.n. (SD). **Chihuahua: Municipio Ocampo**, Parque Nacional de la Cascada de Basaseachi, [28.16666667°N, 108.2083333°W], 1600 m alt., 26 Apr 1985, R. Spellenberg et al. (MEXU), 9056 (MEXU). **Without municipality**, by springs, Sierra Madre, 2900 m alt., 3 Oct 1887, C.G. Pringle 1421 (F, MEXU). **Coahuila**: **Municipio Zaragoza**, Sierra del Carmen, Canyon de Sentenela [Centinela] on Hacienda Piedra Blanca, [29.11021676°N, 101.7054129°W, 860 m alt.], 6 Jul 1936, F.L. Wynf and C.H. Muller 547 (MSC). **Durango: Municipio San Dimas**: Vencedores, camino a las cabañas, 24.44944444°N, 105.7758333°W, 2355 m alt., 25 Aug 2013, S. Heynes 587 (MEXU [*,**]); **Guanajuato: Municipio San Felipe**, Vergel de la Sierra, [21.38504722°N, 101.6354389°W], 2440 m alt., 3 Sep 1981, R. Guzmán 4543 (MEXU [*,**]), R. Santillán 154-R (MEXU). **Hidalgo: Municipio Acaxochitlán**, San Francisco, [20.1897756°N, 98.14532383°W], 2000 m alt., 30 May 1985, A. Villa 186 (MEXU). **Sonora**: Sonora, 24 Jun 1855, A. Schott s.n. (F). **USA. Arizona: Cochise County**, Rucker Canyon, [31.757451°N, 109.360505°W], 2495 m alt., 28 May 1952, E.R. Blakley 1301 (DES). **California: San Diego County**, Cuyamaca Rancho State Park, 0.4 mile NE of intersection of state highway 79 and Stonewall Mine road, ca. 100 m NE of Los Caballos equestrian campground and 20 m south of Stonewall Mine road in north-treading drainage, 32.9728°N, 116.5714°W, 1440 alt., 26 Jun 2005, L. Hendrickson 1093 (BSCA, SD [*,**]). **New Mexico: Grant County**, Mimbres River, [32.858382°N, 107.977694°W], 1676 m alt., 1 Jul 1904, O.B. Metcalfe 1073 (F, NY). **Texas: Brewster County**, Lower Oak Creek, Chisos Mountains, [29.266025°N, 103.301199°W, 1700 m alt.], 6 Jul 1937, B.H. Warnock 20153 (TEX). See Suppl. materials 2, 3 for additional examined specimens.

#### ﻿6. Agrostis ghiesbreghtii

Taxon classificationPlantaePoalesPoaceae

E. Fourn., Mexic. Pl. 2: 97. 1886.

B42D528E-D154-500A-8801-E30F08BCE890

Figs 4F , 5F , 16

Agrostis ghiesbreghtii E. Fourn. ex Hemsl., Biol. Cent.-Amer., Bot. 3: 551. 1885, nom. nud.
= Agrostis setifolia E. Fourn., Mexic. Pl. 2: 97. 1886, nom. illeg. hom., non Brot., 1804. Agrostissetifolia E. Fourn. ex Hemsl., Biol. Cent.-Amer., Bot. 3: 551. 1885, nom. nud. Type. México. Veracruz: pic d’Orizaba, F.M. Liebmann 712 (holotype: C (C10016729 [image!])).

##### Type.

Mexico. Oaxaca: Province d’Oaxaca, 1842, A. Ghiesbreght s.n. (holotype: P (P00740574 [image!]); isotype: US [fragm. ex P] (US00156357 [image!])).

##### Description.

***Plants*** perennial, caespitose. ***Tillers*** extravaginal, with cataphylls. ***Culms*** 30–90 cm long, erect, rarely shortly decumbent at the base, nodes 2–5, glabrous, internodes glabrous. ***Leaves*** basal and cauline; sheaths 4–10 cm long, the lower ones shorter than the internodes, the upper ones longer, glabrous; ligules 1.5–6(–12) mm long, longer than wide, dorsally scaberulous, apices acute, often lacerate; blades 3.5–27 cm long, 1–3.5(–5) mm wide, stiff, linear, convolute or involute, the upper ones sometimes flat, scaberulous on both surfaces. ***Panicles*** 8–30 cm long, 5–19 cm wide, open, lax, ovate, usually long-exserted from the upper sheaths; branches spreading, rebranching about or above mid-length, scaberulous, without spikelets near their base, inferior branches 1.5–10 cm long; pedicels 2.5–5(–10) mm long, ascending to spreading, scaberulous. ***Spikelets*** (2.5–)3–4 mm long, usually purplish; glumes subequal to unequal, lanceolate, apices acute to shortly acuminate, 1-veined, scaberulous on the keel, lower glume (2.5–)3–4 mm long, upper glume (2.2–)2.7–3.9 mm long; callus pubescent, with 2 bunches of trichomes; lemmas (1.5–)1.8–2.5 mm long, elliptic, apices irregularly toothed, 5-nerved, veins prominent, awned from near the base, awn 2.3–5 mm long, geniculate; paleas absent, or up to 0.2 mm long, veinless, glabrous; anthers 3, 1–1.5 mm long. ***Caryopsis*** 1.2–1.5 mm long, elliptic; endosperm soft. 2n= unknown.

**Figure 16.**
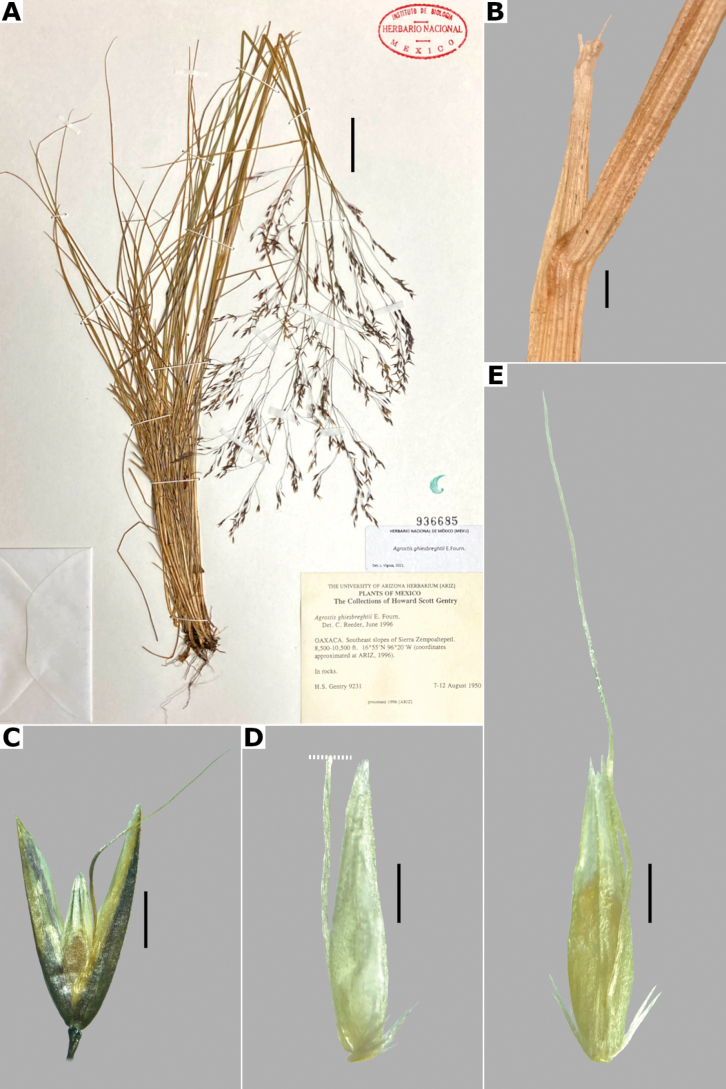
*Agrostisghiesbreghtii***A** whole plant **B** ligular area **C** spikelet **D** floret, lateral view, **E** floret, abaxial view. Based on Gentry 9231 (MEXU). Scale bars: 3 cm (**A**); 1 mm (**B**); 1 mm (**C**); 0.5 mm (**D, E**).

***Leaf anatomy*.** Leaf blades convolute, rarely flat in transversal section; adaxial furrows deep, narrow; adaxial ribs rounded; keel absent; first order bundles circular in outline, sheath interrupted adaxially and abaxially, abaxial and adaxial sclerenchyma in girders; second order bundles circular in outline, sheath interrupted abaxially, abaxial sclerenchyma in girders, narrowing towards the bundle, adaxial sclerenchyma in strands; intercostal sclerenchyma usually present, a hypodermal band, sometimes absent; leaf margins with sclerenchyma continuous with the hypodermal band; colorless cells absent (Fig. 15D, E). Lemmas with transversal thickenings oblong, wider than the unthickened portions of the wall; prickle hairs abundant (Fig. 7F).

##### Distribution and habitat.

Endemic. *Agrostisghiesbreghtii* is distributed from central Mexico to Guatemala. In Mexico, it has been collected in Mexico City and the states of Chiapas, Guanajuato, Guerrero, Hidalgo, México, Michoacán, Morelos, Oaxaca, Puebla and Veracruz (Fig. 8F). It is also reported from Chihuahua and Durango (Sánchez-Ken 2019), but no specimens from these states were seen. In Guatemala, it has been collected in the departments of Quetzaltenango and Sacatepéquez. This species grows in open areas of temperate forests, with *Abies*, *Pinus*, *Quercus*, and alpine grasslands, between 1110–3700 m a.s.l. (Fig. 9I).

##### Phenology.

Flowering and fruiting specimens have been collected year round, but most of them between the months of August and February (Fig. 10I).

##### Commentaries.

This species is similar to *A.laxissima* in the open panicles, awned lemmas and lemma micromorphological features, but it is distinguished in the leaf blades stiffer, usually convolute or involute, with deep adaxial furrows and usually with a hypodermal bland of abaxial sclerenchyma, and usually larger spikelets of (2.5–)3–4 mm long (vs. leaf blades lax, flat, with medium-sized adaxial furrows, without intercostal sclerenchyma, spikelets of 1.7–3 mm long in *A.laxissima*). Some specimens with unusually wide and flat upper leaf blades have been collected in Guerrero and Chiapas, but the other characters are congruent with *A.ghiesbreghtii*. This species is also very similar to *A.mertensii*, and more studies are needed to define its taxonomic boundaries (see the notes in excluded species).

##### Conservation status.

*Agrostisghiesbreghtii* is a widespread species in the study zone. It is represented by 80 collections, with several populations occurring in 12 protected areas. The EOO is 240,863 km^2^ and the AOO is 176 km^2^. Following the IUCN criteria, the preliminary assessment category is Least Concern (LC).

##### Representative specimens examined.

**Guatemala. Sacatepéquez: Distrito Alotenango**, Volcán de Acatenango, [14.499453°N, 90.87175°W], 3700 m alt., 10 Apr 2000, M. Véliz et al. 8303 (MEXU), 8371 (MEXU). **Quetzaltenango: Distrito Quetzaltenango**, Volcán Santa María, upper NE facing slopes to summit of volcano, [14.7583°N, 91.5492°W], 3600 m alt., 13 Jan 1940, J.A. Steyermark 34160 (F). **Mexico. Chiapas: Municipio Motozintla**, near summit of cerro Mozotal, [15.42605394°N, 92.34362676°W], 2750 m alt., 24 Nov 1981, D.E. Breedlove 55885 (MICH, MEXU [*], TEX), 55893 (MEXU, MO). **Guanajuato: Municipio Xichú**, Sierra de Xichú, [21.28736944°N, 100.0888472°W], 1900 m alt., 19 Sep 1981, A. Mora 281-AMB (MEXU). **Guerrero: Municipio General Heliodoro Castillo**, Escalerilla, [17.47217778°N, 100.0386778°W], 2550 m alt., 1 Nov 1998, N. Diego 8296 (FCME [*], MEXU); cerro Teotepec, [17.46666667°N, 100.2166667°W], 3350 m alt., 11 Apr 1963, J. Rzedowski 18137 (ENCB, F, IEB, MICH). **Hidalgo: Municipio Mineral del Chico**, alrededores de Las Ventanas, 5 km al N de Pachuca, [20.19252776°N, 98.73954093°W], 2900 m alt., 2 Nov 1983, S Acosta et al. 416 (FCME, MEXU [*,**], UAMIZ, XAL). **Municipio Zimapán**, 11 km al S de La Luz, [20.733333°N, 99.366667°W], 1100 m alt., 10 Jan 1991, V.M. Huerta 1165 (CIB, IEB, XAL). **México: Municipio San Simón de Guerrero**, 3 km sobre la desviación de Simón de Guerrero, carretera hacia Sultepec, [19.01857206°N, 100.0280164°W], 2870 m alt., 7 Feb 1984, E. Manrique et al. 673 (MEXU). **Mexico City: Alcaldía Cuajimalpa**, loma La Vaquera (arroyo Agua de Leones), 3220 m alt., 16 Oct 1985, A. Miranda and P. Guerrero 136a (MEXU). **Michoacán: Municipio Contepec**, cerro Altamirano, Reserva de la Biósfera Mariposa Monarca, 19.96667°N, 100.13333°W, 3027 m alt., 6 Mar 2005, J. Martínez 1453 (IEB, MEXU [**]). **Morelos: Municipio Huitzilac**, Zempoala, [19.03333333°N, 99.3°W], 3000 m alt., 3 Nov 1951, E. Matuda 26005 (MEXU, US). **Oaxaca: Municipio Santa María Tlahuitoltepec**, SE slopes of Sierra Zempoaltepetl, [17.129956°N, 96.01353°W], 3300 m alt., 7 Aug 1950, H.S. Gentry 9231 (MEXU [*]). **Municipio San Martín Peras**: Santiago Juxtlahuaca, San Martín Peras, carretera Tecomaxtlahuaca-San Martín Peras, 2 km de la intersección a Coicoyán de las Flores, 17.2963333°N, 98.195528°W, 2570 m alt., 17 Oct 1994, J.L. Panero et al. 5117 (MEXU [*], TEX). **Puebla: Municipio Atzitzintla**, Teamalaquilla [Texmalaquilla], [18.943056°N, 97.2875°W], 3100 m alt., 29 Aug 1938, E.K. Balls 5393 (MICH, MSC, US). **Veracruz: Municipio Calcahualco**, barranca de San Miguel Tlacotiopa, [19.11192082°N, 97.20509861°W], 2700 m alt., 19 Jan 1989, P. Tenorio 15470 (CIIDIR, MEXU [*,**]). See the Suppl. material 2 for additional examined specimens.

#### ﻿7. Agrostis gigantea

Taxon classificationPlantaePoalesPoaceae

Roth, Tent. Fl. Germ. 1: 31. 1788.

CDB60DBF-B8F0-569B-BF0F-FB9D88428E73

Figs 4G , 5G , 17

Triticum giganteum (Roth) Roth, Catal. Bot. 3: 22. 1806.
Vilfa gigantea (Roth) P. Beauv., Ess. Agrostogr. 16: 147. 1812.
Agrostis alba L. var. aristata Spenn., Fl. Friburg. 1: 94. 1825.
Agrostis stolonifera L. subsp. gigantea (Roth) Schübl. & G. Martens, Fl. Würtemberg (ed. 1) 64. 1834.
Agrostis stolonifera L. var. gigantea (Roth) Bréb., Fl. Normandie 390. 1835.
Agrostis alba L. var. gigantea (Roth) G. Mey., Chloris Han. 655. 1836, non Spenner 1825.
Agrostis alba L. subsp. gigantea (Roth) Arcang., Comp. Fl. Ital. 768. 1882.
= Agrostis virletii E. Fourn., Mexic. Pl. 2: 96. 1886. Agrostisvirletii E. Fourn. ex Hemsl., Biol. Cent.-Amer., Bot. 3: 552. 1885, nom. nud. Type. Mexico. San Luis Potosí: Prov. de San Luis, 1851, *M. Virlet d’Aoust 1345* (lectotype, designated by Vigosa-Mercado (2022a: 2): P (P00740442 [image!]); isolectotypes: P (P00740440 [image!], P00740441 [image!]), US [fragm. ex P] (US00156515 [image!])).
Agrostis stolonifera L. subsp. gigantea (Roth) Maire & Weiller, Fl. Afrique N. 2(XLV): 120. 1953.
Agrostis stolonifera L. subsp. gigantea (Roth) Beldie, Fl. Republ. Socialiste Romania 12: 152. 1972, nom. illeg. hom.^*^

##### Type.

Germany. Herb. Albrecht de Haller, A.W. Roth s.n. (lectotype, designated by Widén (1971: 97): G (G00195254 [image!])).

##### Description.

***Plants*** perennial, rhizomatous. ***Tillers*** extravaginal, with cataphylls. ***Rhizomes*** up to 25 cm long. ***Culms*** 0.2–1.2 m long, erect, sometimes geniculate, nodes 3–7, glabrous, internodes glabrous. ***Leaves*** basal and cauline; sheaths 4.5–13 cm long, usually shorter than the internodes, glabrous or scaberulous; ligules 1–7 mm long, longer than wide, dorsally scaberulous, apices rounded to truncate, erose to lacerate; blades (2–)3.5–20 cm long, 1–8 mm wide, usually at least some blades larger than 5 mm wide, linear, flat, scaberulous on both surfaces. ***Panicles*** (9–)13–40 cm long, 4–16 cm wide, open, dense to lax, lanceolate to ovate, exserted from the upper sheaths; branches ascending to spreading, rebranching about or above mid-length, scaberulous, without spikelets near their base or often some inferior branches with spikelets, inferior branches 2.5–15 cm long; pedicels 0.3–3 mm long, ascending to spreading, scaberulous. ***Spikelets*** 1.7–3 mm long, greenish to purplish; glumes subequal to unequal, lanceolate to ovate, apices acute to shortly acuminate, 1-veined, scaberulous on the keel, lower glume 1.7–3 mm long, upper glume 1.3–2.8 mm long; callus glabrous or with 2 bunches of few, very short trichomes, inconspicuous; lemmas 1.4–2 mm long, elliptic to oblong, apices entire, acute or sometimes truncate, 3–5-veined, veins inconspicuous or prominent distally, usually unawned; paleas present, 0.5–1.2 mm long, faintly 2-veined, glabrous; anthers 3, 0.7–1.2 mm long. ***Caryopsis*** 1–1.5 mm long, elliptic, endosperm solid. 2n= 42 (Harvey 2007).

**Figure 17.**
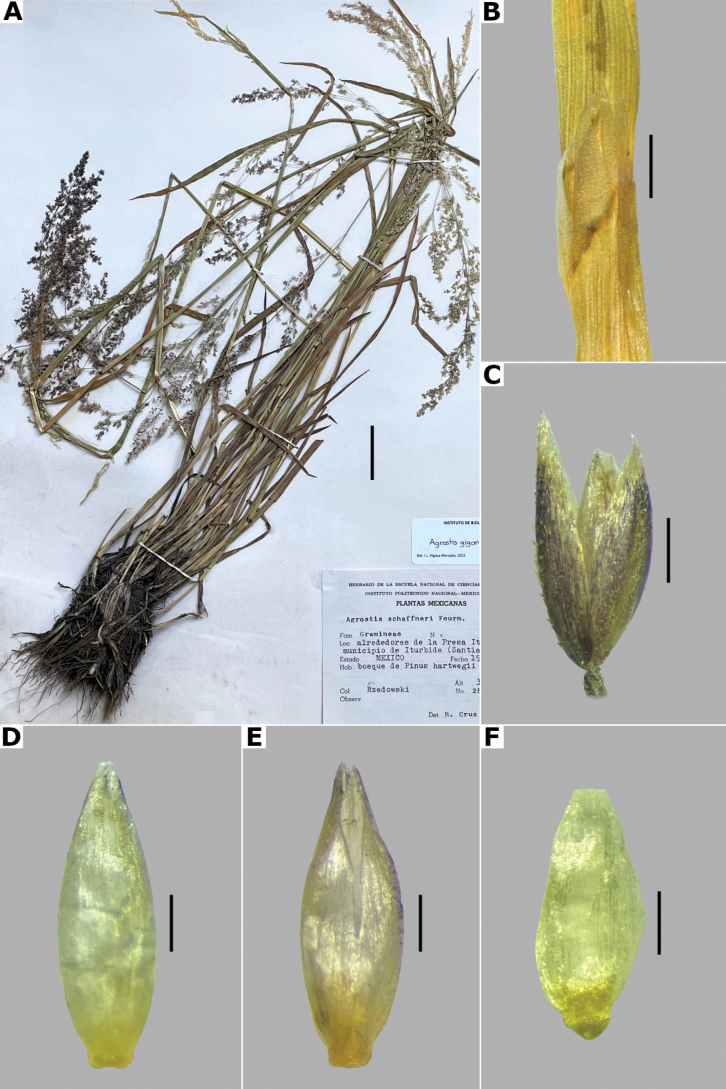
*Agrostisgigantea***A** whole plant **B** ligular area **C** spikelet **D** floret, abaxial view, **E** floret, adaxial view **F** palea. Based on Rzedowski 28545 (IBUG). Scale bars: 3 cm (**A**); 2 mm (**B**); 0.5 mm (**C**); 0.3 mm (**D, E**); 0.2 mm (**F**).

##### Anatomy and micromorphology.

Leaf blades flat in transversal section; adaxial furrows medium-sized, wide; adaxial ribs rounded; keel absent; first order bundles circular in outline, sheath interrupted adaxially and abaxially, abaxial and adaxial sclerenchyma in girders, narrowing towards the bundle; second order bundles circular in outline, sheath interrupted adaxially and abaxially, abaxial and adaxial sclerenchyma in girders, narrowing towards the bundle; intercostal sclerenchyma absent; leaf margins with small to well-developed sclerenchyma caps, rounded; colorless cells absent (Fig. 15F–H). Lemmas without transversal thickenings, or these poorly developed; prickle hairs absent or scarce (Fig. 7G).

##### Distribution and habitat.

**Figure 18.**
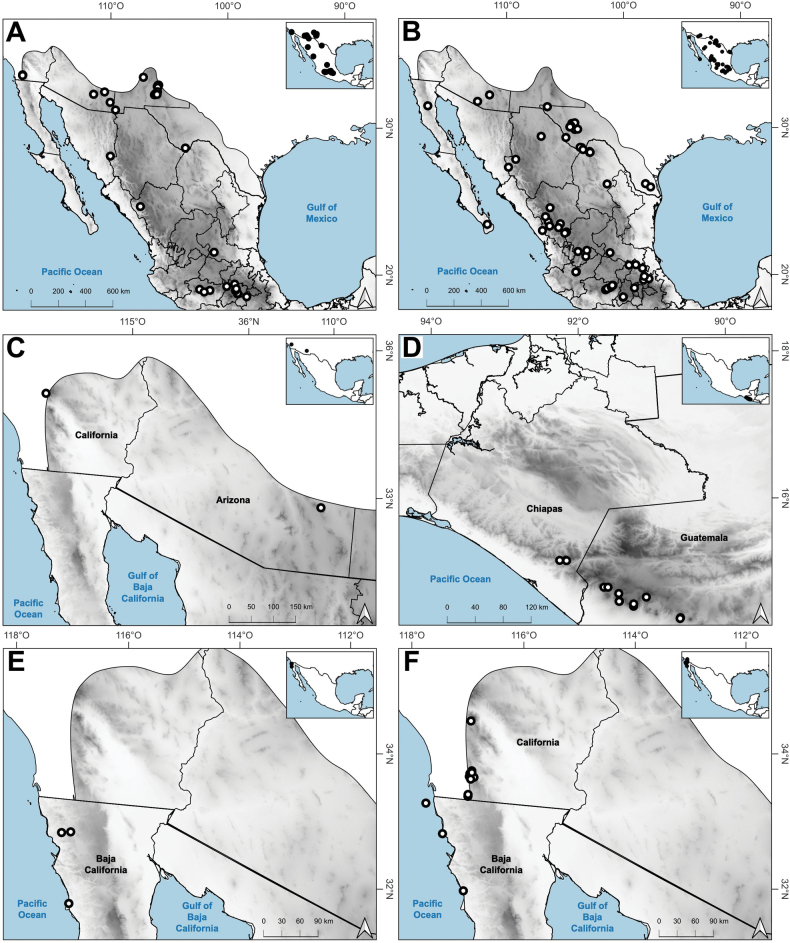
Map of known geographic distribution of *Agrostis* species, based on herbarium specimen data **A***A.gigantea***B***A.hyemalis***C***A.idahoensis***D***A.laxissima***E***A.microphylla***F***A.pallens*.

Introduced. *Agrostisgigantea* is native to Eurasia. In the study zone, it has been collected in the USA states of Arizona, California, New Mexico, Texas, and in Mexico City, and the Mexican states of Durango, México, Michoacán, San Luis Potosí, and Sonora (Fig. 18A). This species has also been reported from Guanajuato, Morelos, and Oaxaca (Sánchez-Ken 2019), but no specimens from these states were found. *Agrostisgigantea* grows in disturbed areas, mainly in moist soils of ditches, marshy places and stream edges, between 651–3300 m a.s.l. (Fig. 9J).

##### Phenology.

Specimens with spikelets have been collected from June to December (Fig. 10J).

##### Commentaries.

It has been reported for other regions that the blades can reach 12 mm wide, spikelets up to 3.2 mm long and lemmas up to 2.2 mm long (Harvey 2007). This species is often confused with *A.bourgaei* (see the note under the description of that species). *Agrostisgigantea* also is similar to *A.stolonifera* in the paleate spikelets, but differs from it in the rhizomatous habit of the plants and more open panicle (vs. stoloniferous plants, panicle usually contracted in *A.stolonifera*).

The rhizomatous plants of *Agrostis*, with paleate spikelets, were formerly known as *A.alba* L., a name that was described without a type designation (Linnaeus 1753). Hitchcock (1905) considered the specimen LINN 84.23 as the type, but this was received by Linnaeus long after 1753 (Jarvis 2007). Widén (1971) indicates that this name must be typified by a specimen at the Van Royen Herbarium (L0052692), since *A.alba* was based on the work of Royen (1740), but that specimen corresponds to *Poanemoralis* L., so currently *A.alba* is a synonym of the latter.

##### Conservation status.

Since *Agrostisgigantea* is an introduced species in the study zone, its conservation status is considered as Least Concern (LC).

##### Representative specimens examined.

**Mexico. Durango: Municipio Canelas**, Las Cebollitas, [25.10273363°N, 106.4441947°W], 2460 m alt., 1 Aug 1990, A. Benítez 1725 (CIIDIR, MEXU, UAMIZ). **México: Municipio Amecameca**, km 15 carretera Amecameca-Tlamacas, [19.08516405°N, 98.68113865°W, 3288 m alt.], 2 Oct 1992, A. Miranda and G. Villegas 634 (MEXU [*]). **Municipio Isidro Fabela**, alrededores de la presa Iturbide, [19.52454734°N, 99.46880154°W], 3300 m alt., 19 Aug 1971, J. Rzedowski 28545 (IBUG [*, **]). **Municipio Jilotepec**, Jilotepec, [19.96787783N, 99.51665054], 2450 m alt., 27 June 1954, E. Matuda 30961 (MEXU [*, **]). **Mexico City: Alcaldía Cuajimalpa**, La Venta, Santa Rosa-Contreras, [19.33155°N, 99.31138889°W], 2600 m alt., 29 Jul 1951, E. Matuda 21271 (MEXU [*, **]). **Michoacán: Municipio Salvador Escalante**, Santa Clara del Cobre, [19.41071944°N, 101.6532583°W], 2150 m alt., E. Pérez 96 (IEB, MEXU). **Sonora: Municipio Yécora**, 5.2 km W of Yécora on Mex 16, 28.36184916N, 108.961472, 1720 m alt., 1 Jun 1999, A.L. Reina et al. 99–160 (TEX). **USA. Arizona: Graham County**, Hospital Flat, Pinaleno Mountains, 32.6651°N, 109.877°W, 700 m alt, 7 Sep 1980, C.E. Jenkins and G. Yatskievych 3120 (ASU). **California: San Diego County**, Cuyamaca Rancho State Park, Cuyamaca Peak, 1 mi E of highway 79, beside Fern Flat Fire Road (unpaved), 0.5 mi SE of intersection with Lookout Fire Road and 0.8 air mile ENE of summit, 32.9487°N, 116.5935°W, 1586 m alt., 18 Sep 2008, L. Hendrickson 3272 (SD). **New Mexico. Socorro County**, Bosque del Apache refuge, San Antonio, [33.917844°N, 106.865859°W], 651 m, 7 Dec 1940, L. Lee s.n. (UNM). **Texas: Brewster County**, along creek from Boot Springs toward Boot, Chisos Mountains, [29.2724465°N, 103.2657998°W], 2065 m alt., 26 Aug 1937, B.H. Warnock 1039 (MICH). See Suppl. materials 2, 3 for additional examined specimens.

#### ﻿8. Agrostis hyemalis

Taxon classificationPlantaePoalesPoaceae

(Walter) Britton, Sterns & Poggenb., Prelim. Cat. 68. 1888.

5300DB20-A08C-5F46-ABBB-D91C856B98D5

Figs 4H , 5H , 19

= Cornucopiae hyemalis Walter, Fl. Carol. 73. 1788. AgrostiscaninaL.var.hyemalis (Walter) Kuntze, Revis. Gen. Pl. 3(3): 338. 1898. Agrestishyemalis (Walter) Lunell, Amer. Midl. Naturalist 4: 216. 1915. Type: USA. South Carolina: Charleston, sandy open ground near Navy Yard, 27 Apr 1912, B.L. Robinson 97 (neotype, designated by Ward (2007: 1099): GH (GH00247993 [image!]): isoneotypes: BH, US (US00955689 [image!])).^*^

##### Type.

Based on *Cornucopiaehyemalis* Walter.

##### Description.

***Plants*** perennial, caespitose. ***Tillers*** extravaginal, with cataphylls. ***Culms*** 15–90 cm long, erect, sometimes shortly decumbent at the base, nodes (2–)3–7, glabrous, internodes glabrous. ***Leaves*** basal and cauline; sheaths 5–11 cm long, usually shorter than the internodes, glabrous or scaberulous; ligules 1–7 mm long, longer than wide, dorsally scaberulous, apices rounded to truncate, erose, sometimes acute, often lacerate; blades 3–10(–15) cm long, 1–2(–3) mm wide, linear, flat, often becoming involute when dry, scaberulous on both surfaces. ***Panicles*** 10–30 cm long, (1.5–)4–25(–30) cm wide, open, lax, ovate, exserted from the upper sheaths, sometimes partially included; branches spreading, sometimes ascending, rebranching in the upper third, scaberulous, without spikelets near their base, spikelets clustered at the tips, inferior branches up to 15 cm long; pedicels (0.1–)0.5–2(–3.5) mm long, appressed, scaberulous. ***Spikelets*** 1–2(–2.5) mm long, greenish to purplish; glumes subequal to unequal, lanceolate, apices acute to shortly acuminate, 1-veined, scaberulous on the keel, sometimes also on the body, lower glume 1–2(–2.3) mm long, upper glume 0.8–1.9(–2.2) mm long; callus pubescent, with two bunches of trichomes; lemmas 0.8–1.3(–1.4) mm long, elliptic, apices entire, acute to obtuse, sometimes truncate, 5-nerved, veins inconspicuous or prominent, unawned; paleas absent or up to 0.2 mm long, veinless, glabrous; anthers 3, 0.2–0.5 mm long. ***Caryopsis*** 0.6–1.2 mm long, elliptic; endosperm soft. 2n= 28 (Harvey 2007).

**Figure 19.**
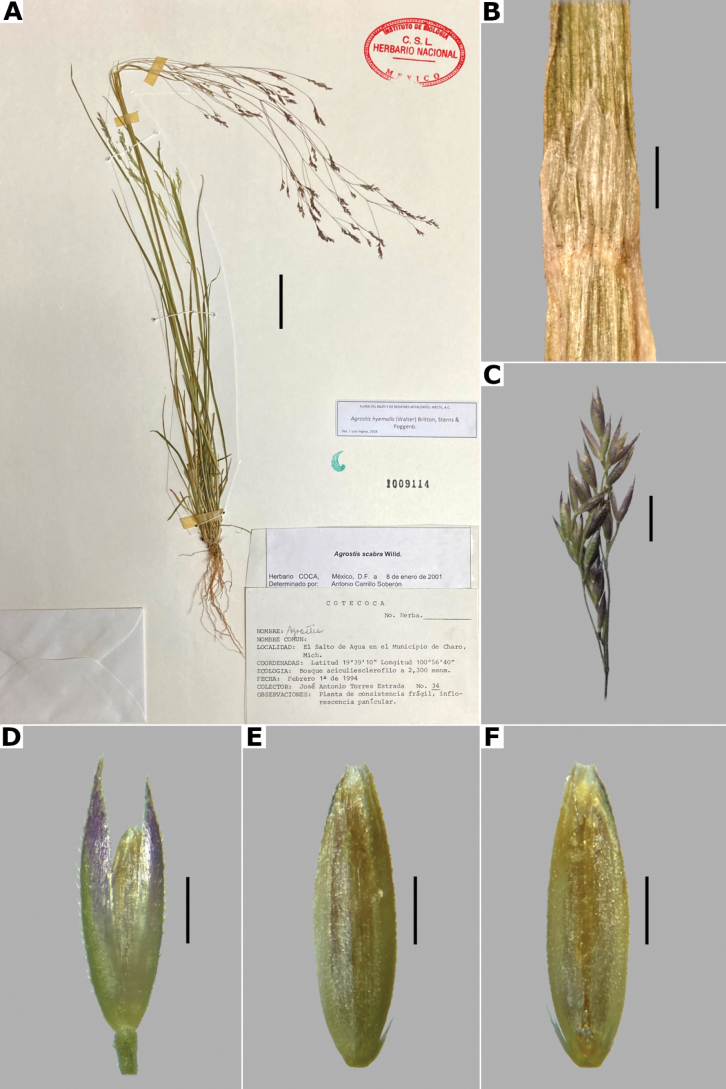
*Agrostishyemalis***A** whole plant **B** ligular area **C** detail of a terminal branch of the panicle **D** spikelet, **E** floret, abaxial view, **F** floret, adaxial view. Based on Torres 34 (MEXU). Scale bars: 3 cm (**A**); 2 mm (**B, C**); 0.5 mm (**D**); 0.3 mm (**E, F**).

##### Anatomy and micromorphology.

Leaf blades flat in transversal section; adaxial furrows medium-sized, wide; adaxial ribs rounded; keel absent; first order bundles circular in outline, sheath interrupted adaxially and abaxially, abaxial and adaxial sclerenchyma in girders, narrowing towards the bundle; second order bundles circular in outline, sheath interrupted abaxially, abaxial sclerenchyma absent or in girders, narrowing towards the bundle, adaxial sclerenchyma absent or in strands; intercostal sclerenchyma absent; leaf margins with well-developed sclerenchyma caps, rounded; colorless cells absent (Fig. 15I–K). Lemmas with transversal thickenings oblong, wider than the unthickened portions of the wall; prickle hairs abundant to scarce (Fig. 7H).

##### Distribution and habitat.

*Agrostishyemalis* is distributed from Ontario province in Canada to central Mexico, and is also present in the West Indies and Ecuador (Harvey 2007). The records of this species in western North America could represent the confusion with *A.scabra* (see note below). It has also been reported from Perú (Soreng and Peterson 2003). In the study zone, this species has been collected in southern Arizona and Texas, USA, and in the Mexican states of Aguascalientes, Baja California, Baja California Sur, Chihuahua, Coahuila, Durango, Hidalgo, Jalisco, México, Michoacán, Puebla, Querétaro, San Luis Potosí and Sonora (Fig. 18B). It has also been reported from the Mexico City and the states of Colima, Guanajuato, Morelos, Nuevo Leon, Oaxaca, Sinaloa, Tlaxcala and Veracruz (Villaseñor 2016), but no specimens from these states were found. *Agrostishyemalis* grows in open areas of temperate forests, with *Abies*, *Juniperus*, *Pinus* or *Quercus*, cloud forests, rocky areas of grasslands and stream edges, between 14–2710 m a.s.l. (Fig. 9K). The Texan populations of this species grow in lower elevations than the Mexican ones.

##### Phenology.

Specimens with spikelets have been collected from January to November, but most of the records are from May (Fig. 10K).

##### Commentaries.

It has been reported for other regions that the panicles can reach 36 cm long (Harvey 2007).

*Agrostishyemalis* is often confused with *A.elliottiana* (see the note under the description of that species). It is also similar to *A.scabra*, in the panicle branches rebranching in the upper third and somewhat clustered spikelets, and also shares several leaf blade anatomy features. *Agrostishyemalis* differs from the latter in the culms with usually more than three nodes, basal and cauline leaves, more clustered and shorter spikelets, of 1–2(–2.5) mm long, and smaller anthers of 0.2–0.5 mm long (vs. culms with usually 1–2(–3) nodes, usually mostly basal leaves, spikelets of 2–3(–3.4) mm long, anthers 0.5–1.4 mm long in *A.scabra*). The identification of these two species is especially difficult if the plants are not collected with the basal parts.

##### Conservation status.

*Agrostishyemalis* is a widespread species in the study zone. It is represented by 86 collections, with several populations occurring in 15 protected areas. The EOO is 1,491,700 km^2^ and the AOO is 248 km^2^. Following the IUCN criteria, the preliminary assessment category is Least Concern (LC).

##### Representative specimens examined.

**Mexico. Aguascalientes: Municipio Calvillo**, margen Presa Los Adobes, 21.805°N, 102.6886111°W, 1960 m alt., 29 Mar 2010, F. Macías 5 (FCME, IEB, INEGI, MEXU [*], UAMIZ). **Baja California: Municipio Ensenada**, Los Llanitos, [30.96666667°N, 115.4333333°W], 2550 m alt., 17 Aug 1967, R. Moran and R.F. Thorne 14267 (MEXU). **Baja California Sur**: **Municipio La Paz**, Sierra de la Laguna, arroyo la Boquilla, [23.53333°N, 109.9°W], 1850 m alt., 2 Jun 1995, M. Domínguez 1009 (SD [*]). **Chihuahua: Municipio Chihuahua**, rancho El Peñasco, km 150 carretera Chihuahua-Ciudad Juárez, [29.875°N, 106.375°W], 750 m alt., 9 Aug 1979, M.E. Siqueiros 340 (MEXU [*]). **Municipio Chínipas**, rancho Byerly, Sierra Charuco, [27.581389°N, 108.696111°W], 1767 m, Apr 1948, H.S. Gentry 8027 (MEXU, US). **Coahuila: Municipio Ocampo**, Madera del Carmen, 0.5 mi from Campo Uno, up the road towards the summit, 28.99611111°N, 102.6113889°W, 2355 m alt., 22 Sep 2007, P.M. Peterson et al. 21016 (CAN, MEXU [*, **], US); **Durango: Municipio Durango**, predio Las Bayas (UJED), arroyo San Rafael, 23.44416667°N, 105.8775°W, 2710 m alt., 8 Aug 1990, A. García and M. González 610 (CIIDIR). **Municipio Suchil**, arroyo El Toboso, potrero Jacales, San Juan de Michis, [23.432778°N, 104.132778°W], 2220 m alt., 3 Jan 1986, J. Alvarado 711 (CIIDIR, IBUG, IEB, MEXU [*], UAMIZ). **Hidalgo: Municipio Huasca de Ocampo**, 0.5 km al W de Bermúdez, sobre el camino de terracería que conduce de Bermúdez a Huasca de Ocampo, [20.1975°N, 98.58861111°W], 2230 m alt., 21 Jul 1994, M. Osorio 27 (MEXU). **Jalisco: Municipio Gudalajara**, periférico de Guadalajara, cercano al auditorio Benito Juárez, [20.72784467°N, 103.3330636°W], 1540 m alt., 13 Nov 1975, C. García 238 (IBUG). **México: Municipio Cuautitlán**, alrededores de Cuautitlán, [19.65834784°N, 99.22657358°W], 2250 m alt., 5 Jun 1982, J. Rzedowski 37841 (CIIDIR, IEB, INEGI, MEXU [*], XAL). **Municipio San Simón de Guerrero**: 3 km sobre la desviación a San Simón de Guerrero, por la carretera Temascaltepec-Tejupilco, [19.01841341°N, 100.0282873°W], 1974 m alt., 15 Mar 1983, E. Manrique et al. 207 (MEXU [*]). **Michoacán: Municipio Charo**: cerca de Pontezuela, 25 km al E de Morelia, sobre la carretera a Mil Cumbres, [19.65730833°N, 100.9891889°W], 2200 m alt., 29 Jan 1987, J. Rzedowski 42417 (CHAPA, CIIDIR, ENCB, FCME, IBUG, IEB, MEXU); El Salto de Agua, 2300 m alt., 1 Feb 1994, J.A. Torres 34 (MEXU [*,**]). **Puebla: Municipio Honey**, 1 km al E de Ocahuales, carretera a Pahuatlán, [20.28333333°N, 98.2°W], 1880 m alt., 4 May 1989, P. Tenorio 15742 (IEB, MEXU, NY, TEX). **Querétaro: Municipio Landa**, 5 km al S de El Lobo, sobre el camino a Agua Zarca, [21.26118889°N, 99.11667778°W], 1500 m alt., 21 Feb 1987, J. Rzedowski 42572 (IEB). **San Luis Potosí: Municipio San Luis Potosí**, Cañada de Lobos, Sierra de San Miguelito, 5 km al S de la ciudad de San Luis Potosí, [22.09433995°N, 100.9645498°W], 1900 m alt., 1968, F. Takaki 2170 (MEXU). **Sonora: without municipality**, Sonora, 24 Jun 1855, A. Schott s.n. (F). **Zacatecas: Municipio Guadalupe**, ladera N del cerro de la Virgen, 215 m, 10 Aug 1988, J. Balleza 1580 (CHAPA). **USA. Arizona: Pima County**, Forest Cabin, Baboquivari Mountains, [31.7897°N, 111.586°W], 2091 m alt., 14 May 1941, C. Goodding 105-41 (ASU). **Texas: Hidalgo County**, ca. 1.3 airmiles SW of junction of Hidalgo, Kennedy and Willacy Counties, Hunke Ranc, La Sal Vieja Quadrangle, 26.59958°N, 97.97172°W, 14 m alt., 16 Mar 2004, W.R. Carr and M. Pons 22785 (TEX). **Jeff Davis County**, NW flank of Mount Livermore, ca. 0.3 mi ESE of Madrea Tank, ca. 0.7 mi, NNW of summit of Baldy Peak, 30.64722°N, 104.17833°W, 1890 m alt., 11 Aug 2000, W.R. Carr 19093 (TEX [*]). See Suppl. materials 2, 3 for additional examined specimens.

#### ﻿9. Agrostis idahoensis

Taxon classificationPlantaePoalesPoaceae

Nash, Bull. Torrey Bot. Club 24(1): 42–43. 1897.

8094F473-8335-58A8-8638-6950C47041A8

Fig. 20

= Agrostis tenuis Vasey, Bull. Torrey Bot. Club 10(2): 21. 1883, nom. illeg. hom., non Sibth., 1794. Agrostistenuiculmis Nash, Mem. New York Bot. Gard. 1: 32. 1900. Type: USA. California: San Bernardino County, on the San Bernardino Mountains, Aug 1881 or 1882, S.B. Parish and W.F. Parish 1085 (holotype: US (US00131119 [image!]).
= Agrostis filiculmis M.E. Jones, Contr. W. Bot. 14: 13. 1912. Type: USA. Arizona: Little De Motte Park on the Kaibab in N Arizona, 19 Sep 1894, M.E. Jones 6056bb (holotype: RSA (RSA0000394 [image!]).^*^

##### Type.

USA. Idaho: Nez Perce County, Forest, 1160 m alt., 1 Jul 1896, A.A. Heller and E.G. Heller 3431 (holotype: NY (NY00327633 [image!]); isotypes: BAA (BAA00001339 [image!]), CAS (CAS0000194 [image!]), DAO (DAO000465362 [image!]), ID (ID00157714 [image!]), JE (JE00020223[image!]), K (K000838198 [image!]), LE (LE00009307 [image!]), MO (MO-123094 [image!]), MIN (MIN1000077 [image!]), MSC (MSC0129856 [image!]), NDG (NDG07456 [image!]), NY (NY00327634 [image!]), P (P00740552 [image!], P00740553 [image!]), S (SG-259 [image!]), US (US00131762 [image!]).

##### Description.

***Plants*** perennial, caespitose. ***Tillers*** extravaginal, with cataphylls. ***Culms*** 8–40 cm long, erect, nodes 2–5, glabrous, internodes glabrous. ***Leaves*** mostly basal; sheaths 5–11 cm long, the lower ones usually shorter larger than the internodes, glabrous or scaberulous; ligules (0.7–)1–2(–4) mm long, longer than wide, dorsally scaberulous, apices rounded to truncate, erose to lacerate; blades 1–7 cm long, 0.5–2 mm wide, linear, flat, often becoming involute when dry, scaberulous on both surfaces. ***Panicles*** 3–13 cm long, 1–6(–8) cm wide, open, lax, lanceolate to ovate, exserted from the upper sheaths; branches ascending, sometimes spreading, rebranching about or slightly above mid-length, scaberulous, without spikelets near their base, inferior branches 1–4 cm long; pedicels 0.5–6.5 mm long, ascending to spreading, scaberulous. ***Spikelets*** 1.5–2.5 mm long, purplish; glumes subequal, lanceolate, apices acute to shortly acuminate, 1-veined, scaberulous on the keel, lower glume 1.5–2.5 mm long, upper glume 1.4–2.4 mm long, sometimes glabrous; callus puberulous; lemmas 1.2–2.2 mm long, elliptic, apices entire, acute to obtuse, 5-nerved, veins inconspicuous, unawned; paleas absent or up to 0.2 mm long, veinless, glabrous; anthers 3, 0.3–0.6 mm long. ***Caryopsis*** 1–1.3 mm long, elliptic; endosperm soft. 2n= 28 (Harvey 2007).

**Figure 20.**
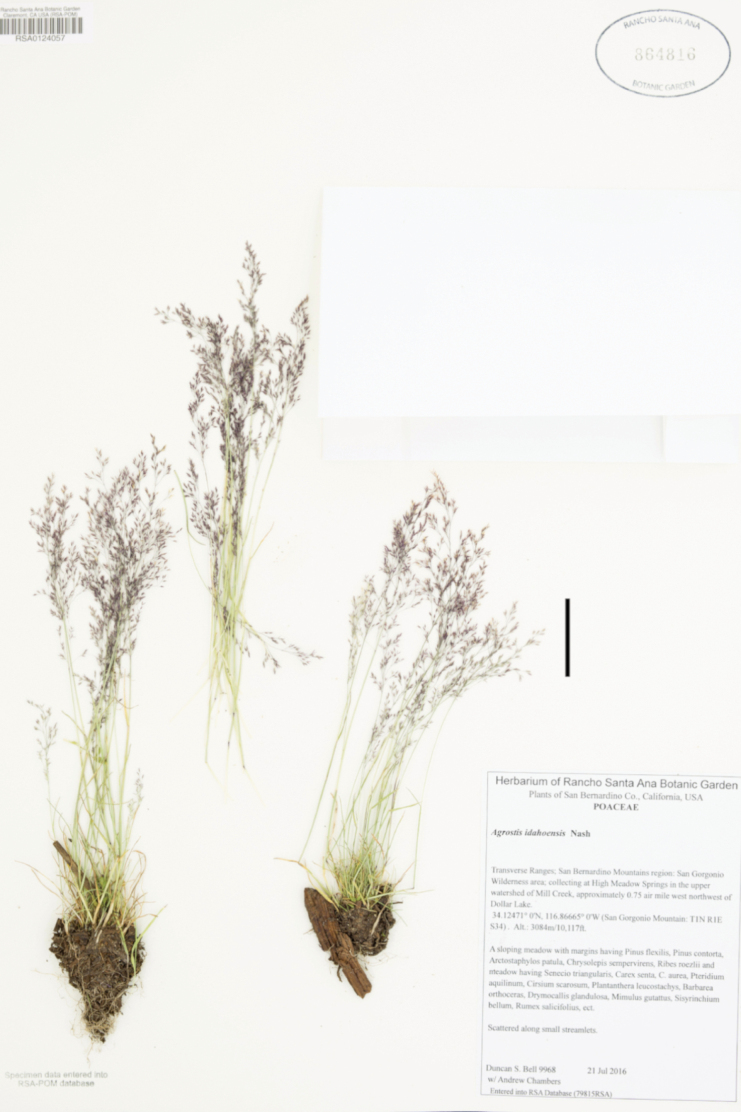
Herbarium specimen of *Agrostisidahoensis*. Based on Bell and Chambers 9968 (RSA). Scale bar: 3 mm.

##### Anatomy and micromorphology.

Not seen.

##### Distribution and habitat.

*Agrostisidahoensis* is distributed from Alaska to California and New Mexico (Harvey 2007). It is also present in Chile and Argentina (Rúgolo and Molina 1997). In the study zone, this species has been collected in southern Arizona and California (Fig. 18C). It grows in stream edges of temperate forests, with *Abies*, *Arctostaphylos*, *Picea* or *Pinus*, between 3084–3121 m a.s.l. (Fig. 9L). There are more records of this species in the study zone, on databases (GBIF 2023b), but not all of them have images, and thus we were unable to confirm their identity.

##### Phenology.

Specimens with spikelets have been collected in July and August (Fig. 10L).

##### Commentaries.

*Agrostisidahoensis* is similar to *A.perennans* sensu lato. It differs from it in the mostly basal leaves, persistent, with leaf blades 0.5–2 mm wide (vs. basal and cauline leaves, the basal ones often drying before anthesis, with leaf blades often more than 2 mm wide in *A.perennans*). It is also similar to *A.scabra*, from which it differs in the branches of the panicle rebranching about or slightly above mid-length, and spikelets not clustered at the branch tips (vs. branches rebranching in the upper third, spikelets somewhat clustered in *A.scabra*). *Agrostisidahoensis* is scarcely different from *A.turrialbae*, distributed from central Mexico to Central America. The former differs in flatter and wider leaf blades, 0.5–2 mm wide (vs. conduplicate or involute leaf blades, 0.3–0.5 mm wide in *A.turrialbae*).

##### Conservation status.

Herbarium specimens from only two localities in the United States were examined, whereby the EOO and AOO cannot be calculated. The category of Deficient Data (DD) is suggested.

##### Specimens examined.

**USA. Arizona: Graham County**, High Peak Cienega, Pinaleno Mountains, 32.693861°N, 109.867556°W, 3121 m alt., 9 Aug 2014, M. Licher 4524 (ASC). **California: San Bernardino County**, San Bernardino National Forest, San Gorgonio Wilderness area, High Meadow Springs in the upper watershed of Mill Creek, approximately 0.75 air miles west northwest of Dollar Lake, 34.12471°N, 116.86665°W, 3084 m alt., 21 Jul 2016, D.S. Bell and A. Chambers 9968 (RSA).

#### ﻿10. Agrostis laxissima

Taxon classificationPlantaePoalesPoaceae

Swallen, Contr. U.S. Natl. Herb. 29(9): 402. 1950.

B98EFC92-F89A-591D-A1D8-E1D9D7C57F94

Figs 4I , 5I , 21

= Agrostis abietorum Swallen, Contr. U.S. Natl. Herb. 29(9): 403. 1950. Type: Guatemala. San Marcos: dry banks in Cupressus-Abies forest, along road between San Sebastian at km 21 and km 8, 8–18 miles NW of San Marcos, 2700–3800 m alt., 15 Feb 1940, J.A. Steyermark 35652 (holotype: F (F0046562F [image!]); isotype: US [fragm. ex F] (US00156356)).

##### Type.

Guatemala. San Marcos: dense *Abies-Cupressus* forest, along road between San Marcos and Serchil, 2700–3150 m alt., 30 Jan 1941, P.C. Standley 85379 (holotype: US (US00131078); isotypes: F (V0046566F [image!]), US (US00131079)).

##### Description.

***Plants*** perennial, caespitose. ***Tillers*** extravaginal, with cataphylls. ***Culms*** 25–85 cm long, decumbent to erect, nodes 2–6, glabrous, internodes glabrous. ***Leaves*** basal and cauline; sheaths 4–15 cm, shorter than the internodes, scaberulous; ligules 2.5–6 mm long, longer than wide, dorsally scaberulous, apices acute, often lacerate; blades 4–15(–25) cm long, 1–4 mm wide, lax, flat, scaberulous on both surfaces. ***Panicles*** 7–18 cm long, 2–9 cm wide, open, lax, lanceolate to ovate, sometimes partially included in the upper sheaths; branches ascending to spreading, rebranching about or above mid-length, scaberulous, without spikelets near their base, inferior branches 2–7 cm long; pedicels 1.5–6 mm long, ascending to spreading, scaberulous. ***Spikelets*** 1.7–3 mm long, greenish; glumes subequal, lanceolate, apices acute to shortly acuminate, 1-veined, scaberulous on the keel, lower glume 1.7–3 mm long, upper glume 1.5–2.8 mm long; callus pubescent, with two bunches of trichomes; lemmas 1.3–2.2 mm long, elliptic, apices irregularly toothed, 5-nerved, veins inconspicuous, awned from near the base, awn 3–4 mm long, geniculate; paleas absent or up to 0.4 mm long, veinless, glabrous; anthers 3, 0.7–1 mm long. ***Caryopsis*** not seen. 2n= unknown.

**Figure 21.**
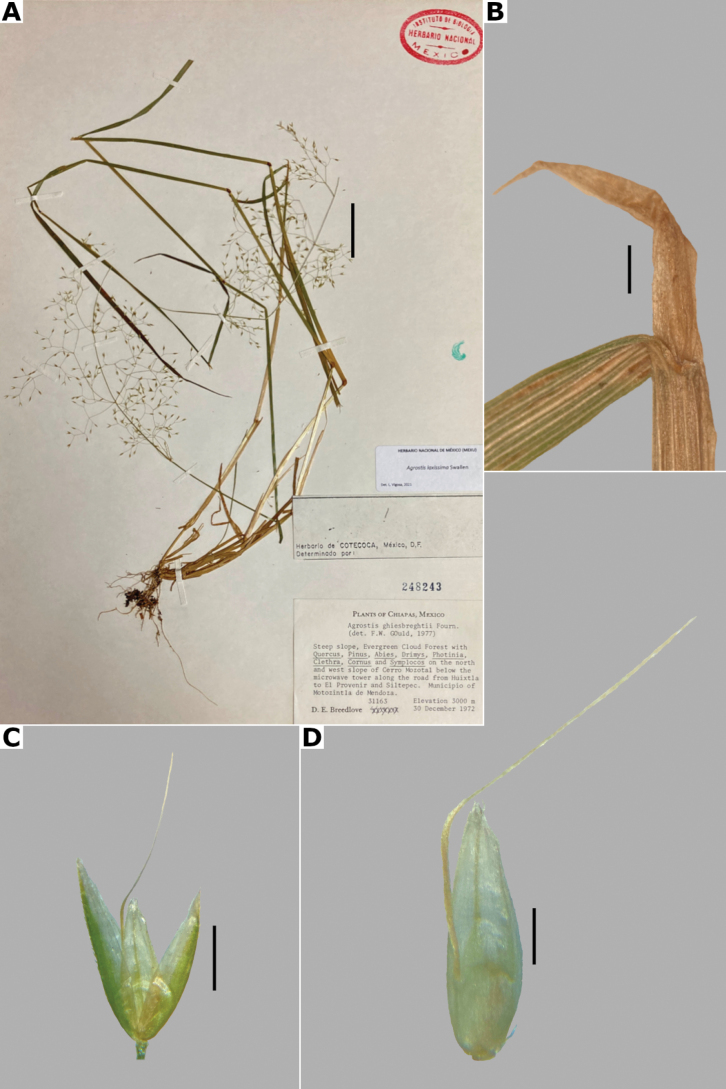
*Agrostislaxissima***A** whole plant **B** ligular area **C** spikelet **D** floret, abaxial view. Based on Breedlove 31163 (MEXU). Scale bars: 3 cm (**A**); 1 mm (**B, C**); 0.5 mm (**D**).

##### Anatomy and micromorphology.

**Figure 22.**
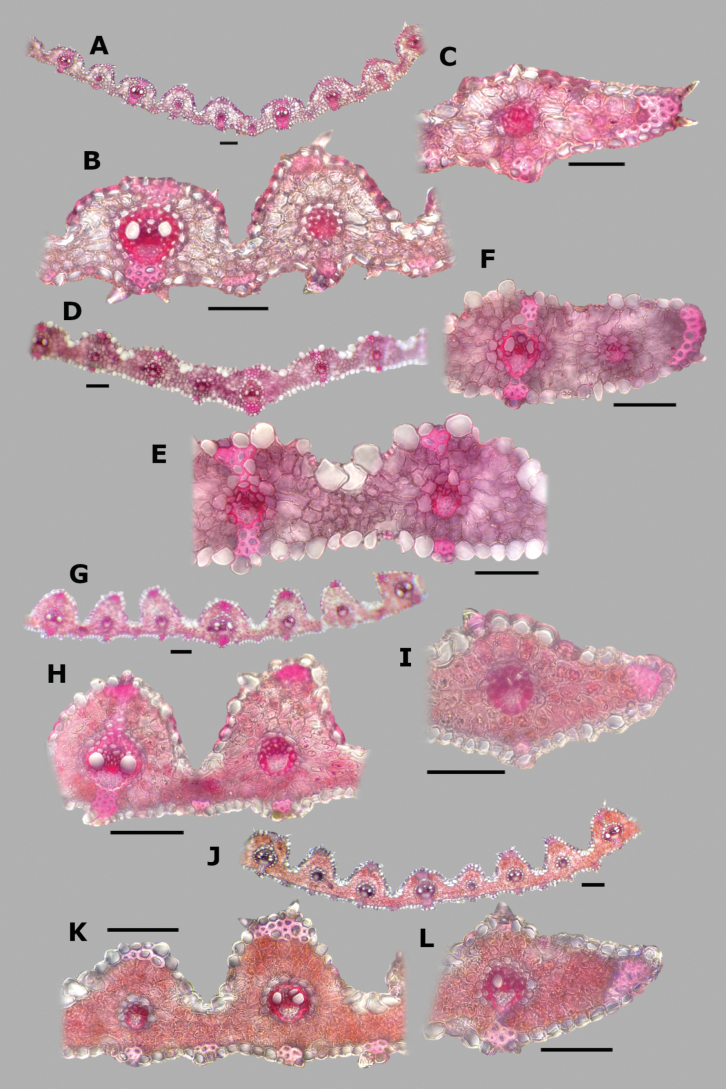
Leaf blade anatomy in transversal section of *Agrostis* species, in general view, and details of lateral bundles. **A**–**C***A.laxissima***D**–**F***A.microphylla***G**–**I***A.pallens***J**–**L***A.perennans*. Scale bars: 0.1 mm.

Leaf blades flat in transversal section; adaxial furrows medium-sized, wide; adaxial ribs rounded; keel absent; first order bundles circular in outline, sheath interrupted abaxially, abaxial sclerenchyma in girders, narrowing towards the bundle, adaxial sclerenchyma in strands; second order bundles circular in outline, sheath interrupted abaxially, abaxial sclerenchyma in girders, narrowing towards the bundle, adaxial sclerenchyma in strands; intercostal sclerenchyma present, abaxial; leaf margins with well-developed sclerenchyma caps, extending along abaxial side of the leaf; colorless cells absent (Fig. 22A–C). Lemmas with transversal thickenings irregular to oblong, wider than the unthickened portions of the wall; prickle hairs abundant (Fig. 7I).

##### Distribution and habitat.

Endemic. *Agrostislaxissima* is distributed from Southern Mexico to Guatemala (Pohl and Davidse 1994). It has also been reported in Honduras (Soreng and Peterson 2003). This species has been collected from the Mexican state of Chiapas, and the departments of Quezaltenango, Sacatepéquez, San Marcos and Solola in Guatemala (Fig. 18D). *Agrostislaxissima* grows in temperate forests, with *Abies*, *Pinus*, or *Quercus*, in cloud forests and grasslands, between 2250–3200 m a.s.l. (Fig. 9M).

##### Phenology.

Specimens with spikelets have been collected from June to March, but most of the records are from December and January (Fig. 10M).

##### Commentaries.

*Agrostislaxissima* is often confused with *A.ghiesbreghtii* (see the note under the description of that species).

##### Conservation status.

*Agrostislaxissima* is only known from a few localities of southern Mexico and Guatemala. It is represented by 17 collections, with several populations occurring in six protected areas. The EOO is 2,615 km^2^ and the AOO is 44 km^2^. Following the IUCN criteria, the preliminary assessment category is Vulnerable (VU).

##### Representative specimens examined.

**Guatemala. Quezaltenango: Municipio San Juan Ostuncalco**, barranco Buena Vista, cuesta El Caracol, Sierra Madre Mountains, about 5 km. northwest of San Juan Ostuncalco, [14.90886385°N, 91.66847343°W], 2800–2900 m alt., 11 Dec 1962, O. Williams et al. 22792 (F, US). **Municipio San Martín Sacatepéquez**, cumbre de Tuilacán, SW of San Martín Chile Verde, [14.80612°N, 91.66872°W], 2400 m alt., 8 Mar 1939, P.C. Standley 67819 (F). **Sacatepéquez: Municipio Alotenango**, Volcán de Acatenango, [14.499453°N, 90.87175°W], 3200 m alt., 11 Sep 1993, I. Arias and M. Véliz 933265a (MEXU [*]), 18 Aug 2000, M. Véliz et al. 10263 (MEXU [*, **]). **Mexico: Chiapas: Municipio Motozintla**, NW slope of cerro Mozotlan, below the microwave tower along the road from Huixtla to El Porvenir and Siltepec, [15.42605394°N, 92.34362676°W], 3000 m alt., 30 Dec 1972, D.E. Breedlove 31163 (MEXU [*, **]); 27 km al NO de Motozintla, camino a Coadesmech, torre de microondas, [15.43104735°N, 92.42724341°W], 3030 m alt., 21 Oct 1985, P. Dávila et al. 181 (MEXU [*]). See the Suppl. material 2 for additional examined specimens.

#### ﻿11. Agrostis microphylla

Taxon classificationPlantaePoalesPoaceae

Steud., Syn. Pl. Glumac. 1: 164. 1854.

55C6AA58-C2D7-50FD-8B19-C8A517CFCE9A

Figs 4J , 5J , 23

Agraulus brevifolius Nees ex Torr., Pacif. Railr. Rep. 4: 154. 1857, nom. inval., pro syn.
Agrostis virescens Kunth var. microphylla (Steud.) Scribn., Circ. Div. Agrostol. U.S.D.A. 30: 2. 1901.
Agrostis exarata Trin. var. microphylla (Steud.) Hitchc., Amer. J. Bot. 2: 303. 1915.
= Agrostis microphylla Steud. var. intermedia Beetle, Bull. Torrey Bot. Club 72(6): 547. 1945. Type. USA. California: Lake County, 2.9 miles north of Middletown, 11 May 1943, J.T. Howell 18063 (holotype: AHUC (AHUC9939 [image!]); isotype: CAS (CAS0000198 [image!])).^*^

##### Type.

America septentrionalis, D. Douglas s.n. (not located).

##### Description.

***Plants*** annual, caespitose. ***Tillers*** absent. ***Culms*** 4–25 cm long, erect, nodes 1–3, glabrous, internodes glabrous. ***Leaves*** usually mostly cauline; sheaths 1.5–8 cm long, longer or shorter than the internodes, glabrous; ligules 1–5 mm long, longer than wide, dorsally scaberulous, apices acute to truncate, erose, often lacerate; blades 1–8 cm long, 0.5–2.5 mm wide, flat, scaberulous on both surfaces. ***Panicles*** 1–9 cm long, 0.5–1.4 cm wide, contracted, dense, spiciform, linear to lanceolate, sometimes interrupted at the base, sometimes partially included in the upper sheaths; branches appressed, rebranching from below mid-length, scaberulous, with spikelets almost to the base, inferior branches 0.6–2.5 cm long; pedicels 0.3–2.5 mm long, appressed, scaberulous. ***Spikelets*** 3–4.5 mm long, greenish to stramineous; glumes unequal, lanceolate, apices long acuminate or awned, 1-veined, scaberulous on the keel, sometimes also on the body, lower glume 3–4.5 mm long including the awn, upper glume 2.5–4 mm long; callus pubescent, with two bunches of trichomes; lemmas 1.5–2 mm long, elliptic, apices 2(–4) toothed, 5-nerved, veins inconspicuous or prominent distally, awned about mid-length, awn 3.5–6 mm long, inserted 0.8–1 mm above the base, geniculate; paleas absent or up to 0.2 mm long, veinless, glabrous; anthers 3, 0.4–0.5 mm long. ***Caryopsis*** 1–1.5 mm long, elliptic; endosperm soft. 2n= 56 (Harvey 2007).

**Figure 23.**
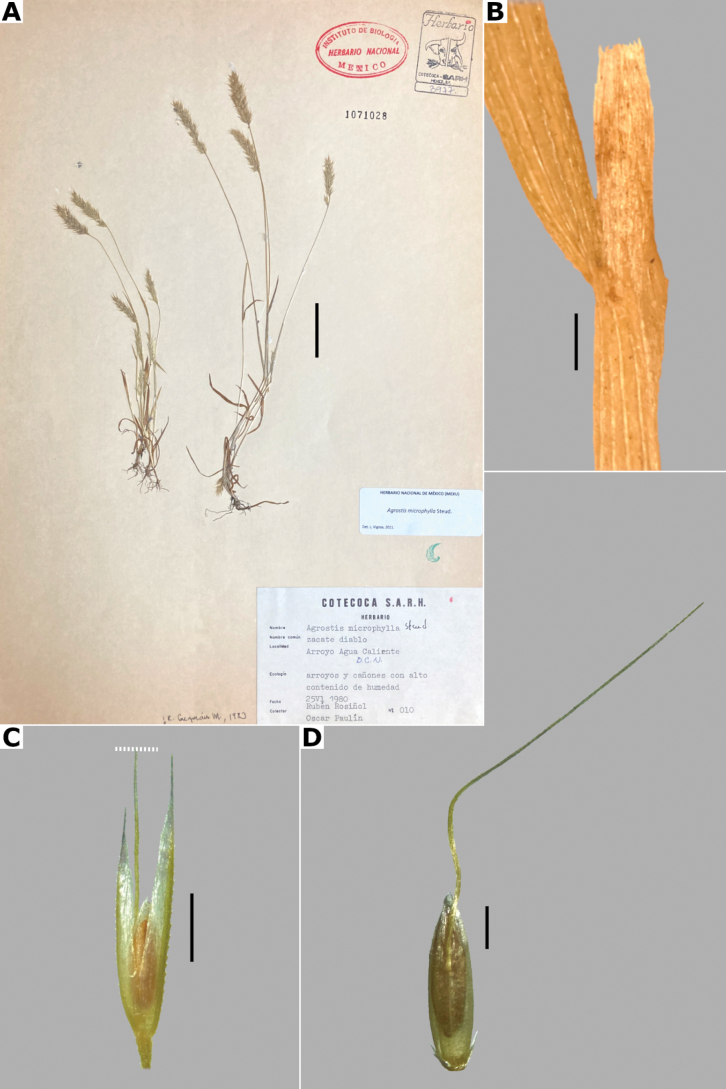
*Agrostismicrophylla***A** whole plant **B** ligular area **C** spikelet **D** floret, abaxial view. Based on Rosiñol 10 (MEXU). Scale bars: 3 cm (**A**); 1 mm (**B, C**); 0.5 mm (**D**).

##### Anatomy and micromorphology.

Leaf blades flat in transversal section; adaxial furrows shallow, wide; adaxial ribs rounded; keel absent; first order bundles circular in outline, sheath interrupted adaxially and abaxially, abaxial and adaxial sclerenchyma in girders, narrowing towards the bundle; second order bundles circular in outline, sheath interrupted abaxially, abaxial and adaxial sclerenchyma in strands; intercostal sclerenchyma absent; leaf margins with small sclerenchyma caps, rounded; colorless cells absent (Fig. 22D–F). Lemmas with transversal thickenings irregular to oblong, wider than the unthickened portions of the wall; prickle hairs scarce to abundant (Fig. 7J).

##### Distribution and habitat.

*Agrostismicrophylla* is distributed from British Columbia, Canada, to the northern peninsula of Baja California, Mexico (Harvey 2007). In the study zone, this species has been collected in the Mexican state of Baja California (Fig. 18E), where it grows in stream edges and vernal pools, between 37–315 m a.s.l. (Fig. 9N). It has also been reported from the state of Baja California Sur, but the database record linked to this report has no images (GBIF 2023c), and thus we were unable to confirm its identity.

##### Phenology.

Specimens with spikelets have been collected from April to June (Fig. 10N).

##### Commentaries.

It has been reported for other regions that the culms can reach 45 cm long, panicles up to 12 cm long, spikelets up to 5 mm, and awns up to 8 mm (Harvey 2007).

*Agrostismicrophylla* is one of the few annual *Agrostis* species. It is often confused with the smaller forms of *A.exarata*, from which it differs in the annual habit, spikelets of 3–4.5 mm long and lemmas with an awn of 3.5–6 mm long (vs. perennial plants, spikelets of 2–2.5 mm, lemmas unawned or with an awn up to 3 mm long in *A.exarata*), as well as the leaf blade anatomy and lemma micromorphology.

##### Conservation status.

*Agrostismicrophylla* is apparently a rare species in the study zone. It is represented by only four collections, with no populations occurring in protected areas. The EOO is 897 km^2^ and the AOO is 12 km^2^. Following the IUCN criteria, the preliminary assessment category is Endangered (EN).

##### Specimens examined.

**Mexico. Baja California: Municipio** Ensenada, 5 km SSO of Johnson Ranch, N of Cabo Colonet, [31.05833°N, 116.29167°W], 37 m alt., 31 May 1980, R. Moran 28447 (SD), 28643 (MEXU [*,**], SD); Guadeloupe [Guadalupe] ranch, [32.0754°N, 116.6217°W, 315 m alt.], 6 Apr 1886, C.R. Orcutt s.n. (US); arroyo Agua Caliente, [32.11163081°N, 116.4650486°W], 300 m alt., 25 June 1980, R. Rosiñol 10 (MEXU [*, **]).

#### ﻿12. Agrostis pallens

Taxon classificationPlantaePoalesPoaceae

Trin., Mém. Acad. Imp. Sci. Saint-Pétersbourg, Sér. 6, Sci. Math., Seconde Pt. Sci. Nat. 6,4(3–4): 328. 1841.

9905255E-FE66-5EF1-8248-CB79C264A622

Figs 4K , 5K , 24

= Agrostis diegoensis Vasey, Bull. Torrey Bot. Club 13: 55. 1886. Agrostismulticulmis Vasey ex Beal, Grass. N. Amer. 328. 1896, nom. inval., pro syn. Type: USA. California: San Diego, 1884, C. Orcutt s.n. (lectotype, designated by Vigosa-Mercado (2022a: 2): US (US00131740 [image!]); isolectotypes: GH (GH00022962 [image!]), K (K000838209 [image!]), NY (NY00327625 [image!]), OSC (OSC0001812 [image!]), W (W19160036561 [image!])).^*^

##### Type.

America borealis, J.D. Hooker 243 (holotype: LE-TRIN (LE-TRIN-1634.01); isotype: US [fragm. ex LE-TRIN] (US00156470 [image!])).

##### Description.

***Plants*** perennial, rhizomatous. ***Tillers*** extravaginal, with cataphylls. ***Rhizomes*** up to 10 cm long. ***Culms*** 10–70 cm long, erect or decumbent at the base, nodes 2–7, glabrous, sometimes rooting at the lower nodes, internodes glabrous. ***Leaves*** mostly cauline; sheaths 2.5–13 cm long, longer or shorter than the internodes, glabrous or scaberulous; ligules 1–5 mm long, longer than wide, dorsally scaberulous, apices acute, lacerate; blades 2.5–14 cm long, 1–4 mm wide, flat, involute when drying, scaberulous on both surfaces. ***Panicles*** (4–)5–20 cm long, 0.4–3 cm wide, contracted to open, dense to lax, lanceolate to narrow ovate, sometimes spiciform, sometimes partially included in the upper sheaths; branches appressed to ascending, rebranching from about mid-length, scaberulous, without spikelets near their base, inferior branches 1.5–3 cm long; pedicels 0.5–4 mm long, appressed to ascending, scaberulous. ***Spikelets*** 2–3.5(–4) mm long, greenish to stramineous, sometimes tinged with purple; glumes equal to subequal, lanceolate, apices acute, 1-veined, scaberulous on the keel, sometimes also on the body, lower gluma 2–3.5(–4) mm long, upper glume 1.8–3.5(–4) mm long; callus pubescent, with two bunches of trichomes, sometimes short and inconspicuous; lemmas 1.5–2.5 mm long, elliptic, apices entire, acute, or toothed, 5-nerved, veins prominent throughout or only distally, unawned, sometimes awned subapically to about mid-length, awn 0.5–1.5(–2.5) mm long, straight; paleas absent or up to 0.2 mm, veinless, glabrous; anthers 3, 0.7–1.5 mm long. ***Caryopsis*** 1–1.5 mm long, elliptic; endosperm solid. 2n= 42, 56 (Harvey 2007).

**Figure 24.**
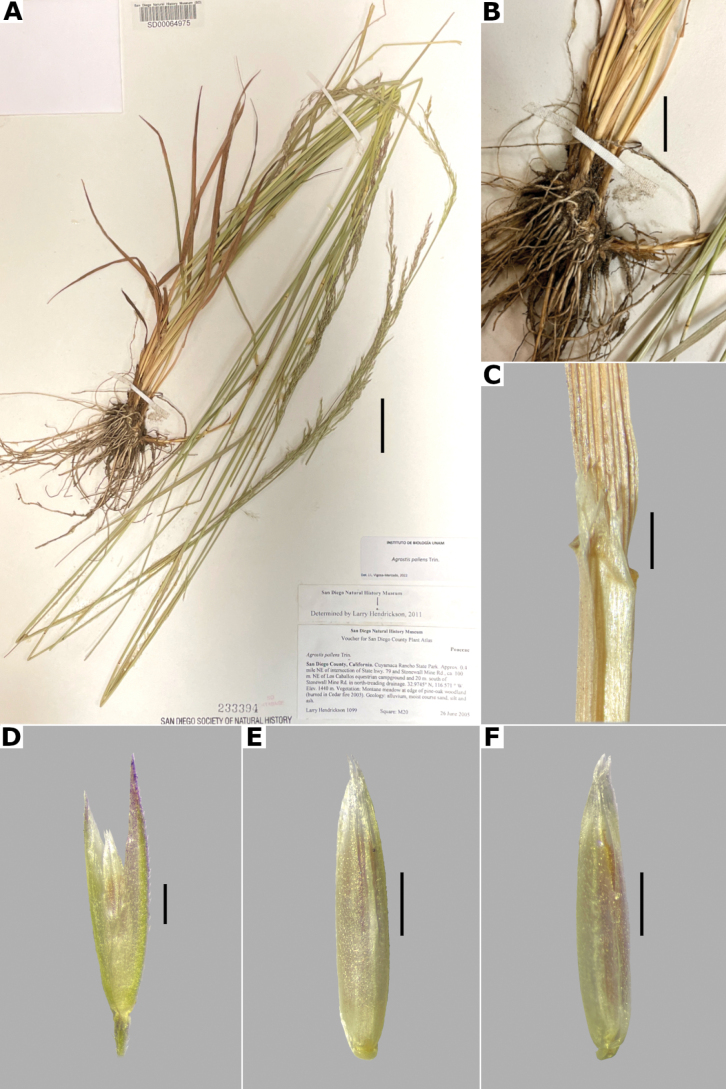
*Agrostispallens***A** whole plant **B** detail of the rhizome **C** ligular area **D** spikelet **E** floret, abaxial view **F** floret, adaxial view. Based on Hendrickson 1099 (SD). Scale bars: 3 cm (**A**); 2 cm (**B**); 2 mm (**C**); 0.5 mm (**D–F**).

##### Anatomy and micromorphology.

Leaf blades flat in transversal section; adaxial furrows deep, narrow; adaxial ribs rounded; keel absent; first order bundles circular in outline, sheath interrupted adaxially and abaxially, abaxial sclerenchyma in strands or girders, narrowing towards the bundle, adaxial sclerenchyma in strands or t-shaped girders; second order bundles circular in outline, sheath interrupted abaxially, abaxial and adaxial sclerenchyma in strands; intercostal sclerenchyma absent; leaf margins with well-developed sclerenchyma caps, rounded; colorless cells absent (Fig. 22G–I). Lemmas with transversal thickenings polygonal, wider than the unthickened portions of the wall; prickle hairs abundant (Fig. 7K).

##### Distribution and habitat.

*Agrostispallens* is distributed from British Columbia, Canada, to Baja California, Mexico, and is also present in Montana and Utah (Harvey 2007). In the study zone, this species has been collected in southern California and the Mexican state of Baja California (Fig. 18F). It grows in coastal sands, stream edges, temperate forests with *Pinus* or *Quercus*, and xeric shrublands, between 40–1635 m a.s.l. (Fig. 9O).

##### Phenology.

Specimens with spikelets have been collected from June to August (Fig. 10O).

##### Commentaries.

It has been reported for other regions that panicles can reach 6(–8) cm wide (Harvey 2007).

The Mexican populations of *A.pallens* have lemmas with longer awns, up to 2.5 mm long, than those of southern California, but the other characters are congruent with the previous descriptions of this species. The plants of lower elevations have more contracted panicles than those of higher elevations, as noted previously by Harvey (2007). Plants of lower elevations are often confused with *A.exarata*, from which they differ in the rhizomatous habit, mostly cauline leaves, and palea absent or up to 0.2 mm long (vs. usually caespitose habit, basal and cauline leaves, palea often present, up to 0.8 mm long in *A.exarata*).

##### Conservation status.

*Agrostispallens* is apparently a rare species in the study zone. It is represented by 12 collections, with several populations occurring in four protected areas. The EOO is 10,902 km^2^ and the AOO is 48 km^2^. Following the IUCN criteria, the preliminary assessment category is Vulnerable (VU).

##### Representative specimens examined.

**Mexico. Baja California: Municipio Ensenada**, S bank of arroyo Jatay, 1.5 km from the mouth, [32.01673537°N, 116.8665199°W], 40 m alt., 11 Jun 1980, R. Moran 28770 (MEXU [*,**], SD). **USA. California: Riverside County**, at Idyllwild, in San Jacinto Mountains, [33.746537°N, 116.715288°W], 1635, m alt. 14 Aug 1971, P.C. Baker 7110 (LOB). **San Diego County**, Cuyamaca Rancho State Park, 0.4 mile NE of intersection of state highway 79 and Stonewall Mine Road, ca. 100 m NE of Los Caballos equestrian campground and 20 m south of Stonewall Mine Road, in north-treading drainage, 32.9745°N, 116.571°W, 1440 m alt., 26 Jun 2005, L. Hendrickson 1099 (SD [*, **]). See Suppl. materials 2, 3 for additional examined specimens.

#### ﻿13. Agrostis perennans

Taxon classificationPlantaePoalesPoaceae

(Walter) Tuck., Amer. J. Sci. Arts 45: 44. 1843, sensu lato.

E79B5134-C7A6-581E-8083-E4C690E01C49

Figs 4L , 5L , 25

Cornucopiae perennans Walter, Fl. Carol. 74. 1788. Type: USA. South Carolina: at the intersection of cut off road and Fire Break 49 on Ft. Jackson Military Reservation, 11 Jul 1995, K.B. Kelly and J.B. Nelson 254 (neotype, designated by Ward (2007: 1099): GH (GH00247994 [image!])).
Agrostis cornucopiae Sm., Gent. Mag. 59: 873. 1789, nom. illeg. superfl.
Agrostis elegans (Walter) Salisb., Prodr. Stirp. Chap. Allerton 25. 1796.
Agrostis anomala Willd., Sp. Pl., 1(1): 370. 1797, nom. illeg. superfl.
Trichodium perennans (Walter) Elliott, Sketch Bot. S. Carolina 1(2): 99. 1816.
= Agrostis michauxii Zuccagni var. alpina Rupr., Bull. Acad. Roy. Sci. Bruxelles 52: 228. 1842, nom. nud. Type. Mexico. Oaxaca. Cordillera, 1840, H.G. Galeotii 5767 (holotype: P (P00740583 [image!]).
Agrostis scabra Willd. var. perennans (Walter) Alph. Wood, Class Book Bot. (3 ed.) 774. 1861.
= Agrostis schaffneri E. Fourn., Mexic. Pl. 2: 94. 1886. Agrostisschaffneri E. Fourn. ex Hemsl., Biol. Cent.-Amer., Bot. 3: 551. 1885, nom. nud. Type. MEXICO. Mexico City: Tacubaya, J.G. Schaffner 308 (lectotype, designated by Vigosa-Mercado (2022a: 3): P (P00740418 [image!]).
= Agrostis schaffneri E. Fourn. var. mutica E. Fourn., Mexic. Pl. 2: 94. 1886. Type. MEXICO. Mexico City: Tacubaya: J.G. Schaffner 1 (holotype: P (P00740419 [image!]).
= Agrostis tacubayensis E. Fourn., Mexic. Pl. 2: 95. 1886. Agrostistacubayensis E. Fourn. ex Hemsl., Biol. Cent.-Amer., Bot. 3: 551. 1885, nom. nud. Type. MEXICO. Mexico City: Tacubaya, J.G. Schaffner 97 (holotype: P (P00740425 [image!])).
= Agrostis chinantlae E. Fourn., Mexic. Pl. 2: 96. 1886. Agrostischinantlae E. Fourn. ex Hemsl., Biol. Cent.-Amer., Bot. 3: 550. 1885, nom nud. Type. MEXICO. Veracruz: Chinantla, May 1841, F.M. Liebmann 709 (lectotype, designated by Vigosa-Mercado (2022a: 2): C (C10016725 [image!]); isolectotypes: C (C10016726 [image!]), K (K000308372 [image!]), US (US00131733).^*^

##### Type.

Based on *Cornucopiaeperennans* Walter.

##### Description.

***Plants*** perennial, caespitose, sometimes developing short pseudostolons. ***Tillers*** extravaginal, with cataphylls. ***Culms*** 0.2–0.8(–1) m long, decumbent to erect, nodes 3–7(–10), glabrous, internodes glabrous, sometimes scaberulous. ***Leaves*** basal and cauline, the basal ones often drying at anthesis in mature individuals; sheaths 2–20 cm long, shorter or longer than the internodes, glabrous or scaberulous; ligules (0.5–)1.5–5(–7) mm long, longer than wide, dorsally scaberulous, rarely glabrous, apices acute to truncate, erose to lacerate, often ciliolate; blades 1–20 cm long, 1–6 mm wide, usually at least some blades larger than 2 mm wide, linear, usually flat, scaberulous on both surfaces. ***Panicles*** (3.5–)8–30(–40) cm long, (1–)2–20 cm wide, slightly contracted to open, dense to lax, lanceolate to ovate, long-exserted from the upper sheaths or partially included; branches ascending to spreading, rebranching slightly above mid-length, scaberulous, without spikelets near their base, inferior branches 4–12 cm long; pedicels 1–10 mm long, ascending to spreading, scaberulous. ***Spikelets*** (1.5–)1.8–3.2 mm long, greenish to purplish; glumes subequal to unequal, lanceolate, apices acute to shortly acuminate, 1-veined, scaberulous on the keel, lower glume (1.5–)1.8–3.2 mm long, upper glume 1.5–3.2 mm long; callus pubescent, with 2 bunches of trichomes; lemmas 1.3–2.2 mm long, elliptic, apices acute, entire to irregularly toothed, 5-nerved, veins inconspicuous to prominent, unawned, rarely awned from above mid-length, awn up to 1.5(–2) mm long, inserted above mid-length, straight or weakly geniculate; paleas absent or up to 0.2(–0.5) mm long, veinless, glabrous; anthers 3, 0.4–1 mm long. ***Caryopsis*** 1–1.9 mm long, elliptic: endosperm liquid to soft. 2n= 42 (Harvey 2007).

**Figure 25.**
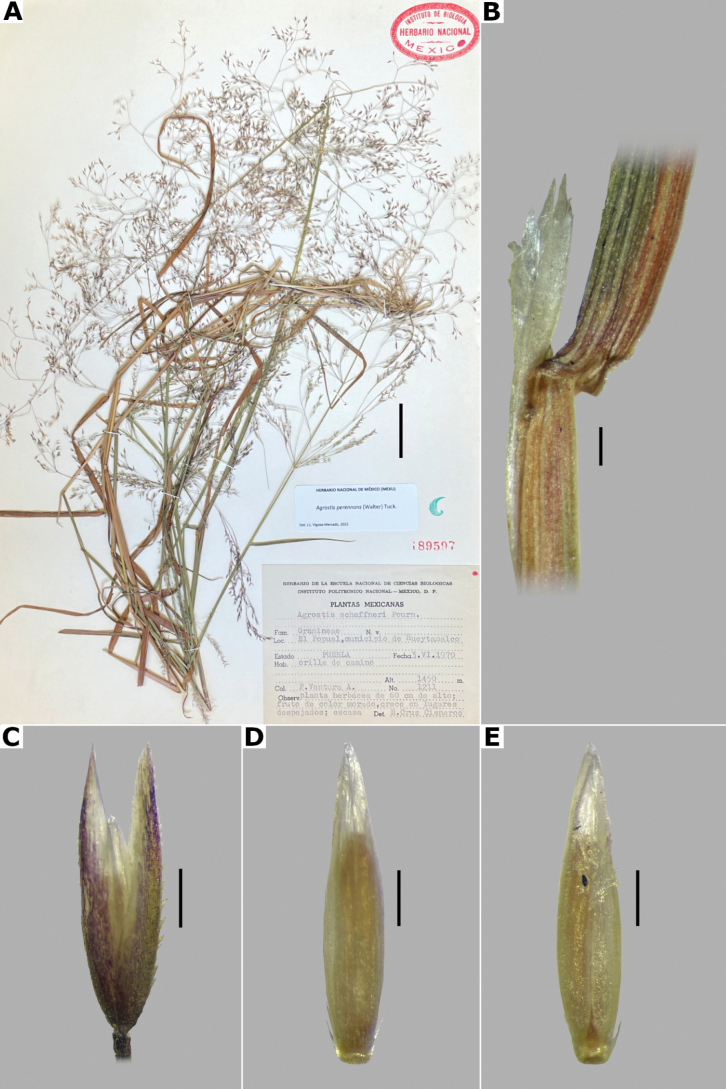
*Agrostisperennans***A** whole plant **B** ligular area **C** spikelet **E** floret, abaxial view **F** floret, adaxial view. Based on Ventura 1211 (MEXU). Scale bars: 3 cm (**A**); 5 mm (**B**); 0.5 mm (**C**); 0.3 mm (**D, E**).

##### Anatomy and micromorphology.

Leaf blades flat in transversal section; adaxial furrows shallow to deep, wide; adaxial ribs rounded; keel absent; first order bundles circular in outline, sheath interrupted abaxially, sometimes also adaxially, abaxial and adaxial sclerenchyma in strands or girders, narrowing towards the bundle; second order bundles circular in outline, sheath interrupted abaxially, abaxial and adaxial sclerenchyma in strands; intercostal sclerenchyma absent; leaf margins with well-developed sclerenchyma caps, pointed to rounded; colorless cells absent (Fig. 22J–L). Lemmas with transversal thickenings oblong, wider than the unthickened portion of the wall; prickle hairs absent, or abundant to scarce (Fig. 7L).

##### Distribution and habitat.

**Figure 26.**
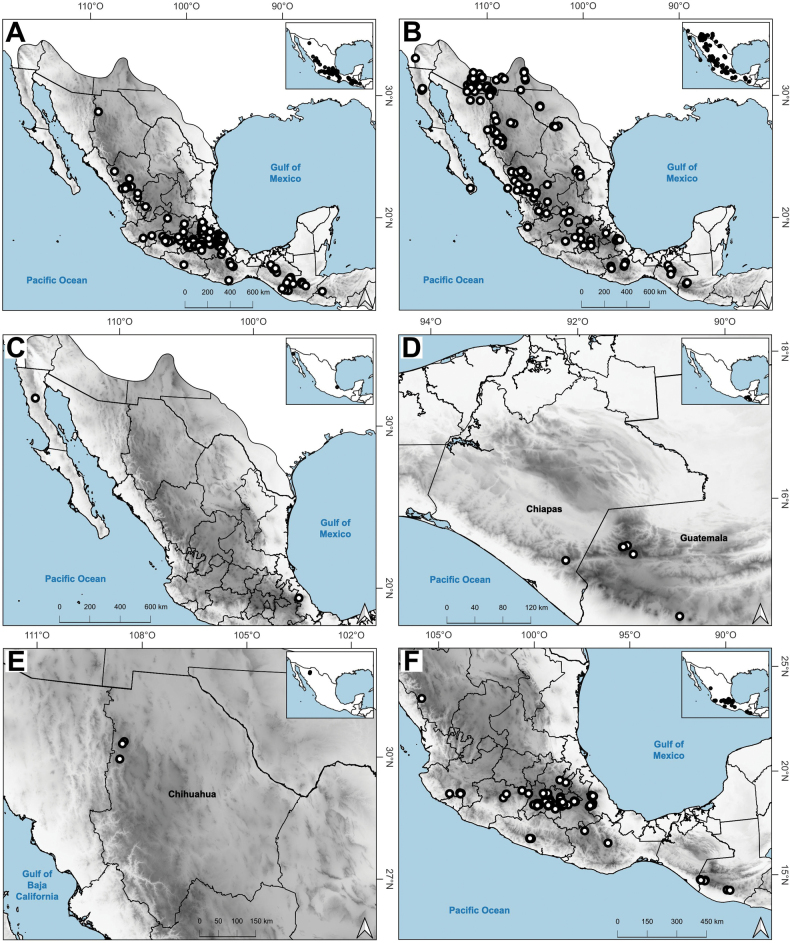
Map of known geographic distribution of *Agrostis* species, based on herbarium specimen data **A***A.perennans***B***A.scabra***C***A.stolonifera***D***A.subpatens***E***A.subrepens***F***A.tolucensis*.

**Figure 27.**
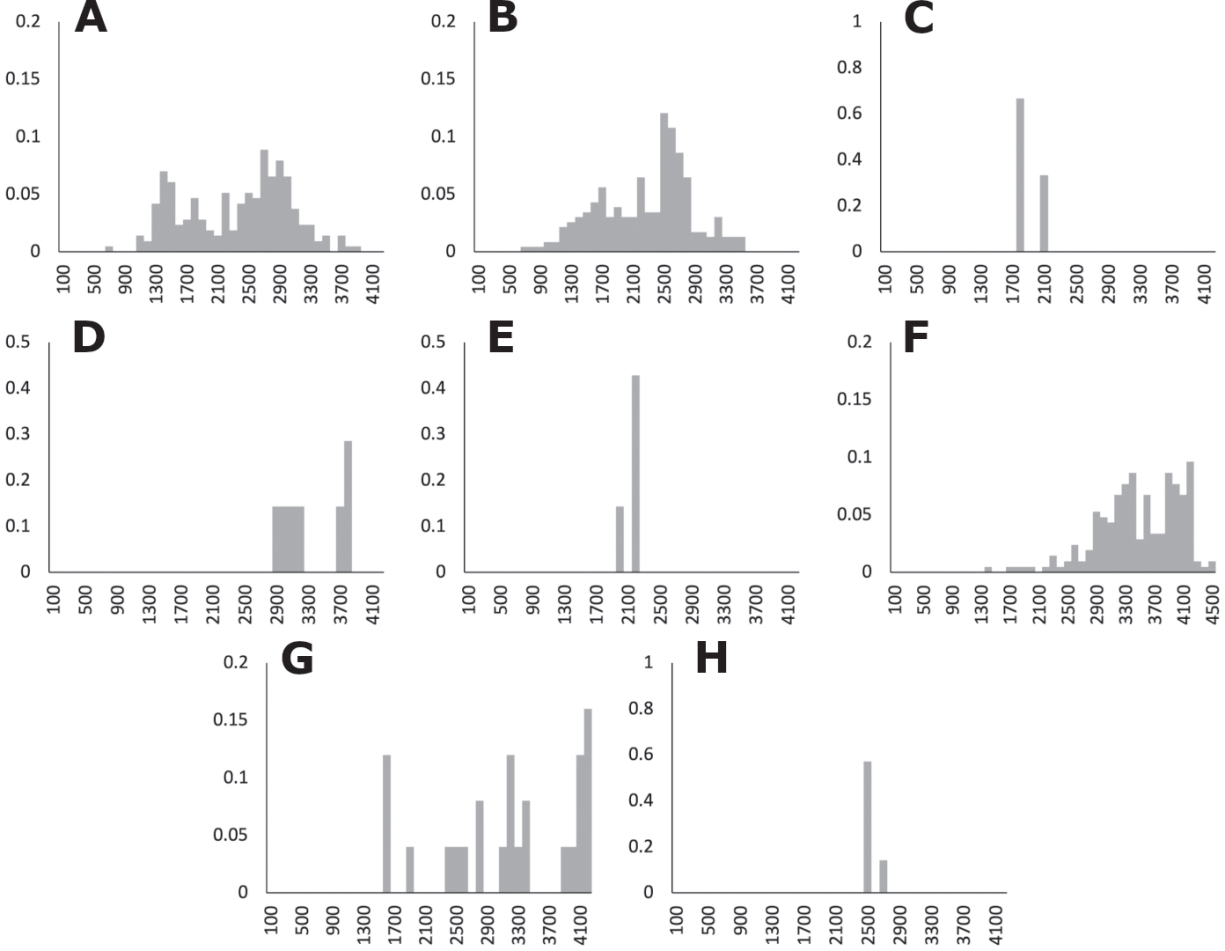
Elevation histograms of *Agrostis* species **A***A.perennans***B***A.scabra***C***A.stolonifera***D***A.subpatens***E***A.subrepens***F***A.tolucensis***G***A.turrialbae***H***A.variabilis*.

*Agrostisperennans* sensu lato is distributed from Alaska to Patagonia, in Argentina and Chile, and also in the West Indies (Sylvester et al. 2020a). In the study zone, this species has been collected in Mexico City and the Mexican states of Chiapas, Chihuahua, Durango, Guanajuato, Guerrero, Hidalgo, Jalisco, México, Michoacán, Morelos, Oaxaca, Puebla, Querétaro, Tlaxcala, Veracruz, and Zacatecas; in the Guatemalan departments of Alta Verapaz, Baja Verapaz, Huehuetenango, Quetzaltenango, Quiché and San Marcos; in the Honduran state of Ocotepeque (Fig. 26A). It has also been reported from the Mexican states of Coahuila and San Luis Potosí (Dávila et al. 2018; Sánchez-Ken 2019), but no specimen from these states have been found. Records from the southern USA, in California, Arizona, New Mexico and Texas, appear to be misidentified specimens of *A.scabra* (Harvey 2007). This species grows in open areas of temperate forests, with conifers and *Quercus*, in cloud forests, stream edges, roadsides, and often in marshy places, between 622–3847 m a.s.l. (Fig. 27A).

##### Phenology.

**Figure 28.**
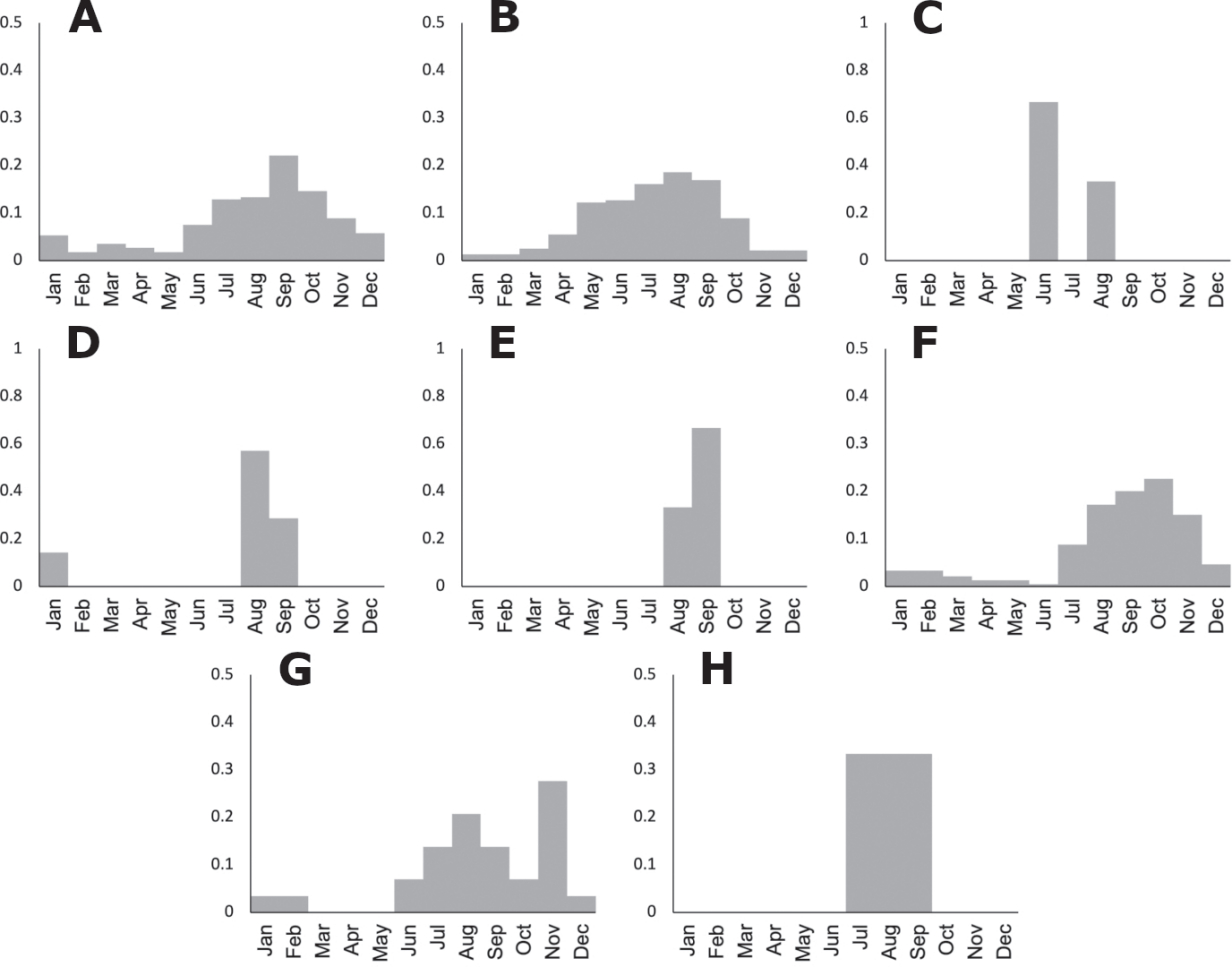
Phenology histograms of *Agrostis* species **A***A.perennans***B***A.scabra***C***A.stolonifera***D***A.subpatens***E***A.subrepens***F***A.tolucensis***G***A.turrialbae***H***A.variabilis*.

Specimens with spikelets have been collected year round, but most of them between the months of July and October (Fig. 28A).

##### Commentaries.

*Agrostisperennans* is a widespread and variable species. It is considered a “dustbin” taxon (Sylvester et al. 2020a), which includes plants with basal and cauline leaves, usually flat leaf blades, more or less open panicles, usually unawned lemmas, and absent or minute paleas. More studies are necessary to clarify the relationships of the plants that have been included under this name, thus a wide circumscription of this species is adopted in this work.

Plants examined from the study zone fit well in *A.perennans* sensu lato, but at least three forms were observed: 1) fragile plants with open panicles; 2) more robust plants with open panicles; 3) robust plants with slightly contracted, denser panicles, and sometimes awned lemmas. The plants of the latter form have been called *A.schaffneri*, but despite the form of the panicles, no differences have been found in other characters, such as leaf anatomy and lemma micromorphology thus, this name is considered a synonym of *A.perennans* sensu lato.

Young plants of *A.perennans* sensu lato often have more conspicuous basal leaves and are confused with *A.scabra*, but differ from it in the leaf blades often greater than 2 mm wide, branches rebranching slightly above mid-length, and spikelets not clustered (vs. leaf blades up to 2(–3) mm wide, branches of the panicle usually rebranching in the upper third, with the spikelets usually clustered at the tips in *A.scabra*).

*Agrostisperennans* sensu lato is often confused with several species distributed in the study zone, which share several macromorphological, leaf blade anatomy, and lemma micromorphology characters. These species are *A.bourgaei*, *A.calderoniae*, *A.ghiesbreghtii*, *A.hyemalis*, *A.laxissima*, *A.idahoensis*, *A.perennans*, *A.scabra*, *A.subrepens*, and *A.turrialbae*. In the identification key provided in this work, a reasonably good separation of the mentioned species was reached using a combination of characters.

##### Conservation status.

*Agrostisperenanns* is a common and widespread species in the study zone. It is represented by 229 collections, with several populations occurring in 18 protected areas. The EOO is 992,915 km^2^ and the AOO is 700 km^2^. Following the IUCN criteria, the preliminary assessment category is Least Concern (LC).

##### Representative specimens examined.

**Mexico. Guatemala. Alta Verapaz: Municipio Cobán**, Chicu’sha, 8 km al SO de Cobán, [15.43333333°N, 90.45°W], 1400 m alt., 22 Jul 1988, P. Tenorio 14646 (CIIDIR, MEXU [*]). **Baja Verapaz: Municipio Salamá**, 6 km al SO de Chilascó, [15.13333333°N, 90.11666667°W], 1700 m alt., 24 Jul 1988, P. Tenorio 14842 (CIIDIR, MEXU [*], US). **Honduras. Ocotepeque: Municipio Belén de Gualcho**, cordillera de Celaque, Cruz Alta, 3 mi N of Belén Gualcho long road to Cucuyagua [14.50722222°N, 88.785°W], 1890 m alt., 23 Jun 1994, G. Davidse et al. 35319 (MEXU). **Mexico. Chiapas: Municipio Amatenango del Valle**, NE slope of Zontehuitz near summit, [16.49659°N, 92.45225°W], 2130 m alt., Jan 1965, D.E. Breedlove and P.H. Raven 8119 (US). **Municipio Unión Juárez**, Volcán Tacaná, 500 m al E de Talquián, [15.09306218°N, 92.08369755°W], 1700 m alt., 26 Apr 1987, E. Martínez and A. Reyes 20291 (MEXU [*]). **Chihuahua: Municipio Casas Grandes**, near Colonia Garcia in the Sierra Madres, [29.97622543°N, 108.3352935°W], 2286 m alt., 22 Aug 1899, C.H.T. Townsend and C.M. Barber 276 (US). **Durango: Municipio Durango**, 5 mi W of Llano Grande along Durango–Mazatlán highway, [23.86°N, 105.25°W], 2500 m alt., 9 Sep 1967, J.R. Reeder and C.G. Reeder 4908 (MEXU, US). **Guanajuato: Municipio San Felipe**, club campestre El Vergel de la Sierra, [21.38504722°N, 101.6354389°W], 2627 m alt., 14 Jul 1997, J. Macías 898a (MEXU). **Guerrero: Municipio General Heliodoro Castillo**, cerro Teotepec, [17.46666667°N, 100.2166667°W], 3350 m alt., 5 Dec 1963, J. Rzedowski 18168 (ENCB). **Hidalgo: Municipio Omitlán de Juárez**, Santa Elena, km 15 de la carretera federal 105 Pachuca–Huejutla, [20.15722222°N, 98.65833333°W], 2640 m alt., 14 Jul 1994, J.P. Pérez 141 (MEXU [*]). **Municipio Tepeapulco**, cerro Santa Ana, [19.764°N, 98.5293°W], 2850 m alt., 8 Sep 1976, A. Ventura 2082 (FCME [**], MEXU, UAMIZ, XAL). **Jalisco: Municipio San Gabriel**, NW slopes of Nevado de Colima, above Jazmín, barranca near upper end of water-line 2–3 km above settlement of El Isote, [19.65°N, 103.7°W], 2600–2800 m alt., 26 Mar 1949, R. McVaugh 10048 (MEXU, US). **México: Municipio Amecameca**, km 18 de la carretera Amecameca–Tlamacas, [19.0917039°N, 98.67702965°W], 3400 m alt., 14 Jan 1982, R. Sánchez 81 (CIIDIR, IEB, MEXU). **Municipio Ocuilan**, carretera Santa Martha–Zempoala, km 2–14, [19.05°N, 99.33333333°W], 2900 m alt., 1 Aug 1987, J. Castañeda 280 (MEXU [*]). **Mexico City: Alcaldía Magdalena Contreras**, Eslava, [19.291667°N, 99.246389°W, 2530 m alt.], 19 Sep 1938, E. Lyonnet 2532 (CHAPA, MEXU, US). Los Dinamos, 2800 m alt., 27 Aug 1979, A. Ventura 3506 (FCME [*], MEXU, UAMIZ, XAL). **Alcaldía Tlalpan**, top of cerro Ajusco, [19.2069°N, 99.2597°W], 3937 m alt., 12 Jul 1959, J.H. Beaman 2794 (US). **Michoacán: Municipio Erongarícuaro**, 1 km al SE de Zínciro, sobre el camino a Eronguarícaro, [19.66463889°N, 101.7333194°W], 2400 m alt., 2 Nov 1989, J. Rzedowski 49202 (CHAPA, CIIDIR, MEXU). **Municipio Salvador Escalante**: alrededores de San Gregorio, [19.39238889°N, 101.5304389°W], 2650 m alt., 14 Sep 1988, E. Pérez-Calix 205 (CHAPA, CIIDIR, IBUG, IEB, MEXU [*,**], TEX). **Morelos: Municipio Tlalnepantla**, 2 km al S de CICITEC, Tlalnepantla, [19.06036734°N, 98.96226062°W], 2750 m alt., 26 Mar 1981, G. Ayala 19 (MEXU [*]); carretera Milpa Alta–Oaxtepec, camino viejo al CICITEC, [19.064835°N, 98.927786°W], 2770 m alt., 21 Oct 1993, A. Miranda et al. 881 (MEXU); **Oaxaca: Municipio San Juan Yaeé**, Santa María Lachichina, [17.43962564°N, 96.285451°W], 2700 m alt., 15 Apr. 2003, A. Flores s.n. (CHAPA, MEXU [*]). **Municipio San Miguel Suchixtepec**, campamento Río de Molino, 4 km al SO de San Miguel Suchixtepec, [16.07677493°N, 96.47030551°W], 2250 m alt., 21 Sep 1965, J. Rzedowski 21046 (CHAPA, IBUG, MEXU). **Puebla: Municipio Hueytamalco**, El Popual, [20.027434°N, 97.274122°W], 1450 m alt., 3 Jun 1970, F. Ventura 1211 (IBUG, MEXU). **Municipio Tlatlauquitepec**, Xucayucan, [19.89833333°N, 97.47833333°W], 1600 m alt., 5 Oct 1998, J.L. Contreras 5886 (MEXU [*,**]). **Querétaro: Municipio Colón**, parte más alta del cerro Zamorano, [20.93305556°N, 100.1797222°W], 3200–3270 m alt., 13 Nov 1971, J. Rzedowski and R. McVaugh 459 (US). **Tlaxcala: Municipio Huamantla**, ladera N del cerro de La Malinche, [19.23705301°N, 98.01935192°W], 3800 m alt., 12 Oct 1986, L. Aragón et al. 46 (MEXU). **Veracruz: Municipio Jilotepec**, El Rincón, 19.60694444°N, 96.94444444°W, 1100 m alt., 19 Jun 1993, M.J. Lizama 26 (CHAPA, CIB, MEXU [*], XAL); comunidad Jilotepec, camino La Cuesta–Zacatal, 19.61527778°N, 96.95138889°W, 1500 m alt., 12 Oct 1993, M.J. Lizama 81 (CHAPA, CIB, MEXU [*,**], XAL); comunidad El Pueblito, camino a La Concepción, 19.59722222°N, 96.925°W, 622 m alt., 17 Jan 1996, M.J. Lizama 622 (CIB). **Zacatecas**, **Municipio Monte Escobedo**, límite entre los estados de Zacatecas y Jalisco, por la terracería a Mezquitic, [22.31821648°N, 103.5748305°W], 2190 m alt., 21 Sep 1989, J. Balleza 2266b (CHAPA). See Suppl. materials 2, 3 for additional examined specimens.

#### ﻿14. Agrostis scabra

Taxon classificationPlantaePoalesPoaceae

Willd., Sp. Pl. 1(1): 370. 1797.

474A8E34-CD40-5409-A26F-232066089089

Figs 4M , 29 , 30A

= Trichodium laxiflorum Michx., Fl. Bor. Amer. 1: 42. 1803, nom. illeg. superfl. Agrostislaxa Schreb. ex Pursh, Fl. Amer. Sept. 1: 61. 1814 [1813], pro syn. Agrostislaxiflora (Michx.) Richardson, Narr. Journey Polar Sea: 731. 1823, nom. illeg. hom., non Poir., 1810. AgrostismichauxiiZuccagnivar.laxiflora A. Gray, N. Amer. Gram. 1: 17. 1834. Agrostishyemalis (Walter) Britton, Sterns & Poggenb. var. laxiflora (Michx.) Beetle, Phytologia 52: 11. 1982. Type. USA. Hab. In humidis et praetensibus a sinu Hudsonis ad Floridam, A. Michaux s.n. (holotype: P).
Trichodium scabrum (Willd.) Muhl., Cat. Pl. Amer. Sept. 10. 1813.
= Agrostis geminata Trin., Gram. Unifl. Sesquifl. 207. 1824. Agrostishyemalis (Walter) Britton, Sterns & Poggenb. var. geminata (Trin.) Hitchc., U.S.D.A. Bur. Pl. Industr. Bull. 68: 44. 1905. AgrostisscabraWilld.var.geminata (Trin.) Swallen, Proc. Biol. Soc. Washington 54: 45. 1941. AgrostisscabraWilld.var.geminata (Trin.) Hultén, Fl. Alaska Yukon 2: 156. 1942, nom. illeg. hom. Type: USA. Alaska: Unalaschka, J.F. Eschschholtz s.n. (holotype: LE-TRIN (LE01026091 [image!]); isotypes: LE-TRIN (LE00009321 [image!]), US [fragm. ex LE-TRIN] (US00156432 [image!])).
Agrostis laxiflora (Michx.) Richardson var. scabra (Willd.) Torr., Fl. New York 2: 442. 1843.
Agrostis hyemalis (Walter) Britton, Sterns & Poggenb. var. scabra (Willd.) H.L. Blomq., Grass. North Carolina 82. 1948.^*^

##### Type.

America borealis, Anonymous s.n. (lectotype, designated by Vigosa-Mercado (2022a: 3): B-W (BW01732020 [image!]); isolectotype: B-W (BW01732010 [image!])).

##### Description.

***Plants*** perennial, caespitose. ***Tillers*** extravaginal, with cataphylls. ***Culms*** 15–90 cm long, erect, nodes usually 1–2(–3), sometimes more, glabrous, internodes glabrous. ***Leaves*** mostly basal, sometimes cauline leaves well developed; sheaths 1.5–13 cm long, the lower ones usually longer than the internodes, the upper ones shorter, glabrous or scaberulous; ligules 0.5–5 mm long, usually longer than wide, dorsally scaberulous, apices rounded to truncate, sometimes acute, erose to lacerate; blades 3–14 cm long, (0.3–)0.5–2(–3) mm wide, the lower ones usually filiform and involute, the upper ones linear and flat, scaberulous on both surfaces. ***Panicles*** (4–)8–30 cm long, (2.2–)4–20(–26) cm wide, open, lax, ovate, lax, usually long-exserted from the upper sheaths; branches spreading, sometimes ascending, rebranching in the upper third, scaberulous, without spikelets near their base, spikelets usually clustered at the tips, inferior branches 1.5–13 cm long; pedicels (0.5–)1–7(–10) mm long, appressed, scaberulous. ***Spikelets*** 2–3(–3.4) mm long, greenish to purplish; glumes subequal to unequal, lanceolate, apices shortly acuminate, 1-veined, scaberulous on the keel, lower glume 1.8–2.8(–3.2) mm long, upper glume 2–3(–3.2) mm long; callus pubescent, with 2 bunches of trichomes; lemmas 1.3–2 mm long, elliptic, apices entire, obtuse, sometimes toothed, 5-nerved, veins prominent distally, unawned, rarely awned from above mid-length, awn up to 2 mm long, inserted 0.8–1.5 mm above the base, straight; paleas absent or up to 0.2 mm long, veinless, glabrous; anthers 3, 0.5–1.4 mm long. ***Caryopsis*** 0.8–1.5 mm long, elliptic; endosperm liquid to soft. 2n= 42 (Harvey 2007).

**Figure 29.**
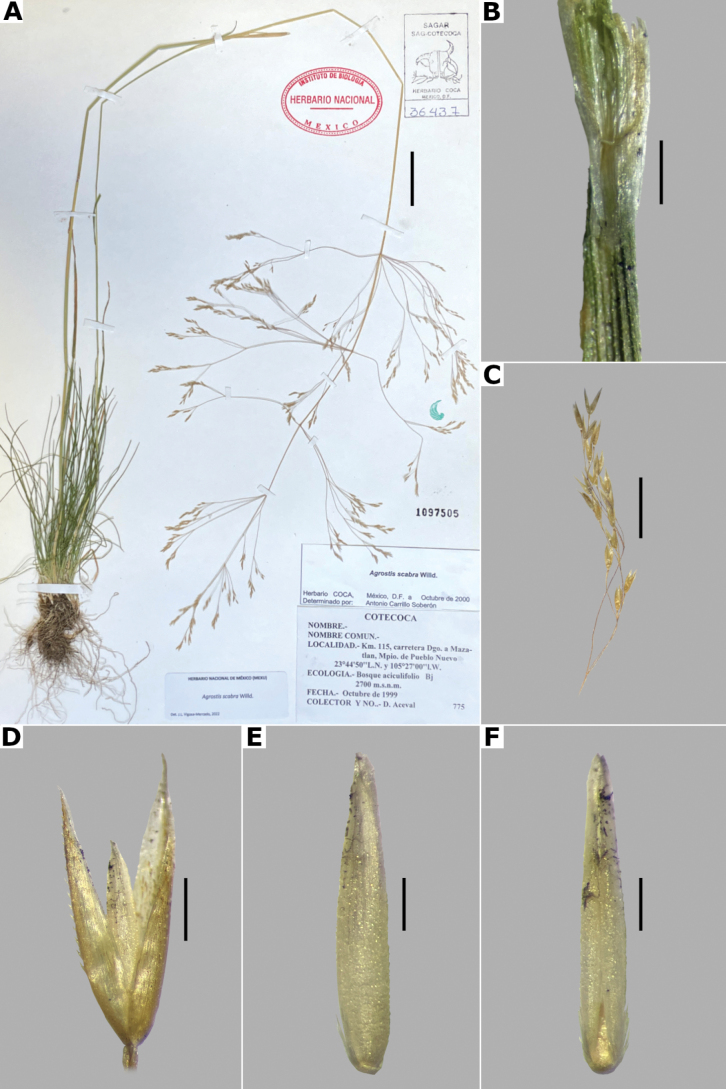
*Agrostisscabra***A** whole plant **B** ligular area **C** detail of a terminal branche of the panicle **D** spikelet, **E** floret, abaxial view, **F** floret, adaxial view. Based on Aceval 775 (MEXU). Scale bars: 3 cm (**A**); 5 mm (**B, C**); 0.5 mm (**D**); 0.3 mm (**E, F**).

**Figure 30.**
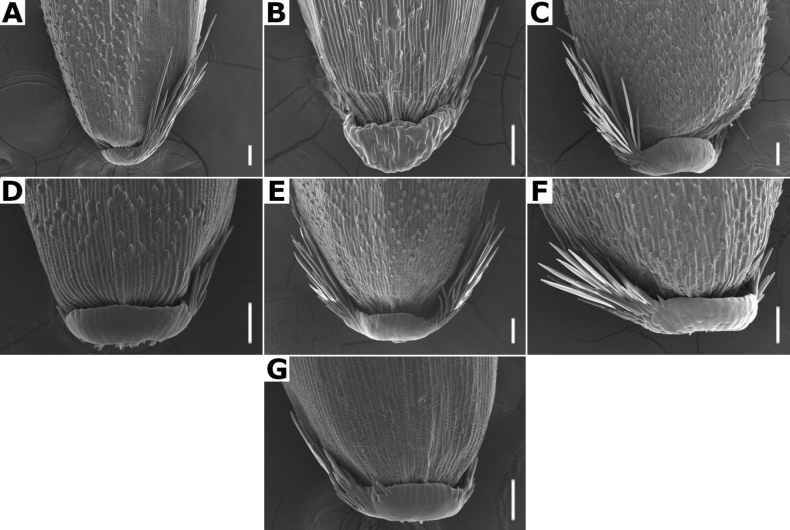
Calluses of *Agrostis* species observed with SEM **A***A.scabra***B***A.stolonifera***C***A.subpatens***D***A.subrepens***E***A.tolucensis***F***A.turrialbae***G***A.variabilis*. Scale bars: 50 μm.

##### Anatomy and micromorphology.

**Figure 31.**
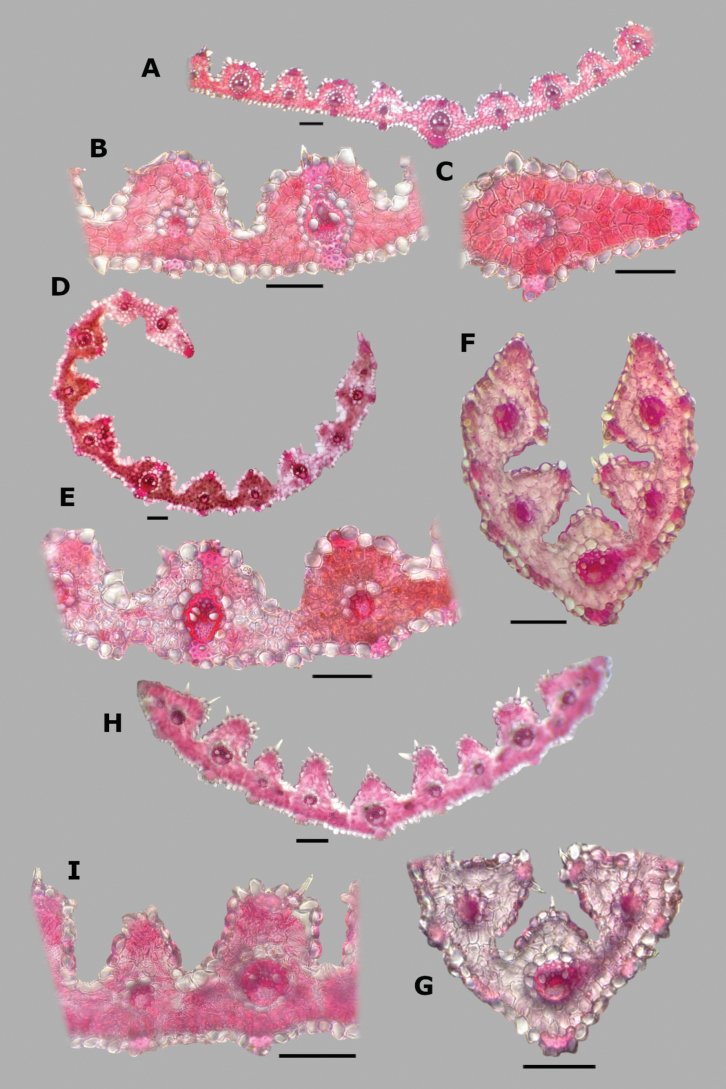
Leaf blade anatomy in transversal section of *Agrostis* species, in general view, and details of lateral bundles. **A**–**C***A.scabra***D**–**E***A.stolonifera***F**–**G***A.subpatens***H**–**I***A.subrepens*. Scale bars: 0.1 mm.

**Figure 32.**
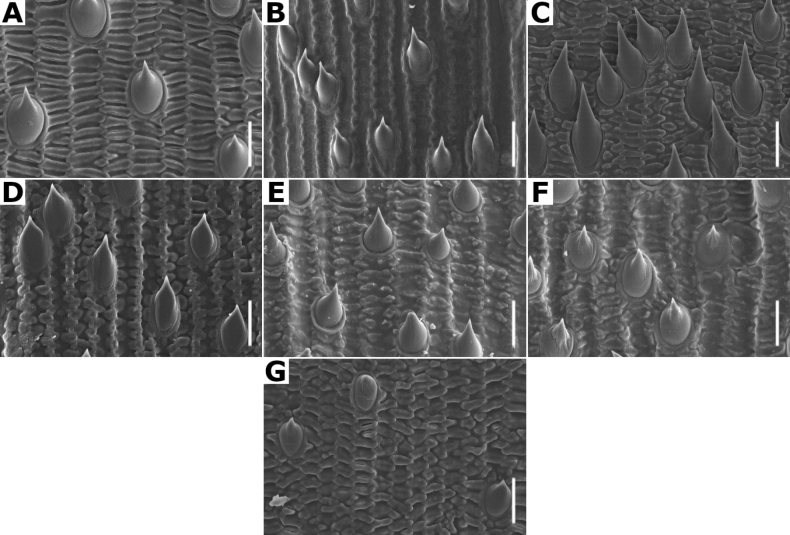
Lemma surface of *Agrostis* observed with SEM **A***A.scabra***B***A.stolonifera***C***A.subpatens***D***A.subrepens***E***A.tolucensis***F***A.turrialbae***G***A.variabilis*. Scale bars:15 μm.

Leaf blades flat in transversal section; adaxial furrows medium-sized, wide; adaxial ribs rounded; keel absent; first order bundles circular to slightly elliptical in outline, sheath interrupted adaxially and abaxially, abaxial and adaxial sclerenchyma in girders, narrowing towards the bundle; second order bundles circular in outline, sheath interrupted abaxially, abaxial and adaxial sclerenchyma in strands; intercostal sclerenchyma absent; leaf margins with well-developed sclerenchyma caps, rounded; colorless cells absent (Fig. 31A–C). Lemmas with transversal thickenings oblong, wider than the unthickened portions of the wall; prickle hairs abundant (Fig. 32A).

##### Distribution and habitat.

*Agrostisscabra* is native from Alaska to Guatemala. It has been introduced in Argentina and Chile (Rúgolo and Molina 1997), Venezuela, the West Indies and Europe (Dorr 2014). In the study zone, this species has been collected in the USA states of Arizona, California, New Mexico and Texas; in the Mexican states of Baja California, Baja California Sur, Chiapas, Chihuahua, Coahuila, Durango, Guanajuato, Jalisco, México, Michoacán, Morelos, Nuevo León, Oaxaca, Puebla, Querétaro, San Luis Potosí, Sinaloa, Sonora, Veracruz and Zacatecas; in the Guatemalan state of Huehuetenango (Fig. 26B). It has also been reported from Mexico City, and the Mexican states of Aguascalientes, Hidalgo, and Tlaxcala (Dávila et al. 2018; Sánchez-Ken 2019), but no specimens from these states were found. *Agrostisscabra* grows in open areas of temperate forests, with conifers and *Quercus*, also in cloud forests, alpine grasslands, stream edges and shrublands, between 630–3500 m a.s.l. (Fig. 27B).

##### Phenology.

Specimens with spikelets have been collected year round, but most of them between the months of April and October (Fig. 28B).

##### Commentaries.

The plants of this species are variable and at least two forms were observed in the study zone: 1) plants with very conspicuous basal leaves, with filiform and involute blades, distributed in Mexico and Guatemala; 2) plants with well-developed cauline leaves, with broader leaf blades, common in southern USA. *Agrostisscabra* is often confused with *A.perennans*, which share several leaf anatomy and lemma micromorphology characters (see the note under the description of that species). *Agrostisscabra* has been considered a synonym or a variety of *A.hyemalis* and is often confused with it (see the note under the description of that species).

##### Conservation status.

*Agrostisscabra* is a common and widespread species in the study zone. It is represented by 240 collections, with several populations occurring in 25 protected areas. The EOO is 2,076,712 km^2^ and the AOO is 712 km^2^. Following the IUCN criteria, the preliminary assessment category is Least Concern (LC).

##### Representative specimens examined.

**Guatemala. Huehuetenango: Municipio Chiantla**, Chancol, 15.4999°N, 91.34986°W, 3300 m alt., 28 Aug 2000, M. Véliz et al. 10046 (MEXU [*]). La Capellanía, 15.42111111°N, 91.44027778°W, 3150 m alt., 20 Jul 2004, M. Véliz 15309 (MEXU [*]). **Mexico. Baja California: Municipio Ensenada**, Sierra San Pedro Mártir, moist stream banks, La Víbora, arroyo La Grulla, 4 km SW of La Grulla, [30.86667°N, 115.50833°W], 1900 m alt., 9 Aug 1977, R. Moran 24402 (MEXU [*], SD). **Baja California Sur: Municipio La Paz**, arroyo Encinos Blancos, Sierra la Laguna, [23.38333333°N, 109.9833333°W], 1870 m alt., 13 Aug 1987, J.L. León de la Luz 2722 (MEXU). **Chiapas: Municipio Jitotol**, 5 km SE of Jitotol, along road to Bochil, [17.03092769°N, 92.8499077°W], 1600 m alt., 24 Feb 1982, D.E. Breedlove 58514 (MEXU, TEX). **Municipio Venustiano Carranza**, ejido Laja Tendida, km 17 carretera Venustiano Carranza–Tuxtla Gutiérrez, 2 km a Flores Magón, [16.342325°N, 92.669313°W], 630 m alt., 5 Sep 1997, A. Miranda 1275 (MEXU [*]). **Chihuahua: Municipio Ocampo**, Parque Nacional de la Cascada de Basaseachic, [28.16666667°N, 108.2083333°W], 1600 m alt., 25 Apr 1987, R. Spellenberg et al. 8428 (MEXU [*,**]); **Municipio Temosachic**, Nabogame, [28.49543744°N, 108.4808032°W], 1800 m alt., 25 Mar 1988, J.E. Laferrière 1419 (MEXU, TEX). **Coahuila: Municipio Arteaga**, El Morro, Sierra de Arteaga, límites con Nuevo León, [25.21307327°N, 100.2847136°W], 2900 m alt., 25 Sep 1991, J.A. Villareal and M.A. Carranza 6300 (CIIDIR, MEXU, XAL). **Durango**: **Municipio Pueblo Nuevo**, ejido El Brillante, lago de Puentecillas, 23.6725°N, 105.4563889°W, 2744 m alt., 28 Oct 2011, S. Heynes et al. 264 (CIIDIR, MEXU [*,**], UAMIZ). **Municipio Santiago Papasquiaro**, Bajío de Vacas (Hacienditas), [25.040833°N, 105.407778°W], 2659 m alt., 5 Oct 1990, A. Benítez 2633 (CHAPA, CIIDIR, MEXU [*], UAMIZ). **Guanajuato: Municipio Guanajuato**, Bufas de Guanajuato, [21.02596283°N, 101.2580131°W], 2140 m alt., 12 May 1981, R. Santillán and A. Mora, 28-R (MEXU). **Jalisco: Municipio Ojuelos**, Cañón de Bacieros, 14 km SO de Ojuelos, [21.81239946°N, 101.6801703°W], 2350 m alt., 4 Jun 1983, M. Alcocer s.n. (CHAPA, MEXU [*]). **México: Municipio Temascaltepec**, 11 km sobre la desviación a Tequesquipan, carretera Toluca–Temascaltepec, [19.06454704°N, 99.94451928°W], 2350 m alt., 15 Feb 1983, E. Manrique 161 (MEXU). **Michoacán: Municipio Pátzcuaro**, La Laguna, cerca de San Gregorio, [19.41886944°N, 101.4982472°W], 2700 m alt., 26 Oct 1985, J.S. Martínez 1013 (IEB). **Morelos: Municipio Huitzilac**, shore of Laguna Zempoala, 20 km NW of Cuernavaca, [19.04991306°N, 99.31567672°W], 2800 m alt., 8 Dec 1950, N.C. Fassett 28453 (F, US). **Nuevo León: Municipio Galeana**, cima del cerro Potosí, [24.87220276°N, 100.232885°W], 3500 m alt., 16 Aug 1989, A. García and S. González 196 (CIIDIR, IBUG, IEB, MEXU, UAMIZ). **Oaxaca: Municipio San Miguel El Grande**, 36 km de Tlaxiaco rumbo a Chalcatongo, [17.08138782°N, 97.61677573°W], 2559 m alt., 25 Jun 1980, A.A. Beetle M-4760 (CHAPA, IBUG, MEXU). **Puebla: Municipio Huachinango**, near Huachinango, [20.11°N, 98.03°W], 1890 m alt., 12 Apr 1962, A.A. Beetle M-567 (UTC). **Querétaro: Municipio Pinal de Amoles**: Puerto de los Velázquez, [21.12092778°N, 99.67360833°W], 2650 m alt., 17 Sep 1993, V. Jaramillo et al. 813 (IEB, MEXU). **San Luis Potosí: Municipio Villa de Arriaga**, near the village of San Francisco in the Sierra de San Miguelito ca 25 km SW of San Luis Potosi., [22N, 101.14W], 2200–2400 m alt., 5 Sep 1954, E.R. Sohns 1070 (US). **Sinaloa: Municipio Concordia**, El Palmito, en el parteaguas, 8 km al O del poblado, [23.55579174°N, 105.8468406°W], 2350 m alt., 18 Nov 1984, R. Vega 1385 (MEXU). **Sonora: Municipio Yécora**, arroyo El Otro Lado, 3.9 Km E of Yecora on Mex 16, 28.375°N, 108.898333°W, 1560 m alt., 25 May 1998, T.R. Van Devender et al. 98-640 (ASU, MEXU, NY, TEX). **Veracruz: Municipio Las Vigas de Ramírez**, Toxtlacoaya, 19.63333333°N, 97.06111111°W, 2320 m alt., 15 Sep 91, H. Sandoval 97 (CHAPA, CIB, MEXU [*]). **Zacatecas: Municipio Jerez**, Sierra Los Cardos, 10.5 mi NW of Jerez, W of El Cargadero, on road towards Monte de los Garcia, 22.7099°N, 103.131°W, 2570 m alt., 19 Oct 2007, P.M. Peterson et al. 21406 (CAN, US). **USA. Arizona: Cochise County**, Coronado National Forest, ca. 6.6 miles E on Pinery Canyon road from fork to Chiricahua National Monument, [32.00439°N, 109.243154°W], 1676 m alt., 12 Jun 1987, L.R. Landrum and S.S. Landrum 5500 (ASC, ASU). **Pima County**, Rose Canyon Lake, E end, ca. 3 km S of Mount Bigelow, [32.3833°N, 110.708°W], 2113, 17 Sep 1992, M.A. Baker 10209 (ASU [*]). **California: San Diego County**, SW of Combs Peak, canyon off of Chihuahua-Lost Valley Road just NW of Sky Oaks Field Station, on Bureau of Land Management lands, 33.38207°N, 116.62718°W, 1405 m alt., 15 Aug 2010, J.P. Rebman 20319 (SD [*]). **New Mexico: Hidalgo County**, Peloncillo Mountains, Cloverdale Creek, just inside National Forest boundary, 31.40937429°N, 108.9147098°W, 1585 m alt., 1 May 1991, K.W. Allred 5232 (IBUG). **Texas: Jeff Davis County**, Madera Canyon, roadside park in canyon along highway 118, 30.7°N, 104.1°W, 1768 m alt., 29 Jun 1979, R.D. Worthington 4691 (COLO, UTEP). See Suppl. materials 2, 3 for additional examined specimens.

#### ﻿15. Agrostis stolonifera

Taxon classificationPlantaePoalesPoaceae

L., Sp. Pl. 1: 62. 1753.

F04ABA30-1B33-5C1C-8962-D6E1EEDF7117

Figs 4N , 30B , 33

= Agrostis palustris Huds., Fl. Angl. (Hudson) 27. 1762. AgrostisstoloniferaL.var.palustris (Huds.) Farw., Rep. (Annual) Michigan Acad. Sci. 21: 351. 1920. AgrostispolymorphaHuds.var.palustris (Huds.) Huds., Fl. Angl. (ed. 2) 1: 32. 1778. AgrostisalbaL.var.palustris (Huds.) Pers., Syn. Pl. 1: 76. 1805. Aperapalustris (Huds.) Gray, Nat. Arr. Brit. Pl. 2: 148. 1821. AgrostisstoloniferaL.subsp.palustris (Huds.) Tzvelev, Novosti Sist. Vyssh. Rast. 8: 58. 1971. Type: England. 119. Gramen Miliac. maj. panic. viridi. In Herb. Petiver (lectotype, designated by Widén (1971: 77): BM).
Agrostis polymorpha Huds. var. stolonifera (L.) Huds., Fl. Angl. (ed. 2) 1: 31. 1778.
Decandolia stolonifera (L.) Bastard, Essai Fl. Maine et Loire 29. 1809.
Vilfa stolonifera (L.) P. Beauv., Ess. Agrostogr. 16. 1812.
Milium stoloniferum (L.) Lag., Elench. Pl. Nov. 10. 1816.
Agrostis alba L. var. stolonifera (L.) Sm., Engl. Fl. 1: 93. 1824.
Agrostis vulgaris With. var. stolonifera (L.) G. Mey., Chloris Han.: 657. 1836.
Agrostis vulgaris With. var. stolonifera (L.) W.D.J. Koch, Syn. Fl. Germ. Helv. 782. 1837.
Agrostis tenuis Sibth. var. stolonifera (L.) Podp., Kvetena Moravy (Prace Marav. Prir. Spolc.) 6: 354. 1926.
Agrostis palustris Huds. var. stolonifera (L.) Druce, Fl. Oxfordshire (ed. 2) 473. 1927.
Agrostis capillaris L. var. stolonifera (L.) Druce, List Brit. Pl. 126. 1928.^*^

##### Type.

Herb. A. van Royen s.n. (lectotype, designated by Widén (1971: 77): L (L0059234 [image!])).

##### Description.

***Plants*** perennial, stoloniferous. ***Tillers*** extravaginal, with cataphylls. **Stolons** up to 1(–2) m long. ***Culms*** (8–)15–60 cm long, erect, decumbent at the base, nodes (2–)3–7, glabrous, lower nodes rooting, internodes glabrous. ***Leaves*** mostly cauline; sheaths 2.5–8 cm long, usually shorter than the internodes, glabrous or scaberulous; ligules 1–7 mm long, longer than wide, dorsally scaberulous, apices rounded to truncate, erose to lacerate; blades (1–)2–10 cm long, (1–)2–6 mm wide, linear, flat, becoming convolute when dry scaberulous on both surfaces. ***Panicles*** (3–)4–20 cm long, 0.5–3 cm wide, open at anthesis, becoming contracted, dense, lanceolate, sometimes spiciform; branches appressed to ascending, branching from below mid-length, scaberulous, inferior branches 2–6 cm long, lateral branches often with spikelets near their base; pedicels 0.5–3.3 mm long, appressed to ascending, scaberulous. ***Spikelets*** 1.6–2 (–3) mm long, greenish, often tinged with purple; glumes subequal to unequal, lanceolate, apices acute to shortly acuminate, 1-veined, scaberulous on the keel, lower glume 1.6–2 (–3) mm long, upper glume 1.4–2(1.6–2 (–3) mm long; callus puberulous, with 2 bunches of short trichomes, sometimes inconspicuous; lemmas 1.4–2 mm long, elliptic to oblong, apices entire, acute to obtuse, sometimes toothed, 3(5)-veined, veins inconspicuous or prominent distally, unawned, rarely awned from above mid-length, awn up to 1.5 mm long, inserted 0.8–1.5 mm above the base, straight or weakly geniculate; paleas present, 0.7–1.4 mm long, 2-veined, glabrous; anthers 3, 0.9–1.5 mm long. ***Caryopsis*** 0.9–1.3, elliptic; endosperm solid. 2n= 28, 35, 42 (Harvey 2007).

**Figure 33.**
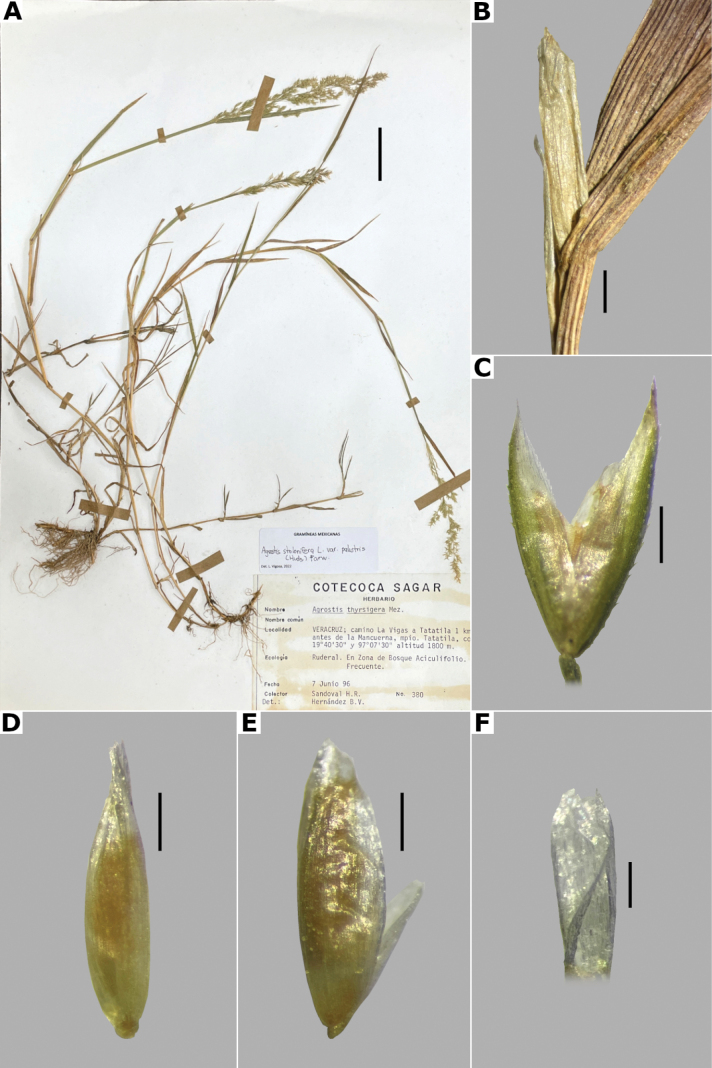
*Agrostisstolonifera***A** whole plant **B** ligular area **C** spikelet **D** floret, abaxial view, **E** floret, lateral view showing the lemma and the palea **F** palea. Based on Sandoval and Hernández 380 (CIB). Scale bars: 3 cm (**A**); 1 mm (**B**); 0.5 mm (**C**); 0.3 mm (**D, E**); 0.2 mm (**F**).

##### Anatomy and micromorphology.

Leaf blades flat to convolute in transversal section; adaxial furrows medium-sized to deep, wide; adaxial ribs rounded; keel absent; first order bundles circular to slightly elliptical in outline, sheath interrupted adaxially and abaxially, abaxial and adaxial sclerenchyma in girders, narrowing towards the bundle; second order bundles circular in outline, sheath interrupted abaxially, abaxial and adaxial sclerenchyma in strands; intercostal sclerenchyma absent; leaf margins with well-developed sclerenchyma caps, rounded; colorless cells absent (Fig. 31D, E). Lemmas without transversal thickenings, prickle hairs abundant to scarce (Fig. 32B).

##### Distribution and habitat.

Introduced. *Agrostisstolonifera* is native to Eurasia and northern North America (Harvey 2007). In the study zone, this taxon has been collected from the Mexican states of Baja California and Veracruz (Fig. 26C). *Agrostis* has also been reported from the southern United States and Mexico City, also from the states of Chiapas, Chihuahua, Coahuila, Hidalgo, Jalisco, México, Michoacán, Nuevo León, Puebla, and Tlaxcala (Dávila et al. 2018; Sánchez-Ken 2019), but no specimens from these states have been seen. This taxon has been collected on stream edges and open areas of pine forests, between 1800–2100 m a.s.l. (Fig. 27C). There are more records of this species in the study zone, on databases (GBIF 2023d), but not all of them have images, and thus we were unable to confirm their identity.

##### Phenology.

Specimens with spikelets have been collected from June to August (Fig. 28C).

##### Commentaries.

*Agrostisstolonifera* is often confused with *A.gigantea* (see the note under the description of that species). This is a variable species and several infraspecific taxa have been recognized (e.g., Pohl and Davidse 1994; Rúgolo and Molina 1997). The plants from the study zone fit well in A.stoloniferavar.palustris, which is distinguished from the typical variety in the more contracted panicles, up to 3 cm wide, and smaller spikelets of 1.6–2 mm long (vs. more open panicles, up to 6 cm wide, spikelets 2–2.5 mm long in the typical variety). The width of the panicles could be related to the age of the panicles, since they become contracted after anthesis, and thus varieties are not recognized here.

This taxon is also confused with *Polypogonviridis* (Gouan) Breistr., from which it is distinguished in the spikelets disarticulating above the glumes (vs. disarticulation below the glumes, with a pedicel fragment in *P.viridis*).

##### Conservation status.

Since *Agrostisstolonifera* is an introduced taxon in the study zone, its conservation status is considered as Least Concern (LC).

##### Specimens examined.

**Mexico. Baja California: Municipio Ensenada**, Sierra San Pedro Mártir, La Grulla, [30.88916°N, 115.46223°W], 2100 m alt., 21 Aug 1967, R. Moran and R.F. Thorne 14466 (SD). **Veracruz: Municipio Tatatila**, camino Las Vigas–Tatatila, 1 km antes de La Mancuerna, 19.675°N, 97.125°W, 1800 m alt., 7 Jun 1996, H.R. Sandoval and B.V. Hernández 370 (CIB), 380 (CIB, MEXU [*, **], XAL).

#### ﻿16. Agrostis subpatens

Taxon classificationPlantaePoalesPoaceae

Hitchc., in Britton, N. Amer. Fl. 17(7): 527. 1937.

E90CA428-F83D-57D4-BCED-354355691E63

Figs 4O , 30C , 34

= Agrostis vinosa Swallen, Contr. U.S. Natl. Herb. 29(9): 402. 1950. Type. Guatemala. Huehuetenango: alpine meadow, vicinity of Chémal, summit of Sierra de los Cuchumatanes, 3700–3750 m alt., 8 Aug 1942, J. Steyermak 50290 (holotype: US (US00131130); isotypes: F (F0046567F [image!]), MO (MO-501391 [image!]), US (US00624107 [image!])).

##### Type.

Costa Rica. Cerro de la Muerte, 3100 m alt., Jan 1897, H. Pittier 10470 (holotype: US (US00131113); isotype: G (G00192032 [image!])).

##### Description.

***Plants*** perennial, caespitose. ***Tillers*** extravaginal, with cataphylls. ***Culms*** up to 30 cm long, erect, decumbent at the base, nodes 1–2, glabrous, internodes glabrous. ***Leaves*** mostly basal; sheaths 1–6 cm long, longer or shorter than the internodes, glabrous or scaberulous; ligules 2–3(5) mm long, longer than wide, dorsally scaberulous, apices acute, often lacerate; blades 2.2–10(15) cm long, 0.4–0.8 mm wide, filiform, conduplicate to convolute, sometimes flat at the base, scaberulous on both surfaces. ***Panicles*** 5–11.5 cm long, 0.5–4 cm wide, contracted to open, somewhat lax, linear to lanceolate, exserted from the upper sheaths; branches appressed to ascending, rebranching about mid-length or below, scaberulous, without spikelets near their base, inferior branches up to 2.8 cm long cm long; pedicels 2–7 mm long, usually longer than the spikelets, appressed to ascending, scaberulous. ***Spikelets*** 2.1–3 mm long, purplish; glumes subequal to unequal, lanceolate, apices acute to shortly acuminate, 1-veined, scaberulous on the keel, lower glume 2.1–3 mm long, upper glume 1.9–2.8 mm long; callus pubescent, with 2 bunches of trichomes; lemmas 1.3–2.2 mm long, elliptic, apices toothed, 5-veined, veins prominent distally, awned near the base, awn 3–3.5 mm long, weakly geniculate, reaching the lemma apices; paleas absent; anthers 3, 0.9–1.5 mm long. ***Caryopsis*** 0.8–1.5 mm long, elliptic; endosperm solid. 2n= 28 (Pohl and Davidse 1971).

**Figure 34.**
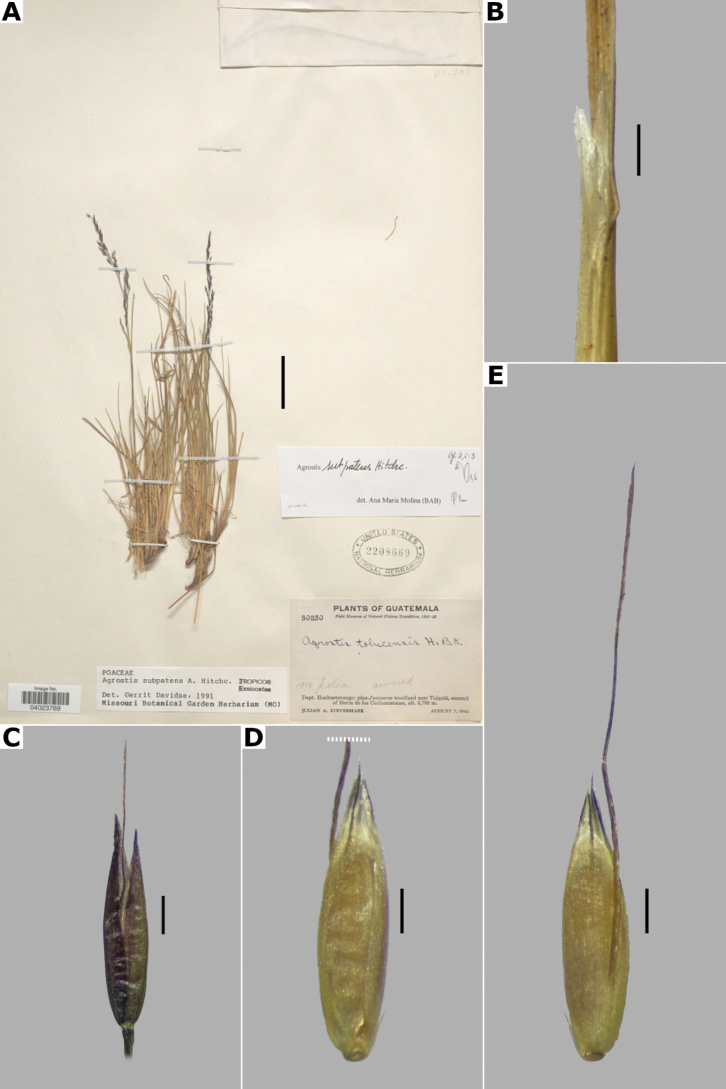
*Agrostissubpatens***A** whole plant **B** ligular area **C** spikelet **D** floret, lateral view, **E** floret, abaxial view. A based on Steyermark 50230 (US), B–E based on Breedlove 40355 (MEXU). Scale bars: 3 cm (**A**); 1 mm (**B**); 0.5 mm (**C**); 0.3 mm (**D, E**).

##### Anatomy and micromorphology.

Leaf blades convolute to v-shaped in transversal section; adaxial furrows deep, narrow; adaxial ribs rounded to triangular; keel absent; first order bundles circular in outline, sheath not interrupted, abaxial and adaxial sclerenchyma in strands; second order bundles circular in outline, sheath not interrupted, abaxial and adaxial sclerenchyma in strands; intercostal sclerenchyma present, abaxial; leaf margins with well-developed sclerenchyma caps, rounded; colorless cells absent (Fig. 31F, G). Lemmas with transversal thickenings irregular to oblong, wider than the unthickened portion of the wall; prickle hairs abundant (Fig. 32C).

##### Distribution and habitat.

*Agrostissubpatens* is distributed from Chiapas, Mexico to Costa Rica (Pohl and Davidse 1994). It has also been reported from Venezuela (Luteyn 1999). In the study zone, it has been collected in the Mexican state of Chiapas and in the Guatemalan departments of Chimaltenango and Huehuetenango (Fig. 26D). This species has also been reported from Mexico City and the Mexican states of Hidalgo, Jalisco, México, Michoacán, Oaxaca, Puebla, Querétaro, Tlaxcala and Veracruz (Villaseñor 2016; Dávila et al. 2018; Sánchez-Ken 2019; Vigosa-Mercado and Ruiz-Sánchez, 2020), but these records correspond to misidentified specimens of *A.tolucensis* and *A.turrialbae*. *Agrostissubpatens* grows in open areas of temperate forests with *Pinus* and *Juniperus*, and in alpine grasslands, between 2900–3790 m a.s.l. (Fig. 27D).

##### Phenology.

Specimens with spikelets have been collected in January, August and September (Fig. 28D).

##### Commentaries.

This species is similar to *A.tolucensis* and *A.turrialbae*, with which it shares the basal filiform leaves, as well as several leaf blade anatomy and lemma micromorphology characters. *Agrostissubpatens* differs from *A.tolucensis* in the less dense and often more open panicles, with pedicels usually longer than the spikelets (vs. usually dense and spiciform panicles, pedicels usually shorter than the spikelets in *A.tolucensis*). It differs from *A.turrialbae* in the awned lemmas and absent palea (vs. unawned lemmas, palea up to 0.2 mm long in *A.turrialbae*).

##### Conservation status.

*Agrostissubpatens* is known in the study zone from a few localities in southern Mexico and Guatemala. It is represented by seven collections, with several populations occurring in two protected areas. The EOO is 5,589 km^2^ and the AOO is 20 km^2^. Following the IUCN criteria, the preliminary assessment category is Endangered (EN).

##### Specimens examined.

**Guatemala. Chimaltenango: Municipio Acatenango**, slopes of Volcán de Acatenango, above Las Calderas, [14.52463248°N, 90.87729338°W], 2900 m alt., 3 Jun 1939, P.C. Standley 61878 (F). **Huehuetenango: Municipio Chiantla**, Llano de Tsajualá, 3170 m alt., 26 Aug 1976, D.N. Smith 383 (F); cerca del cementerio, aldea San Nicolás, [15.43172006°N, 91.43878244°W], 3090 m alt., 3 Sep 1976, D.N. Smith 430 (F). **Municipio Todos Santos Cuchumatán**, Cerro Alto entre Llano de San Miguel y Todos Santos Cuchumatán, [15.54382199°N, 91.57855143°W], 3790 m alt., 29 Aug 1976, D.N. Smith 411 (F); near Tojquiá, summit of Sierra de los Cuchumatanes, [15.54360741°N, 91.56627528°W], 3700 m alt., 7 Aug 1942, J.A. Steyermark 50230 (F, US). **Mexico. Chiapas: Municipio Siltepec**, on the N and W slope of cerro Mozotal below the microwave tower along the road from Huixtla to El Porvenir and Siltepec, [15.4275°N, 92.341944°W], 3000 m alt., 19 Sep 1976, D.E. Breedlove 40355 (DS, MEXU [*, **]).

#### ﻿17. Agrostis subrepens

Taxon classificationPlantaePoalesPoaceae

(Hitchc.) Hitchc., in Britton, N. Amer. Fl. 17(7): 525. 1937.

948A2A63-7B9E-5E8E-800D-50278004588E

Figs 4P , 30D , 35

Agrostis hyemalis (Walter) Britton, Sterns & Poggenb. var. subrepens Hitchc., U.S.D.A. Bur. Pl. Industr. Bull. 68: 44. 1905. Type: Mexico. Chihuahua: in wet places, pine plains, base of Sierra Madre Mountains, 28 Sep 1887, C.G. Pringle 1420 (holotype: US (US00131756 [image!]); isotypes: F (F-104784 [image!], F-2108718 [image!]), K (K000308371 [image!]), NY (NY-327645 [image!], NY-327646 [image!], US (US00131757 [image!])).

##### Type.

Based on *Agrostishyemalis* (Walter) Britton, Sterns & Poggenb. var. subrepens Hitchc.

##### Description.

***Plants*** perennial, rhizomatous or developing pseudostolons. ***Tillers*** extravaginal, with cataphylls. ***Rhizomes and pseudostolons*** up to 5 cm long. ***Culms*** 0.6–1 m long, erect, decumbent at the base, nodes 2–4, glabrous, internodes glabrous. ***Leaves*** mostly cauline; sheaths 3.5–7 cm long, usually shorter than the internodes, glabrous; ligules 1–2 mm long, longer than wide, dorsally scaberulous, apices truncate, erose; blades 3–5 cm long, 1–1.5 mm wide, linear, flat or involute, scaberulous on both surfaces. ***Panicles*** 9–22 cm long, 5–10 cm wide, open, lax, pyramidal, long-exserted from the upper sheaths; branches spreading, rebranching about mid-length, scaberulous, without spikelets near their base, inferior branches 2–8 cm long; pedicels 1–3 mm long, ascending to spreading, scaberulous. ***Spikelets*** 1.8–2.8 mm long, purplish; glumes subequal, lanceolate, apices acute, 1-veined, scaberulous on the keel, lower glume 1.8–2.8 mm long, upper glume 1.7–2.7 mm long; callus glabrous or with a few trichomes, Inconspicuous; lemmas 1.3–2 mm long, elliptic, apices entire, acute, 5-veined, veins prominent, unawned; paleas absent; anthers 3, 1–1.3 mm long. ***Caryopsis*** ca. 1.2 mm long, elliptic; endosperm solid. 2n= unknown.

**Figure 35.**
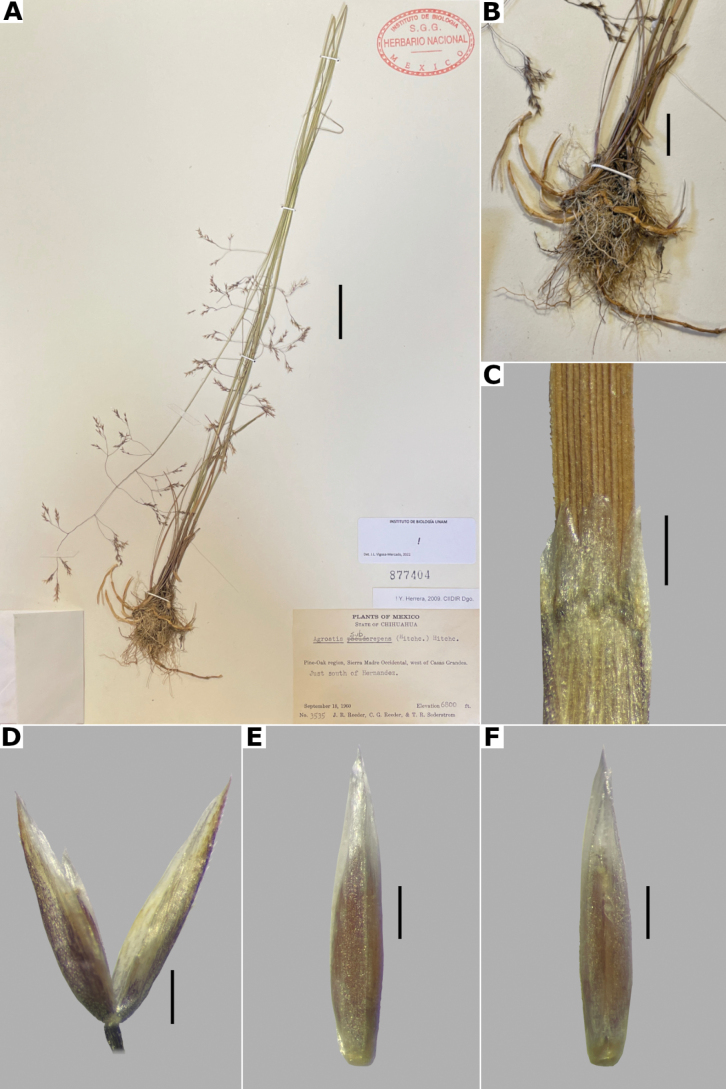
*Agrostissubrepens***A** whole plant **B** detail of the pseudostolons **C** ligular area **D** spikelet **E** floret, abaxial view **F** floret, adaxial view. Based on Reeder et al. 3535 (MEXU). Scale bars: 3 cm (**A**); 1 cm (**B**); 1 mm (**C**); 0.5 mm (**D**); 0.3 mm (**E, F**).

##### Anatomy and micromorphology.

Leaf blades flat in transversal section; adaxial furrows deep, narrow; adaxial ribs square to triangular; keel absent; first order bundles circular in outline, sheath not interrupted, abaxial and adaxial sclerenchyma in strands; second order bundles circular in outline, sheath not interrupted, abaxial and adaxial sclerenchyma in strands; intercostal sclerenchyma absent; leaf margins with well-developed sclerenchyma caps, rounded; colorless cells absent (Fig. 31H, I). Lemmas with transversal thickenings irregular, wider than the unthickened portion of the wall; prickle hairs abundant (Fig. 32D).

##### Distribution and habitat.

*Agrostissubrepens* was described from the Sierra Madre Occidental, in Chihuahua, Mexico (Fig. 26E). It has also been reported from South America, in Bolivia, Ecuador, Colombia, Paraguay, Perú and Venezuela (Soreng and Peterson 2003; Idárraga-Piedrahita et al. 2011), but the specimens from these countries were not seen, and could represent misidentifications of other species. In the study zone, *A.subrepens* grows in wet areas, in forests with *Pinus* and *Quercus*, between 2000–2168 m in elevation (Fig. 27E).

##### Phenology.

Specimens with spikelets have been collected from August to September (Fig. 28E).

##### Commentaries.

The status of *A.subrepens* as a distinct species and its distribution has been put in doubt recently (Sylvester et al. 2020a). This species is very similar to *A.perennans* sensu lato and other awnless species of the study zone, but differs from them in the presence of pseudostolons, leaf blades with square to triangular adaxial ribs, and lemmas with irregular thickenings (vs. caespitose plants, leaf blades with rounded adaxial ribs, lemmas with usually polygonal thickenings). Some individuals of *A.perennans* sensu lato sometimes develop pseudostolons, but despite the leaf anatomy, there are few differences between the two taxa. We recognise *A.subrepens* as a distinct species, until more evidence is available.

It could also be confused with *A.pallens* from California and Baja California, but it is distinguished in the more open panicles, and unawned lemmas (vs. panicles often contracted, often awned lemmas), as well as the leaf anatomy.

##### Conservation status.

*Agrostissubrepens* is known in the study zone from a few localities in Chihuahua, Mexico. It is represented by five collections, with no populations occurring in protected areas. The EOO is 74 km^2^ and the AOO is 12 km^2^. Following the IUCN criteria, the preliminary assessment category is Endangered (EN).

##### Specimens examined.

**Mexico.** Chihuahua: Municipio Casas Grandes, Sierra Madre Occidental, W of Casas Grandes, just S of Hernández [30.04186415°N, 108.2901759°W], 2000 m alt., 18 Sep 1960, J. Reeder et al. 3535 (MEXU [*, **], US). **Municipio Madera**, Chuhuichupa, [29.60543842°N, 108.3736947°W, 2168 m alt.], Aug–Sep 1936, H. LeSueur 87 (US), 198 (US), near Colonia Garcia, in the Sierra Madre, [29.9833°N, 108.333°W, 2149 m alt.], 1 Aug 1899, E.W. Nelson 6195 (US).

#### ﻿18. Agrostis tolucensis

Taxon classificationPlantaePoalesPoaceae

Kunth, in Humb., Bonpl. & Kunth, Nov. Gen. Sp. 1: 135. 1816.

435EF884-60DE-5387-94D0-DDE29B78E1F4

Figs 4Q , 30E , 36

= Agrostis virescens Kunth, in Humb., Bonpl. & Kunth, Nov. Gen. Sp. 1: 135–136. 1816. Agrostistolucensis Willd. ex Steud., Syn. Pl. Glumac. 1: 164. 1854, nom. inval., pro syn. Type: Mexico. State of México: in planitie Tolucana, A. Humboldt and A. Bonpland s.n. (holotype: P (P00669395 [image!]); isotypes: LE-TRIN, P (P00136912 [image!], P00740428 [image!], P00740429 [image!], P00740430 [image!])).^*^

##### Type.

Mexico. State of México: crescit in apricis, aridis regni Mexicani, prope urbem Toluca et Islahuaca [Ixtlahuaca], 1380 hexap. alt., A. Humboldt and A. Bonpland s.n. (holotype: P (P00669394 [image!]); isotypes: P (P00136913 [image!]), P00136914 [image!], P00136915 [image!], P00740426! [image!]), US [fragm. ex P] (US00156505 [image!]).

##### Description.

***Plants*** perennial, caespitose, or shortly rhizomatous. ***Tillers*** extravaginal and intravaginal, with cataphylls. ***Rhizomes*** if present, up to 1 cm, ascendent. ***Culms*** 5–30(–60) cm long, erect, nodes 1–3, glabrous, internodes glabrous. ***Leaves*** mostly basal or basal and cauline; sheaths 1–10 cm long, the lower ones longer than the internodes, the upper ones shorter, glabrous or scaberulous; ligules 2–5(–6) mm long, longer than wide, dorsally scaberulous, apices acute to truncate, erose or lacerate; blades 3–10(–19) cm long, 0.5–3(–4) mm wide, filiform to linear, conduplicate to involute, sometimes flat, scaberulous on both surfaces. ***Panicles*** (3–)5–12 cm long, (0.3–)0.5–1 cm wide, contracted, dense, spiciform, linear to lanceolate, often interrupted at the base, often partially included in the upper foliage sheats; branches appressed, rebranching below mid-length, scaberulous, with spikelets near their base, inferior branches up to 3 cm long; pedicels 0.5–3 mm long, appressed, scaberulous. ***Spikelets*** 2–3(–3.6) mm long, greenish to purplish; glumes subequal to unequal, lanceolate, apices acute to shortly acuminate, 1-veined, scaberulous on the keel, lower glume 2–3(–3.6) mm long, upper glume 1.8–2.8(–3.4) mm long; callus pubescent, with 2 bunches of trichomes; lemmas 1.2–2 mm long, elliptic, apices toothed, 5-veined, veins prominent, awned near the base, sometimes above mid-length, rarely awnless, awn 1.5–3.5 mm long, geniculate, reaching the lemma apices; paleas absent or up to 0.2 mm long; anthers 3, 0.5–1 mm long. ***Caryopsis*** 0.7–1.5 mm long, elliptic; endosperm soft to solid . 2n= 28 (Pohl and Davidse 1971).

**Figure 36.**
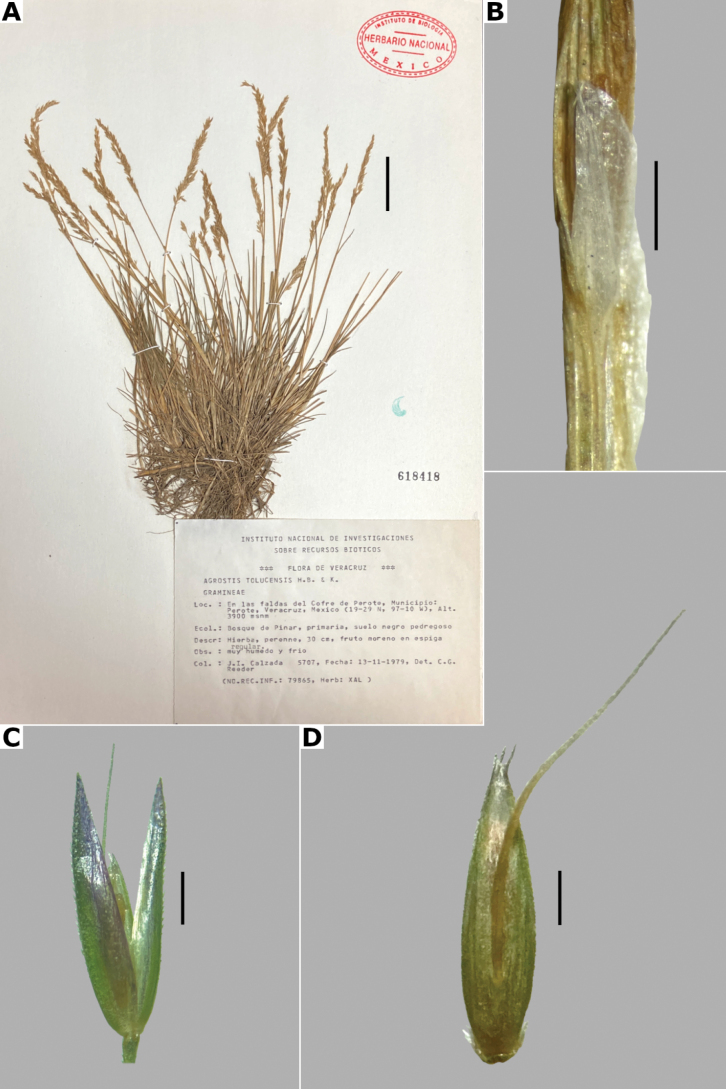
*Agrostistolucensis***A** whole plant **B** ligular area **C** spikelet **D** floret, abaxial view. Based on Calzada 5707 (MEXU). Scale bars: 3 cm (**A**); 1 mm (**B**); 0.5 mm (**C**); 0.3 mm (**D**).

**Figure 37.**
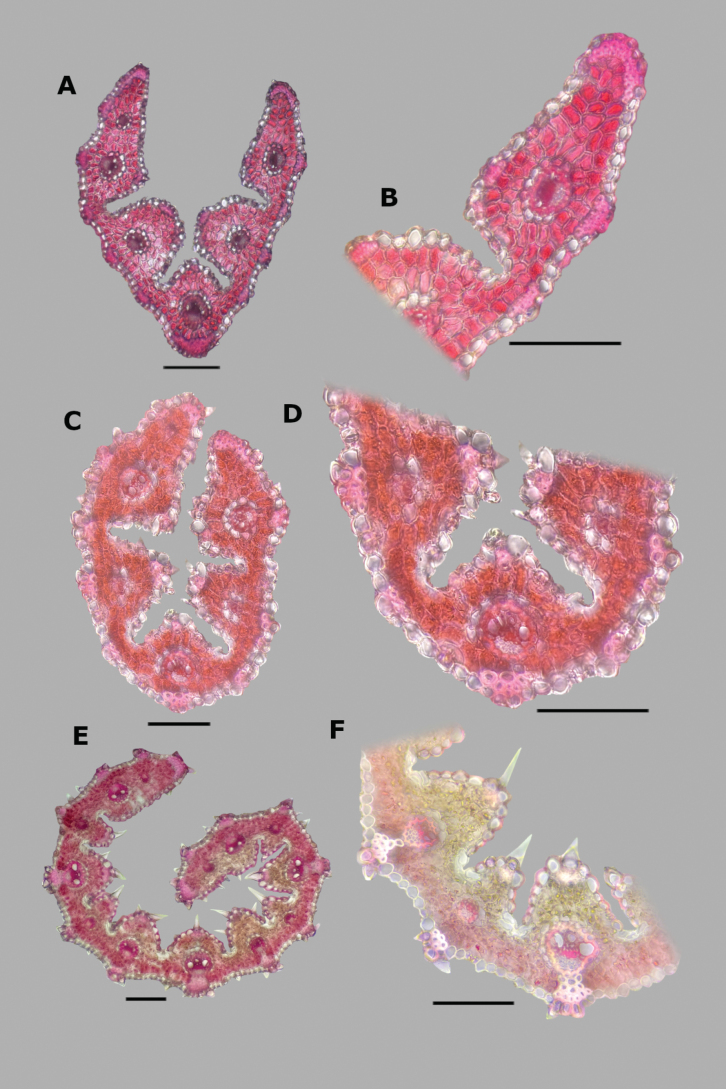
Leaf blade anatomy in transversal section of *Agrostis* species, in general view, and details of lateral bundles. **A**, **B***A.tolucensis***C**, **D***A.turrialbae***E**, **F***A.variabilis*. Scale bars: 0.1 mm.

***Leaf anatom*y.** Leaf blades involute to v-shaped in transversal section; adaxial furrows deep, narrow; adaxial ribs rounded to triangular; keel absent; first order bundles circular in outline, sheath not interrupted, abaxial and adaxial sclerenchyma in strands; second order bundles circular in outline, sheath not interrupted, abaxial and adaxial sclerenchyma; intercostal sclerenchyma present, abaxial; leaf margins with well-developed sclerenchyma caps, rounded; colorless cells absent (Fig. 37A, B). Lemmas with transversal thickenings irregular to oblong, wider than the unthickened portion of the wall; prickle hairs abundant (32E).

##### Distribution and habitat.

*Agrostistolucensis* is distributed from northern Mexico to Chile. In the study zone, it has been collected in Mexico City, and the Mexican states of Chiapas, Durango, Guerrero, Hidalgo, Jalisco, México, Michoacán, Morelos, Oaxaca, Puebla, Tlaxcala, and Veracruz; in the Guatemalan departments of Sacatepéquez and San Marcos (Fig. 26F). It has also been reported from the Mexican states of Colima, Guanajuato, and San Luis Potosí (Villaseñor 2016; Sánchez-Ken 2019), but no specimens from these states were found. *Agrostistolucensis* grows in open areas of temperate forests with *Abies*, *Pinus*, and *Quercus*, and alpine grasslands, between 1330–4520 m a.s.l. (Fig. 27F).

##### Phenology.

Specimens with spikelets have been collected year round, but most of them between the months of July and November (Fig. 28F).

##### Commentaries.

It has been reported that South American populations of this species develop long rhizomes (e.g. Renvoize 1998). This species is often confused with *A.exarata* and *A.subpatens* (see the notes under the description of these species). *Agrostistolucensis* is a variable species, and several infraspecific taxa have been described, none of which are recognized in this work. In the study zone, at least three forms have been observed, but there is a continuous interval of variation in the populations of this species: 1) small plants with narrow leaf blades and panicles shortly exceeding the foliage, 2) larger plants with narrow leaf blades and panicles long-exserted from the foliage, 3) larger plants with broader leaf blades, more open panicles, and lemmas with awn inserted above mid-length of the lemma. The plants of the latter form have been called *A.virescens*, but they fit well in the continuous interval of variation of the populations. This species is also confused with *A.meyenii* Trin. (see the note under excluded species).

##### Conservation status.

*Agrostistolucensis* is a common and widespread species in the study zone. It is represented by 244 collections, with several populations occurring in 17 protected areas. The EOO is 439,417 km^2^ and the AOO is 432 km^2^. Following the IUCN criteria, the preliminary assessment category is Least Concern (LC).

##### Representative specimens examined.

**Guatemala. San Marcos: Municipio Sibinal**, Volcán Tacaná, 500 m al E de Talquián, 15.13055556°N, 92.10694444°W, 4028 m alt., 24 May 2000, N. Gallardo et al. 8851 (MEXU). **México. Chiapas: Municipio Unión Juárez**, SE side of the summit of Volcán Tacaná, [15.09306218°N, 92.08369755°W], 3600 m alt., 10 Nov 1972, D.E. Breedlove 29340 (MEXU [*], MO); cráter del Volcán Tacaná, [15.09306218°N, 92.08369755°W], 4000 m alt., 9 Oct 1987, E. Martínez 20869 (MEXU [*]). **Durango: Municipio San Dimas**, 3 mi S of Guachichiles, upper slopes of cerro Huehento, 24.0786°N, 105.743°W, 3078–3249 m alt., 30 Sep 2008, P.M. Peterson and J.M. Saarela 22445 (CAN, US). **Guerrero: Municipio General Heliodoro Castillo**, cerro Teotepec, [17.46666667°N, 100.2166667°W], 3200 m alt., 17 Oct 1999, E. Domínguez 1200 (FCME [*]), 5 Dec 1993, M. González and C. Catalán 564 (CHAPA, MEXU). **Hidalgo: without municipality**, pasando desviación a Pachuca, autopista a México, 19 Jul 1976, J.J. Soto s.n. (IBUG). **Jalisco: Municipio Ciudad Guzmán**, 2 km antes de llegar a La Casita, camino El Refugio–Nevado de Colima, 2860 m alt., 3 Feb 1994, J. Reynoso 1735 (CIIDIR, IBUG). **Municipio San Gabriel**, N slopes of Nevado de Colima, [19.60110761°N, 103.5808018°W, 3000 m alt.], 19 Sep 1980, A.A. Beetle and R. Guzmán M-5380 (IBUG, MEXU [*]). **Mexico: Municipio Amecameca**, La Joya de Alcalican, pies de Iztaccíhuatl, [19.141667°N, 98.675°W], 3950 m alt., 23 Nov 1975, L. Alonso 66 (CHAPA, IBUG, MEXU); SW del Volcán Iztaccíhuatl, 1.5 km al SE de La Joyita, 19.13291667°N, 98.64144444°W, 3997 m alt., 15 Nov 2012, R. Hernández-Cárdenas and L. Arredondo-Amezcua 867 (MEXU [*]); km 20 carretera Amecameca–Tlamacas, [19.0917039°N, 98.67702965°W, 3402 m alt.], 2 Oct 1992, A. Miranda and G. Villegas 650 (MEXU [*]); La Joya de Alcalican, extremo SW del Iztaccíhuatl, [19.152778°N, 98.673333°W], 3900 m alt, 26 Nov 1978, H.J. Soriano 116 (ASU, CIIDIR, MEXU [*], XAL). **Municipio Zinacantepec**, camino al Nevado, [19.1225°N, 99.77888889°W], 3200 m alt., 1 Oct 1992, A. Miranda et al. 598 (MEXU [*, **]). **Mexico City**, **Alcaldía Tlalpan**, volcán Pelado, [19.151°N, 99.2171°W], 3100 m alt., 1 Jul 1985, A. Miranda et al. 25 (MEXU). **Michoacán: Municipio Angangeueo**, alrededores del Llano de las Papas, [19.65618889N, 100.2717278], 3200 m alt., 9 Oct 1988, J. Rzedowski 47403 (CHAPA, CIIDIR, IBUG, IEB, MEXU [**], XAL). **Morelos: Municipio Huitzilac**, Zempoala, [19.05034984°N, 99.31696647°W, 2812 m alt.], 1938, E. Lyonnet 2497 (MEXU, US). **Municipio Oaxtepec**, Oaxtepec, [18.90219847°N, 98.96287658°W], 1333 m alt., Aug 1952, F. Gallegos 438 (MEXU). **Oaxaca: Municipio San Miguel Amatlán**, 8.3 mi N of San Cualimojoyas on road towards Santa Maria Yavesia, 17.1819°N, 96.4445°W, 2794 m alt., 20 Sep 2008, P.M. Peterson and J.M. Saarela 22308 (US). **Puebla: Municipio Atzitzintla**, Sierra Negra, SW of Pico de Orizaba, summit of mountain, [18.98528°N, 97.310745°W], 4520 m alt., 10 Sep 1958, J.H. Beaman 2506 (MEXU, US). **Municipio San Nicolás de los Ranchos**, 6 km al SE de Paso de Cortés, brecha a Xalitzintla, [20.18°N, 98.44°W], 3400 m alt., 14 Sep 1988, P. Tenorio 15092 (MEXU, TEX); Buenavista, 5 km al E de Xalitzintla, [19.1°N, 98.55°W], 3300 m alt., 15 Feb 1988, P. Tenorio 15099 (MEXU [**]). **Tlaxcala: Municipio Huamantla**, volcán La Malinche, 4200 m alt., 4 Nov 1988, R. Acosta 2556 (CIB, MEXU [*]); parte alta de La Malinche, [19.25666667°N, 98.02833333°W], 3500 m alt., 7 Oct 1993, R. Hernández 96 (CIIDIR, MEXU). **Veracruz: Municipio Perote**, Cofre de Perote, 500 m al NO de la estación de televisión, [19.496389°N, 97.151389°W], 4030 m alt., 10 Sep 1992, B.V. Hernández 66 (MEXU [*], CHAPA, CIB, XAL). See Suppl. material 2 for the full list of examined specimens.

#### ﻿19. Agrostis turrialbae

Taxon classificationPlantaePoalesPoaceae

Mez, Repert. Spec. Nov. Regni Veg. 18(1–3): 4. 1922.

1F942862-15D4-53C3-9B64-BCE9C9965372

Figs 4R , 30F , 38

= Agrostis arcta Swallen, Contr. U.S. Natl. Herb. 29(9): 405. 1950. Type. Guatemala. Chimaltenango: moist roadside at Santa Elena, 17 Jul 1933, A.F. Skutch 422 (holotype: US (US00131720)).
= Agrostis vesca Swallen, Contr. U.S. Natl. Herb. 29(9): 405. 1950. Type. Guatemala. Chimaltenango: moist roadside at Santa Elena, 17 Jul 1933, A.F. Skutch 420 (holotype: US (US00131129)).

##### Type.

Costa Rica. Cartago: plateau au field W du Turrialba, 2600 m alt., 27 Jan 1884, H. Pittier 855 (holotype: B; isotypes: US (US00131127, US04023770 [image!])).

##### Description.

***Plants*** perennial, caespitose. ***Tillers*** extravaginal, with cataphylls. ***Culms*** 13–32 cm long, erect, nodes 1–2, glabrous, internodes glabrous. ***Leaves*** mostly basal; sheaths 0.8–6.5(–10) cm long, usually longer than the internodes, glabrous or scaberulous; ligules 0.5–1.6 mm long, longer than wide, dorsally scaberulous, apices acute to rounded, erose or lacerate; blades 1–9 cm long, 0.2–0.5 mm wide, filiform, conduplicate to involute, rarely flat in the upper leaves, scaberulous on both surfaces. ***Panicles*** 3.8–10 cm long, 1.7–5(–8) cm wide, open, lax, ovate, exserted from the upper sheaths; branches ascending to spreading, rebranching about or slightly above mid-length, scaberulous, without spikelets near their base, inferior branches 0.7–2.5 cm long; pedicels 0.7–4 mm long, ascending to spreading, scaberulous. ***Spikelets*** 1.5–2.4 mm long, purplish; glumes subequal to unequal, lanceolate, apices acute, 1-veined, scaberulous on the keel, lower glume 1.5–2.4 mm long, upper glume 1.4–2.3 mm long; callus pubescent, with 2 bunches of trichomes; lemmas 1.3–1.8 mm long, elliptic, apices entire, acute, sometimes irregularly toothed, 5-veined, veins prominent, unawned, rarely awned near the apices, awn ca. 0.2 mm long, straight; paleas absent or up to 0.2 mm long, veinless, glabrous; anthers 3, ca. 0.7 mm long. ***Caryopsis*** 1.5–2.2 mm elliptic; endosperm solid. 2n= unknown.

**Figure 38.**
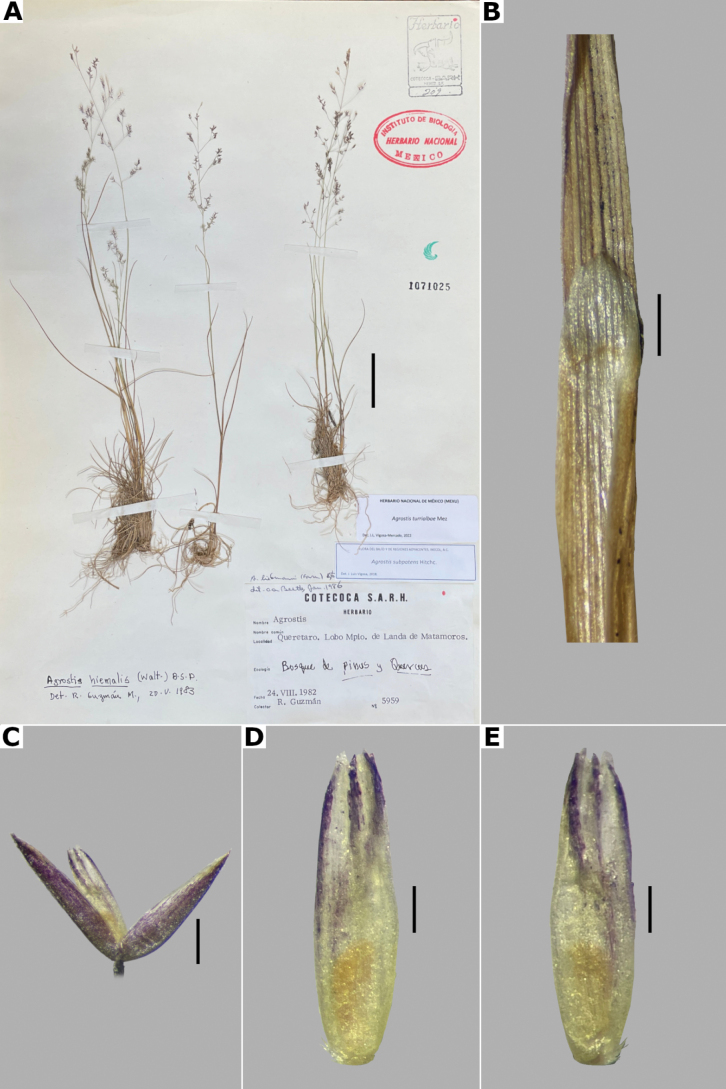
*Agrostisturrialbae***A** whole plant **B** ligular area **C** spikelet **E** floret, abaxial view **F** floret, adaxial view. Based on Guzmán 5959 (MEXU). Scale bars: 3 cm (**A**); 1 mm (**B**); 0.5 mm (**C**); 0.2 mm (**D, E**).

##### Anatomy and micromorphology.

Leaf blades v-shaped to involute in transversal section; adaxial furrows deep, narrow; adaxial ribs rounded to triangular; keel absent; first order bundles circular in outline, sheath interrupted abaxially, abaxial and adaxial sclerenchyma in strands; second order bundles circular in outline, sheath interrupted abaxially, abaxial and adaxial sclerenchyma in strands; intercostal sclerenchyma present, abaxial; leaf margins with well-developed sclerenchyma caps, rounded; colorless cells absent (Fig. 37C, D). Lemmas with transversal thickenings irregular to oblong, wider than the unthickened portion of the wall; prickle hairs present, abundant (Fig. 32F).

##### Distribution and habitat.

**Figure 39.**
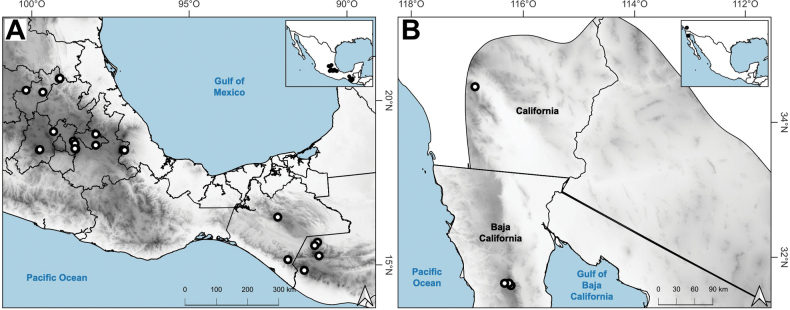
Map of known geographic distribution of *Agrostis* species, based on herbarium specimen data **A***A.turrialbae***B***A.variabilis*.

*Agrostisturrialbae* is distributed from central Mexico to Costa Rica. It has also been reported from Colombia and Venezuela (Luteyn 1999; Soreng and Peterson 2003). In the study zone, this species has been collected in the Mexican states of Chiapas, México, Querétaro, Tlaxcala, and Veracruz, and the Guatemalan departments of Huehuetenango and San Marcos (Fig. 39A). It has also been reported from Hidalgo (Sánchez-Ken 2019), but no specimens from this state were seen. *Agrostisturrialbae* grows in open areas of temperate forests, with *Pinus* and *Quercus*, and alpine grasslands, between 1600–4240 m a.s.l. (Fig. 27G).

##### Phenology.

Specimens with spikelets have been collected from June to February (Fig. 28G).

##### Commentaries.

Populations of this species from central Mexico have been confused with *A.subpatens* by some authors (e.g., Acosta 2001; Vigosa-Mercado and Ruiz-Sánchez 2020) (see the note under the description of that species). *Agrostisturrialbae* is similar to *A.perennans* sensu lato, and its identity has been put in doubt recently by Sylvester et al. (2020a). Both species share open panicles and unawned lemmas, as well as several lemma micromorphology characters. In the study zone, it has been found that the plants identified as *A.turrialbae* are consistently different from *A.perennans*, and are characterized by the small size of the plants, mostly basal leaves, leaf blades narrow and conduplicated to involute, with adaxial ribs rounded to triangular, with abaxial intercostal sclerenchyma, and purplish spikelets (vs. usually larger plants, usually basal and cauline leaves, but the basal ones often drying at anthesis, leaf blades wider and flat, with rounded adaxial ribs, without intercostal sclerenchyma, greenish to purplish spikelets in *A.perennans*). This species is scarcely different from *A.idahoensis*, from the southern USA (see the note under the description of that species).

##### Conservation status.

*Agrostisturrialbae* is a widespread species in the study zone. It is represented by 30 collections, with several populations occurring in six protected areas. The EOO is 183,426 km^2^ and the AOO is 84 km^2^. Following the IUCN criteria, the preliminary assessment category is Least Concern (LC).

##### Representative specimens examined.

**Guatemala. Huehuetenango: Municipio Santa Eulalia**, top of cerro Chemalito, Sierra de los Cuchumatanes, 3.5 mi W of Santa Eulalia, [15.7822223°N, 91.5111111°W], 3100–3150 m alt., 2 Aug 1942, J.A. Steyermark 49905 (F, US). **San Marcos: Municipio San Marcos**, between San Sebastián and summit of Volcán Tajumulco, [15.04824546°N, 91.86726456°W, 3353 m alt.], 13 Feb 1940, J.A. Steyermark 35477 (F). **Mexico. Chiapas: Municipio Motozintla**, near summit of cerro Mozotal., [15.419722°N, 92.336667°W], 2750 m alt., 24 Nov 1981, D.E. Breedlove and B.M. Bartholomew 55845 (MO). **México: Municipio Amecameca**: ladera SW del volcán Iztaccíhuatl, rumbo al primer Portillo, 19.13697222°N, 98.64898611°W, 4050 m alt., 8 Nov 2014, R. Hernández and S. Villalobos 2057 (IEB, MEXU [**]). **Querétaro: Municipio Colón**, antena El Zamorano, [20.93305556°N, 100.1797222°W], 3355 m alt., 24 Nov 1981, A. Mora and J. Ramírez 401-AMB (MEXU [*,**]). **Municipio Landa**, Lobo, [21.29275°N, 99.11930833°W], 1600 m alt., 24 Aug 1982, R. Guzmán 5959 (MEXU [*, **]). **Tlaxcala: Municipio Huamantla**, ladera N del Volcán La Malinche, 19.23486111°N, 98.03338889°W, 4190 m alt., 29 Jun 2013, R. Hernández-Cárdenas and L. Arredondo-Amezcua 1123 (IEB), 1929 (IEB). **Veracruz: Municipio Calcahualco**, La Cuchilla, camino al Pico de Orizaba, por Coscomatepec, [19.06728503°N, 97.19152382°W], 3160 m alt., 22 Jul 1982, R. Guzmán 5847 (MEXU). See Suppl. material 2 for the full list of examined specimens.

#### ﻿20. Agrostis variabilis

Taxon classificationPlantaePoalesPoaceae

Rydb., Mem. New York Bot. Gard. 1: 32. 1900.

D216FC37-FDB5-54FC-BE59-527D16F512E3

Fig. 4S , 30G , 40

Agrostis varians Trin., Mém. Acad. Imp. Sci. Saint-Pétersbourg, Sér. 6, Sci. Math., Seconde Pt. Sci. Nat. 6,4(3–4): 314. 1841, nom. illeg. hom., non Thuillier 1799. Type: America borealis [Rocky Mountains?], J.D. Hooker T-217 (holotype: LE-TRIN; isotypes: MO (MO-992441 [image!], NY (NY327643 [image!], US (US00156511 [image!])).

##### Type.

Based on *Agrostisvarians* Trin.

##### Description.

***Plants*** perennial, slender, usually caespitose, rarely rhizomatous. ***Tillers*** extravaginal, with cataphylls. ***Rhizomes*** if present, up to 2 cm long. ***Culms*** 10–45 cm long, erect, nodes 1–2, glabrous, internodes glabrous. ***Leaves*** mostly basal; sheaths 0.8–6(–10) cm long, the lower ones longer than the internodes, the upper ones shorter, scaberulous; ligules 1–2.2 mm long, longer than wide, dorsally scaberulous, apices acute, erose to lacerate; blades 1–7 cm long, 0.5–1 mm wide, filiform, conduplicate to convolute, scaberulous on both surfaces. ***Panicles*** 1.5–13 cm long, 0.2–0.7 cm wide, contracted, dense, spiciform, linear, sometimes interrupted at the base, sometimes partially included in the upper sheaths; branches appressed, rebranching from below mid-length, scaberulous, with spikelets near their base, inferior branches up to 1 cm long; pedicels 0.3–2.2 mm long, appressed, scaberulous. ***Spikelets*** 1.4–2 mm long, greenish to stramineous, often tinged with purpure; glumes subequal, lanceolate, apices acute, 1-veined, scaberulous on the keel, lower glume 1.4–2 mm long, upper glume 1.3–1.9 mm long; callus glabrous; lemmas 1–1.4 mm long, elliptic, apices entire, acute, 5-veined, veins prominent distally, usually unawned, rarely awned above mid-length, awn up to 1 mm long, straight, not reaching the lemma apices; paleas absent; anthers 3, 0.3–0.6 mm long. ***Caryopsis*** 0.9–1.2 mm long, elliptic; endosperm solid. 2n= 28 (Harvey 2007).

**Figure 40.**
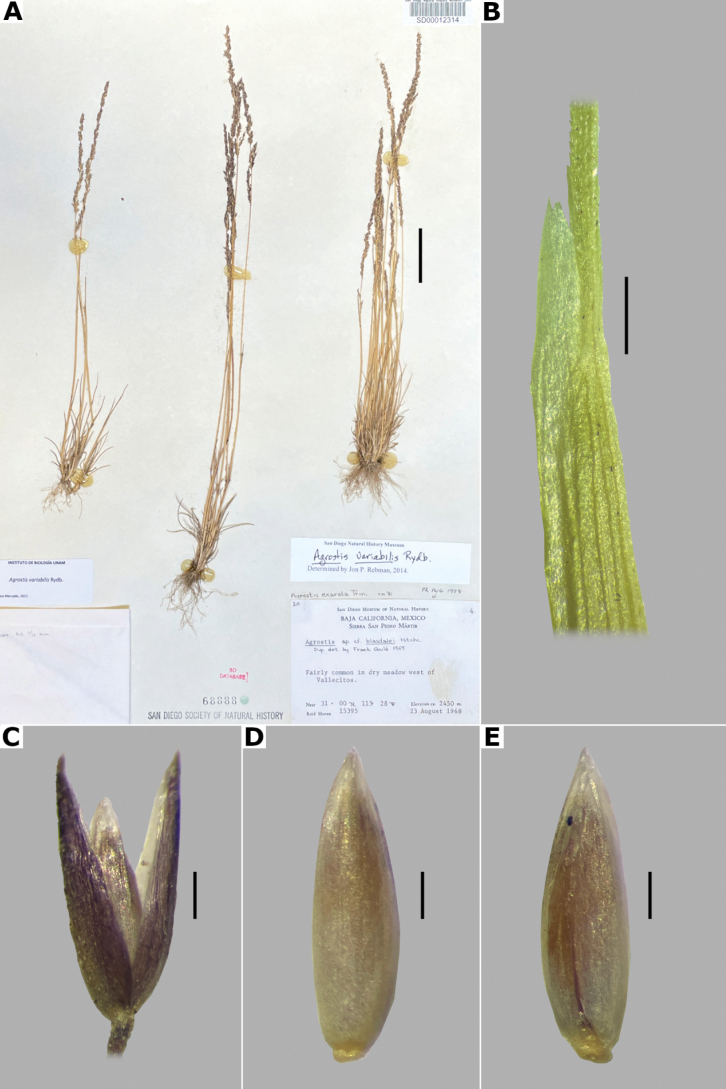
*Agrostisvariabilis***A** whole plant **B** ligular area **C** spikelet **E** floret, abaxial view **F** floret, adaxial view. Based on Moran 15395 (SD). Scale bars: 3 cm (**A**); 1 mm (**B**); 0.3 mm (**C**); 0.2 mm (**D, E**).

**Anatomy.** Leaf blades convolute to v-shaped in transversal section; adaxial furrows medium-sized to deep, narrow; adaxial ribs rounded to triangular: keel absent; first order bundles circular in outline, sheath interrupted abaxially, abaxial sclerenchyma in strands or girders, narrowing towards the bundle, adaxial sclerenchyma in strands; second order bundles circular in outline, sheath interrupted abaxially, abaxial and adaxial sclerenchyma in strands; intercostal sclerenchyma absent; leaf margins with well-developed sclerenchyma caps, rounded; colorless cells absent (Fig. 37E, F). Lemmas with transversal thickenings irregular to oblong, wider than the unthickened portion of the wall; prickle hairs present, scarce (32G).

##### Distribution and habitat.

*Agrostisvariabilis* is distributed from Alaska to the northern peninsula of Baja California, Mexico. In the study zone, it has been collected in the USA state of California, and the Mexican state of Baja California (Fig. 39B). This species has also been reported from Chihuahua (Sánchez-Ken 2019), but no specimens from this state were seen. *Agrostisvariabilis* grows in open areas of temperate forests with *Pinus*, between 2400–2639 m a.s.l. (Fig. 27H).

##### Phenology.

Specimens with spikelets have been collected from July to September (Fig. 28H).

##### Commentaries.

It has been reported for other regions that the spikelets can reach 2.5 mm long (Harvey 2007). This species is often confused with the smaller forms of *A.exarata*, but differs from them in the mostly basal leaves, with filiform and convolute to involute leaf blades, 0.5–1 mm wide (vs. basal and cauline leaves, leaf blades linear, flat, 1.5–4(–8) mm wide in *A.exarata*).

##### Conservation status.

*Agrostisvariabilis* is apparently a rare species in the study zone. It is represented by six collections, with its populations occurring in two protected areas. The EOO is 1,621 km^2^ and the AOO is 16 km^2^. Following the IUCN criteria, the preliminary assessment category is Endangered (EN).

##### Specimens examined.

**Mexico. Baja California: Municipio Ensenada**, Sierra San Pedro Mártir, fairly common in dry meadow W of Vallecitos, 31.01089°N, 115.49504°W, 2450 m alt., 23 Aug 1968, R. Moran 15395 (SD [*,**], UC); common on gravelly arroyo bank, Tasajera, ca. 3 km NW of Los Llanitos, 30.98333°N, 115.44167°W, 2500 m alt., 3 Sep 1979, R. Moran 28010 (SD); Sierra San Pedro Mártir, fairly common in arroyo, Jeffrey Pine forest, Yerba Buena, 31.01292°N, 115.48002°W, 2475 m alt., 16 Aug 1967, R. Moran and R.F. Thorne 14157 (SD). Sierra San Pedro Mártir, 25 Sep 1982, A. Preciado 299 (MEXU [*,**]); Sierra San Pedro Mártir, campground and main gate area, 31N, 115.557W, 2461 m alt., 14 Jul 2013, S. Ratay et al. 235 (SD). **USA. California: Riverside County**, Mt. San Jacinto State Park, N side of Hidden Lake, 33.80022°N, 116.64119°W, 2639 m alt., 12 Jul 1999, L. Hendrickson 10430 (BSCA).

### ﻿Excluded names

***Agrostisalba* L.**, **Sp. Pl. 1: 63. 1753.**

The rhizomatous plants with paleate spikelets were formerly treated under this name, but the original material corresponds to *Poanemoralis* L., so currently *A.alba* is a synonym of the latter.

***Agrostisavenacea* J.F. Gmel**., **Syst. Nat., ed. 13 2(1): 171. 1791.**

This name is a synonym of *Lachnagrostisfiliformis* (G. Forst.) Trin., which differs from *Agrostis* species in the hairy lemmas, well developed paleas and the rachilla prolongated as a hairy bristle.

***Agrostisblasdalei* Hitchc., Proc. Biol. Soc. Washington 41: 160–161. 1928.**

This species is distributed from Mendocino to Santa Cruz Counties, in California USA, where it grows in shrublands, and coastal cliffs and dunes (Harvey 2007). *Agrostisblasdalei* has been reported in Baja California, Mexico (Sánchez-Ken 2019), but the records of this species in the study zone appear to be erroneous identifications of specimens of *A.exarata*, from which differs in the panicles included in the upper sheaths at maturity, callus of the floret glabrous, and lemma shortly dentate (vs. panicles usually exserted, callus with at least a few trichomes, lemmas usually entire in *A.exarata*).

***Agrostisborealis* Hartm., Handb. Skand. Fl. (ed. 3) 17. 1838.**

This name is a synonym of *A.mertensii* Trin. (see below). It has been reported in the state of Mexico (Beetle et al. 1983).

***Agrostiscastellana* Boiss. & Reut. Diagn. Pl. Nov. Hisp. 26. 1842.**

This species is native to Europe. It has been reported from Durango, México (Sánchez-Ken 2019), but specimens from this state have not been seen. *Agrostiscastellana* is often confused with *A.capillaris*, which shares the rhizomatous habit, and paleate spikelets. *Agrostiscastellana* is distinguished in the ligules longer than wide, panicle branches densely scaberulous, lemmas often awned, and lemma of the terminal spikelet of each branchlet with callus and dorsal surface pubescent (vs. ligules usually shorter than wide, panicle branches scarcely scaberulous and lemmas usually unawned, with a glabrous callus or with a few and inconspicuous trichomes in *A.capillaris*)

***Agrostisdensiflora* Vasey, Contr. U.S. Natl. Herb. 3(1): 72. 1892.**

This species is distributed from coastal Oregon to California (Harvey 2007). The records of *A.densiflora* in the study zone appear to be erroneous identifications of specimens of *A.exarata*. It is distinguished in the dentate lemmas and anthers of 0.5–1.2 mm long (vs. lemmas usually entire, anthers of 0.3–0.7 mm long in *A.exarata*).

***Agrostisexserta* Swallen, Contr. U.S. Natl. Herb. 29(9): 404. 1950.**

This species has recently been transferred to the genus *Podagrostis*, under the name *P.exserta* (Swallen) Sylvester & Soreng (Sylvester et al. 2020b). It is distributed from Oaxaca, Mexico, to Guatemala (Vigosa-Mercado, 2022b). *Podagrostis* species are distinguished from *Agrostis* in the glumes as long as the floret, paleas well developed and rachilla prolonged as a glabrous bristle (vs. glumes longer than the floret, paleas often absent or minute, rachilla not prolonged).

***Agrostishumilis* Vasey, Bull. Torrey Bot. Club 10(2): 21. 1883.**

This species has been recognized as part of the genus *Agrostis* or *Podagrostis*. The molecular and morphological evidence confirms *Podagrostis* as a distinct genus (Peterson et al. 2020; Sylvester et al. 2020b). *Podagrostishumilis* (Vasey) Björkman is distributed in Canada and USA (Molina et al. 2021). See the note under *A.exserta* for the differences between *Agrostis* and *Podagrostis* species.

***Agrostisliebmannii* (E. Fourn.) Hitchc., in Britton, N. Amer. Fl. 17(7): 519. 1937.**

This species has recently been transferred to the genus *Podagrostis*, under the name *P.liebmannii* (Swallen) Sylvester & Soreng (Sylvester et al. 2020b). It is endemic to Mexico, known in the states of Hidalgo, Puebla and Veracruz (Vigosa-Mercado, 2022b). See the note under *A.exserta* for the differences between *Agrostis* and *Podagrostis* species.

***Agrostismertensii* Trin., Linnaea 10(3): 302. 1836.**

This species has a disjunct distribution in Scandinavia, Europe, Canada, Alaska to North Carolina, USA, and South America (Harvey 2007; Sylvester et al. 2020a). *Agrostismertensii* has been reported from central Mexico (Harvey 2007; Villaseñor 2016; Dávila et al. 2018). Some authors consider that *A.ghiesbreghtii* could be a synonym of this species (Steven P. Sylvester pers. communication). We have checked descriptions and type material of *A.mertensii* and its synonyms. At first glance, *A.ghiesbreghtii* fits well in the interval of variation of *A.mertensii*, and Mexican populations could represent large and robust forms of the latter. However, we have not seen a great number of specimens of *A.mertensii*, nor studied the anatomy and micropmorphology. At this moment, we prefer to recognize *A.ghiesbreghtii* as a distinct species, until more evidence is available.

***Agrostismeyenii* Trin., Mém. Acad. Imp. Sci. Saint-Pétersbourg, Sér. 6, Sci. Math., Seconde Pt. Sci. Nat. 6,4(3–4): 312. 1841.**

This species is distributed from Bolivia to Tierra del Fuego, Chile (Rúgolo and Molina 1997). *Agrostismeyenii* has been reported in the study zone from misidentified specimens of *A.tolucensis*, but is distinguished from it in the lemmas unawned or awned from the upper third, awn up to 1.2 mm long, straight, and paleas of 0.2–0.7 mm long (lemmas awned from mid-length or near the base, awn 1.5–3.5 mm long, geniculate, paleas absent or up to 0.2 mm long in *A.tolucensis*).

***Agrostisnovogaliciana* McVaugh, Fl. Novo-Galiciana 14: 41–42, f. 10. 1983.**

This species has recently been transferred to the genus *Podagrostis*, under the name *P.novogaliciana* (McVaugh) A.M. Soriano & Rúgolo (Molina et al. 2021). It is endemic to Jalisco, Mexico. See the note under *A.exserta* for the differences between *Agrostis* and *Podagrostis* species.

***Agrostispittieri* Hack., Oesterr. Bot. Z. 52(2): 60. 1902.**

This species is endemic to Costa Rica (Pohl and Davidse 1994). *Agrostispittieri* has been reported in the study zone from misidentified specimens of *A.subpatens*, but differs from it in the paleas of 0.5–1 mm long (vs. paleas absent or up to 0.2 mm long in *A.subpatens*).

***Agrostisrosei* Scribn. & Merr., Bull. Div. Agrostol., U.S.D.A. 24: 21, f. 5. 1901.**

This species has recently been transferred to the genus *Podagrostis*, under the name *P.rosei* (Swallen) Sylvester & Soreng (Sylvester et al. 2020b). It is endemic to Mexico, known from the states of Durango and Zacatecas (Vigosa-Mercado, 2022b). See the note under *A.exserta* for the differences between *Agrostis* and *Podagrostis* species.

***Agrostissemiverticillata* (Forssk.) C. Chr., Dansk Bot. Ark. 4(3): 12. 1922.**

This name is a synonym of *Polypogonviridis* (Gouan) Breistr., which differs from *Agrostis* species in the spikelets disarticulating below the glumes, with a fragment of the pedicel (vs. disarticulation above the glumes).

***Agrostistandilensis* (Kuntze) Parodi., Darwiniana 6: 158. 1943.**

This species is native to South America, introduced to USA, where it is known to occur in vernal pools in coastal zones of Monterrey, San Diego, and Solano Counties (Rúgolo 2007), outside the study zone. It has also has been reported from Baja California, Mexico (Beetle et al. 1983), but no herbarium specimens associated with this record have been found. Some authors have treated this and other South American species as part of the genus *Bromidium*. *Agrostistandilensis* differs from other *Agrostis* species in the hairy lemmas, with lateral veins excurrent as two long teeth (vs. lemmas glabrous or with hairs only on the callus, veins not or shortly excurrent).

## Supplementary Material

XML Treatment for Agrostis

XML Treatment for Agrostis bourgaei

XML Treatment for Agrostis calderoniae

XML Treatment for Agrostis capillaris

XML Treatment for Agrostis elliottiana

XML Treatment for Agrostis exarata

XML Treatment for Agrostis ghiesbreghtii

XML Treatment for Agrostis gigantea

XML Treatment for Agrostis hyemalis

XML Treatment for Agrostis idahoensis

XML Treatment for Agrostis laxissima

XML Treatment for Agrostis microphylla

XML Treatment for Agrostis pallens

XML Treatment for Agrostis perennans

XML Treatment for Agrostis scabra

XML Treatment for Agrostis stolonifera

XML Treatment for Agrostis subpatens

XML Treatment for Agrostis subrepens

XML Treatment for Agrostis tolucensis

XML Treatment for Agrostis turrialbae

XML Treatment for Agrostis variabilis

## Footnotes

See Suppl. material 1 for other heterotypic synonyms.

## ﻿Additional information

### Author ORCIDs

J. Luis Vigosa-Mercado https://orcid.org/0000-0002-9999-0272

Alfonso Delgado-Salinas https://orcid.org/0000-0002-9322-9968

Leonardo O. Alvarado Cárdenas https://orcid.org/0000-0002-4938-8339

Luis E. Eguiarte https://orcid.org/0000-0002-5906-9737

### Data availability

All of the data that support the findings of this study are available in the main text or Supplementary Information.

## Supplementary materials

Supplementary material 1 Other heterotypic synonyms Data type Text Explanation note This archive contains other heterotypic synonyms that were not cited in the main text. https://binary.pensoft.net/file/891276This dataset is made available under the Open Database License (http://opendatacommons.org/licenses/odbl/1.0/). The Open Database License (ODbL) is a license agreement intended to allow users to freely share, modify, and use this Dataset while maintaining this same freedom for others, provided that the original source and author(s) are credited.Author: J. Luis Vigosa-Mercado

Supplementary material 2 Additional specimens examined Data type Text Explanation note This archive contains other additional specimens examined that were not cited in the main text, in Word format. https://binary.pensoft.net/file/891277This dataset is made available under the Open Database License (http://opendatacommons.org/licenses/odbl/1.0/). The Open Database License (ODbL) is a license agreement intended to allow users to freely share, modify, and use this Dataset while maintaining this same freedom for others, provided that the original source and author(s) are credited.Author: J. Luis Vigosa-Mercado

Supplementary material 3 Additional specimens examined Data type Text Explanation note This archive contains other additional specimens examined that were not cited in the main text, in Excel format. https://binary.pensoft.net/file/891278This dataset is made available under the Open Database License (http://opendatacommons.org/licenses/odbl/1.0/). The Open Database License (ODbL) is a license agreement intended to allow users to freely share, modify, and use this Dataset while maintaining this same freedom for others, provided that the original source and author(s) are credited.Author: J. Luis Vigosa-Mercado
