# Supplementary material for: ﻿Revision of the genus Agrostis (Poaceae, Pooideae, Poeae) in Megamexico
Source: PhytoKeys. 2023 Aug 11;230:157–256. doi: 10.3897/phytokeys.230.105878 (PMC10439499; doi:10.3897/phytokeys.230.105878)
Supplement: Supplementary material 1 — Other heterotypic synonyms [file phytokeys-230-157_article-105878__-s001.docx]

**Supplementary Material 1. Other heterotypic synonyms**

***Agrostis* L., Sp. Pl. 1: 61. 1753, nom. et typ. cons.**

*= Candollea* Bastard ex Steud. Nomencl. Bot. (ed. 2) 1: 273. 1840, nom. illeg. hom., non. Mirb., 1803. Type: not designated

*= Agrestis* Bubani, Fl. Pyren. 4: 281. 1901, nom. illeg. superfl. Type: not designated

*= Pentatherum* Nábĕlek, Spisy Přír. Fak. Masarykovy Univ. 111: 8. 1929. Type: *Calamagrostis olympica* Boiss.

*= Senisetum* Honda, Bot. Mag. (Tokyo) 46: 371. 1932. Type: *Agrostis hideoi* Ohwi

*= Heptaseta* Koidz., Bot. Mag. (Tokyo) 47: 146. 1933, pro syn. Type: *H. japonica* Koidz.

***Agrostis capillaris* L., Sp. Pl. 1: 62. 1753.**

*= Agrostis sylvatica* Huds., Fl. Angl. 28. 1762. *Agrostis alba* L. var. *silvatica* (Huds.) K. Richt., Pl. Eur. 1: 43. 1890. *Agrostis polymorpha* Huds. var. *sylvatica* (Huds.) Huds., Fl. Angl. (ed. 2) 1: 32. 1778. *Agrostis alba* L. var. *sylvatica* (Huds.) Sm., Engl. Fl. 1: 93. 1824. *Agrostis alba* L. var. *sylvatica* (Huds.) K. Richt., Pl. Eur. 1: 43. 1890. *Frumentum sylvaticum* (Huds.) E.H.L. Krause, Bot. Centralbl. 73: 339. 1898. Type: ENGLAND. Staffordshire: habitat in sylvis humosis, Buddle s.n. [not located].

*= Agrostis pumila* L., Mant. Pl. 1: 31. 1767. *Agrostis polymorpha* Huds. var. *pumila* (L.) Huds., Fl. Angl. (ed. 2) 1: 31. 1778. *Agrostis vulgaris* With. var. *pumila* (L.) Pers., Syn. Pl. 1: 75. 1805. *Vilfa* divaricata (Hoffm.) Gray var. *pumila* (L.) Gray, Nat. Arr. Brit. Pl. 2: 147. 1821. *Agrostis rubra* L. var. *pumila* (L.) Wimm. & Grab., Fl. Siles. 1: 53. 1827. *Agrostis vulgaris* With. var. *pumila* (L.) Klett & Richter ex Peterm., Fl. Lips. Excurs. 83. 1838, *Agrostis vulgaris* With. var. *pumila* (L.) Mathieu, Fl. Gen. Belgique, Spermatoph. 1: 595. 1853, nom. hom. *Agrostis alba* L. subvar. *pumila* (L.) Coss. & Germ., Fl. Descript. Analytique Paris (ed. 2) 797. 1861. *Agrostis alba* L. subvar. *pumila* (L.) Coss. & Durand, Fl. Descript. Analytique Paris (ed. 2) 797. 1861, nom. hom. *Agrostis tenuis* Siibth. var. *pumila* (L.) Druce, List. Brit. Pl. 79. 1908. *Agrostis capillaris* L. var. *pumila* (L.) Druce, Fl. Oxfordshire (ed. 2) 474. 1927. Type. SWEDEN. Hammarby, *anonymous s.n.* (lectotype, designated by Philipson (1935: 71): LINN (LINN-84.28 [image!]).

*= Agrostis polymorpha* Huds., Fl. Angl. (ed. 2) 1: 31. 1778, nom. illeg. superfl*. Agrestis polymorpha* (Huds.) Bubani, Fl. Pyren. 4: 283. 1901. Type: ENGLAND [not located].

*= Agrostis stricta* Muhl., Descr. Gram. 65. 1817, nom. illeg. hom., non J.F. Gmel., 1791. *Agrostis diffusa* Muhl. ex Spreng., Syst. Veg. 1: 260. 1825 [1824], pro syn. Type. USA. Habitat in Nova Anglia et Carolina (holotype [possible]: PH (PH00031990 [image!]).

*= Agrostis vulgaris* With., Arr. Brit. Pl. (ed. 3) 2: 132. 1796. *Decandolia vulgaris* (With.) Bastard, Essai Fl. Maine et Loire 28. 1809. *Vilfa vulgaris* (With.) P. Beauv., Ess. Agrostogr. 16, t. 5, f. 8. 1812. *Vilfa vulgaris* (With.) Gray, Nat. Arr. Brit. Pl. 2: 146. 1821, nom. illeg. hom. *Agrostis alba* L. var. *vulgaris* (With.) Coss. & Durieu, Expl. Sci. Algérie 2: 63. 1855. *Agrostis alba* var. *vulgaris* (With.) Plues, Brit. Grass. 151. 1867, nom. illeg. hom. *Agrostis stolonifera* L. var. *vulgaris* (With.) Čelak., Prodr. Fl. Böhmen 710. 1881, nom. illeg. hom., non Heuff., 1858. *Agrostis alba* L. var. *vulgaris* (With.) Fiori, Fl. Italia 1: 63. 1896, nom. illeg. hom. *Agrostis alba* L. subsp. *vulgaris* (With.) Douin, in Bonnier, Fl. Ill. France 11: 142. 1931. Type: ENGLAND [not located].

= *Agrostis hispida* Willd., Sp. Pl. 1: 370. 1797. *Agrostis vulgaris* With. var. *hispida* (Willd.) G. Mey., Hannover. Mag. 1823: 140. 1824. *Agrostis vulgaris* With. var. *hispida* (Willd.) Gaudin, Fl. Helv. 1: 191. 1828, nom. illeg. hom. *Agrostis* *stolonifera* L. var. *hispida* (Willd.) Farw., Michigan Acad. Sci. Rep. 1919: 351. 1920. *Agrostis tenuis* Sibth. var. *hispida* (Willd.) Philipson, J. Linn. Soc., Bot. 51: 86. 1937. Type. Habitat in Europea pratis, pascuis et arvensis [not located].

*= Agrostis stricta* Willd., Sp. Pl. 1: 366. 1797, nom. illeg. hom., non J.F. Gmel., 1791*.* *Agrostis alba* L. var. *aristata* A. Gray, Manual 578. 1848, nom. illeg. hom., non Spenn., 1825. *Agrostis alba* L. var. *stricta* Alph. Wood, Class-book Bot. 774. 1861. *Agrostis palustris* Huds. var. *stricta* (Willd.) House, New York State Mus. Bull. 254: 98. 1924. Type: habitat in America boreali (syntypes [possible]: B-W (B-W 01685-010 [image!], B-W 01685-020 [image!])).

*= Agrostis tenella* Hoffm., Fl. Germ. ed. 2 1: 36. 1800. *Agrostis vulgaris* With. var. *tenella* (Hoffm.) Gaudin, Agrost. Helv. 1: 84. 1811. *Agrostis rubra* L. var. *tenella* (Hoffm.) Wimm. & Grab., Fl. Siles. 1: 52. 1827. *Agrostis stolonifera* L. var. *tenella* (Hoffm.) Wimm. & Grab., Fl. Siles. 1: 52. 1827. Type. EUROPE [not located].

*= Trichodium strictum* Roem. & Schult., Syst. Veg. (ed. 15 bis) 2: 281. 1817. *Agrostis stricta* (Roem. & Schult.) Buse, Pl. Jungh. 3: 341. 1854, nom. illeg. hom., non J.F. Gmel., 1791. Type. In America boreali et Groenlandia [not located].

*= Agrostis laxa* Gray, Nat. Arr. Brit. Pl. 2: 147. 1821. Type: ENGLAND [not located].

*= Agrostis lithuanica* Besser ex Roem. & Schult., Mant. 3(Add. 1): 568. 1827. Type: LITUANIA. In graminosis circa Vilnam, anonymous s.n. [ex herb. Besser] (holotype [possible]: HAL (HAL0134569 [image!]).

*= Agrostis vulgaris* With. var. *aristata* Parn., Grass. Scotland 1: 34. 1842. *Agrostis tenuis* Sibth var*. aristata* (Parn.) Druce, List Brit. Pl.: 79. 1908. *Agrostis tenuis* Sibth. fo. *aristata* (Parn.) Wiegand, Rhodora 26(301): 2. 1924. *Agrostis capillaris* L. var. *aristata* (Parn.) Druce, Fl. Oxfordshire (ed. 2): 474. 1927. Type. SCOTLAND [not located].

*= Agrostis alba* L. var. *minor* Vasey, Contr. U.S. Natl. Herb. 3(1): 78. 1892. *Agrostis stolonifera* L. var. *minor* (Vasey) Farw., Rep. Michigan Acad. Sci. 6: 202. 1904, nom. illeg. hom., non Meinsh., 1878. Type: USA. Washington D.C., 1887, G.Vasey s.n. (holotype: not located; isotype: US (US00131717 [image!])).

*= Agrostis capillaris* L. var. *aristulata* Hitchc., Proc. Biol. Soc. Washington 41: 160. 1928. Type. USA. Virginia: on lowland clay soil, in shade of bushes, at Alexandria, 4 Jul 1905, A. Chase 87 (holotype: US (US00131732 [image!]).

***Agrostis exarata* Trin., Gram. Unifl. Sesquifl. 207. 1824.**

*= Agrostis canina* L. var. *aenea* Trin., Mém. Acad. Imp. Sci. St.-Pétersbourg, Sér. 6, Sci. Math. 2(2): 170. 1832. *Agrostis aenea* (Trin.) Trin., Mém. Acad. Imp. Sci. Saint-Pétersbourg, Sér. 6, Sci. Math., Seconde Pt. Sci. Nat. 6,4(3-4): 332. 1841, nom. illeg. hom., non Spreng., 1827. *Agrostis exarata* Trin. var. *aenea* (Trin.) Griseb., Fl. Ross. [Ledebour] 4(13): 441. 1852. *Agrostis alaskana* Hultén, Fl. Aleut. Isl. 71. 1937. Type: USA. Alaska: Sitka, 1829, K. Mertens s.n. (syntypes: LE-TRIN (LE-TRIN-1585.02, LE-TRIN-1585.01), S (S-G-237 [image!]).

*= Agrostis canina* L. var. *melaleuca* Trin., Mém. Acad. Imp. Sci. St.-Pétersbourg, Sér. 6, Sci. Math. 2(2): 170. 1832. *Agrostis melaleuca* (Trin.) Hitchc., U.S.D.A. Bur. Pl. Industr. Bull. 68: 51. 1905. Type: USA. Alaska: Sitka, T. Drummond 217 (holotype: LE; isotypes: K, US [fragm. ex K] (US00156386 [image!])).

*= Agrostis exarata* Trin. var. *minor* Hook., Fl. Bor.-Amer. 2: 239. 1839. *Agrostis drummondii* Torr. ex Hook. f., Fl. Antarct. 2: 372. 1846, pro syn. *Agrostis exarata* Trin. subsp. *minor* (Hook.) C.L. Hitchc., Vasc. Pl. Pacif. N.W. 1: 467. 1969. Type: USA. Valleys of the Rocky Mountains, T. Drummond 182 (syntype [possible]: NY (NY327626 [image!]), D. Douglas 243 (syntype [possible]: NY (NY00327627 [image!])).

*= Agrostis asperifolia* Trin., Mém. Acad. Imp. Sci. Saint-Pétersbourg, Sér. 6, Sci. Math., Seconde Pt. Sci. Nat. 6,4(3-4): 317. 1841. *Agrostis exarata* Trin. fo. *asperifolia* (Trin.) Vasey, U.S.D.A. Div. Bot. Bull. 13(1): 31. 1892. Type: NORTH AMERICA [possible]. T Hooker 182 (holotype: LE (LE-TRIN-1591.0); isotype: US [fragm. ex LE-TRIN] (US00156373 [image!])).

*= Agrostis grandis* Trin., Mém. Acad. Imp. Sci. Saint-Pétersbourg, Sér. 6, Sci. Math., Seconde Pt. Sci. Nat. 6,4(3-4): 316. 1841. Type: USA. Columbia, J*.D. Hooker 376* (holotype: LE-TRIN; isotype: US fragm. ex LE-TRIN] (US00156433 [image!])).

*= Agrostis scouleri* Trin., Mém. Acad. Imp. Sci. Saint-Pétersbourg, Sér. 6, Sci. Math., Seconde Pt. Sci. Nat. 6,4(3-4): 329. 1841. Type: CANADA. Vancouver: Nutka Island, Scouler s.n. (holotype: LE-TRIN).

*= Agrostis albicans* Buckley, Proc. Acad. Nat. Sci. Philadelphia 14: 91. 1862. *Agrostis oregonensis* Nutt. ex A. Gray, Proc. Acad. Nat. Sci. Philadelphia 14: 334. 1862, pro syn. Type: USA. Oregon: Columbia Woods, T. Nutall s.n. (holotype: PH [specimen of the left] (PH00024938 [image!]); isotype: US [fragm. ex PH] (US00156364 [image!])).

*= Agrostis berlandieri* E. Fourn., Mexic. Pl. 2: 96. 1886. *Agrostis berlandieri* E. Fourn. ex Hemsl., Biol. Cent.-Amer., Bot. 3: 550. 1885, nom. nud. Type: MEXICO. Totoniho, 25 Mar 1827, J.L. Berlandier 531 (holotype: P (P00740578 [image!]); isotypes: P (P00740579 [image!]), US (US00156376), W (W0007178 [image!])).

*= Agrostis exarata* Trin. var. *pacifica* Vasey, U.S.D.A. Div. Bot. Spec. Bull. (new ed.) 1889: 107. 1889. Type: USA California: Colton, Sep 1882, M.E. Jones s.n. (holotype: US (US00131743 [image!])).

*= Agrostis microphylla* Steud. var. *major* Vasey, Contr. U.S. Natl. Herb. 3(1): 58. 1892. *Agrostis* *exarata* trin. var. *microphylla* S. Watson ex Vasey, Contr. U.S. Natl. Herb. 3(1): 58. 1892, pro syn*.* Type: USA. Nevada: Truckee Valley, Sep 1867, S. Watson 1284 (syntypes: GH (GH00022970 [image!]), NY (NY00327636 [image!]), US (US00131089 [image!], US00131090 [image!])).

*= Agrostis ampla* Hitchc., U.S.D.A. Bur. Pl. Industr. Bull. 68: 38. 1905. Type: USA. Oregon: Multnomah Co. Near Rooter Rock, wet rocks near, 16 Jul 1885, W.N. Suksdorf 135 (holotype: US (US00131719 [image!])).

*= Agrostis longiligula* Hitchc., U.S.D.A. Bur. Pl. Industr. Bull. 68: 54. 1905. Type: USA California: Mendocino County, near Fort Bragg, 1899, J.B. Davy and W.C. Blasdale 6110 (holotype: US (US00131085 [image!])).

*= Agrostis alaskana* Hultén var. *breviflora* Hultén, Fl. Aleut. Isl. 71. 1937. Type: USA Alaska: Aleutian Islands, Umnak, 30 Jul 1932, E. Hultén 7084 (holotype: S (S-G238).

*= Agrostis exarata* Trin. var. *purpurascens* Hultén, Fl. Aleut. Isl. 73. 1937. Type: USA. Alaska: Aleutian Islands, 24 Aug 1932, W.J. Eyerdam 2285 (holotype: S (S-G-253 [image!]); isotype: K (K000838208 [image!])).

*= Agrostis longiligula* Hitchc. var. *australis* J.T. Howell, Leafl. W. Bot. 4(10): 246. 1946. Type: USA California: Marin County, Ledum Swamp on road to Pt. Reyes, 4 Aug 1943, J.T. Howell 18250 (holotype: CAS (CAS0000196 [image!]); isotypes: CAS (CAS0000197 [image!]), UC (UC1177034 [image!]), US (US00131086 [image!])).

***Agrostis gigantea*** **Roth, Tent. Fl. Germ. 1: 31. 1788.**

*= Agrostis dubia* Leers, Fl. Herborn. 21. 1775. *Agrostis compressa* Willd., Bot. Mag. (Römer & Usteri) 4(11): 39. 1790, nom. illeg. superfl. Type: ALEMANIA. Habitat in motosis apricis am Homberg [not located].

*= Agrostis nigra* With., Arr. Brit. Pl. (ed. 3) 2: 131. 1796. *Vilfa nigra* (With.) Gray, Nat. Arr. Brit. Pl. 2: 145. 1821. *Agrostis tenuis* Sibth. var. *nigra* (With.) Druce, List Brit. Pl. 79. 1908. *Agrostis capillaris* L. var. *nigra (With.)* Druce, Comital. Fl. Brit. Isl. 350. 1932, nom. nud. Type: ENGLAND [not located].

*= Agrostis dispar* Michx., Fl. Bor.-Amer. 1: 52. 1803. *Vilfa dispar* (Michx.) P. Beauv., Ess. Agrostogr. 16. 1812. *Agrostis alba* L. var. *dispar* (Michx.) Alph. Wood, Class-book Bot. (ed. 1861): 774. 1861. *Agrostis gigantea* Roth var. *dispar* (Michx.) Philipson, J. Linn. Soc., Bot. 51: 93. 1937. Type: USA. South Carolina: habitat in Carolina inferiore, A. Michaux s.n. (holotype: P (P00680072 [image!]); isotypes: P (P00740541 [image!], P00740544 [image!], P00680073 [image!], P00740543 [image!], P00740542 [image!]).

*= Agrostis seminuda* Knapp, Gram. Brit. pl. 115 1804. Type: GREAT BRITAIN [not located].

*= Agrostis diffusa* Host, Icon. Descr. Gram. Austriac. 4: 32. 1809. *Agrostis stolonifera* L. var. *diffusa* (Host) Neilr., Fl. Nied.-Oesterr. 2: 43. 1859. *Agrostis alba* L. var. *diffusa* (Host) Asch. & Graebn., Syn. Mitteleur. Fl. 1: 174. 1899. *Agrostis stolonifera* L. fo. *diffusa* (Host) Maire & Weiller, Fl. Afrique N. 2: 123. 1953. Type: not designated

*= Agrostis decumbens* Host, Icon. Descr. Gram. Austriac. 4: 31. 1809. *Agrostis signata* Schur var. *decumbens* (Host) Schur, Oesterr. Bot. Z. 9: 48. 1859. Type: AUSTRIA [not located].

*= Vilfa alba* Gray, Nat. Arr. Brit. Pl. 2: 145. 1821, nom. illeg. hom., non. P. Beauv., 1812. Type: GREAT BRITAIN [not located].

*= Vilfa alba* (L.) P. Beauv. var. *ramosa* Gray, Nat. Arr. Brit. Pl. 2: 145. 1821. *Agrostis gigantea* Roth var. *ramosa* (Gray) Philipson, J. Linn. Soc., Bot. 51: 91. 1937. *Agrostis stolonifera* L. var. *ramosa* (Gray) Veldkamp, Blumea 28(1): 223. 1982. Type: GREAT BRITAIN [not located].

*= Agrostis alba* L. var. *vulgaris* G. Mey., Neues Hamburg. Mag. 1823: 134. 1824. Type: GERMANY [not located].

*= Agrostis alba* L. var. *maior* Gaudin, Fl. Helv. 1: 189. 1828. *Agrostis stolonifera* L. var. *major* (Gaudin) Farw., Rep. (Annual) Michigan Acad. Sci. 21: 351. 1919 [1920], orth. var. Type: SWITZERLAND. Berna: locis humidis arenosisque cum *Arundinibus*, passim *Neviduni,* herbarium Albrecht de Haller filius (lectotype (designated by Hackel and Briquet, 1907: 37): G).

*= Agrostis campestris* Phil., Linnaea 29(1): 87. 1858. Type: CHILE. Insula Chiloë, locis cultis, F. Fonk s.n. (holotype: SGO (SGO000000035 [image!]); isotypes: BAB (BAB00000205[image!], W (W19160040661 [image!])).

*= Agrostis stolonifera* L. var. *flagellare* Neilr., Fl. Nieder-Oesterr. 43. 1859. Type: AUSTRIA [not located].

*= Agrostis exarata* Trin. var. *mutica* Hicken, Physis (Buenos Aires) 2: 6. 1915. Type: ARGENTINA. Río Negro: vicinity of General Roca, 24 Dic 1914, W. Fischer 199 (holotype: SI (SI000496 [image!]); isotypes: BAA [fragm. ex SI] (BAA00001330 [image!]), BKL (BKL00000466 [image!]), CM (CM2611 [image!]), K, MO (MO-2114569 [image!]), NY (NY00327667 [image!]), SI (SI000497! [image!]), US (US00131744 [image!])).

*= Agrostis stolonifera* L. fo. *aristigera* Fernald, Rhodora 35: 317. 1933. *Agrostis alba* L. fo. *aristata* (Fernald) Fernald, Rhodora 49(580): 112. 1947, nom. illeg., orth. var. Type: USA. Massachusetts: Granville, along brook in meadow, 27 Jun 1914, F.C. Seymour 309 (holotype: GH (GH00023003 [image!])).

*= Agrostis praticola* Klokov, Bot. Mater. Gerb. Bot. Inst. Komarova Akad. Nauk SSSR 12: 40. 1950. *Agrostis gigantea* Roth var. *praticola* (Klokov) Tzvelev, Novosti Sist. Vyssh. Rast. 8: 58. 1971. Type: UKRAINE [not located].

*= Agrostis graniticola* Klokov, Bot. Mater. Gerb. Bot. Inst. Komarova Akad. Nauk SSSR 12: 40. 1950. Type: UKRAINE Mariupol: Río Berda, Jul 1930, Anonymous s.n. (holotype: LE).

*= Agrostis sabulicola* Klokov, Bot. Mater. Gerb. Bot. Inst. Komarova Akad. Nauk SSSR 12: 37. 1950*.* Type: UKRAINE. Odessa: Tsyuryupinsk, 12 Jul 1932, M. Kosets s.n*.* (holotype: LE).

***Agrostis hyemalis*** **(Walter) Britton, Sterns & Poggenb., Prelim. Cat. 68. 1888.**

*= Trichodium laxiflorum* Muhl., Descr. Gram. 60. 1817, nom. illeg. hom., non Michx., 1803. *Trichodium laxum* Schult., Mant. 2: 157-158. 1824. Type: AMERICA. America septentrionalis, habitat in arvis siccis [not located].

*= Agrostis laxiflora* Poir., in Lam., Encycl., Suppl. 1(1): 255. 1810. Type: USA. Carolinas, M. Bosc s.n. (holotype: P?).

*= Agrostis aphanes* Trin., Mém. Acad. Imp. Sci. Saint-Pétersbourg, Sér. 6, Sci. Math., Seconde Pt. Sci. Nat. 6,4(3-4): 346. 1841. Type: CANADA. Terra Nova, 1828, C.S. Kunth 413 (holotype: LE-TRIN (LE-TRIN-1589.01); isotype: US [fragm. ex LE-TRIN] (US00156369 [image!])).

*= Agrostis leptos* Steud., Syn. Pl. Glumac. 1: 169. 1855 [1854]. Type: USA Louisiana, Hartman s.n. (holotype: P (P00740564 [image!])).

*= Agrostis antecedens* E.P. Bicknell, Bull. Torrey Bot. Club 35(10): 473-474. 1908. Type: USA Massachusetts: Nantucket Island, dry places near Miacomet Pond, 9 Jun 1908, E.P. Bicknell s.n. (holotype: NY (NY00327615 [image!]; isotype: US [fragm. ex NY] (US00156368 [image!])).

***Agrostis microphylla* Steud., Syn. Pl. Glumac. 1: 164. 1855 [1854].**

*= Polypogon alopecuroides* Buckley, Proc. Acad. Nat. Sci. Philadelphia 14: 88. 1862. *Agrostis alopecuroides* (Buckley) A. Gray, Proc. Acad. Nat. Sci. Philadelphia 14: 333. 1862, nom. illeg. hom., non Lam. 1791. *Deyeuxia alopecuroides* Nutt. ex A. Gray, Proc. Acad. Nat. Sci. Philadelphia 14: 333. 1862, pro syn. Type: USA Oregon: Columbia plains, T. Nutall s.n. (holotype: PH [plant of the right] (PH00021093 [image!]); isotypes: BM (BM001042282 [image!], BM001042283 [image!]), MO (MO-123095 [image!]), US (US01164914)).

*= Agrostis inflata* Scribn., Canad. Rec. Sci. 6: 152. 1894*.* Type: CANADA. Vancouver: Victoria, 9 Jun 1893, J. Macoun 258 (syntype: GH (GH00022969 [image!])), 259 (syntypes: K (K000838214[image!]), MO (MO-126927 [image!]), VT (UVMVT015685 [image!])).

***Agrostis pallens* Trin., Mém. Acad. Imp. Sci. Saint-Pétersbourg, Sér. 6, Sci. Math., Seconde Pt. Sci. Nat. 6,4(3-4): 328. 1841.**

*= Agrostis foliosa* Vasey, Bull. Torrey Bot. Club 13: 55. 1886, nom. illeg. hom., non Roem. & Schult., 1817. *Agrostis diegoensis* Vasey var. *foliosa* (Vasey) Vasey, Contr. U.S. Natl. Herb. 3(1): 74. 1892. *Agrostis pallens* Trin. var. *foliosa* (Vasey) Hitchc., U.S.D.A. Bur. Pl. Industr. Bull. 68: 34. 1905. *Agrostis pallens* Trin. var. *vaseyi* H. St. John, Fl. SE. Washington: 30. 1937. Type: USA. Oregon: on dry ridges, Cascade Mountains near Mount Hood, 13 Aug 1881, T.J. Howell s. n. (holotype: US (US00131749 [image!]); isotypes: BM (BM001042285 [image!]), BR (BR0000006863944 [image!]), GH (GH00022963 [image!]), LE (LE00009320 [image!]), MO (MO-123093 [image!]), NY (NY00327628 [image!], NY00688632[image!]), OSC (OSC0001222 [image!], OSC0001813[image!]), PH (PH00000768[image!])).

*= Agrostis canina* L. var. *stolonifera* Vasey, Contr. U.S. Natl. Herb. 3(1): 75. 1892, nom. illeg. hom., non Blytt, 1847. Type: USA. Oregon, 1882, L.F. Henderson and J.T. Howell s.n. (holotype: US (US00131731 [image!]); isotype [possible]: OSC (OSC0001214 [image!])).

*= Agrostis lepida* Hitchc., Fl. Calif. 1: 121. 1912. . Type: USA. California: Siberian Pass, Sequoia National Park, 6 Sep 1908., A.S. Hitchcock 3455 (holotype: US (US00131083 [image!]; isotype: ISC (ISC-v-0000484 [image!])).

***Agrostis perennans*** **(Walter) Tuck., Amer. J. Sci. Arts 45: 44. 1843.**

*= Cornucopiae altissima* Walter, Fl. Carol. 74. 1788. *Agrostis altissima* (Walter) Tuck., Amer. J. Sci. Arts 45(1): 44. 1843. *Trichodium altissimum* (Walter) Michx. ex Alph. Wood, Class-book Bot. 599. 1847. Type: not designated.

*= Alopecurus carolinianus* Spreng., Bot. Gart. Halle 10. 1801, nom. illeg. Hom., non Walter, 1788. Type: USA. Carolina [not located].

*= Agrostis michauxii* Trin., Gram. Unifl. Sesquifl.: 206. 1824, nom. illeg. hom., non. Zucc., 1809. *Trichodium decumbens* (Walter) Michx., Fl. Bor.-Amer. 1: 42. 1803, nom. illeg. superfl. *Agrostis decumbens* (Michx.) Link, Hort. Berol. 1: 80. 1827, nom. illeg. hom., non Host 1809. Type: USA. Habitat in Carolina praesertim in umbrosis ripariis amnium, A. Michaux s.n. (holotype: P).

*= Trichodium elatum* Pursh, Fl. Amer. Sept. 1: 61. 1814 [1813]. *Agrostis elata* (Pursh) Trin., Mém. Acad. Imp. Sci. Saint-Pétersbourg, Sér. 6, Sci. Math., Seconde Pt. Sci. Nat. 6,4(3-4): 317. 1841. *Agrostis perennans* (Walter) Tuck. var. *elata* (Pursh) Hitchc., U.S.D.A. Bur. Pl. Industr. Bull. 68: 50. 1905. *Agrostis hyemalis* (Walter) Britton, Sterns & Poggenb var. *elata* (Pursh) Fernald, Rhodora 23(274): 229. 1921 [1922]. Type: USA. F.T. Pursh s.n.? (holotype [possible]: K (K000838197 [image!])).

*= Vilfa elegans* Kunth, in Humb., Bonpl. & Kunth, Nov. Gen. Sp. (quarto ed.) 1: 113. 1815 [1816]. *Agrostis elegans* (Kunth) Roem. & Schult., Syst. Veg. (ed. 15 bis) 2: 362. 1817, nom. illeg. hom., non Salisb., 1796. *Agrostis pulchella* Kunth, Enum. Pl. 1: 223. 1833, nom. illeg. hom., non Roth, 1817. *Agrostis humboldtiana* Steud., Nomencl. Bot. 1: 40. 1840. Type: ECUADOR. Cochapamba, in regione temerata regni Quitens, A. Humboldt and A. Bonpland 3010 (holotype: P (P00669401 [image!]); isotypes: BM (BM000938529 [image!], BM000938530 [image!]), HAL (HAL0106913 [image!]), P (P00740586 [image!], P00740587 [image!], P00740588 [image!])).

*= Vilfa fasciculata* Kunth, in Humb., Bonpl. & Kunth, Nov. Gen. Sp. (quarto ed.) 1: 139. 1815 [1816]*. Agrostis fasciculata* (Kunth) Roem. & Schult., Syst. Veg. (ed. 15 bis) 2: 362. 1817. Type: ECUADOR. Lulumbamba, in montibus Quitensium, A. Humboldt and A. Bonpland s.n. (holotype: P (P00669402 [image!]); isotypes: HAL (HAL0106915 [image!]), LE-TRIN (LE-TRIN-1610.01), P (P00740548 [image!], P00740549 [image!], US (US00156821)).

*= Trichodium muhlenbergianum* Schult., Mant. 2: 159. 1824. Type: USA. Pennsylvania, G. Muhlenberg s.n. (holotype: US (US00141875 [image!])).

*= Agrostis noveboracensis* Spreng., Syst. Veg. 1: 260. 1825 [1824]. *Trichodium noveboracense* (Spreng.) Schult., Mant. 3(Add. 1): 555. 1827. Type: USA. New York [“Nov. Eborae”]: sylvae, J. Torrey s.n*.* [not located].

*= Agrostis schiedeana* Trin., Mém. Acad. Imp. Sci. Saint-Pétersbourg, Sér. 6, Sci. Math., Seconde Pt. Sci. Nat. 4: 327. 1841. Type: MEXICO. Schrader s.n*.* (holotype: LE-TRIN (LE-TRIN-1651.01)).

*= Agrostis oreophila* Trin., Mém. Acad. Imp. Sci. Saint-Pétersbourg, Sér. 6, Sci. Math., Seconde Pt. Sci. Nat. 6,4(3-4): 323. 1841. *Agrostis hyemalis* (Walter) Britton, Sterns & Poggenb var. *oreophila* (Trin.) Farw., Rep. Michigan Acad. Sci. 6: 202. 1904. Type: USA Pennsylvania: Bethlehem, 1832, C.J. Moser s.n*.* (holotype: LE-TRIN (LE-TRIN-1633.01); isotypes: JE (JE00020198! [image!]), NY (NY327638 [image!]), US [fragm. ex LE-TRIN] (US00156469 [image!])).

*= Agrostis schweinitzii* Trin., Mém. Acad. Imp. Sci. Saint-Pétersbourg, Sér. 6, Sci. Math., Seconde Pt. Sci. Nat. 6,4(3-4): 311. 1841. Type: USA. Pennsylvania, L.D. Schweinitz s.n. (holotype: LE-TRIN (LE-TRIN-1653.01); isotype: US [fragm. ex LE-TRIN] (US00156492 [image!])).

*= Agrostis novae-angliae* Tuck., Mag. Hort. Bot. 9(4): 143. 1843. Type: USA New Hampshire: White Mountains, brooks and wet rocks in the Notch [not located].

= *Agrostis michauxii* Zuccagni var. *alpina* Rupr., Bull. Acad. Roy. Sci. Bruxelles 52: 228. 1842, nom. nud. Type. MEXICO. H.G. Galeotii 5767 (holotype: P (P00740583 [image!]).

*= Agrostis tenuifolia* Curtis var. *fretensis* Hook. f., Fl. Antarct. 2: 372. 1846. Type: CHILE. Port Famine, P.P. King s.n. (holotype: K (K000308391 [image!]); isotypes: BAA [fragm. ex K] (BAA00001369 [image!]), E00514140 [image!])).

*= Agrostis campyla* Tuck., Amer. J. Sci. Arts, ser. 2 6: 231. 1848. Type: USA. New England: in montibus, E. Tuckerman s.n. (holotype: not located; isotype: GH (GH00022971 [image!])).

*= Agrostis aberrans* Steud., Syn. Pl. Glumac. 1: 422. 1855 [1854]. Type: CHILE: prope Sandy Point, W. Lechler 1219 (holotype: S (S05-10083 [image!]; isotypes: BAA (BAA00001295 [image!], FR (FR0031117 [image!]), G (G00099218 [image!], G00099219 [image!]), GOET (GOET005577 [image!]), K (K000308399 [image!]), LE (LE00000641 [image!]), P (P00740589 [image!], P00740591 [image!], P00740590 [image!], P00740592 [image!]), US (US00156360 [image!]), W (W18890240128 [image!])).

*= Agrostis flavidula* Steud., Syn. Pl. Glumac. 1: 421. 1855 [1854]. Type: CHILE. Magallanes, prope Sandy Point, W. Lechler 1225 (holotype: P (P00740593 [image!]); isotypes: BAA (BAA00001336 [image!]), G (G00099213 [image!], G00099214 [image!]), K (K000308392 [image!]), LE (LE00000650 [image!]), P (P00740594 [image!]), S (S05-10058 [image!]), US [fragm. ex W] (US00156426 [image!])).

*= Agrostis violacea* Phil., Anales Univ. Chile 43: 560. 1873. Type: CHILE. Magallanes, cerca de Punta Arenas, 1865, R.A. Philippi s.n. (holotype: SGO (SGO000000069 [image!]); isotypes: BAA (BAA00001374 [image!]), JE (JE00003706 [image!]), SGO (SGO000000070 [image!]), W (W0025293 [image!], W18890096415 [image!], W19160040644 [image!])).

*= Agrostis perennans* (Walt.) Tuck var. *aestivalis* Vasey, Contr. U.S. Natl. Herb. 3(1): 76. 1892. Type: USA Illinois: Menard County, Athens, Sep 1864, E. Hall s.n. (holotype: US (US00131099 [image!])).

*= Agrostis intermedia* Scribn. ex Kearney, Bull. Torrey Bot. Club 20(12): 476. 1893, nom. illeg. hom., non Balb., 1801. *Agrostis pseudointermedia* Farw., Rep. (Annual) Commiss. Parks Boulevards Detroit 11: 46. 1900. *Agrostis scribneriana* Nash ex Small, Fl. S.E. U.S. 126. 1903, nom. illeg. superfl. Type: USA. Kentucky: Harlan County, near Harlan court house, 1893, T.H. Kearney 39 (syntypes: MO (MO-2151626 [image!]), OS (OS0000015 [image!]), US (US00131763 [image!])).

*= Agrostis kufium* Speg., Anales Mus. Nac. Buenos Aires 5: 82. 1896. Type: CHILE. Tierra del Fuego, Punta Arenas, 1882, C. Spegazzini 245 (holotype: LP (LP-14323 [image!]); isotypes: BAA (BAA00000019 [image!]), US [fragm. ex BAA] (US00156445 [image!])).

*= Agrostis exarata* Trin. var. *angustifolia* Hack., Svenska Exped. Magell. 3(5): 219. 1900. Type: CHILE. Tierra del Fuego, Río Grande, 25 Jan 1896, P. Dusen s.n. (holotype: W (W1916-0036331 [image!]); isotypes: US (US00156419 [image!])).

*= Agrostis canina* L. subsp. *grandiflora* Hack., Wiss. Erb. Schwed. Südpolar-Exp. 4(4): 5. 1906, nom. illeg. hom., non Druce, 1890. Type: ARGENTINA, Tierra del Fuego, Ushuaia, in silvis, C. Skottsberg Ser. I. 239 (holotype: S (S-R-165 [image!])).

*= Agrostis weberbaueri* Mez, Repert. Spec. Nov. Regni Veg. 18(1-3): 1. 1922. Type: PERU. Bei Huacaspistana und bei Monzon, Weberbauer s.n (holotype: B? [possibly destroyed]).

*= Agrostis perennans* (Walt.) Tuck var. *humilis* Farw., Pap. Michigan Acad. Sci. 1: 87. 1923. Type: USA. Michigan: Wayne County, Detroit, 29 Aug 1920, O.A. Farwell 5672 1/2 (holotype: BLH (BLH0000016 [image!]); isotype: MICH (MICH1108582 [image!]), US (US00131100 [image!])).

*= Agrostis perennans* (Walt.) Tuck fo. *atherophora* Fernald, Rhodora 35: 317. 1933. Type: CANADA. Quebec: Terrebonne County, island in Lac Tremblant, 1 Aug 1922, J.R. Churchill s.n. (holotype: GH (GH00023007 [image!]); isotype: MO (MO-123102 [image!])).

*= Agrostis perennans* (Walt.) Tuck fo. *chaetophora* Fernald, Rhodora 35: 317. 1933. Type: USA. Pennsylvania: Huntingdon County, Aug 1865, J.R. Lowrie s.n. (holotype: GH (GH00022972 [image!])).

***Agrostis scabra* Willd., Sp. Pl. 1(1): 370. 1797.**

*= Agrostis laxa* Muhl., Trans. Amer. Philos. Soc. 4: 236. 1799, nom .nud.

*= Trichodium montanum* Torr., Fl. N. Middle United States 84. 1823. *Agrostis laxiflora* (Michx.) Richardson var. *caespitosa* Torr., Fl. New York 2: 442. 1843. *Agrostis laxiflora* (Michx.) Richardson var. *montana* (Torr.) Tuck., Amer. J. Sci., ser. 4 45(1): 43. 1843. *Agrostis torreyi* Tuck., Mag. Hort. Bot. 9(4): 143. 1843, nom. illeg. hom., non Kunth 1833. *Agrostis scabra* Willd. var. *oreophila* Alph. Wood, Class-book Bot. (ed. 1861) 774. 1861, nom. illeg. superfl. *Agrostis scabra* Willd. var. *montana* (Torr.) Fernald, Proc. Portland Soc. Nat. Hist. 2: 91. 1895. *Agrostis peckii* House, Amer. Midl. Naturalist 7(4-5): 126. 1921. Type: USA. New York: on the summit of Fishkill Mountains, Anonymous s.n. (holotype: NY (NY431676 [image!])).

*= Trichodium album* J. Presl, Reliq. Haenk. 1(4-5): 244. 1830. *Agrostis nutkaensis* Kunth, Enum. Pl. 1: 222. 1833. *Agrostis nootkaensis* Trin., Mém. Acad. Imp. Sci. Saint-Pétersbourg, Sér. 6, Sci. Math., Seconde Pt. Sci. Nat. 6,4(3-4): 326. 1841, nom. illeg. superfl. *Agrostis hyemalis* (Walter) Britton, Sterns & Poggenb. var. *nutkaensis* (Kunth) Scribn. & Merr., Contr. U.S. Natl. Herb. 13(3): 56. 1910. Type: CANADA. British Columbia: hab. in sinu Nutkaensi, 1791, T. Haenke s.n. (holotype: PR; isotypes: BM (BM001042284 [image!]), HAL (HAL0133126[image!]), MO (MO-123101 [image!]), W (W0007120 [image!], W0007121[image!])).

*= Agrostis scabra* Willd. var. *tenuis* Tuck., Amer. J. Sci. Arts 45: 45. 1843. *Agrostis laxiflora* (Michx.) Richardson var. *tenuis* (Tuck.) Torr., Fl. New York 2: 442. 1843. *Agrostis hyemalis* (Walter) Britton, Sterns & Poggenb. var. *tenuis* (Tuck.) Gleason, Phytologia 4(1): 21. 1952. Type: USA. New Hampshire: Grafton County, Lincoln, rocks of the Flume [not located].

*= Agrostis scabriuscula* Buckley, Proc. Acad. Nat. Sci. Philadelphia 14: 90. 1862. *Agrostis scabrata* Nutt. ex A. Gray, Proc. Acad. Nat. Sci. Philadelphia 14: 334. 1862, pro syn. Type: USA. Oregon: Columbia Plains, T. Nutall s.n*.* (holotype: PH (PH00003821 [image!]); isotypes: BM (BM000578805 [image!]), US [fragm. ex PH] (US00729696 [image!])).

*= Agrostis hyemalis* (Walter) Britton, Sterns & Poggenb. var. *keweenawensis* Farw., Rep. Michigan Acad. Sci. 6: 203. 1904. Type: USA Michigan: Keweenaw County, Lake Superior, rocky shores within influence of the spray, 1 Jul 1895, O.A. Farwell 600c (holotype: BLH (BLH0000015 [image!]); isotype: BLH (BLH0000014 [image!])).

*= Agrostis geminata* Trin. fo. *exaristata* Fernald, Rhodora 35(414): 211. 1933. *Agrostis scabra* Willd. fo. *exaristata* (Fernald) Hultén, Acta Univ. Lund. 38: 156. 1942. *Agrostis hyemalis* (Walter) Britton, Sterns & Poggenb. fo. *exaristata* (Fernald) Scoggan, Fl. Canada 1: 51. 1978. Type: CANADA. Quebec: muddy border of small lake at about 530 m alt. back of North Fork Camp, North Fork of Madeleine River, 12 Aug 1923, M.L. Fernald, C.W Dodge and L.B. Smith 25485 (holotype: GH (GH00022964 [image!])).

*= Agrostis scabra* Willd. fo. *tuckermanii* Fernald, Rhodora 35(414): 207. 1933. *Agrostis hyemalis* (Walter) Britton, Sterns & Poggenb. fo. *tuckermanii* (Fernald) Scoggan, Fl. Canada 1: 51. 1978. Type: USA. Massachusetts: Sandy flats of Monatiquot River, above Union Street, Braintree, 1 Jul 1911, J.R. Churchill s.n*.* (holotype: GH (GH00022976 [image!]); isotypes: MO (MO-123104 [image!], MO-123105 [image!], MO-123106 [image!], MO-123107 [image!], MO-123108 [image!])).

*= Agrostis scabra* Willd. fo. *setigera* Fernald, Rhodora 35(414): 210. 1933. Type: CANADA: Newfoundland: wet moss and peat on the gneiss hills near Sand Bank, west of Burgco, 9 Sep 1926, M.L. Fernald, B. Long and J.M. Fogg 79 (holotype: GH (GH00022975 [image!])).

*= Agrostis scabra* Willd. var. *septentrionalis* Fernald, Rhodora 35(414): 209. 1933. *Agrostis scabra* Willd. subsp. *septentrionalis* (Fernald) Á. Löve & D. Löve, Bot. Not. 128(4): 504. 1975 [1976]. Type: CANADA. Newfoundland: dry serpentine slopes near Winterhouse Brook, Bonne Day, 8 Aug 1929, M.L. Fernald, B.H. Long and J.M. Fogg 1310 (holotype: GH (GH00022974 [image!])).

*= Agrostis scabra* Willd. var. *aristata* Hultén, Acta Univ. Lund. n.s. 38(1): 156. 1942. Type: USA Alaska: Valdez, 1 Auf 1935, J.P. Anderson 2861 (syntypes: GB (GB-0047749 [image!], GB-0047750 [image!]), GH (GH00023006 [image!]), K (K000838199 [image!])).

***Agrostis stolonifera* L., Sp. Pl. 1: 62. 1753.**

*= Agrostis maritima* Lam., Encycl. 1(1): 61. 1783. *Vilfa maritima* (Lam.) P. Beauv., Ess. Agrostogr. 16. 1812*. Milium maritimum* (Lam.) Clem. & Rubic, Ensay Vid. Andaluc. 285. 1807. *Agrostis alba* L. var. *maritima* (Lam.) G. Mey., Neues Hamburg. Mag. 1823: 138. 1824. *Agrostis stolonifera* L. var. *maritima* (Lam.) W.D.J. Koch, Syn. Deut. Schweiz. Fl. 781. 1837. *Agrostis alba* L. fo. *maritima* (Lam.) Parl., Fl. Ital. 1: 181. 1848. *Agrostis alba* L. subsp. *maritima* (Lam.) Arcang., Comp. Fl. Ital. 768. 1882. *Agrostis alba* L. *subsp*. maritima (Lam.) P. Fourn., Quatre Fl. France: 49. 1946, nom. illeg. hom. *Agrostis stolonifera* L. subsp. *maritima* (Lam.) Vasc., Pl. Areias e Rochedos Litor. (Fanerogam.) 39. 1974. *Agrostis stolonifera* L. var. *maritima* (Lam.) L. Vil'Yasoo, Fl. Estonsk. SSR 11: 251. 1979, nom. illeg. hom. Type: FRANCE. Environs de Narbonne, *P.A. Pourret s.n.* (holotype: P-LA; isotype: P-LA).

*= Agrostis flava* O.F. Müll., Fl. Dan.: 5. 1782. Type: DENMARK. Fridrichsdal [not located]

= *Agrostis aspera* Weber, Suppl. Fl. Holsat. 6: 4. 1787. Type: GERMANY. Rendsburg: lecta in uliginosis prope Wiek & Holtenau, ad Eidoram prope rendsburg, Scheuchz. 6 [not located].

*= Agrostis filifolia* Link, J. Bot. (Schrader) 2: 313. 1799. *Agrostis alba* L. subsp. *filifolia* (Link) Henriq., Bol. Soc. Brot. 20: 43. 1905. Type: PORTUGAL [not located].

*= Agrostis brevis* Knapp, Gram. Brit. pl. 116. 1804. Type: GREAT BRITAIN [not located].

= *Agrostis mutabilis* Knapp, Gram. Brit. pl. 28. 1804. Type: GREAT BRITAIN [not located].

*= Agrostis stolonifera* L. var. *dulcis* Pers., Syn. Pl. 1: 75. 1805. *Agrostis dulcis* (Pers.) Sibth. ex Kunth, Enum. Pl. 1: 218. 1833. Type: EUROPE [not located].

*= Agrostis patula* Gaudin, Alpina 3: 14. 1808. *Agrostis alba* L. var. *patula* (Gaudin) Gaudin, Fl. Helv. 1: 188. 1828. *Agrostis stolonifera* L. var. *patula* (Gaudin) Rchb., Fl. Germ. Excurs. 26. 1830. *Agrostis alba* L. subsp. *patula* (Gaudin) Arcang., Comp. Fl. Ital.: 768. 1882. *Agrostis stolonifera* L. fo. *patula* (Gaudin) Beldie, Fl. Republ. Socialiste Romania 12: 151. 1972. Type: not located.

*= Agrostis decumbens* Hall. f. ex Gaudin, Agrost. Helv. 1: 78. 1811, nom. illeg. hom., non Host 1809. *Agrostis alba* L. subvar. *decumbens* (Gaudin) Meyer, Hannover. Mag. 1823: 134. 1824. *Agrostis alba* L. var. *decumbens* Gaudin, Fl. Helv. 1: 187. 1828. *Agrostis alba* L. subsp. *decumbens* (Gaudin) Arcang., Comp. Fl. Ital.: 768. 1882. *Agrostis stolonifera* L. fo. *decumbens* (Gaudin) Soó, Acta Bot. Acad. Sci. Hung. 17(1-2): 122. 1971[1972]. Type: SWITZERLAND. *Anonymous s.n.* (lectotype, designated by Hackel and Briquet (1907: 37): G).

*= Agrostis stolonifera* L. var. *latifolia* G. Sinclair, Hort. Gram. Woburn. 112. 1816. Type: not designated.

*= Agrostis ambigua* Roem. & Schult., Syst. Veg. (ed. 15 bis) 2: 352. 1817. Type: EUROPE. In littorali ad mare et aliis in locis [not located].

*= Agrostis straminea* Hartm., Gen. Gram. Scand. 4. 1819. *Agrostis stolonifera* L. var. *straminea* (Hartm.) Hartm., Svensk och Norsk Exc. Fl. (ed. 4): 14. 1866. *Agrostis alba* L. var. *straminea* (Hartm.) K. Richt., Pl. Eur. 1: 43. 1890. *Agrostis stolonifera* L. subsp. *straminea* (Hartm.) Tzvelev, Novosti Sist. Vyssh. Rast. 5: 58. 1971. Type: SWEDEN. Hab. ad littorae marina Hallandiae, C.J. Hartman s.n. (holotype: UPS).

= *Vilfa glaucescens* C. Presl, Cyper. Gramin. Sicul.: 23. 1820. *Agrostis glaucescens* (C. Presl) Spreng., Syst. Veg. [Sprengel] 1: 258. 1825[1824]. *Agrostis sicula* Kunth, Révis. Gramin. 1: 71. 1829, nom. inval., pro syn. *Agrostis alba* L. var. *glaucescens* (C. Presl) K. Richt., Pl. Eur. 1: 43. 1890. Type: ITALY. Hab. in humidis maritimis Pondascheddi inter et Trabiam [not located].

= *Agrostis bryoides* Dumort., Fl. Belg. 152. 1827. Type: BELGIUM [not located].

*= Agrostis stolonizans* Besser ex Schult. & Schult. f., Mant. 3(add. 1): 567. 1827. *Agrostis* *stolonifera* L. subsp. *stolonizans* (Besser ex Schult. & Schult. f.) Soó, Acta Bot. Acad. Sci. Hung. 23(3-4): 391. 1977[1978]. Type: UKRAINE. Volhynia, W. Besser s.n. (lectotype, designated by Widén (1971: 78): H).

*= Agrostis alba* L. var. *decumbens* Eaton & Wright, Man. Bot. (ed. 5): 98. 1829, non Gaudin, 1828. Type: USA. Pennsylvania [not located].

*= Agrostis caespitosa* Gaudich., Voy. Uranie: 407. 1830, nom. illeg. hom, non Salisbury 1796. *Vilfa gaudichaudii* Steud., Nomencl. Bot. (ed. 2) 2: 767. 1841. Type: UNITED KINGDON. Falkland Islands, in insulis Maclovianis [not located].

*= Agrostis albida* Trin., Mém. Acad. Imp. Sci. Saint-Pétersbourg, Sér. 6, Sci. Math., Seconde Pt. Sci. Nat. 6,4(3-4): 344. 1841. *Agrostis alba* L. var. *albida* (Trin.) Griseb., Fl. Ross. [Ledebour] 4(13): 437. 1852. *Agrostis stolonifera* L. subsp. *albida* (Trin.) Tzvelev, Novosti Sist. Vyssh. Rast. 8: 58. 1971. Type: RUSSIA [not located].

= *Agrostis stolonifera* L var. *compacta* Hartm., Handb. Skand. Fl. (ed. 8): 24. 1843. Type: SWEDEN. Göteborg, *G. Wahlenberg s.n.* (lectotype, designated by Widén (1971: 78): UPS).

= *Agrostis stolonifera* L. var. *prorepens* W.D.J. Koch, Syn. Fl. Germ. Helv. 902. 1843. *Agrostis alba* L. var. *prorepens* (W.D.J. Koch) G. Meyer ex Asch., Fl. Brandenburg 1: 819. 1864. *Agrostis prorepens* (W.D.J. Koch) G. Mey. ex Asch., Fl. Brandenburg 1: 819. 1864. *Agrostis prorepens* (W.D.J. Koch) Rouy, Fl. France 14: 61. 1913, nom. illeg. hom. Type: EUROPE. W.F.R. Suringar s.n. (lectoype, designated by Widén (1971: 78): L (L0043566 [image!]).

= *Agrostis prostrata* Hook. f., Fl. Antarct. 2: 373. 1846. Type: UNITED KINGDOM. Falkland Islands, boggy ground on Hog Island, Berkeley Sound, J.D. Hooker s.n. (holotype: K; isotypes: BAA (BAA00001363 [image!], BAA00001364[image!]), BM (BM000938542 [image!]), MPU (MPU027086 [image!]), P (P00740424 [image!]), US (US00156482 [image!])).

= *Agrostis sinaica* Boiss., Diagn. Pl. Orient., ser. 1, 13: 46. 1854. Type: Hab. ad aquas jugi Sinaitici Bove, pl. exs. et in Decaisne Fl. Sin. no. 44 sub A. maritima (syntypes: not located).

= *Agrostis aristulata* Müll. Hal., Bot. Zeitung (Berlin) 14(20): 349. 1856. Type: UNITED KINGDON. Falkland Islands, Patriae Insulae Falkland, J.D. Hooker s.n. [not located].

= *Agrostis nemoralis* Phil., Linnaea 30(2): 205. 1859. Type: CHILE.  in nemoribus prope Puerto Montt crescit in nemoribus prope Puerto Montt crescit, R.A. Philippi 162 (holotype: SGO; isotype: US (00156466 [image!]).

= *Agrostis adscendens* Lange, Naturhist. For. Kjobenhavn. Vid. Medd. II 1: 33. 1860. Type: SPAIN [not located].

*= Agrostis depressa* Vasey, Bull. Torrey Bot. Club 13: 54. 1886. Type: USA. Colorado: damp places along Clear Creek, 1885, H.N. Patterson s.n. (syntypes: US (US00131739 [image!]), GH (GH00022961[image!])); Clear Creek Canyon, near Georgetown, 1885, H.N. Patterson 46 (syntype: US (US00131738 [image!])).

*= Agrostis exarata* Trin. var. *stolonifera* Vasey, Bull. Torrey Bot. Club 13: 54. 1886. Type: USA. Washington: bottom lands of the Columbia River, 1833, W.N. Suksdorf 40 (holotype: US (US00131742 [image!]); isotype [possible]: MO (MO-123103 [image!])).

*= Agrostis eliasii* Sennen, Bol. Soc. Iber. Ci. Nat. 1907. 1907, nom. nud.

*= Agrostis karsensis* Litv., Sched. Herb. Fl. Ross. 8: 147. 1917. *Agrostis stolonifera* L. subsp. *karsensis* (Litv.) Valdés & H. Scholz, Willdenowia 36(2): 662. 2006. Type: TURKEY. Kars: marshy meadow aldong Baiburt River near the village Kachmez along the highway Between Sarykamysh and Karb, 27 Jun 1914, D. Litvinov 2692 (syntype: LE (LE00009332 [image!]).

= *Agrostis reptans* Rydb., Fl. Rocky Mts. 54. 1917. Type: USA [not located].

*= Agrostis sibirica* Petrov, Fl. Iakut. 1: 175. 1930. Type: RUSSIA. Yakutia: environs of Olekminks, Olenin s.n. (holotype: LE).

= *Agrostis stolonifera* L. fo. *aristigera* Fernald, Rhodora 35: 317. 1933. *Agrostis alba* L. fo. *aristigera* (Fernald) Fernald, Rhodora 51(609): 192. 1949. Type: USA. Massachusetts: Granville, along brook in meadow, 27 Jun 1914, F.C. Seymour 309 (holotype: GH (GH00023003 [image!])).

*= Agrostis jacutica* Schischk., Fl. SSSR 2: 179. 1934. Type: RUSSIA. Inter Jakutsk et Vilujstk, Lacus prope stationem Byrylackskaja in trunco putrido natante, 3 Aug 1912, R. Abolin 759 (holotype: LE (LE0101094 [image!]); isotype: LE (LE01010946 [image!]).

= *Agrostis macrantha* Schischk., Fl. SSSR 2: 177. 1934. Type: Caucasus, inter sinum maris Nigri Gelendzhik et opp. Gelendzhik, 21 Jul 1912, I. Palibin s.n. (holotype: LE).

= *Agrostis pseudoalba* Klokov, Bot. Mater. Gerb. Bot. Inst. Komarova Akad. Nauk SSSR 12: 38. 1950. Type: UKRAINE. Kiev: Shevchenko region, between the villages Usovka and Chereki along the bank of Supy swamp, 11 Jul 1928, D. Zerovs and K. Zerov s.n. (holotype: KW).

= *Agrostis zerovii* Klokov, Bot. Mater. Gerb. Bot. Inst. Komarova Akad. Nauk SSSR 12: 39. 1950. Type: UKRAINE. Kiev: Nezhinsk, Veprik, 18 Jul 1928, D. Zerov and K. Zerov s.n. (holotype: LE (LE01078575 [image!]).

***Agrostis tolucensis* Kunth, in Humb., Bonpl. & Kunth, Nov. Gen. Sp. 1: 135. 1815.**

*= Vilfa glomerata* J. Presl, in C. Presl, Reliq. Haenk. 1(4-5): 239. 1830. *Agrostis glomerata* (J. Presl) Kunth, Enum. Pl. 1: 219. 1833. Type: PERU. Hab. in montanis Peruviae huanoccensibus, 1791, T. Haenke s.n. (holotype: PR?; isotypes: HAL (HAL0106916 [image!]), PRC (PRC450953 [image!]), US [fragm.] (US00589472 [image!]), W (W0025326 [image!])).

*= Agrostis nana* (J.Presl) Kunth var. *aristata* Griseb., Abh. Königl. Ges. Wiss. Göttingen 24: 294. 1879. Type: ARGENTINA. Salta: Nevado del Castillo, Mar 1873, P.G. Lorentz and G. Hieronymous 82 (holotype: GOET (GOET006541 [image!]); isotypes: BAA (BAA00000723 [image!], BAA00000724 [image!]), CORD (CORD00004693 [image!]).

*= Agrostis nana* (J.Presl) Kunth var. *andicola* Pilg., Bot. Jahrb. Syst. 37: 505. 1906. *Agrostis tolucensis* Kunth var. *andicola* (Pilg.) Rúgolo & A.M. Molina, Parodiana 8(2): 142. 1993. Type: ECUADOR. Chimborazo: monte Chimborazo, in regione paramo dicta, Jun 1903, H. Meyer 145 (syntypes: JE (JE00020225 [image!], JE00020227 [image!]), US (US00156464 [image!]), 146 (syntypes: JE (JE00020226 [image!], JE00020228 [image!]).

*= Agrostis hoffmanii* Mez, Repert. Spec. Nov. Regni Veg. 18: 3. 1922. Type: COSTA RICA. Irazú, C. Hoffman s.n. (holotype: B? [possibly destroyed]).

**References**

Hackel E, Briquet J (1907) Revision des graminées de L’Herbier d’Albr. de Haller filius. Annuaire du Conservatoire et du jardin botaniques de Genève 10: 26–98.

Philipson WR (1935) Abnormal spikelets in the genus *Agrostis*. The Journal of botany, British and foreign 73: 65–75.

Widén KG (1971) The genus *Agrostis* L. in eastern Fennoscandia. Taxonomy and distribution. Flora Fennica 5: 1–209. <http://hdl.handle.net/10138/36219>
