# Supplementary material for: ﻿Revision of the genus Agrostis (Poaceae, Pooideae, Poeae) in Megamexico
Source: PhytoKeys. 2023 Aug 11;230:157–256. doi: 10.3897/phytokeys.230.105878 (PMC10439499; doi:10.3897/phytokeys.230.105878)
Supplement: Supplementary material 2 — Additional specimens examined [file phytokeys-230-157_article-105878__-s002.docx]

**Supplementary Material 2. Additional specimens examined**

***Agrostis bourgaei***

**MEXICO. Hidalgo**: **Municipio Mineral del Chico**, alrededores de Las Ventanas, 5 km al N de Pachuca, [20.168309N, 98.73611W], 2900 m alt., 2 Nov 1983, S. Acosta and M. Medina 413a (HUMO); Laguna La Estanzuela, [20.16690456N, 98.75430133W], 3200 m alt., 5 Aug 1978, C.L. Díaz 9638 (MEXU). **Mexico: Municipio Amanalco de Becerra**, Amanalco, [19.25121538N, 100.0124057W], 2368 m alt., 3 Dec 1993, A. Bolaños s.n. (MEXU). **Municipio Amecameca**, 5 km al W de Paso de Cortés, vertiente W del Popocatépetl, [19.08873853N, 98.67164797W], 3500 m alt., 9 Oct 1966, J. Rzedowski 23267 (MEXU). **Municipio Chimalhuacán**, en orilla de laguna, 2300 m alt., 22 Oct 1950, E. Matuda 18548 (US). **Municipio Huixquilican**, puerto El Guarda, 6 km al W de San Francisco Chimalpa, [19.409425N, 99.380795W], 3100 m alt., 2 Oct 1969, A.M. Pérez 87 (IBUG). **Municipio Ixtapaluca**, Estación Experimental de Investigación y Enseñanza de Zoquiapan, S of Río Frío, [19.280108N, 98.671828W,] 3250 m alt., 11 Jul 1975 S.D. Koch 75328 (US). **Municipio Jilotzingo**, 3 km al NW de San Luis Ayucan sobre la carretera a Santa Ana Jilotzingo, [19.49613283, 99.3491131W], 2850 m alt., 29 Oct 1978, J. Rzedowski 35955 (INEGI). **Municipio Lerma**, San Lázaro, [19.825N, 99.1325W], 1 Jul 1918, E. Lyonnet 269 (MEXU, US). **Municipio San Felipe del Progreso**, carretera San Felipe del Progreso−Carmona, 3 km sobre la desviación a El Guardia de Guadalupe, ranchos La Rosa y La Rosa de la Palma, 15 Jun 1990, A. Miranda 567 (MEXU). **Municipio Temascaltepec**, frente a camino en construcción de Cieneguillas de Labra, por la carretera Sultepec−La Puerta, [18.991715N, 99.911636W], 2600 m alt., 1 Aug 1981, R. Guzmán 4025 (MEXU); Tequesquipan, [19.056389N, 99.944719W, 2480 m alt.], 18 Aug 1932, G.B. Hinton 1338 (TEX); 1 km después de Cajones, rumbo a Sultepec, [19.04436944N, 99.875W, 2960 m alt.], 7 Feb 1984, E. Manrique et al. 656 (MEXU). **Municipio Tlalmanalco**, La Ciénega, región de Peñas Cuatas, ladera W del Iztaccíhuatl [19.668889N, 99.328611W], 3600 m alt., 19 Aug 1984, S. Acosta 691 (MEXU, UAMIZ), 692 (UAMIZ); La Ciénega, región de la cabeza del Iztaccíhuatl, [19.2N, 98.8W], 3600 m alt., 18 Jul 1982, J. Rzedowski 37853 (IEB, XAL). **Municipio Villa de Allende**, criadero San Cayetano, 4 km al N de Agua Escondida, [19.39327778N, 100.0760083W], 2500 m alt., 22 Aug 1982, M.J. Díaz 263 (MEXU). **Municipio Villa de Allende**, Villa de Allende, 19.37618121N, 100.1489403W], 2440 m alt., 5 Oct 1952, E. Matuda 26816 (MEXU). **Municipio Villa del Carbón**, cerro la Bufa, [19.66649556N, 99.55298776W], 2900−3500 m alt., E. Matuda 29738 (MEXU, US). **Without municipality,** at slow running stream at the edge of a marsh, 20 mi E of Toluca, 10 Aug 1947, F.A. Barkley et al. 606 (MEXU); 12.5 mi from turnoff to the summit of Popocatepetl, 3460 m alt., 18 Oct 1976, J. Brunken and C. Perino 419 (MEXU); entre Las Juntas y Texcaltitlán, al S del Nevado de Toluca, 1 Aug 1981, R. Guzmán 4013 (MEXU); Rancho Buenavista y Libertad, 2520 m alt., 8 Aug 1981, R. Guzmán 4231 (MEXU); criadero de trucha , 3000 m alt., 3 Sep 1950, E. Matuda 19394 (MEXU, US); Sierra de las Cruces, 10 Jul 1982, C.G. Pringle 5199 (MEXU). **Mexico City: Alcaldía Álvaro Obregón**, Lomas de Mixcoac, [19.370556N, 99.210278W, 2300 m alt.], 13 Aug 1938, E. Lyonnet 2266 (MEXU); Santa Rosa, [19.32360833N, 99.28045W], 2700 m alt., 18 Aug 1977, A. Ventura 3004 (CIIDIR, MEXU, XAL). **Alcaldía Cuajimlapa**, Puerto de las Cruces, [19.28971944N, 99.34563889W], 3100 m alt., 18 Sep 1983, Y. Herrera 288 (CIIDIR, IEB, MEXU, TEX); Desierto de los Leones, [19.31250833N, 99.30847778W, 2900 m alt.], 1 Jan 1938, E. Lyonnet 2038 (MEXU, US), 2600 (MEXU, US), 2721 (US); alrededores del Llano de la Cieneguilla, arriba del Desierto de los Leones, [19.24694444N, 99.33444444W], 3400 m alt., 5 Sep 1979, J. Rzedowski 36364 (CIIDIR). **Alcaldía Magdalena Contreras**, Cuarto Dinamo, ca. 250 m subiendo por el río, 19.26395N, 99.29516W, 3103 m alt., 3 Sep 2022, L. Vigosa 115 (MEXU). **Michoacán:** **Municipio Angangueo**, Sierra Chincua, 19.67805556N, 100.2744444W, 2910 m alt., 28 Jul 2005, M.G. Cornejo and G. Ibarra 1267 (MEXU); Encino Gordo, Sierra Chincua, 2930 m alt., 13 Jan 1988, M. Mejía s.n. (IEB). **Municipio Charo**, between the río del Salto and La Polvilla, ca 18 mi E of Morelia, [19.65N, 100.95W, 2438 m alt.], Nov 1961, R.M. King and T.R. Soderstrom 5106 (US). **Municipio Morelia**, cerca de la cortina de la presa Cointzio, [19.60850833N, 101.2547889W], 2100 m alt., 15 Oct 1986, J. Rzedowski 41001 (IBUG, IEB). **Municipio Queréndaro**, Parque Garnica, ca. 29 km E of Morelia along highway 15 to Toluca, [19.71337939N, 100.7886386W], 2800 m alt., 19 Aug 1975, G. Davidse and J. Davidse 9871 (MEXU); presa Malpaís, afueras de Queréndaro, [19.81802778N, 100.8531778W], 1861 m alt., 11 Aug 2000, A. Novelo y L. Ramos 4056 (IEB, MO); 3 km al N de San José Lagunillas, sobre el camino a Milpillas, [19.69740833N, 100.8752083W], 2750 m alt., J. Rzedowski 40484 (IBUG, IEB); El Salto, 2 km al S de San Miguel de las Cuevas, [19.74185833N, 100.8085694W], 2330 m alt., S. Zamudio and C. Medina 14392 (IEB, XAL). **Municipio Zinapécuaro**, 5 km de Pueblo Viejo, 19.7575N, 100.7855556W, 2330 m alt., 17 Jun 1995, L. Aragón 305 (MEXU). **Morelos:** **Municipio Huitzilac**, Parque Nacional Lagunas de Zempoala, Laguna Tonatiahua, [19.05529062N, 99.31609205], 3200 m alt., 20 Aug 1986, P.R. Matosic et al. 1274 (MEXU); upper end of lake 1, Lagunas de Zempoala National Park, 28 Jul 1949, C.M. Rowell 2028 (US). **Puebla: Municipio San Nicolás de los Ranchos**, declive E del Paso de Cortés, 3.5 km (5 km por la brecha) al NE del entroque de la carretera a Amecameca y el camino entre Popocatépetl e Iztaccíhuatl, 15 km al ESE de Amecameca, [19.0667N, 98.4833W], 3600 m alt., 6 Dec 1976, S.D. Koch 76313 (CHAPA, US).

***Agrostis exarata***

**MEXICO. Baja California.** **Municipio Ensenada**, Sierra San Pedro Mártir, seep along road to Observatory, between Observatory and road to La Tassajera, 31.03555N, 115.46861W, 2546 m alt., 31 Jul 2005, S.J. De Groot and R. Lauri 5159 (BRY); Baja California, Sierra San Pedro Mártir, just outside National Park entrance, ca. 20 mi E of Meling Ranch, [30.831617N, 115.556858W], 1000 m alt., 22 Aug 1972, L. McGill 39 (ASU); Sierra San Pedro Mártir, arroyo 2 km SE of Yerbabuena, [31.004167N, 115.45W], 2450 m alt., 4 Sep 1978, R. Moran 26241 (DES, MSC); Sierra San Pedro Mártir, in arroyo, Jeffrey Pine forest, Yerba Buena, [31.01292N, 115.48002W], 2475 m alt., 16 Aug 1967, R. Moran and R.F. Thorne 14158 (SD); Sierra San Pedro Mártir, occasional by small stream, Jeffrey Pine forest, Yerba Buena, [31.01292N, 115.48002W], 2550 m alt., 16 Aug 1967, R. Moran and R.F. Thorne 14218 (SD); Sierra San Pedro Mártir, Los Llanitos, [30.96667N, 115.43333W], 2400 m alt., 17 Aug 1967, R. Moran and R.F. Thorne 14251 (SD); Sierra San Pedro Mártir, cypress canyon near end of upper Vallecitos road at Los Llanitos, ca. 6.4 mi SE from Observatory road at Vallecitos near SW base of cerros la Botella Azul, [31.03333333N, 115.4583333W], 2300 m alt., 19 Jul 1988, R.F. Thorne et al. 63486 (ARIZ); Parque Nacional Sierra San Pedro Mártir, Vallecitos, [31.03333333N, 115.4583333W], 2430 m alt., 1 Sep 1985, R.F. Thorne et al. 61432 (MEXU), 61433 (US). **Municipio San Felipe**, Canyon del Diablo, to the north and west of Picacho del Diablo (cerro La Encantada), E flank of Sierra San Pedro Mártir, [31.083333N, 115.383333W, 1600 m alt.], 16 Jun 1954, K. Chambers 557 (UC), 616 (UC). **Without municipality**, Tia Juana [Tijuana?], Jun 1895, S.G. Stokes (SD). **Chihuahua: Municipio Casas Grandes**, in the Tinaja, about 20 miles from the home of Herman Hatch in Colonia Juárez, [30.35991519N, 108.0890618W, 1580 m alt.], 6 Jun 1965, G. Moore 527 (BRY); at Pacheco, on road to Keith Bowman's ranch and at the ranch, 30.08707N, 108.34424W, 1900 m alt., 11 Jul 1997, J. Spencer and D. Atwood 687 (BRY). **Municipio Guachochi**, Rejogochic, N of Humira and the gorge of the Barranca del Cobre, S of Creel, [27.415857N, 107.483091W, 1870 m alt.], 1 Nov 1975, R. Bye 5728 (ASU, COLO); SW of Cusarare at brink of Cascada de Cusarare, [27.625N, 107.541667W], 1075 m alt., 22 Jul 1973, R.A. Bye 4317b (COLO, MEXU). **Municipio Janos**, 1 mi S of Los Azules, in Sierra La Brena on the Sierra Madre Occidental, 30.21766N, 108.52998W, 2039 m alt., 26 Sep 1998, J. Spencer and D. Atwood 1301 (BRY). **Municipio Ocampo**, Cascada de Basaseachic, río Basaseachic, 28.175N, 108.2111111W, 1800 m alt., 4 Jun 1999, T. Van Devender et al. 99-218 (ASC). **Durango: Municipio Canelas**, 28.5 km de Canelas por el camino a Durango, [25.114722N, 106.5W], 1325 m alt., 6 Oct 1985, S. González 3459 (IEB, MEXU, CIIDIR); Cuevecillas, 1.8 km al E del Entronque Topia-Canelas, por el camino a Durango, [25.131944N, 106.449722W], 2600 m alt., 6 Oct 1985, S. González et al. 3476 (CIIDIR). **Municipio San Dimas**, Vencedores, paraje El Pantano, al SO del poblado, 24.42222222N, 105.7144444W, 2460 m alt., 24 Aug 2013, S. Heynes et al. 557 (CIIDIR); state of Durango, P.I. Garcia 378 (US). **Guanajuato:** **Municipio San Felipe**, Vergel de la Sierra, [21.38504722N, 101.6354389W], 2440 m alt., 3 Sep 1981, A.A. Beetle M-7402 (MICH), R. Santillán 154-R, (MEXU). **USA. Arizona:** **Cochise County**, Huachuca Mountains, Carr Canyon, near end of road, [31.429832N, 110.304438W], 1920 m alt., 12 Aug 1947, F.W. Gould and L.E. Gillogly 4398 (F, NY); Turkey Creek below highway 181, ca. 50 km (by air) ESE of Willcox, Sulphur Springs Valley, 31.87198333N, 109.4914167W, 1472 m alt., 8 May 2016, C.M. Roll 2054 (ARIZ). **Pima County**, Saguaro National Park, Rincon Mountain District, Rincon Mountains. Near pond at Manning Camp, [32.208333N, 110.55W], 2438 m alt., 26 Jun 1982, J.E. Bowers and S.P. McLaughlin R-296 (ARIZ); Sabino Canyon, Santa Catalina Mountains, [32.3302N, 110.795W], 2000 m alt., 2 May 1903, J.J. Thornber 492 (ASU). **California: San Bernardino County**, San Bernardino National Forest, San Gorgonio Wilderness, headwaters of the Whitewater watershed, head of the North Fork of Whitewater River, collecting at North Fork Meadows, San Gorgonio Mountain, 34.10182N, 116.80157W, 2590 m alt., 6 Jul 2016, D.S. Bell and A. Chambers 9893 (RSA). **San Diego County**, along Joaquapin Creek, 200 m NE of Dyar Spring Trail junction, Cuyamaca Rancho State Park, [32.92084N, 116.54563W], 1450 m alt., 26 Jul 1985, M. Curto 65 (SD); along the Sweetwater River through Green Valley, especially near Juaquapin Creek junction, Cuyamaca Rancho State Park, [32.91356N, 116.5695W], 1220 m alt., 9 Jul 1986, M. Curto 343 (SD); near Terry's Lodge, Laguna Mountains, [32.867N, 116.44169W, 1740 m alt.], 31 Aug 1938, F.F. Gander 6430 (SD); Hot Springs Mountain area, in clearing at edge of road 200 m northwest of gate at boundary on Los Coyotes Indian Reservation, road to Lost Valley, [33.314367N, 116.578603W, 1900 m alt.], 16 Jun 2000, L. Hendrickson 318 (BSCA); Cuyamaca Rancho State Park, Green Valley east of state highway 78 and access road to historic Dyer house, Sweetwater River near confluence with Cold Stream, 32.92534N, 116.55899W, 1232 m alt., 6 Oct 2005, L. Hendrickson 1180 (SD); Cuyamaca Rancho State Park, Cuyamaca Peak, 1 mi E of highway 79, beside Fern Flat Fire road (unpaved), 0.5 mile SE of intersection with Lookout Fire road and 0.8 air mile ENE of summit, 32.9487N, 116.5935W, 1586 m alt., 18 Sep 2008, L. Hendrickson 3276 (BSCA, SD); Cuyamaca Rancho State Park, Green Valley, S-79 at Park Headquarters, County School Camp, E on Upper Green Valley Fire Road past campus to gate, then ca. 1 mile north on UGVFRd., 32.9423N, 116.5499W, 1234 m alt., 1 Jul 2006, L. Lee and L. Hendrickson 149 (SD); Laguna Mountais, Lucky 5 Ranch, southern portion of new acquisition by California State Parks, W side of Sunrise highway (S-1) between Cuyamaca Lake and Mount Laguna, 32.93222N, 116.49028W, 1624 m alt., 15 Jul 2003, J.P. Rebman et al. 9373 (BSCA, SD); Cuyamaca Rancho State Park, along Azalea Spring Fire road between Middle Peak and Cuyamaca Peak, vicinity of Azalea Spring, 32.96054N, 116.59978W, 1625 m alt., 16 Jun 2009, J.P. Rebman et al. 18144 (BSCA, SD); Cleveland National Forest, vicinity of Buckman Springs, NE of Morena Reservoir, S of Morena Stokes Valley, Corral Canyon road, and W of Buckman Springs road, 32.70482N, 116.50647W, 930 m alt., 26 Jun 2012, J.P. Rebman et al. 25033 (SD); Cuyamaca State Park, near the Cold Stream Trail, E of Hwy 79, about 1/4 mile SE of the West Mesa parking area, 1.5 mi SE of park headquarters, 32.93712N, 116.56237W, 1277 m alt., 16 Sep 2005, M. Sweet y B. Bruce 207 (BSCA). **New Mexico:** **Luna County**, Windmill Canyon, [32.155513N, 107.595391W], 2438 m alt., 2 Oct 1994, E.H. Roalson and K.W. Allred 1201 (BRY). **Texas:** **Jeff Davis County**, Davis Mountains, Little Aguja Canyon, [30.920424N, 103.82685W], 1520 m alt., 12 Jun 1931, J.A. Moore and J.A. Steyermark 3069 (MICH, NY); Davis Mountains, Little Aguja Canyon, [30.808174N, 103.909122, 1520 m alt.], 12 Jun 1928, E.J. Palmer 34496 (NY); Livermore Ranch, ca. 15 air miles E of State Highway 166 via dirt ranch road and ca. 18 air miles W of Fort Davis, S end of Davis Mountains, 30.61892N, 104.18906W, 1950 m alt., 24 Jul 2016, G. Yatskievych et al. 16-224 (TEX), 16-232a (TEX).

***Agrostis ghiesbreghtii***

**GUATEMALA. Sacatepéquez:** **Distrito Santa María de Jesús**, Volcán de Agua, [14.468149N, 90.736509W], 3200 m alt., 24 Aug 2000, M. Véliz et al. 10298 (MEXU). **MEXICO. Chiapas:** **Municipio Motozintla**, NW slope of cerro Mozotlan, below the microwave tower along the road from Huixtla to El Porvenir and Siltepec, [15.42605394N, 92.34362676W], 3000 m alt., 15 Oct 1980, D.E. Breedlove 46272 (MEXU [*]). **Guerrero:** **Muncipio Atoyac de Álvarez**, 6 km después de Puerto del Gallo hacia cerro Teotepec, [17.46472222N, 100.1527778W], 2700 m alt., 12 Dec 1998, E. Velázquez 1859 (MEXU). **Muncipio Coyuca de Benítez**, cerro Teotepec Chico [cerro Tlacotpec], [17.38330556N, 100.0833306W], 2860 m alt., 1 Oct 1992, F. Lorea 2244 (FCME, MEXU); El Parteaguas, 8 km despúes de la desviación a Pueblo Viejo, hacia Puerto del Gallo, [17.45921944N, 100.1336389W], 3090 m alt., 9 Dec 1998, E. Velázquez 1769 (MEXU). **Municipio General Heliodoro Castillo**, cerro Teotepec, ladera N, [17.46666667N, 100.2166667W], 3000 m alt., 27 Jan 1993, N. Diego 6730 (MEXU); Puerto Unión, ladera del cerro Teotepec, [17.45130833N, 100.0585778W], 3030 m alt., 31 Oct 1998, N. Diego 8204 (MEXU); vertiente SO del cerro Teotepec, [17.48333333N, 100.2W], 3400 m alt., 29 Jan 1965, J. Rzedowski and R. McVaugh 209 (ENCB); cerro Teotepec, [17.46666667N, 100.2166667W], 3350 m alt., 11 Apr 1963, J. Rzedowski 16505 (ENCB, MSC), 18167 (F, MICH, US). **Hidalgo:** **Municipio Mineral del Chico**, El Chico, Aug 1928, E. Lyonnet 46 (MEXU), 2223 (FCME, MEXU, US); 3 km al S de El Chico, 2800 m alt., 11 Jul 1988, M. Medina and M.A. Barrios 3619 (IBUG); cerro de Las Ventanas, [20.18539N, 98.73468W], 3050 m alt., 21 Aug 1983, J. Rzedowski 38184 (IEB, XAL). **Municipio Real del Monte**, parte alta del cerro Zumate, cerca de Pueblo Nuevo, [20.16803515N, 98.66675269W], 3000 m alt., 21 Aug 1983, J. Rzedowski 38182 (IEB, MEXU). **Mexico: Municipio Ixtapaluca**, Río Frío, camino a Puebla, [19.3547153N, 98.66863414W, 3000 m alt.], Sep 1927, E. Lyonnet 67 (MEXU, US). **Municipio Ocuilán,** Laguna Tonatihua, [19.05529062N, 99.31609205W], 2800 m alt., 6 Oct 1987, J. Castañeda 642 (MEXU); Lagunas de Zempoala, [19.05034984N, 99.31696647W], 2890 m alt., 31 Oct 1987, J. Castañeda 860 (MEXU). **Municipio Temascaltepec**, carretera Toluca-Temascaltepec, 0.5 km de la desviación a Peñuelas, [19.17470462N, 99.85592092W], 3108 m alt., 3 Apr 1981, R. Guzmán and L. Rico 1185 (MEXU); Mesón Viejo, [19.16666667N, 99.88333333W, 2784 m alt.], 29 Nov 1932, G. Hinton et al. 2734 (GBH, NY, US), 2742 (US). **Municipio Zinacantepec**, Crucero, [19.13333333N, 99.83333333W, 3359 m alt.], 17 Feb 1934, G. Hinton et al. 5590 (MEXU, NY, US), 8393 (NY); desviación a la Peñuela, carretera Toluca-Temascaltepec, [19.17604226N, 99.85107148W], 3120 m alt., 27 Oct 1992, A. Vega et al. 192 (MEXU). **Morelos:** **Municipio Huitzilac**, Parque Nacional Lagunas de Zempoala, cerca de la salida a Chalma, [19.0337N, 99.3013W], 3100 m alt., 3 Nov 1965, J. Rzedowski 21622 (MICH). **Oaxaca: Municipio Candelaria Loxicha**, 150 km de Oaxaca a Puerto Ángel, [15.97655444N, 96.49359223W], 1318 m, anonymous s.n. (IBUG, MEXU). **Municipio** **Oaxaca de Juárez**, cerro de San Felipe, [17.16095186N, 96.6893169W], 3100 m alt., 29 Dec 1936, W.H. Camp 2408 (NY); Sierra de San Felipe, [17.16095186N, 96.6893169W], 3048 m alt., 18 Sep 1894, C.G. Pringle 4895 (MSC, US), 1894, C.L. Smith 922 (US). **Municipio** **San Juan Mixtepec**, laderas 3 km al S del río San Lucas, a un costado de Tziquicuaza, Distrito Juxtlahuaca, 2260 m alt., 20 Dec 1989, J. Reyes 2133 (MEXU). **Municipio** **San Martín Peras**, Santiago Juxtlahuaca, San Martín Peras, 2.3 Km al S de la intersección a San Martín Peras sobre la carr a Coicoyán de las Flores, 17.29425N, 98.202056W, 2455 m alt., 31 Oct 1994, J.L. Panero and I. Calzada 5326 (MEXU, TEX). **Municipio** **San Miguel el Grande**, 23 km rumbo a Chalcatongo de Tlaxiaco, [17.13037088N, 97.61779244W], 3093 m alt., 24 Jun 1980, A.A. Beetle M-4754 (MEXU). **Municipio** **San Pablo Macuiltianguis**, Los Tanques, paraje La Brecha, 2850 m alt., 27 Jul 1980, R. Lucero 55 (XAL); 32 km N of the turnoff to Guelatato, Distrito Ixtlán, 2050 m alt., 1 Feb 1981, G.J. Martin 289 (MEXU); cerca de la Puerta del Sol, rumbo a San Pablo Macuiltianguis km 8, [17.5331089N, 96.51222237W], 2650 m alt., 10 Jan 1981, R.V. Ortega and M. Ortiz 1616 (MEXU); sobre brecha 290 a Macuiltianguis, [17.54345447N, 96.53783114W], 2610 m alt., 20 Mar 1980, G. Pérez C-73 (MEXU). **Municipio** **San Pedro Yolox**, 7.5 km N of the juncture of the road between Yolox and highway 197 and the road to Reforma, [17.59543294N, 96.53789214W], 2500 m alt., 25 Mar 1981, G. Martin 452 (MEXU); cerro Carrizo, subida por San Martín Buenavista, Sierra de Juárez, [17.65972222N, 96.57694444W], 2386 m alt., 21 May 2002, R. Torres 16296 (MEXU). **Municipio** **Santa Catarina Ixtepeji**, road above Paraje la Cumbre, Sierra Juarez, Parque Nacional Benito Juarez, 22 km NNE of Cd. Oaxaca on MEX 175, [17.17407129N, 96.60481062W], 2600 m alt., 3 Nov 2002, A.L. Reina and T. Van Devender 2002-1035, (ARIZ), 2002-1036 (ARIZ); El Punto, 28 km al NE de Oaxaca, sobre la carretera a Ixtlán de Juárez, [17.21768954N, 96.5834652W], 2400 m alt., 2 Dec 1964, J. Rzedowski 19247 (MICH). **Municipio** **Santa María Tlahuitoltepec**, 35 km N of Ayutla along road from Mitla to Choapam, slope of cerro Zempoaltepetl, [17.14245N, 96.00997W], 2470 m alt., 22 Nov 1986, D.E. Breedlove and J. Sigg 65965 (TEX); vecinity of cerro Zempoaltepetl, [17.129956N, 96.01353W], 3300 m alt., 10 Aug 1950, B. Hallberg 912 (US). **Municipio** **Santiago Comaltepec**, Humo Chico, [17.56576644N, 96.49880183W], 3160 m alt., 13 Jan 81, R.V. Ortega and M. Ortiz 1701 (IBUG, MEXU, UAMIZ); parte más alta del cerro Humo Chico, distrito de Ixtlán, [17.5607N, 96.5355W], 2200 m alt., 25 Feb 1972, J. Rzedowski 28833 (MICH). **Municipio** **Santiago Textitlán**, paraje arriba del Chorro, 16.69369444N, 97.26777778W, 1717 m alt., 3 Jan 2007, I. Trujillo 1281 (INEGI); paraje Tierra Morada, 16.72897222N, 97.41727778W, 1792 m alt., 8 Jan 2007, I. Trujillo 1300 (INEGI). **Municipio** **Santiago** **Zoochila**, Santa María de Yalina, district of Villa Alta, [17.2322N, 96.2576W], 1930 m alt., 1 Nov 1944, J. Vera 3583 (MICH). **Municipio** **Zimatlán de Álvarez**, camino al Sotol, San Pedro El Alto, [16.72388889N, 97.11888889W], 2700 m alt., 3 Feb 1995, E. García 3 (MEXU). **Without municipality**, between Mitla and Cuesta, 3 Jan 1966, W.R. Ernst 2359 (MEXU, US); between Mitla and cerro San Felipe, 14 Feb 1966, W.R. Ernst 2760 (MEXU), 2765 (MEXU, US); 14 km al S de San Pedro Teococuilco, 2900 m alt., 28 Feb 1972, J. Rzedowski 28918 (IBUG). **Veracruz: Municipio Calcahualco**, 3.5 km by road N of Vaquería, 0.8 km from junction with Coscomatepec-Escola-Jacal road, 1 km by road W of Escola, [19.11666667N, 97.23333333W], 2700 m alt., 15 Nov 1981, M. Nee 23149 (XAL). **Municipio La Perla**: vertiente del volcán Pico de Orizaba en el borde con el estado de Puebla, [19.03164594N, 97.22135931W], 3600 m alt., 27 Jul 1971, I. Nevling and A. Gómez-Pompa 2072 (F); Mt. Orizaba, [19.03333333N, 97.26666667W], C.A. Purpus 3013 (F, US), 27 Feb 1892, J.G. Smith 591 (US), 600 (US).

***Agrostis gigantea***

**MEXICO. Mexico:** **Municipio El Oro**, near Tultenango, [19.8122218N, 100.0753397W, 2560 m alt], 13 Jul 1901, J.N. Rose and R.H. Hay 5421 (US). **Municipio Nicolás Romero**, 2 km al W de San José del Vidrio, sobre el camino a Villa del Carbón, [19.64942358N, 99.40663068W], 2716 m alt., 30 Jul 1981, R. Guzmán 3952 (MEXU). **Municipio Toluca**, 2 km al W de Toluca, por la carretera a Morelia, 8 Aug 1981, R. Guzmán 4184 (MEXU). **Mexico City** **Alcaldía Cuajimalpa**, Área Natural Protegida Desierto de los Leones, 19.28722222N, 99.31472222W, 2250 m alt., 14 Jun 2003, J.A. Gutiérrez et al. 719 (MEXU [*]). **Michoacán: Municipio Nahuatzen**, 2 km al S de Arantepacua, [19.58546309N, 101.9724333W], 2300 m alt., 13 Nov 1991, E. García and E. Pérez 4101 (CIIDIR). **USA.** **Arizona:** Cochise County, Chiricahua Mountains, Barefoot Park, [32.0044N, -109.356W], 2500 m, 7 Oct 1992, J.R and C.G. Reeder 8948 (ASU). **Pima County**, Mount Lemmon highway, ca. 2k northwest of Mount Bigelow, Bear Wallow, 32.4225N, 110.737W, 17 Sep 1992, M.A. Baker 10214 (ASU, DES). **California**, **San Diego County**, Cuyamaca Mountains, Cuyamaca Rancho State Park, Arroyo Seco Creek, NW of Green Valley Campground. 0.4 mi NW of Arroyo Seco Fire road gate, 32.90959N, 116.58862W, 1192 m alt., 18 Sep 2010, L. Hendrickson 5156 (SD). **New Mexico**: **Lincoln County**, Eagle Creek and FSR 120, 33.390912N, 105.610703W, 2134 m alt., 13 Sep 2002, R.D. Worthington 21703 (UTEP); SW side of Ruidoso along Hwy 70 near country line crossing, 33.30383333N, 105.6498333W, 2049 m alt., 20 Jul 2003, R.D. Worthington 32157 (UTEP); back of San Andres Canyon at Cottonwood Spring, 32.78722222N, 105.8844444W, 1760 m alt., 30 Jul 2002, R.D. Worthington 31453 (UTEP); Silver Springs Canyon and highway 24 at the reservation boundary, 33.0066N, 105.655702W, 2393 m alt., 14 Aug 2002, R.D. Worthington 31510 (UTEP); Sacramento Mountains, Lower Cathey Canyon about 2.2 road miles, 32.80843333N, 105.7859W, 2774 m alt., 2 Sep 2007, R.D. Worthington 34965 (UTEP); Sacramento Mointains, about 4.7 road miles up Wills Canyon (FSR 169) from Río Penasco, 32.8221N, 105.6772333W, 2423 m alt., 21 Aug 2013, R.D. Worthington 37342 (UTEP). **Otero County**, Karr Canyon road at Trail 105, [32.879731N, 105.79004W], 2743 m alt., 26 Aug 1979, R.D. Worthington 5140 (UTEP).

***Agrostis hyemalis***

**MEXICO. Aguascalientes: Municipio San José de Gracia,** presa La Araña, [22.22426408N, 102.6244147W], 2600 m alt., 22 Sep 1986, M. de la Cerda 30-25 (IEB). **Chihuahua: Ocampo,** near Mirador de Cascada de Basaseachic, 28.1647N, 108.197, 2022 m alt., 7 Sep 2008, P.M. Peterson and J.M. Saarela 22080 (US). **Coahuila: Municipio Monclova**, Monclova, Gloria Mountains, [26.81784N, 101.2228W, 1846 m alt.], 12 Apr. 1939, B.C. Tharp s.n. (SRSC). **Municipio Ocampo**, Madera del Carmen, 13.5 mi NE of Los Pilares, 28.9497N, 102.586W, 2309 m alt., 21 Sep 2007, P.M. Peterson et al. 20973 (US); Madera del Carmen, 17.7 mi NE of Los Pilares on road towards Campo Dos, 28.9645N, 102.556W, 1954 m alt., 21 Sep 2007, P.M. Peterson et al. 21008 (US); Madera del Carmen, 2.8 mi from Campo Uno, up the road towards the summit, 29.0139N, 102.603W, 2594 m alt., 22 Sep 2007, P.M. Peterson et al. 21032 (US); Cañón de Sentenela [Centinela], Hacienda Piedra Blanca, Sierra del Carmen, [28.958333N, 102.558333W, 2000 m alt.], 1936, anonymous 242 (MEXU), 6 Jul 1936, F.L. Wynd 544 (NY). **Durango: Municipio Durango**, áreas agrícolas de riego del Valle del Guadiana, 23.98416667N, 104.5977778W, 1850 m alt., May 1996, S. Aguirre 194 (MEXU); Sierra Madre Occidental, S of Durango on road towards La Flor and 4.7 mi N of Tableteros, 23.7106N, 104.721W, 2135 m alt., 1 Oct 2007, P.M. Peterson et al. 21178 (CIIDIR, US); 23 mi N of highway 40 turnoff on road towards San Luis de Villa Corona, 24.0727N, 105.49W, 2194 m alt., 29 Sep 2008, P.M. Peterson and J.M. Saarela 22426 (US). **Municipio Pueblo Nuevo**, Aserraderos, El Salto, [23.7833N, 105.367W, 2530 m alt.], 1934, anonymous s.n. (MEXU). **Municipio San Dimas**, ejido Vencedores, NE del poblado Vencedores, rodal 1297, 24.40555556N, 105.7111111W, 2550 m alt., Aug 1997, A. Park 53 (CIIDIR). **Municipio Santiado Papasquiaro**, Bajío de Vacas (Hacienditas), 25.040833N, 105.407778W, 2650 m alt., 5 Oct 1990, A. Benítez 2636 (CIIDIR, MEXU). **Municipio Suchil**, Arroyo El Temascal, 4 km al SO de Piedra Herrada, [23.35N, 104.25W], 2519 m alt., 3 May 1981, S. González 1637 (CIIDIR, IBUG, IEB, MEXU). **Hidalgo: Municipio Tlanchinol**, 4 km al NE de Tlanchinol, [21.01878448N, 98.64726743W], 1500 m alt., 16 Jan 1975, R. Servín s.n. (CIIDIR). **Jalisco: Municipio Atenguillo,** 500 m al N del camino Volcanes-Tlapa, 1850 m alt., 20 Apr 1987, J. Pérez de la Rosa 1333 (IBUG). **Municipio Colotlán**, Arroyo de la Cantera, [22.12677032N, 103.2633633W], 1665 m alt., 6 Nov 1976, V. Vazquez s.n. (IBUG); Colotlán, [22.12347176N, 103.254677W], 1685 m alt., 6 Feb 1976, V. Vazquez s.n. (IBUG). **Municipio Guadalajara**, Guadalajara, 1552 m alt., 17 Apr 1975, anonymous s.n. (IBUG), 15 Apr 1975, R. Bravo s.n. (IBUG); Country Club, Guadalajara, [20.70803914N, 103.3725923W], 1550 m alt., 24 Nov 1976, J. Rodríguez s.n. (IBUG). **Municipio Mexquitic**, entre Charca Azul y Pinos Altos, 2570, 9 Aug 1986, F.J Santana 1813 (IBUG). **Mexico: Municipio San Simón de Guerrero**, 3 km sobre la desviación a San Simón de Guerrero, por la carretera Temascaltepec-Tejupilco, [19.01841341N, 100.0282873W], 1974 m alt., 15 Mar 1983, E. Manrique et al. 213a (MEXU); 4 km sobre la desviación a San Simón de Guerrero, por la carretera Temascaltepec-Tejupilco, [19.01816997N, 100.0225366W], 2000 m alt., 15 Mar 1983, E. Manrique et al. 214 (MEXU); 1 km antes de San Simón de Guerrero, [19.01987402N, 100.0169576W], 2100 m alt., 7 Feb 1984, E. Manrique et al. 693 (MEXU). **Municipio Temascaltepec**, alrededores de Temascaltepec, por la salida a Real de Arriba, [19.04379182N, 100.0196481W], 1930 m alt., 28 Oct 1983, E. Manrique et al. 538 (MEXU). **Michoacán: Municipio Morelia**, lagunetas y zonas pantanosas aledañas a la cortina de la presa que se encuentra en el poblado de Umécuaro, 40 km al S de Morelia, [19.52337778N, 101.2533778W], 2177 m alt., 28 Aug 1999, A. Novelo and L. Ramos 3720 (IEB, MEXU); 3 km al S de San Miguel del Monte, [19.60498889N, 101.1231389W], 2250 m alt., 1 May 1988, J. Rzedowski 46520 (CHAPA, IEB, MEXU, XAL). **Municipio Queréndaro**, 2 km al N de Real de Otzumatlán, [19.73527778N, 100.8565583W], 2173 m alt., J. Rzedowski 41859 (IEB). **Municipio Zinapécuaro**, 500 m al O del balneario Las Adjuntas, [19.84393317N, 100.7548502W], 2104 m alt., 3 Feb 1989, M.J. Jasso 836 (IEB, MEXU); 500 m al SE de Santa Cruz, [19.83698889N, 100.7832389W], 2033 m alt., M.J. Jasso 847 (IEB); 19 km al E de Queréndaro, sobre la carretera a Maravatío, [19.84277778N, 100.73515W], 2300 m alt., 15 Feb 1987, J. Rzedowski 42461 (CHAPA, CIIDIR, ENCB, IBUG, IEB); cañada El Salto, cerca de Bocaneo, [19.83600833N, 100.7823389W], 2000 m alt., 1 Feb 1986, J. Santos 1225 (CHAPA, ENCB, IBUG, IEB, MEXU). **Querétaro: Municipio Pinal de Amoles**, 9 km al S de Santa Águeda, sobre el camino a Ahuacatlán, [21.23845N, 99.60752778W], 1386 m alt., J. Rzedowski 46654 (IEB). **San Luis Potosí: Municipio San Luis Potosí**, San Luis Potosí, 1877, J.G. Schaffner 1033 (MEXU); ex convalli San Luis Potosí, J.G. Schaffner 140 (NY). **USA. Texas:** **Brewster County**, E slopes of Lost Mine Peak in Pine Canyon of Chisos Mountains, Big Bend National Park, [29.26134942N, 103.2218174W], 1402 m alt., 28 May 1959, B.H. Warnock 17863 (SRSC); along upper Cattail Canyon stream bed, Big Bend National Park, [29.27343273N, 103.335177W], 1828 m alt., 26 Aug 1966, B.H. Warnock 20722 (SRSC); Iron Mountain, Bill Blakemore ranch, [29.12982574N, 103.106205W], 1371 m alt., 23 May 1969, B.H. Warnock 22859 (SRSC). **Brooks County**, SW side of Vargas Creek ca. 0.6 airmiles NW of culvert on R. M. 755, 1.1 airmiles SE of La Viznaga Windmill, Encinitos Ranch, Santa Elena SE Quadrangle, 26.83206N, 98.34886W, 66 m alt., 20 May 2007, W.R. Carr et al. 25530 (TEX). **El Paso** **County**, near tank at the base of the North Mountain at Hueco Tanks State Park, ca. 30 miles east of El Paso, [31.917799N, 106.043523W], 1381 m alt., 28 Apr 1976, M.L. Butterwick and S. Osburn 2664 (TEX); above rock-wall dam at Hueco Tanks, about 30 miles northeast of El Paso, [31.917799N, 106.043523W], 1219 m alt., 30 May 1947, B.H. Warnock 5844 (SRSC, TEX); above rock-wall dam at Hueco Tanks, 30 mi NE of El Paso, [31.917799N, 106.043523W], 1219 m alt., 30 May 1947, B.H. Warnock 5845 (LL, SRSC); above rock-wall dam at Hueco Tanks, Hueco Mountains, [31.917799N, 106.043523W], 1219 m alt., 30 May 1947, B.H. Warnock 5857A (SRSC); above rockwall dam at Hueco Tanks; Hueco Mountains, [31.917799N, 106.043523W], 1218 m alt., 30 May 1947, B.H. Warnock 5858 (NY, SRSC, TEX); East Mountain, wet area below dam, [31.92054186N, 106.0372341W], 1219 m alt., 20 May 1995, R.D. Worthington 24779 (NLU). **Hidalgo** **County**, McAllen, 26 Apr 1973, L.M. González 652 (IBUG). **Jeff Davis County**, along Cherry Creek downstream from (N of) Big Rock and mouth of Willow Canyon, Cherry Canyon Ranch, Buckhorn Mountain Quadrangle, 30.87767N, 104.04439W, 1389 m alt., 31 May 2005, W.R. Carr et al. 23988 (TEX); Fern Canyon, 17 May 1946, V.L. Cory 53155 (MICH); Cherry Canyon Ranch, 30 Apr 2007, R.T. Harms 65 (TEX); Davis Mountains, spring in Madera Canyon on NW slope, Mount Livermore, [30.63300225N, 104.1879866W], 2100 m alt., 4 Aug 1936, L.C. Hinckley 618 (NY); Mount Livermore, 1 Jan 1936, L.C. Hinckley s.n. (TEX); Mitre Peak Girl Scout Camp, [30.48001837N, 103.7911201W], 1432 m alt., 1 May 1965, E. Keough 302 (SRSC, TEX); NE Davis Mountains, 12 mi W of highway 17 on FM 1832, Boy Scout Camp, Little Aguja Canyon, 30.92081003N, 103.8268713W, 1402 m alt., 19 May 1985, J. Larke 177 (SRSC); along sand and gravel bars of creek, Little Aguja Canyon, Davis Mountains, [30.808174N, 103.909122W], 1310 m alt. 12 Jun 1928, E. Palmer 34495 (NY); N slopes of Timber Mountain, Davis Mountains, [30.84737489N, 103.9517162W], 1524 m alt., 14 May 1950, B.H. Warnock 9053 (LL SRSC); at mouth of Madera Canyon in Davis Mountains, [30.92938253N, 103.8134304W, 1030 m alt.], 1 May 1955, B.H. Warnock 12205 (LL, SRSC); Fern Canyon, 16 May 1937, B.H. Warnock T266 (SRSC, TEX, US); Musquiz Canyon between Castle Hill and Flying Eagle, [30.50915544N, 103.5712028W, 1217 30.92938253N, 103.8134304W], 1 May 1938, B.H. Warnock T358 (SRSC, TEX); Davis Mountains Madera Canyon, roadside park on highway 118, 30.705N, 104.1W, 1767 30.92938253N, 103.8134304W, 25 Jun 1994, R.D. Worthington 23198 (GA, SAT, VDB); Canyon Star Mountain, Davis Mountains, 12 Apr 1914, M.S. Young s.n. (TEX); Gorge, Mount Livermore, [30.63627504N, 104.1730738W, 2506 m alt.], 14 Aug 1914, M.S. Young s.n. (TEX). **Presidio County**, at spring about 2 mi SE Russell Menzies ranch house in Chinati Mountains, [29.900195N, 104.457718W], 1371 m alt., 31 May 1947, L.C. Hinckley 3842 (NY).

***Agrostis laxissima***

**GUATEMALA. Quezaltenango: Municipio San Martín Sacatepéquez**, región Las Nubes, S of San Martín Chile Verde, [14.80276N, 91.66606W], 2250 m alt., 16 Jan 1941, P.C. Standley 83681 (F); region of Boxantin, SE of San Martin Chile Verde, [14.80110457N, 91.66992199W], 2400 m alt., 16 Jan 1941, P.C. Standley 83813 (F, US); Sierra Madre Mountains, about 6 km (airline) N of San Marcos, [15.00802067N, 91.80683806W], 2700 m alt., 13 Dec 1963, O. Williams et al. 25889 (F). **Municipio Zunil**, Las Fuentes "Georginas" (aguas termales), [14.74850191N, 91.47996016W], 2400 m alt., 6 Jan 1953, M. Koninck 144 (US). **Sacatepéquez: Municipio Alotenango**, middle and upper S facing slopes of Volcán Gemelos, [14.48965817N, 90.87703949W], 2100−3200 m alt., 26 Jan 1942, J.A. Steyermark 43252 (F). **San Marcos: Municipio San Marcos**, Mountains along the road between San Marcos and Serchil, 2700–3150 m alt., 30 Jan 1941, P.C. Standley 85387 (F, US); outer slopes of Tajumulco Volcano, Sierra Madre Mountains, about 8-10 km W of San Marcos, [15.01322208N, 91.85952686W], 2300 m alt., 31 Dec 1964, O. Williams et al. 26811 (F). **Solola: Municipio Nahualá**, uppermost ridge of Volcán Santo Tomás, [14.7172469N, 91.48329055W], 3000-3200 m alt., 22 Jan 1940, J.A. Steyermark 34941 (F); near Nahualá, Sierra Madre Mountains, [14.82516609N, 91.30651545W], 2500 m alt., 17 Dec 1962, L.O. Williams et al. 23221 (F, US). **MEXICO: Chiapas: Municipio Motozintla**, near summit of cerro Mozotal, [15.419722N, 92.336667W], 2750 m alt., 24 Nov 1981, D.E. Breedlove and B. Bartholomew 55831 (MO).

***Agrostis pallens***

**MEXICO. Baja California: Municipio Ensenada**, Arroyo Hediondo, 5 Km SE of Eréndira, [31.241667N, 116.341667W], 75 m alt., 1 Jun 1980, R. Moran 28673 (SD). **Municipio Tijuana**, Coronado Islands, NE slope, South Island, [32.4133N, 117.2474W], 100 m alt., 21 Jun 1960, R. Moran 8312 (SD). **USA. California: San Diego County**, Town of Potrero, Hauser Mountain, Harris Ranch road, near old house site, ca. 3 mi N of Canyon City at highway 94, ca. 4 mi W of Cameron Corners (inters of highway 94 and Buckman Springs Road), 32.63583N, 116.5375W, 1099 m alt., 30 Jun 2006, L. Aerene et al. 137 (SD); 20+ bunches on north slope adjacent to Japacha Fire Road, 1.5 km NW of highway 79, Cuyamaca Rancho State Park, [32.92988N, 116.57268W], 1310 m alt., 21 Jul 1987, M. Curto 404 (SD); Anza-Borrego Desert State Park, Lucky 5 south (unofficial park name), 7.0 miles SSE of intersection of state highway 79 and county highway S-1, a short distance W of S-1, 32.93194N, 116.4897W, 1594 m alt., 11 Jul 2005, L. Hendrickson 1109 (SD); Anza-Borrego Desert State Park. Cuyamaca Mountains, NE of intersection of state highway 79 and county highway S-1, 1.1 surface mi NE of intersection of highway 79 and Mason Valley Truck Trail, W of Golden Queen Mine, 33.02093N, 116.54794W, 1458 m alt., 20 Jun 2006, L. Hendrickson 1683 (SD); East Mesa, Cuyamaca Rancho State Park, 1 mi SW of Granite Spring on the east side of East Mesa Fire road, 32.8954N, 116.5415W, 1487 m alt., 9 Jun 2004, L. Hendrickson et al. 165 (SD); Cuyamaca Lake, Lucky 5 acquisition, location 1, N side of the creek on slope and toe of slope NW of Fages Plaque, 32.995N, 116.54028W, 1440 m alt., 29 Jun 2003, A.E. Pigniolo 1416 (BSCA, SD); Hauser Wilderness, Cleveland National Forest, S of Morena Reservoir and E of Barrett Lake, along the Pacific Crest Trail in Hauser Canyon, N of the Creek, 32.6642N, 116.5411W, 829 m alt., 2 Jun 2008, J.P. Rebman et al. 15457 (SD).

***Agrostis perennans***

**GUATEMALA. Alta Verapaz: Municipio Cobán**, Cobán, [15.47675949N, 90.38294267W], 1219 m alt., 25 Aug 1920, H. Johnson 618 (US); finca Samac near Cobán, [15.47140812N, 90.44624693W], 1372 m alt., 5 Jan 1920, W. Popenoe 914 (US); near Cobán, [15.47675949N, 90.38294267W], 1260−1440 m alt., 1939, P.C. Standley 69533 (F); Cobán, [15.47675949N, 90.38294267W], 1350 m alt., Jun 1906, H. Türckheim 1251 (US). **Municipio Santa Cruz Verapaz**, along Río Frío, S of Santa Cruz, [15.36197719N, 90.43037982W], 1350 m alt., 28 Mar 1941, P.C. Standley 90171 (F). **Municipio Tactic**, SE of Tactic, [15.30719451N, 90.34491273W], 1500 m alt., 30 Mar 1939, P.C. Standley 69975 (F); large swamp just E of Tactic, [15.32263351N, 90.34735885W], 1300 m alt., 20 Feb 1942, J.A. Steyermark 44017 (F, US). **Without municipality**, N of Santa Rosa, 30, Mar 1939, P.C. Standley 69840 (F). **Huehuetenango: Municipio Barillas**, vicinity of Maxbal, about 17 mi N of Barillas, Sierra de los Cuchumatanes, [15.97869873N, 91.30677228W], 1500 m alt., Jul 1942, J.A. Steyermark 48773 (F, US); between Barillas and cerro Victoria, Sierra de los Cuchumatanes, [15.7728N, 91.2875W], 1700–1800 m alt., 29 Jul 1942, J.A. Steyermark 49696a (US). **Municipio San Juan Atitán**, La Sierra (Tujimach), across river from San Juan Atitán, Sierra de los Cuchumatanes, [15.4325N, 91.6481W], 2500–2900 m alt., 8 Sep 1942, J.A. Steyermark 51967 (F, US). **Municipio San Juan Ixcoy**, between Tojquiá and Caxín bluff, summit of Sierra de los Cuchumatanes, [15.5589N, 91.5006W], 3700 m alt., 6 Aug 1942, J.A. Steyermark 50209 (F, US). **Municipio San Mateo Ixtatán**, cerro Cananá, between Nucapuxlac and Cananá, Sierra de los Cuchumatanes, [15.8719N, 91.4203W], 2500–2800 m alt., 18 Jul 1942, J.A. Steyermark 49005 (F, US). **Municipio Todos Santos Cuchumatán**, Sierra de los Cuchumatanes, immediately north of Tojiah [Tojquiá] at km 322 on Ruta Nacional 9N, [15.56363N, 91.65777W], 3200 m alt., 1 Aug 1960, J.H. Beaman 3921 (US). **Quetzaltenango, Municipio La Esperanza**, La Esperanza, [14.87580809N, 91.57920309W], 2400 m alt., 20 Jun 1954, M. Koninck 164 (US). **Quiché: Municipio Nebaj**, Nebaj, in the village, [15.408878N, 91.142943W, 1860 m alt.], 7 Jul 1964, E. Contreras 5255 (US). **San Marcos: Municipio Esquipulas** **Palo Gordo**, Barranco Eminencia, road between San Marcos and San Rafael Pie de la Cuesta, in upper part of the barranco between finca La Lucha and Buena Vista, [14.95036156N, 91.85898547W], 2500–2700 m alt., 6 Feb 1941, P.C. Standley 86307 (F). **MEXICO. Chiapas**: **Municipio Chamula**, Bautista Chico, [92.71970542, 16.80088754W], 2600 m alt., 3 Apr 1986, L. Soto 1285 (MEXU), 1400 (MEXU). **Municipio Jitotol**, 3 mi S of Jitotol, [17.04115208N, 92.85427925W], 1615 m alt., 12 Feb 1965, D.E. Breedlove 8905 (US). **Municipio Pueblo Nuevo Solistahuacán**, 1 km del mirador El Caminero hacia Tapilula, 1900 m alt., 28 Mar 1983, M.A. Magaña 1074 (CHAPA). **Municipio Rincón Chamula San Pedro**, 3 mi N of Pueblo Nuevo Solistahuacán, W slope, [17.1942484N, 92.92057968W], 1800 m alt., 22 Jun 1965, E.W. Lathrop 5850 (US). **Municipio Tenejapa**, along road to San Cristóbal las Casas above Tenejapa Center, [16.79N, 92.5667W], 2164 m alt., 12 Jul 1965, D.E. Breedlove 10877 (US); in the paraje of Sibakte'el, [16.816N, 92.516W], 1768 m alt., 6 Sep 1966, D.E. Breedlove 14815 (US), 14873 (US). **Without municipality**, trail between San Cristobal Las Casas and San Gregorio, 2134 m alt., E. Hernández X X-542 (US). **Durango: Municipio Canantlán**, Sierra del Epazote, cima del cerro de las Antenas, [24.60222222N, 105.1086111W], 3202 m alt., 15 Oct 2010, M.G. Nava et al. 129 (CIIDIR). **Municipio Canelas**, 28.5 km de Canelas, por el camino a Durango, [25.12914162N, 106.4680867W], 2650 m alt., 6 Oct 1985, S. González 3438 (CIIDIR). **Municipio Durango**, Estación Coyotes, al NNO, 15 km al NNO del crucero, por la carretera a San Miguel de Cruces, 23.95388889N, 105.3769444W, 2498 m alt., 23 Aug 2013, S. Heynes et al. 437 (MEXU); 13 mi E of La Ciudad on the Mazatlán–Durango road, [23.9395933N, 104.8897411W], 2770 m alt., J.R. Reeder and C.G. Reeder 2528 (CHAPA). **Municipio Pueblo Nuevo**, near El Salto, Los Angeles National Park, small stream road between Durango and Mazatlan, [23.71854267N, 105.7156774W], 2600 m alt., 6 Sep 1981, A.A. Beetle M-7709 (MEXU); El Salto, Ejido La Victoria (vivero), UAF 6, [23.75N, 105.4166667W], 2620 m alt., 28 Sep 1985, R. Miranda 25 (CIIDIR); Sierra Madre Occidental, 1 mi E of Los Charcos on road towards Mesa La Gloria, 23.0241N, 104.3W, 2700 m alt., 12 Sep 2003, P.M. Peterson and F. Sánchez 17748 (US). **Municipio Suchil**, Reserva de la Biosfera La Michilía, mesa El Burro, [23.41083333N, 104.3108333W], 2650 m alt., 16 Sep 1982, Y. Herrera 258 (CIIDIR, MEXU). **Without municipality**, San Ramon, C.G. Pringle 13289a (US). **Hidalgo: Municipio Agua Blanca de Iturbide**, between San Pedrito and Agua Blanca, [20.36531343N, 98.35109164W], 1980 m alt., 28 Aug 1945, A.J. Sharp 45843 (MEXU, US). **Municipio Epazoyucan**, 1 km al S de El Guajolote, [20.016667N, 98.633333W], 2800 m alt., 25 Aug 1984, J. Rzedowski 38470 (FCME, MEXU, XAL). **Municipio Metztitlán**, en el predio al N de Tres Cruces, 11 km al NE de la población de Metztitlán, 800 m aguas arriba en relación a la cerretera federal Zacualtipán–Molango, [20.51709842N, 98.71100933W], 1388 m alt., 8 Jul 1992, J.L. López 141 (MEXU). **Municipio Mineral del Chico**, alrededores de Las Ventanas, 5 km al N de Pachuca, [20.19252776N, 98.73954093W], 2900 m alt., 2 Nov 1983, S. Acosta et al. 412 (CIIDIR, MEXU); El Chico, 1 Aug 1928, E. Lyonnet 46a (MEXU); Las Cuevas, cerca de Las Monjas, 2550 m alt., 12 Sep 1987, M. Medina 3559 (INEGI); Llanos Grandes, 5 km al NNE de Pachuca, [20.17795406N, 98.7130513W], 2950 m alt., 13 Sep 1995, M. Medina 4225 (MEXU); Mineral del Chico, [20.21157854N, 98.72578571W], 2486 m alt., 19 May 1993, J.P. Pérez 30 (MEXU). **Municipio Mineral del Monte**, 4 km sobre el camino de terracería Real del Monte–Santa Rosalía, [20.11666667N, 98.63333333W], 2620 m alt., 13 Jul 1994, A. Olmos 23 (MEXU); La Minita, 4 km al S de la cabecera municipal, [20.14444444N, 98.69222222W], 2780 m alt., 13 Jul 1994, J.P. Pérez 128 (MEXU). **Municipio Molango de Escamilla**, Coatitlamixtla, 1.7 km al NW, [20.76430281N, 98.69369949W], 1900 m alt., 30 Jun 1997, E. Mayorga and O. Alcántara 748 (MEXU). **Municipio Singuilucan**, 1 km al N de Nopalillo, [20.08283873N, 98.57806355W], 2900 m alt., 12 Oct 1979, J. Rzedowski 36465 (CIIDIR, MEXU). **Municipio Tlanchinol**, Santa María, 2 km al SE, [21.02403883N, 98.60949554W], 1200 m alt., 20 Jun 1998, R. Mayorga and O. Alcántara 1210 (MEXU); cerca de Lontla, [21.08288979N, 98.62347386W], 1450 m alt., 30 Nov 1981, A. Mora and J. Ramírez 606-AMB (MEXU). **Without municipality**, 30 km al NE de Tulancingo, camino a Poza Rica, 1400 m alt., 18 Dec 1988, E. Martínez M-24096ª (MEXU). **México: Municipio Amecameca**, 6.4 mi SE of Amecameca, [19.08728583N, 98.68205303W, 3270 m alt.], 31 Dec 1971, D.B. Dunn and D. Dunn 18576 (MEXU); cerro Venacho, cerca de Amecameca, [19.1177N, 98.6891W], 2800 m alt., 16 Dec 1951, E. Matuda 25723 (MEXU, MICH); Amecameca, [19.1167N, 98.7667W, 2316 m alt.], Sep 1905, C.A. Purpus 1646 (US); ladera W del Iztaccíhuatl, cerca de El Salto, Valle de Ayolco, 3700 m alt., 19 Sep 1966, J. Rzedowski 23500 (CHAPA). **Municipio Atlautla**, Tlamacas, cañada al E de la torre de microondas, [19.06646567N, 98.62811938W], 3700 m alt., 20 Sep 1983, S. Acosta 424 (MEXU). **Municipio Coyotepec**, parte alta de la Sierra de Alcaparrosa, 2800 m alt., 16 Sep 1981, J. Rzedowski 37569 (MEXU, XAL). **Municipio Huixquilucan**, 17 mi E of Toluca, [19.4165813N, 99.37142271W], 3110 m alt., 5 Sep 1953, J.R. Reeder and C.G. Reeder 2219 (MEXU). **Municipio Ixtapaluca**, Llano Tepochaico, 10 km al S de Llano Grande, [19.3N, 98.883333W], 3250 m alt., 12 Aug 1980, J. Rzedowski 26816 (XAL); Estación Experimental de Investigación y de Enseñanza de Zoquiapan, 8 km al S de Río Frío, Llano Aculco, [19.29079527N, 98.67281007W], 3230 m alt., 10 Sep 1978, R. Vega 514 (CHAPA). **Municipio** **Jilotzingo**, 1 km al E de Santa María Mazatla, [19.50981698N, 99.37950437W], 2400 m alt., 16 sep 1979, J.H. Cota 19 (MEXU); Santa María Mazatla, [19.51591205N, 99.37832944W], 2850 m alt., 16 Sep 1979, Kohlmann 290 (MEXU). **Municipio Joquicingo**, carretera Joquicingo–Malinalco, 3 km al NE de Joquicingo, [19.05833333N, 99.5W], 2650 m alt., 1 Sep 1991, J, Gonzálezand O. Miranda 612 (MEXU). **Municipio Juchitpec**, 8 km al E de la exhacienda del Mayorazgo, 2900 m alt., 8 Dec 1974, J. Ortíz 101 (MEXU); ex-hacienda de El Mayorazgo, 2900 m alt., 8 Dec 1974, J. Rzedowski 32625 (CIIDIR). **Municipio Nicolás Romero**, 1 km al NW de Cahuacán, [19.64748836N, 99.41457337W], 2700 m alt., 1 Nov 1983, S. Acosta and S. Aguilar 436 (FCME, UAMIZ), 437 (CIIDIR, IEB, MEXU), J. Rzedowski 38370 (CIIDIR, IEB, XAL). **Municipio Ocuilán**, carretera Tres Marías–Chalma, 2980 m alt., 22 Jan 1998, A. Miranda 1403 (MEXU). **Municipio San José del Rincón**, 4.35 km al SO de San José del Rincón, [19.65083333N, 100.1941667W], 2920 m alt., 20 Oct 1999, A. Ramírez 912 (MEXU). **Municipio Temascaltepec**, frente al camino en construcción, por la carretera Sultepec–La Puerta, al S del Nevado de Toluca, [18.991715N, 99.911636W], 2600 m alt., 1 Aug 1981, R. Guzmán 4019 (MEXU); Cajones, [19.052781N, 99.879719W, 1240 m alt.], 7 Jan 1932, G.B. Hinton (TEX); rancho La Ciénega, Ejido de La Comunidad, [19.12444444N, 99.89083333W], 2860 m alt., 15 Sep 1993, R. Mendoza 63 (MEXU). **Municipio Texcoco**, cerro Tláloc, 18 km al E de Tequexquinahuac, sobre la brecha maderera a la cima, 17.5 km al ESE de Texcoco, [19.48275708N, 98.70691757W], 3440 m alt., 26 Oct 1976, S.D. Koch 76223 (CHAPA, MEXU); cerro Tláloc, 15 km al E de Tequesquinahuac, 17.5 km al ESE de Tezcoco, [19.48275708N, 98.70691757W], 3440 m alt., 26 Oct 1976, S.D. Koch 76223 (US). **Municipio Tlalmanalco**, San Rafael, falda W del Iztaccíhuatl, [19.21152965N, 98.72950356W], 2900 m alt., 16 Nov 1952, E. Matuda 27610 (MEXU); cerca de Santo Tomás Atzingo, [19.16956401N, 98.77445615W], 2500 m alt., 26Oct 1970, J. Rzedowski 27905 (CHAPA). **Municipio Villa de Allende**, 55 km NW of Toluca on Mex 15, [19.40910737N, 100.1127971W], 2500 m alt., 20 Oct 1976, J. Brunken and C. Perino 431 (MEXU). **Municipio Villa Guerrero**, 3.5 km por camino de terracería Porfirio Díaz–Totolmajac, [18.92253164N, 99.70119685W], 2360 m alt., 29 Jul 1987, R. Méndez 96 (MEXU). **Municipio Xalatlaco**, Xalatlaco, [19.18550681N, 99.40693398W], 2800 m alt., 4 Dec 1993, A. Bolaños s.n. (MEXU). **Municipio Xonacatlán**, San Juan del Potrero (El Potrero), 8 km al W de San Francisco Chimalpa, [19.4489N, 99.4136W], 3100 m alt., 3 Sep 1967, J. Rzedowski 24337 (MICH). **Municipio Zinacantepec**, 1.3 km al SW de la comunidad La Joya, [19.16888889N, 99.78861111W], 3405 m alt., 12 Oct 1995, A. Ramírez 679 (MEXU). **Without municipality**, Sualcingo, límite con el estado de Tlaxcala, 8 Nov 1953, E. Matuda 29587 (MEXU, US); near Mexico, 2286 m alt., 1 Oct 1895, C.G. Pringle 5958 (US). **Mexico City: Alcaldía Azcapotzalco**, Azcapotzalco, vicinity of México, Jul 1910, A.S. Hitchcock 5920 (US). **Alcaldía Cuajimalpa**, La Venta, [19.3349N, 99.3106W, 2860 m alt.], 19 Sep 1930, M. Saint-Pierre 921 (MICH, US). **Alcaldía Milpa Alta**, valle intermontano al E del cerro Acopiaxco y al N del Chichinautzin, [19.12027804N, 99.13781462W], 3040 m alt., 9 Aug 1985, R. Méndez 24 (MEXU); camino al Volcán San Lorenzo Tlacoyucan, [19.15328715N, 99.05535579W], 2900 m alt., 10 Oct 1985, A. Miranda and P. Guerrero 60 (MEXU); cerro Xilcoayo [Cilcuayo], [19.103N, 98.988W], 2900 m alt., 24 Jan 1976, A. Ventura 896 (CHAPA); cerro Xigtoni, 3050 m alt., 28 Feb 1976, A. Ventura 1070 (CIIDIR, IBUG). **Alcaldía Tlalpan**, Volcán Pelado, [19.151N, 99.2171W], 3200 m alt., 11 Oct 1986, Campos et al. 420 (MEXU), PEL-NW-335-7 (FCME); Ajusco, [19.2333N, 99.2W], 2590 m alt., 18 Aug 1926, G.L. Fisher 60 (US); cerro Magdalena, Serranía del Ajusco, [19.22851N, 99.1865W], 2930 m alt., 23 Oct 1937, E. Lyonnet 1904 (CHAPA, MEXU, US); cerro Malacatepec, [19.15185786N, 99.27051218W], 3060 m alt., 24 Oct 1985, E. Manrique et al. 1198 (MEXU), 1206 (MEXU); Ajusco, [19.203539N, 99.255122W], 3500 m alt., 9 Dec 1951, E. Matuda 25758 (MEXU, US); caldera El Guarda, al S de Parres, [19.13666651N, 99.17806128W], 3000 m alt., 5 Oct 1985, A. Miranda and P. Guerrero 114 (MEXU); Parres, [19.13666651N, 99.17806128W], 3000 m alt., 30 Aug 1910, C.R. Orcutt 4441 (MEXU); Ajusco, [19.13666651N, 99.17806128W], 3000 m alt., 20 Aug 1910, C.R. Orcutt 6341 (US); Estación La Cima, Serranía del Ajusco, [19.113331N, 99.195W], 3000 m alt., 22 Aug 1965, J. Rzedowski 20447 (CHAPA); cerca de la Estación La Cima, Serranía del Ajusco, [19.113331N, 99.195W], 3000 m alt., 24 Oct 1965, J. Rzedowski 21535 (TEX); La Cumbre, al O del pueblo de Parres, [19.14387336N, 99.20107279W], 3300 m alt., 6 Oct 1994, P. Tenorio 18930 (MEXU). **Alcaldía Xochimilco**, camino de San Francisco Tlalnepantla hacia el Volcán Cuautzin, [19.19774888N, 99.11085861W], 2860 m alt., 11 Oct 1985, A. Miranda and P. Guerrero 91 (MEXU); Tochuca, [19.20296504N, 99.10272242W], 2500 m alt., 4 Sep 1976, A. Ventura 2046 (FCME, MEXU, UAMIZ). **Michoacán: Municipio Chilchota**, 14 km S of the junction of highways 15 and 37 at Carapan, [19.84889096N, 102.0342454W], 2070 m alt., 20 Aug 1975, G. Davidse and J. Davidse 9905 (MEXU). **Municipio Epitacio Huerta**, 2 km al SE de Laguna de Servín, [20.26131944N, 100.2556083W], 2750 m alt., 15 Nov 1987, J. Rzedowski 45770 (IEB). **Municipio Huiramba**, parte alta del cerro Burro, [19.43503889N, 101.5121083W], 3200 m alt., 6 Sep 1987, J. Rzedowski 44637 (IEB). **Municipio Jungapeo**, río Tuxpan, bajo de Agua Blanca, [100.4951188N, 19.47811839W], 1380 m alt., 24 Apr 1982, E. Martínez 350 (MEXU). **Municipio Morelia**, 2 km al S de Umécuaro, [19.51352778N, 101.2654278W], 2200 m alt., 17 Oct 1988, J. Rzedowski 47593 (IEB). **Municipio Salvador Escalante**, 18 mi S of Pátzcuaro, [19.343067N, 101.650097W], 2713–2743 m alt., 20 Nov 1961, R.M. King and T.R. Soderstrom 5166 (TEX, US); La Laguna, cerca de San Gregorio, [19.41886944N, 101.4982472W], 2700 m alt., J.S. Martínez 1012 (CHAPA, ENCB, IEB). **Municipio Tacámbaro**, at km 56–7 above Quiterio on road from Pátzcuaro to Tacámbaro, [19.4N, 101.5W, 2700 m alt.], 10 Nov 1949, H.E. Moore et al. 5592 (US). **Municipio Tingambato**, Llano de Cananguio, al NE de Pichátaro, [19.60430556N, 101.8310278W], 2600 m alt., H. Díaz-Barriga and E. Pérez 5929 (CIIDIR, IEB). Cerro Comburinda, [19.46563889N, 101.8139722W], 2676 m alt., 24 Jul 2015, M. Sales 287 (MEXU). **Municipio Zinapécuaro**, 5 km al NW de la presa Laguna Larga, [19.82405833N, 100.6922194W], 2970 m alt., 30 Aug 1986, H. Díaz-Barriga and S. Zamudio 2712 (ENCB, FCME, IEB). **Morelos: Municipio** **Huitzilac**, Parque Nacional Lagunas de Zempoala, Laguna Tonatihua, [19.05529062N, 99.31609205W], 2900 m alt., 17 Sep 1986, V.L. Cardoso 1349 (IEB, MEXU); Laguna Zempoala, [19.05034984N, 99.31696647W], 2790 m alt., 25 Jul 1987, J. Castañeda 184 (MEXU), 187 (MEXU); 2.88 km lineales del NE del puente Paso Morelos, 19.08839722N, 99.18872778W, 3061 m alt., 31 Jul 2012, F. Hinterholzer 201a (HUMO); Tres Marías (Camino de Cuernavaca), [19.0667N, 99.2167W, 3190 m alt.], Sep 1926, E. Lyonnet 46a (US), 46b (MEXU); Lago de Zempoala, [19.05034984N, 99.31696647W], 3000 m alt., 7 Oct 1951, E. Matuda 25591 (MEXU); El Capulín, 5 km de los límites con el Distrito Federal, [19.08619552N, 99.22534177W], 2950 m alt., 19 Oct 1993, J. Morales 169 (MEXU); Parque Nacional Lagunas de Zempoala (Laguna de Tonatihua), [19.028333N, 99.304722W], 2900 m alt., 9 Sep 1986, T. Mundo and L. Cardoso 88 (XAL); Tres Marías, [19.05673704N, 99.23538215W, 2880 m alt.], 23 Aug 1910, C.R. Orcutt 3744 (US); new Cuernavaca–Mexico road, km 45, N of Tres Cumbres, [19.0714009N, 99.21657351W, 3100 m alt.], 18 Jul 1954, G.B. Van Schaack 1966 (CHAPA); Zempoala, [19.05034984N, 99.31696647W], 2750–2950 m alt., 8 Oct 1972, J. Vázquez 3904 (MEXU). **Oaxaca: Municipio** **Santiago Comaltepec**, 26.6 km S of Puente Valle Nacional along MEX 175 from Tuxtepec to Oaxaca and 1.8 km N of Vista Hermosa, [17.64281056N, 96.33685248W], 1490 m alt., 25 May 1990, Utley and Utle 8497 (MEXU). **Municipio** **Totontepec** **Villa de Morelos**, 2 km de la desviación a Mixistlan, camino Mitla–Totontepec, 3000 m alt., 6 Dec 1989, A. Flores 1421 (UAMIZ); road between Ayutla and Totontepec, at turnoff to Villa Alta [17.23333333N, 96.06666667W], 2370 m alt., 5 May 1986, R. Gereau et al. 2104 (MEXU). **Municipio** **Villa Talea de Castro**, prope Talea, Oajaca [Oaxaca], Mina de Dolores, [17.3667N, 96.25W, 1570 m alt.], Aug 1842, F.M. Liebmann 12941 [989] (US). **Without municipality**, Ixtlahuaca, 2500 m alt., 1 Jun 2003, J. Pérez s.n. (CHAPA). **Puebla:** **Municipio Acajete**, El Pinal, a 10 Km al E de Acajete, [19.16441462N, 97.92569791W], 2560 m alt., 16 Sep 1988, P. Tenorio 15195 (IEB, MEXU, TEX). **Municipio Cohuecan**, near río Otlata 72 km SE Mexico City, [18.68N, 98.71W, 2652 m alt.], 12 Aug 1942, J.N. Weaver 948 (US). **Municipio Coyomeapan**, 4 km del entronque con Zoquitlán, 6 km al W de Coyomeapan, [18.28445889N, 97.04862972W], 2451 m alt., 19 Oct 1995, L. Aragón et al. 520 (MEXU), 531 (CHAPA). **Municipio Honey**, 1 km al NE de Honey, sobre la terracería a Pahuatlán (18 km directo al NE de Huachinango), [20.2473066N, 98.20598925W], 2050 m alt., 13 May 1978, S.D. Koch 7820 (CHAPA), Trinidad, near Honey Station, [20.2421N, 98.2105W, 2070 m alt.], 22 Apr 1904, C.G. Pringle 13289b (MICH). **Municipio Hueytamalco**, 14 km después de Teziutlán, rumbo a Hueytamalco, [19.90039036N, 97.29112096W], 1460 m alt., 22 Nov 1985, I. Núñez 409 (MEXU); **Municipio Tepeyahualco**, Central Geotérmica Los Humeros, [19.64662924N, 97.43679992W], 2880 m alt., 27 Jun 2005, I. Acosta 2769 (XAL). **Municipio Teziutlán**, Teziutlán, [19.8167N, 97.35W, 1870 m alt.], 6 Sep 1910, C.R. Orcutt 3953 (US). **Municipio Tlatlauquitepec**, 4 km después de Tlatlauquitepec, rumbo a Teziutlán, [19.84219764N, 97.4801928W], 1860 m alt., 20 Nov 1985, I. Núñez 364 (MEXU). **Municipio Xicotepec**, 5 km de la desviación a Pahuatlán, carretera Huauchinango–Tulancingo, [20.29764384N, 97.97307418W], 2125 m alt., 24 Oct 1987, A. Miranda et al. 523 (MEXU); 1 km de la desviación a la unión de la carretera Huachinango–Xicotepec de Juárez, [20.231213N, 97.960254W], 1220 m alt., P. Tenorio 15772 (TEX). **Municipio Zacapoaxtla**, 8 km después de Zaragoza rumbo a Zacapoaxtla, [19.82318708N, 97.57156961W], 2140 m alt., 21 Nov 1985, I. Núñez 369 (MEXU). **Without municipality**, camino Tlaola–Tlapacoya, 1400 m alt., 9 Sep 1993, F. Basurto 1497 (MEXU). **Querétaro**: **Municipio Amealco**, 8 km al NE de San Pablo, 2700 m alt., 23 Oct 1989, J. Rzedowski 49189a (IEB). **Tlaxcala:** **Municipio** **Tlaxco**, km 32 carretera Tlaxco–Zacatlán, al O de la estación de microondas, 2960 m alt., 30 Oct 1985, A. Miranda 209 (MEXU); 3 mi NE of Tlaxco, [19.68345849N, 98.09150913W], 2800 m alt., 23 Sep 1953, E. Sohns 596 (MEXU). **Without municipality**, estación de microondas, 4 km del límite con Puebla–Tlaxcala, 2850 m alt., 24, Sep 1980, J. Rosasco s.n. (MEXU). **Veracruz: Municipio Acajete**, Acajete, 19.58333333N, 97.01666667W, 1800 m alt., 3 Sep 1980, M.T. Mejía 268 (XAL); Plan de Sedeño, 19.57035087N, 97.00735168W, 1800 m alt., 23 Jul 1982, M.T. Mejía et al. 914 (XAL); comunidad Mesa de la Hierba, [19.55833333N, 97.01666667W], 2000 m alt., 15 Jan 1995, M.J. Lizama 421 (CIB, MEXU). **Municipio Banderilla**, Banderilla, [19.6N, 96.9333W], 1463 m alt., 26 Aug 1959, T.R. Soderstrom 472 (US). **Municipio Calcahualco**, Totozinapa, La Ranchería, camino al Pico de Orizaba, por Coscomatepec, [19.1286664N, 97.16472761W], 2200 m alt., 22 Jul 1982, R. Guzmán et al. 5824 (MEXU). **Municipio Chiconquiaco**, Chiconquiaco, [19.74027778N, 96.82222222W] 2200 m alt., 18 Jan 1995, M.J. Lizama 430 (CIB, MEXU, XAL). **Municipio Coatepec**, Coatepec, 19.45N, 96.95W, 1250 m alt., 13 Nov 1980, M.T. Mejía 424 (XAL), 434 (XAL). **Municipio Cosautlán de Carvajal**, ONO de Cosautlán, [19.34861111N, 97.03416667W], 1330 m alt., 4 Dec 1992, B.V. Hernández 105 (MEXU, XAL); carretera Teocelo–Cosautlán, 1 km antes de Cosautlán, [19.34579593N, 96.96365096W], 1200 m alt., 1 Jul 1998, M.J. Lizama 1094 (MEXU). **Municipio Coscomatepec**, camino al Pico de Orizaba, por Coscomatepec, [19.05844254N, 97.14998176W], 2400 m alt., 22 Jul 1982, R. Guzmán 5853a (MEXU), 5828 (MEXU); carretera entre Coscomatepec y el Pico de Orizaba, [19.0591839N, 97.08396617W], 1790 m alt., 22 Jul 1982, R. Guzmán et al. 5813 (MEXU). **Municipio Huayacocotla**, 17 km del centro de Huayacocotla, dirección NE, [20.5446756N, 98.4725372W, 2130 m alt.], 16 Mar 1980, L. Ballesteros and R. Jiménez 125 (MEXU, XAL); capilla en las afueras de Huayacocotla (2–3 km), carretera a Agua Blanca, [20.5446756N, 98.4725372W], 2100 m alt., 27 Jun 1984, M. Cházaro and S. Solheim 3181 (IBUG). **Municipio Ixhuacán de los Reyes**, carretera Cruz Blanca–Las Minas, 5 km de distancia al empezar a bajar, 19.4N, 97.1W, 2140 m alt., 19 Jun 1988, C. Durán et al. 401 (MEXU, XAL); 4.5 km al WNW de Cosautlán, [19.34027778N, 97.03416667W], 1330 m alt., 4 Dec 1992, B.V. Hernández 105 (CIB). **Municipio Jalacingo**, Allende, [19.79393787N, 97.26256287W], 1700 m alt., 15 Jul 1970, F. Ventura 1368 (CHAPA); 1.5 km W of Orilla del Monte, 14 km (by air) NW of town of Perote, [19.66666667N, 97.31666667W], 2360 m alt., 30 Nov 1981, M. Nee 23406 (XAL). **Municipio Jilotepec**, comunidad Linderos, 19.61944444N, 96.96666667W, 1600 m alt., 6 Nov 1993, M.J. Lizama 115 (CIB); El Esquilón, 19.62496603N, 96.9410784W, 1310 m alt., 30 Sep 1980, M.T. Mejía 297 (CIB, XAL). **Municipio Miahuatlán**, Miahuatlán, 19.70694444N, 96.87222222W, 1700 m alt., 11 Jan 1995, M.J. Lizama 405 (CIB, FCME, UAMIZ). **Municipio Naolinco**, Naolinco, 19.65N, 96.86666667W, 1556 m alt., 13 Dec 1981, M.T. Mejía 693 (XAL); 2 km al O de Noalinco, [19.65972222N, 96.89027778W], 1600 m alt., 16 Sep 1991, H. Sandoval 144 (CHAPA, CIB, MEXU). **Municipio Orizaba**, Orizaba, [18.86217238N, 97.0879177W, 1500 m alt.], M. Botteri 687 (US), Aug 1910, A.S. Hitchcock 6388 (US). **Municipio La Perla**, Mt. Orizaba., Aug 1910, A.S. Hitchcock 6262 (US). **Municipio Perote**, camino a la Toma, 19.56N, 97.245W, 2700 m alt., 23 Oct 1995, G. Castillo-Campos et al. 14558 (XAL); Caja de Agua, [19.55144819N, 97.22500263W], 2350 m alt., 25 Nov 1972, F. Ventura 7460 (CHAPA). **Municipio Tatatila**, camino Las Vigas–Tatatila, 1 km antes de La Mancuerna, 19.675N, 97.125W, 1800 m alt., 7 Jun 1996, H.R. Sandoval and B.V. Hernández 372 (MEXU, CIB, XAL). **Municipio Teocelo**, 2.25 km al NO de Teocelo, 19.40138889N, 96.985W, 1050 m alt., 4 Dec 1992, H. Sandoval 282 (CIB). **Municipio Las Vigas de Ramírez**, carretera Jalapa–Perote, Rafael Ramírez, [19.63128922N, 97.10931188W], 1800 m alt., 26 Aug 1972, J. Dorantes et al. 1604 (MEXU, XAL). **Municipio Xalapa**, Terreno de la SARH, km 4.6 carretera Xalapa–Veracruz, 19.50972222N, 96.86944444W, 1220 m alt., 31 Mar 1993, B.V. Hernández 167 (CIB); Jalapa [Xalapa], [19.5333N, 96.9167W, 1402 m alt.], Sep 1910, A.S. Hitchcock 365 (US), 6615 (US), 6640 (US), F.W. Johnson s.n. (US); El Castillo, 10 km al NNE de Xalapa, 19.55N, 96.86666667W, 1250 m alt., 1 Sep 1981, M.T. Mejía 631 (XAL); El Seminario, NNO de Jalapa, 19.55N, 96.93333333W, 1300 m alt., 1 Jan 1982, M.T. Mejía 733 (XAL); cerca del río Sedeño, al NO de Xalapa, 19.51666667N, 96.95W, 1035 m alt., 3 Jan 1982, M.T. Mejía 751 (XAL); cerro Macuiltépetl, 19.5483N, 96.9208W, 1400 m alt., 11 Sep 1982, M.T. Mejía 939 (XAL); rancho Guadalupe, 3 km W de Xalapa, carretera vieja Xalapa–Coatepec, 1450 m alt., 23 Aug 1975, L. Monroy et al. 62 (MEXU). **Municipio Yecuatla**, 5 km al SO de Yecuatla, [19.83888889N, 96.8125W], 1330 m alt., 16 Sep 1991, H. Sandoval 147 (CIB, MEXU). **Municipio Zongolica**, from El Rancho de Tehuilango to El Rancho de Tepetlampa, Ejido de Zapaltecatl, on the Sierra Madre Oriental, 20–25 km NW of campo experimental de hule, El Palmar, Zongolica, [18.5667N, 96.9167W], 671–1097 m alt., Jan 1944, J.V. Santos 3070 (US). **Without municipality**, La Barranca, carretera a Coscomatepec, 1920 m alt., 22 Jul 1982, R. Guzmán et al. 5857 (MEXU); Mirador, Aug 1841, F.M. Liebmann 714 (US); Atilpan, 1350 m alt., 28 Jun 1974, F. Ventura 10227 (CHAPA, IBUG).

***Agrostis scabra***

**MEXICO. Baja California:** **Municipio** **Ensenada**, Sierra San Pedro Mártir, along crest of range, Dead Horse meadow, SW end of cerro Venado Blanco, [31.01667N, 115.4667W], 2501 m alt., 17 Jul 1988, S. Boyd 2629 (MEXU); Sierra San Pedro Mártir, meadows along road heading S of Vallecitos towards La Encantada, base of cerro Botella Azul, [30.95807N, 115.42347W], 2501 m alt., 19 Jul 1988, S. Boyd et al. 2659 (MEXU); Sierra San Pedro Mártir, along road to Observatory, between Observatory and road to La Tasajera, 31.03333N, 115.47027W, 2501 m alt., 31 Jul 2005, S.J. De Groot and R.K. Lauri 5162 (BRY); Sierra San Pedro Mártir, Jeffrey Pine forest, Yerbabuena, 31N, 115.45W, 2475 m alt., 16 Aug 1967, R. Moran and R.F. Thorne 14168 (TEX); Sierra San Pedro Mártir, Los Llanitos, [30.96667N, 115.43333W], 2550 m alt., 17 Aug 1967, R. Moran and R.F. Thorne 14276 (MEXU, SD); Sierra San Pedro Mártir (along road to Botella Azul, E of Llanitos, at creek crossing), 30.95807N, 115.42347W, 2456 m alt., 13 Jul 2013, S. Ratay et al. 213 (SD); Sierra San Pedro Mártir National Park, upper Vallecitos Meadow, [31.01667N, 115.4667W], 2430 m alt., R.F. Thorne et al. 57212 (ASU); Parque Nacional Sierra San Pedro Mártir, Vallecitos, [31.03333333N, 115.425W], 2438 m alt., 1 Sep 1985, R.F. Thorne et al. 61431 (MEXU); Parque Nacional Sierra San Pedro Mártir, open Jeffrey Pine forest on rocky sloes along intermittend stream with scattered pools of water, in Tenajas, along Observatory Road, [31.033333N, 115.470278W], 2502 m alt., 2 Sep 1985, R.F. Thorne et al. 61493 (MEXU). **Baja California Sur: Municipio La Paz**, The Laguna, Laguna Mountains [Sierra de la Laguna], 22 Sep 1930, M.E. Jones s.n. (RSA). **Chiapas:** **Municipio Chamula**, Bautista Chico, [16.80267242N, 92.71916374W, 2435 m alt.], 22 Aug 1988, B.Y. López 15 (MEXU); cerca de Bautista Chico, [16.80267242N, 92.71916374W, 2435 m alt.], 29 Jul 1993, B.Y. López 924 (MEXU), 926 (MEXU), 3 Jun 1986, L. Soto 1286 (MEXU), 1287 (MEXU), 1288 (MEXU), 1289 (MEXU), 1290 (MEXU), 1291 (MEXU), 1292 (MEXU). **Municipio Jitotol**, 10 km N of Jitotol near Río Hondo, [17.11501243N, 92.86500343W], 1675 m alt., 8 Nov 1981, D.E. Breedlove 55125 (NY); 10 km N of Jitotol near Río Hondo, [17.11501243N, 92.86500343W], 1675 m alt., 8 Nov 1981, D.E. Breedlove and G. Davidse 55125 (MEXU); 12 km N of Jitotol along a side road to an oil well, [17.1209148N, 92.86915287W], 2000 m alt., 28 Oct 1971, D.E. Breedlove and R.F. Thorne 21448 (MEXU). **Municipio** **San Cristóbal de las Casas**, 10 km de la carretera San Cristóbal–Ocosingo, [16.65945436N, 92.55771715W], 2100 m alt., 14 Sep 1993, Borrego 3 (MEXU); N end of San Cristóbal de las Casas valley towards Chamula, [16.76400131N, 92.67686924W], 2200 m alt., 2 Sep 1974, D.E. Breedlove 37118 (MEXU). **Chihuahua: Municipio Bocoyna**, entronque carretera Creel, entrada Bocoyna, Rancho de Lucia, [27.83798907N, 107.5928871W, 2239 m alt.], 10 Sep 2003, R.A. Bye et al. 31881 (US); Arroyo Rituchi con Rancho y Cueva, 27.691N, 107.424W, 2205 m alt., 12 Sep 2003, R.A. Bye et al. 32281 (US); Sánchez, [27.7333N, 107.683W, 2438 m alt.], 12 Oct 1910, A.S. Hitchcock 7680 (US); río Oteros, W of Creel, [27.7535N, 107.667W], 2425 m alt., 16 Oct 1977, R.A. Bye 8233 (MICH). **Municipio Chihuahua**, Sierra del Nido Complex, Nuevas Delicias along río El Alamo, 29.09583333N, 106.4833333W, 1775 m alt., 16 Sep 1981, R.D. Worthington 7354 (UTEP). **Municipio Guachochi**, km 20 carretera Guachochi–Creel, [27.208333N, 107.425W], 2219 m alt., 9 Jul 1997, R. Fierros 1611 (MEXU); Sierra Madre Occidental, Yamuco at 1 km E of Hwy towards Basihuare and Creel, N of río Urique crossing, 27.4004N, 107.489W, 1891 m alt., 26 Aug 2003, P.M. Peterson et al. 17553 (US). **Municipio Madera**, proximidad a la Colonia Chuhuichupa, [29.61317607N, 108.3629683W], 2240 m alt., 1 Sep 1990, A. Benítez 2181 (CIIDIR, MEXU, UAMIZ); vicinity of Madera, [29.2N, 108.15W], 2130 m alt., 27 May 1928, E. Palmer 263 (F, US). **Municipio Ocampo**, near Concheño, [28.313408N, 108.221445W, 1968 m alt.], 3 Jul 1936 H. LeSueur 103 (IND, LSU, MEXU); Basaseachic Falls area, [28.133333N, 108.25], 1951 m alt., 9 Jun 1976, McGill et al. 13258 (ASU); Cascada de Basaseachic, at the confluence of río Basaseachic and río Durazno, ca 2 mi S of Village of Basasechic, just upstream from footbridge, [28.208889N, 108.213611W], 2000–2100 m alt., 17 Oct 1986, G. Nesom and L. Vorobik 5696 (TEX); Basasiachic a 21 km al S de San Juanito y 9 km al N de Creel, [28.225N, 108.058333W], 2150 m alt., 16 Sep 1983, P. Tenorio and R. Torres 4365 (MEXU); Parque Nacional Cascada de Basasachic, [28.16666667N, 108.2083333W], 1600 m alt., 5 Aug 1994, C. Yen 2861 (NY), 2872 (NY). **Municipio Riva Palacio**, campo 73, 29.14888889N, 106.7988889W , 2020 m alt., 18 Jun 2015, U. Lazo 111 (INEGI), 112 (INEGI). **Municipio Urique**, Cuiteco, Ranchito en la cañada, a 5 km de la desviación a San Rafael, [27.474N, 107.913W], 2170 m alt., 12 Sep 2003, P. Tenorio et al. 21977 (US), 21987 (US). **Without municipality**, Marsh Lake, Sierra Madre Mts., 2134 m alt., 19 Sep 1903, M.E. Jones s.n. (RSA); Meadow Valley, Sierra Madre Mts., 2130 m alt., 17 Sep 1903, M.E. Jones s.n. (RSA); cerca del río, 6 Apr 1886, C.G. Pringle s.n. (MEXU). **Coahuila: Municipio Acuña**, Sierra del Carmen; Canyon de Sentenela [Centinela] on Hacienda Piedra Blanca, [29.0833N, 102.317W], 1197 m alt., 6 Jul 1935, F. Wynd et al. 644 (US). **Municipio Arteaga**, La Escondida, Sierra de los Lirios, [25.391667N, 100.575W], 1300 m alt., 3 Jul 1943, E. Lyonnet 4011 (MEXU); 18 km al SE de la Plaza Principal San Antonio de las Alazanas (SE de Saltillo), sierra de la Martha, [25.233333N, 100.483333W], 3000 m alt., 15 Oct 1981, J. Valdés 1431 (TEX); S del Puerto El Tarillal, 39 km al O de Villa de Santiago, [25.45003828N, 100.5447932W], 2600 m alt., 12 Jul 1977, V. Valdez 629 (INEGI); Las Vigas, Cañón de Jamé, Sierra de Arteaga, [25.3333N, 100.65W], 2600–3300 m alt., 15 Sep 1988, J.A. Villareal and M.A. Carranza 4621 (ASU, CIIDIR). **Municipio Ocampo**, Cañón de Centinela, just S and SW of Pico de Centinela, Sierra del Jardín, [29.06666667N, 102.7666667W], 1600–2225 m alt., 31 Jul 1973, M.E. Johnston et al. 11985 (ASU, F, MEXU, OBI); Sierra Maderas del Carmen, at Campo El Tres, an abandoned logging campo in the high country, 29N, 102.6W, 2600 m alt., 5 Aug 1974, T. Wendt and A. Adamcewicz 493 (CHAPA, MEXU); Sierra Maderas del Carmen, near Campo Tres, on ridge between camp and "Hell's Kitchen" to the N, 29N, 102.6W, 2600 m alt., 6 Aug 1974, T. Wendt and A. Adamcewicz 518e (MEXU). **Durango: Municipio Canelas**, predio particular Las Cebollitas, [25.10273363N, 106.4441947W], 2460 m alt., 1 Aug 1990, O. Bravo 1049 (CIIDIR, MEXU); San Ramón, [25.05487828N, 106.4228846W], 2574 m alt., May 1906, E. Palmer 82 (US). **Municipio Durango**, 61 km al S de Durango, por el camino a La Flor, [23.6N, 104.732778W], 2670 m alt., 13 Jul 1990, S. González and A. García 4467 (CIIDIR); entrada al predio Las Bayas (UJED), 93 km al S de Durango, [23.46666667N, 104.8333333W], 2700 m alt., 13 Jul 1990, S. González and A. García 4485 (CIIDIR); predio Las Bayas (UJED), cerro La Grulla, [23.45N, 104.8333333W], 2870 m alt., 15 Jul 1990, S. González and A. García 4544 (CIIDIR); El Rosillo, predio Las Bayas (UJED), [23.48111111N, 104.8338889W], 2723 m alt., 24 Sep 2013, S. Heynes et al. 831 (CIIDIR), 855 (CIIDIR), 857 (CIIDIR), 865 (CIIDIR). **Municipio Durango**, Tres Molinos, en la ribera del río, 23.83333333N, 104.7833333W, 2200 m alt., 28 Jul 2006, L. López et al. 476ª (CIIDIR). **Municipio Mezquital**, Los Charcos, 10 km por el camino a Santa María de Ocotlán, [22.96666667N, 104.35W], 2680 m alt., 18 Jun 1992, S. González and Y. Herrera 5154 (CIIDIR); alrededores del rancho La Escondida, 16 Aug 1985, Y. Herrera 678 (CIIDIR). **Municipio Nuevo Ideal**, río Guatimapé, [24.73583333N, 105.0086111W], 2020 m alt., 21 Oct 1999, A. García 3871 (CIIDIR). **Municipio Pueblo Nuevo**, km 115 carretera Durango–Mazatlán, [23.74722222N, 105.45W], 2700 m alt., Oct 1999, D. Aceval 775 (MEXU); Los Tanques; 20 km S of El Salto, W of Santa Barbara, Arroyo del Infierno, [23.6001N, 105.356W], 2411 m alt., 23 Aug 1963, A. Gordon 9 (MICH); 7 km al O de El Salto, [23.76406781N, 105.3967126W], 2700 m alt., 21 Aug 1981, Y. Herrera 191 (CIIDIR); La Remuda, 17 km al SE de El Salto, 2800 m alt., 12 Sep 1982, Y. Herrera 219 (CIIDIR, NY); La Ciudad, en Mexiquillo, 8 km hacia los puentes, después del sexto tunel, [23.66666667N, 106.6833333W], 2700 m alt., 27 Feb 1999, L. López 3 (CIIDIR); Mexiquillo, pasando el cuarto tunel, en la cascada, La Ciudad, [23.71666667N, 105.6666667W], 2520 m alt., 16 May 2004, L. López et al. 244 (CIIDIR); km 95 carretera Durango–Mazatlán, [23.808889N, 105.337778W], 2570 m alt., 26 Sep 1997, A. Miranda 1373 (MEXU, US); km 109 carretera Durango–Mazatlán, [23.808889N, 105.337778W], 2570 m alt., 3 Dec 1997, A. Miranda 1390 (MEXU, US); El Salto (Aserraderos), [23.7833N, 105.367W], 2530 m alt., 28 Aug 1934, F.W. Penell 18366 (US); Sierra Madre Occidental, 0.4 mi W of Pericos, E of La Ciudad on highway 40, 23.7404N, 105.548W, 2804 m alt., 3 Oct 2007, P.M. Peterson and J.M. Saarela 21265 (US); Sierra Madre Occidental, W of Ciudad Durango, 5 mi W of El Salto, [23.82698808N, 105.3314386W], 2804 m alt., 27 Aug1958, J.R. Reeder and C.G. Reeder 3113 (MEXU); 3 mi E of El Salto, [23.80690869N, 105.3382346W], 2577 m alt., 13 Aug 1957, U.T. Waterfall and C. S. Wallis 13726 (F, TEX, US); km post #113 on highway 40 from Ciudad Durango to Mazatlán, ca. 12 km W of El Salto, 23.73318N, 105.46154W, 2700 m alt., 25 Jul 1985, G. Yatskievych et al. 85-243 (IND). **Municipio San Dimas**, ejido El Maguey, ladera al O del paraje Ojo del Becerro, 24.33333333N, 106.1166667W, 2100 m alt., 12 May 2005, S. González and J. Bacon 7039 (CIIDIR); 3 mi S of Guachichiles, upper slopes of cerro Huehento, 24.0786N, 105.743W, 3078–3249 m alt., 30 Sep 2008, P.M. Peterson and J.M. Saarela 22444 (US). **Municipio Santiago Papasquiaro**, lomeríos y márgenes del río Ramos, entre el poblado El Olote (J. Salomé Acosta) y el cañón de Pichagua, 25 km al N de Santiago Papasquiaro, [25.25199383N, 105.4504342W], 1650 m alt., 5 May 1994, R. Corral-Díaz et al. s.n. (CIIDIR); 45 km del entronque Los Herreras–Topia, rumbo a El Yaqui, 2710 m alt., 2 May 1994, R. Corral-Díaz et al. s.n. (CIIDIR); Santiago Papasquiaro, [25.02210854N, 105.4082118W], 1724 m alt., 1995, R. Corral-Díaz 6158 (MEXU); Los Altares, 5 km hacia Topia, bajada al río San Nicolás, [25.02833333N, 105.9455556W], 2710 m alt., 4 Aug 2015, L. López and E. Monreal 941 (CIIDIR). **Municipio Suchil**, potrero Los Anegados, 3 km de El Alemán, Reserva de la Biósfera, La Michilía, [23.30833333N, 104.17W], 2240 m alt., 11 Oct 1986, F. Acevedo 303 (CIIDIR); potrero Escobas, San Juan de Michis, [23.43144037N, 104.1267265W], 2218 m alt., 13 Jan 1986, J. Alvarado 758 (CIIDIR); arroyo San Pedro, Reserva de la Biósfera La Michilía, [23.37638889N, 104.2255556W], 2445 m alt., 14 May 1992, A. García 1325 (CHAPA, CIIDIR); alrededores de Laguna de Morrillos, [23.40946903N, 104.1716001W], 2560 m alt., 26 Oct 1985, S. González 3682 (CIIDIR, IEB, MEXU); rancho El Temascal, entre el arroyo El Olvido y el casco del rancho, [23.3855N, 104.2469W], 2482 m alt., 15 Jul 1985, Y. Herrera 653 (CIIDIR); Laguna del Coyote, 5 km de San Juan de Michis, hacia la Reserva de la Biósfera, [23.40155346N, 104.1732526W], 2368 m alt., 19 Oct 1992, Y. Herrera and C. González 1030 (CIIDIR). **Municipio Tepehuanes**, 27 km al NO de Santiago Papasquiaro, [25.18547524N, 105.6017025W], 2000 m alt., 16 Jul 1982, R. Hernández et al. 7980 (MEXU). **Without municipality**, state of Durango, P. Ibarra 377 (US); at Tobar [Tovar], 28 May 1906, E. Palmer 243 (NY, US); 6.5 mi W of El Santo, 2680, 30 Sep 1953, J.R. Reeder and C.G. Reeder 2540 (MEXU). **Jalisco: Municipio Bolaños**, km 30 al N de Bolaños, terracería a Tuxpan, [21.91839492N, 103.8832514W], 2600 m alt., 8 Oct 1981, Luquín et al. 145 (IBUG); 25 km al NO de Bolaños por la brecha a Los Asomoles, [21.90657051N, 103.8679489W], 2470 m alt., 9 Aug 1986, F.J. Santana 1777 (IBUG). **Municipio Mascota**, S de Mascota, [20.5087N, 104.79W], 1287 m alt., 18 Mar 1971, R. González 178 (MICH). **Municipio Mazamitla**, rancho El Tigre, Sierra de Mazamitla, 2700 m alt., 21 Sep 1986, R. Ramírez 571 (IBUG); cerro El Tigre, 5 km al SE de Mazamitla, 2700 m alt., 21 Sep 1986, R. Soltero 590 (IBUG). **México: Municipio Jilotzingo**, ejido Santa María Mazatla, [19.52562201N, 99.34846247W], 2756 m alt., 25 Jun 2011, J. Escutia and N. Ávila 12 (FCME). **Municipio San Felipe del Progreso**, 4.35 km SO de San José del Rincón, [19.65083333N, 100.1941667W], 2920 m alt., 20 Oct 1999, A. Ramínez 907 (MEXU). **Municipio Xalatlaco**, 4 km de Xalatlaco sobre la carretera Xalatlaco–Ajusco, [19.165N, 99.3675W], 3100 m alt., 18 Oct 1994, R. Mendoza 242 (MEXU). **Michoacán: Municipio Pátzcuaro**, cerro Burro, along the road to the microwave towers, 2.5 km (by road) N of Mex 41, [19.43503889N, 101.5121083W], 3270 m alt., V.W. Steinmann 2140 (IEB). **Morelos: Municipio Huitzilac**, shore of Laguna Zempoala, 20 km NW of Cuernavaca, [19.04991306N, 99.31567672W], 2800 m alt., 8 Dec 1950, N.C. Fassett 28438 (US). **Oaxaca: Municipio Heroica Ciudad de Tlaxiaco**, Colonia Adolfo López Mateos, 42 km de Puebla rumbo a Tlaxiaco, [17.3025N, 97.6678W, 2124 m alt.], 23 Jun 1980, A.A. Beetle M-4723 (MICH). **Municipio San Pedro Yólox**, 55 km de Guelataeo rumbo a Tuxtepec, [17.60051925N, 96.41986707W], 2110 m alt., 1 Jul 1980, A.A. Beetle M-5020 (MEXU). **Municipio Santa Catarina Ixtepeji**, 27 km de Oaxaca rumbo a Tuxtepec, [17.2461537N, 96.565485W], 2710 m alt., 1 Jul 1980, A.A. Beetle M-5006 (MEXU). **Municipio Santa María Jaltianguis**, 13 km después de Guelatao, [17.37081461N, 96.51327756W], 1 Jun 1980, A.A. Beetle M-4996 (MEXU). **Puebla: Municipio Zacatlán**, barranca de Zacatlán, 1900 m alt., 4 Aug 1981, S. Contreras 551 (MEXU). **Querétaro: Municipio Amealco**, El Aserrin, [20.29329964N, 100.2221305W], 2400 m alt., 19 Sep 1994, M. Ávalos 85 (IEB, MEXU). **Municipio Pinal de Amoles**, Puerto de Agua Fría, ca. 10 km al S de Pinal de Amoles, [21.14548889N, 99.57377778W], 2800 m alt., 5 Sep 1985, R. Fernández 3076 (IEB); caseta forestal, Puerto del Pino, [21.12612778N, 99.63825833W], 2620 m alt., 26 Nov 1981, A. Mora and J. Ramírez 467-AMB (MEXU); cerro del Pingüical, cañada del Agua Fría, 5.6 km from Mex 120 and 1.8 km from the road to Microondas Pingüical, [21.16722222N, 99.69238889W], 3000 m alt., V.W. Steinmann and S. Zamudio 2607 (IEB, NY). **San Luis Potosí: Municipio Guadalcázar**, near the Minas de San Rafael in the Sierra de Guadalcazar, 1900–2100 m alt., 3 Oct 1954, E.R. Sohns 1534 (US). **Municipio Villa de Arriaga**, in canyons in the Sierra de San Miguelito, ca 2 km W of Terrero,1850–2200 m alt., 8 Sep 1954, E.R. Sohns 1142 (US). **Without municipality**, San Luis Potosí, 1878, E. Palmer and C.C. Parry 923 (US), 1877, J.G. Schaffner 140 (US). **Sinaloa: Without municipality**, between Mazatlan and Durango, between La Fraguita and El Madroño, 6 Jan 1975, A.A. Beetle et al. M-3727 (MEXU). **Sonora: Municipio** **Agua Prieta**, Rancho Los Ojos, El Cajón Bonito, 50 km (by air) E of Agua Prieta, Cuenca Los Ojos Foundation conservation area, Sierra San Luis, 31.27803N, 109.00102W, 1297 m alt., 22 Apr 2017, S. Carnahan et al. 2395 (ARIZ, ASU); Punta del Agua, Cajón Bonito, Rancho la Victoria, 56.5 km (by air) ESE of Agua Prieta, Sierra San Luis, 31.22256N, 108.94292W, 1389 m alt., 23 Apr 2017, S. Carnahan et al. 2416 (ARIZ), 2417 (ARIZ, ASU). **Municipio** **Fronteras**, Sierra Buenos Aires, Upper Arroyo San Vicente; 53 km ESE (by air) of Cananea., 30.73342N, 109.82305W, 1714 m alt., 16 Aug 2016, S. Carnahan 1880 (ARIZ). **Municipio** **Imuris**, Cajón El Chorro, ca. 1.5 km southwest of cerro La Pirinola, W slopes of Sierra de la Madera, ca. 17.5 km (by air) E-NE of Magdalena, 30.66944444N, 110.7761111W, 1403 m alt., 28 Apr 2005, A.L. Reina and T.R. Van Devender 2005-766 (USON). **Municipio** **Nogales**, canyon on the northeast slopes of cerro Manzanero, near Rancho Esmeralda (Rancho Las Borregas), Sierra Las Avispas, 31.3417N, 111.175W, 1090 m alt., 24 Apr 2005, A.L. Reina et al. 2005-683 (ASU). **Municipio** **Yécora**, Ciénega de Camilo, near Arroyo, 28.43333333N, 108.5666667W, 1500 m alt., 20 Mar 1998, T.R. Van Devender s.n. (CIIDIR). **Veracruz: Municipio Ayahualulco**, entronque del camino a la toma de agua, entre El Triunfo–Los Laureles, 19.45N, 97.18333333W, 3000 m alt., 22 Nov 1995, J. Becerra et al. 348 (XAL). **Municipio Xalapa**, terreno de la SARH, km 4.6 carretera Xalapa–Veracruz, 19.50972222N, 96.86944444W, 1220 m alt., 31 Mar 1993, B.V. Hernández 166 (CIB, MEXU). **Zacatecas: Municipio General Francisco R. Murguía**, cañadas en la cima del cerro de la Virgen, [24.1594N, 103.18055W], 2450 m alt., 10 Aug 1988, J. Balleza 1580 (CHAPA). **Municipio Tlaltenango de Sánchez Román**, km 30 por la terracería entre Tlaltenango y Jalpa, [21.73333202N, 103.1849335W], 2500 m alt., 12 Jan 1990, J. Balleza 2721 (CHAPA). **USA. Arizona: Cochise County**, San Pedro River floodplain near Cascabel, 32.3045N, 110.391W, 933 m alt., 28 May 2001, K. Bagstad s.n. (ASU); Rustler Peak Quadrangle. Rustler Park, campground area, 1.9 km south of Barfoot Peak, 31.9N, 109.267W, 2465 m alt., 29 Aug 1999, M.A. Baker 13483 (ASU); Chiricahua Mountains, Barfoot Park, [31.915091N, 109.284506W], 2438–2515 m alt., 13 Sep 1906, J.C. Blumer 1374 (F); Cave Creek Public Camp, Chiricahua Mountains, [31.89904245N, 109.1622898W], 1507 m alt., Jul 1940, R.S. Ferris 9978 (MICH); Wickershams Cabin, Huachuca Mountains, [31.48889N, 110.4075W], 2180 m alt., 9 Aug 1909, L.N. Goodding s.n. (ASC); Sitgreaues National Forest, Chiricahua Mountains, Rucker Resevoir, [31.77975N, 109.30884W], 1847 m alt., 19 Jun 1997, K.D. Heil 10-969 (SJNM); Chiricahua National Monument, near permanent spring south of Silver Spur Meadow, [32.0044N, 109.356W], 1646 m alt., 10 Jun 1975, T. Reeves 2861 (ASU); Chiricahua National Monument, Lost Canyon near junction with Surprise Canyon, [32.0044N, 109.356W], 1859 m alt., 2 Jul 1975, T. Reeves 3263 (ASU); Chiricahua National Monument, near W boundary W of superintendent's residence, lower Bonita Canyon, [32.0044N, 109.356W], 1585 m alt., 13 Jul 1975, T. Reeves 3442 (ASU); Turkey Creek below highway 181, ca. 50 km (by air) ESE of Willcox, Sulphur Springs Valley, 31.88865N, 109.491417W, 1472 m alt., 8 May 2016, C.M. Roll 2056 (ARIZ); Dragoon Mountains, Slavin Gulch, 31.87535N, 110.02929W, 1463 m alt., 29 Mar 2017, J. Verrier 790 (ARIZ); Parker Canyon Cienega, down stream from Parker Canyon Lake dam, 31.42637N, 110.458218W, 1340 m alt., 10 May 2013, D. Wolkis and D. Setaro 618 (DES); Rustlers Park Recreation Area, 31.903882N, 109.279557W, 2591 m alt., 11 Sep 1982, R.D. Worthington 9068 (UTEP). **Graham County**, Pinaleno Mountains, Lower Jacobson Canyon, [32.6408N, 109.843W], 1219 m alt., 25 Jun 1976, S. Bingham 2310 (ASU); Laurel Canyon, E of canyon, 32.8818N, 110.304W, 1463 m alt., 22 May 1998, J.J. Buegge 211 (ASU); Coconino National Forest, Buford Canyon, 32.8529N, 110.218W, 1615 m alt., 6 Jun 1998, J.J. Buegge 324 (ASU); CCC Camp near Columbine, Pinaleno Mountains, [32.7055556N, 109.9091667W], 2591 m alt., 6 Sep 1942, R.A. Darrow s.n. (UTC); Pinaleno Mountains, Mount Graham, [32.71365N, 109.90179W], 2682 m alt., 2 Aug 1983, B.T. Johnson 11274 (ASU); 6 mi below Pine Crest, Graham Mountains, [32.6683333N, 109.8708333W], 1768 m alt., 19 May 1953, B. Maguire et al. 11466 (UTC); Marijilda Canyon, NE slopes, Graham Mountains, [32.6691667N, 109.8630556W], 2938 m alt., 2 Jun 1935, B. Maguire et al. 12048 (UTC); Mount Graham, Emerald Cienega, [32.694N, 109.894W], 3158 m alt., 6 Aug 1985, K. Reichhardt 85-142 (ASU); Pinaleno Mountains, Columbine Spring, E of Soldier Creek and NE of Soldier Creek campground, 32.703333N, 109.918333W, 2760 m alt., 8 Aug 2014, T.R. Van Devender s.n. (ASU); Pinaleno Mountains, Hospital Flat along Swift Trail in vicinity of Forest Service campground, ca. 11 mi SW of Safford, 32.66944N, 109.87275W, 2761 m alt., 9 Aug 2014, T.R. Van Devender s.n. (ASU). **Pima** **County**, Saguaro National Park (Rincon Mountains District), Rincon Creek, 32.157833N, 110.555484W, 1442 m alt., 20 Apr 2018, D. Beckman s.n. (ARIZ); Rincon Mountains, Manning Camp, [32.20952N, 110.553968W], 2438 m alt., 20 Sep 1909, J.C. Blumer 3385 (F); Mica Meadow, Rincon Mountains, [32.1319N, 110.519W], 2560 m alt., 23 Jul 1982, J.E. Bowers and S.P. McLaughlin R-365 (ASU); under rock overhang in streambed, Chimenea Canyon, Rincon Mountains, [32.2518N, 110.664W], 1393 m alt., 22 May 1982, J.E. Bowers et al. R-142 (ASU); Bear Wallow, Santa Catalina Mountains, [32.42450073N, 110.7396501W], 2391 m alt., 30 Aug 1973, M.R. Brand s.n. (USF); Mount Lemmon, Catalina Mountains, [32.5N, 110.921W], 2286 m alt., 12 Oct 1940, M.H. Buehler s.n. (ASU); Santa Catalina Mountains, Bear Canyon, between upper picnic area and lowest crossing of the highway, [32.3667N, 110.708W], 1699 m alt., 21 May 1995, M. Fishbein et al. 2370 (ASU); Arizona National Scenic Trail, Santa Catalina Mountains, Coronado National Forest, WSW of Bellota Ranch, 32.321833N, 110.648033W, 1170 m alt., 28 Apr 2005, W.C. Hodgson et al. 20206 (ARIZ, DES); Molino Basin, [32.337032N, 110.69357], 1321 m alt., 14 May 1966, D.J. Keil 351 (OBI); 3.5 mi below Molino Basin Campground, Santa Catalina Mountains, [32.2848N, 110.696W], 1158 m alt., 18 Apr 1969, D.J. Keil and M. Lucklow 4296 (ASU); upper Bear Canyon, Santa Catalina Mountains, [32.4303N, 110.705W], 1969 m alt., 24 May 1989, R. Perrill and R. Duncan 5895 (ASU); E end of Rincon Mountains, 4 km north of Pima-Cochise county line along USFS-35 (Mescal Road), rocky canyon of Ash Creek, [31.8856N, 111.219W], 1460 m alt., 1 May 1987, J.R. Reeder and C.G. Reeder 8029 (ASU); Tucson, [32.221743N, 110.926479W], 758 m alt., 1892, J.W. Toumey s.n. (NY); Santa Catalina Mountains, Hollin Canyon, 32.34298N, 110.6892W, 1556 m alt., 24 Apr 2017, J. Verrier 981 (ARIZ); Santa Catalina Mountains, Willow Canyon, 32.477722N, 110.722389W, 2134 m alt., 24 Aug 2011, J.F. Wiens and J.H. Wiens 2011-065A (ASDM). **Pinal County**, San Pedro River floodplain near Cascabel, 32.9842N, 110.783W, 900 m alt., 29 May 2001, K. Bagstad s.n. (ASU). **Santa Cruz County**, Pena Blanca Lake, near boat ramp at S end of lake, E bank, boggy area, [31.3979N, 111.088W], 1167 m alt., 25 May 1991, M.A. Baker 8383 (ASU, DES); unnamed canyon between Temporal Gulch Trail and Josephine Canyon, Santa Rita Mountains, Coronado National Forest, 31.67747N, 110.85677W, 1929 m alt., 27 May 2018, S. Carnahan and C. Smith 3094 (ARIZ); Santa Cruz, Sycamore Cyn in Atacosa Mountains, NW of Nogales, [31.43268553N, 111.1883009W], 1219 m alt., 29 Dec 1995, D.J. Keil and M. Lucklow 19052 (OBI); Santa Rita Mountains, Baldy Saddle Trail, 31.69533N, 110.83703W, 2484 m alt., 17 Sep 2017, J. Verrier and S. Carnahan 1233 (ARIZ). **New Mexico: Grant County**, Gila National Forest, Sawmill Canyon, 32.57077N, 108.475376W, 1875 m alt., 18 Jul 1987, C.S. Lieb 867 (UTEP); Mimbres River, [32.858382N, 107.977694W], 1676 m alt., 1 Jul 1940, O.B. Metcalfe 1064 (F). **Hidalgo County**, Gray Ranch, Double Adobe Creek, [31.656N, 108.74W, 1607 m alt.], 29 Jun 1993, D. Bleakly 1122a (UNM); Peloncillo Mountains, Lower Lee, just below old corral, 31.713867N, 108.944117W, 1678 31.656N, 108.74W, 27 Aug 2008, E. Makings and W. Moyer 3069 (DES); Peloncillo Mountains, Miner canyon, The Bioresearch Ranch, near Maverick Spring, 31.7027N, 108.90014W, 1645 m alt., 12 May 2004, W.H. Moir s.n. (ASC); rocky slopes of Lower Indian Creek Canyon in the Animas Mountains, [31.61275N, 108.7798W], 1767 m alt., 21 May 1975, W.L. Wagner 782 (UNM); Animas Mountains, Upper Indian Creek Canyon, 31.59389548N, 108.7695189W, 2042 m alt., 17 Jun 1975, W.L. Wagner 1010 (UNM); Coronado National Forest, along Cloverdale Creek, ca. 1 mi. SE Pendleton Ranch House, 31.43664N, 108.973867W, 1631 m alt., 17 May 1986, R.D. Worthington 14085 (UTEP); Peloncillo Mountains, FSR 63 in Clanton Draw, 3 mi E of crest, 31.54249024N, 108.8786469W, 1524–1676 m alt., 31 May 1997, R.D. Worthington 26546 (UTEP). **Lincoln County**, Sierra Blanca ski area, [33.396441N, 105.8013W], 3108 m alt., 9 Jul 1977, S.L. Cox and W. Haggren 647 (UNM), W. Haggren 329 (UNM), 414 (UNM), W. Haggren and P.J. Knigh 205 (UNM), 7 Aug 1977, W. Haggren, W. and S.L. Cox 771 (UNM), G.T. Manthey and W. Haggren 1954 (UNM); about 2 mi N of Sierra Blanca Peak, Sacramento Mountains, Lincoln National Forest, [33.4N, 105.816667W], 3048 m alt., 7 Sep 1966, W.H. Moir 66-30 (COLO); White Mountains, [33.4833333N, 105.7972222W], 2134 m alt., 5 Aug 1897, E.O. Wooten 282 (UTC); White Mountains, 2 mi W Sierra Blanca, ski lodge at Apache Bowl ski area, 33.397877N, 105.825W, 3414 m alt., 16 Aug 1980, R.D. Worthington 6364 (COLO, UTEP); White (Sacramento) Mountains, about 1 mi NNW top Sierra Blanca peak near Lookout Mountain vista point, 33.3977N, 105.825W, 3350 m alt., 28 Jul 1991, R.D. Worthington 19638 (DES, NY). **Otero County**, Bluff Springs, Sacramento Mountains, Potato Canyon, 850 m NE of its confluence with Pepper Canyon, 8km WSW of Sacramento, 32.7683N, 105.64W, 2415 m alt., 19 Aug 2005, M.A. Baker 16046 (UNM); 6 mi SE of Cloudcroft, [32.9148333N, 105.6946667W], 2629 m alt., 6 Aug 1976, S.L. Hatch 2204 (UTC); Sacramento Mountains Apache Bowl Ski Area, 1 mi N Sierra Blanca Peak and trail down canyon, 33.388756N, 105.809433W, 3454 m alt., 22 Jul 2001, R.D. Worthington 30647 (SAT). **Sierra County**, Gila National Forest, Taylor Creek, 32.727512N, 107.688329W, 1829 m alt., 13 Aug 1982, T.R. Van Devender et al. 85 (UTEP). **Texas:** **Brewster County**, Chisos Mountains, 17 Aug 1931, C.H. Mueller 7951a (TEX); in bed of small stream, Hidden Valley, near Alpine, 20 Jun 1941, R. Rose-Innes and B.R. Moon 1263 (TEX). **El Paso County**, Hueco Tanks State Park and Historic Site, NE side of North Mountain, 31.92361111N, 106.0425W, 1395 m alt., 7 Jul 1979, R.D. Worthington 4748 (COLO). **Jeff Davis County**, in upper Madera Canyon on the SW slope of Mount Livermore in the Davis Mountains, 2134 m alt., Aug 1936, L.C. Hinckley 618 (SRSC); in upper Madera Canyon, Mount Livermore Quadrangle, 30.636944N, 104.17W, 2346 m alt, 20 Jun 2012, J.J. Keeling 222 (SRSC); in damp shaded areas near top of Mount Livermore, Davis Mountains, 2438 m alt., 11 Sep 1947, B.H. Warnock 7433 (ARIZ, LL); in Limpia Canyon, 1 mi N of Wild Rose Pass, Kokernot ranch, Davis Mountains, 1676 m alt., 6 Apr 1948, B.H. Warnock 7751 (SRSC); in upper Madera Canyon of Mount Livermore, Davis Mountains, 2286 m alt., 23 Aug 1968, B.H. Warnock 23013 (SRSC); Limpia Creek at Old Mcguire Ranch Ruins, Davis Mountains Resort, 30.625N, 104.0916667W, 25 Jun 1994, R.D. Worthington 23177 (UTEP). **Kenedy County**, King's Ranch, near Norias, 15 Mar 1942, C.L. Lundel and A.A. Lundell 10741 (MEXU).

***Agrostis tolucensis***

**GUATEMALA. Sacatepéquez: Municipio Alotenango**, cráter del Volcán Acatenango, [14.499453N, 90.87175W, 4035 m alt.], 7 Feb 1907, W.A. Kellerman 6234 (MEXU); Volcán de Acatenango, 14.499453N, 90.87175W, 3950 m alt., 11 Apr 2000, M. Véliz et al. 8406 (MEXU), 22 Aug 2000, M. Véliz et al. 10282 (MEXU). **Municipio Santa María de Jesús**, Volcán de Agua, 14.468149N, 90.736509W, 3400 m alt., 24 Aug 2000, M. Véliz et al. 10312 (MEXU), 10364 (MEXU). **San Marcos: Municipio Sibinal**, Volcán Tacaná, 15.13055556N, 92.10694444W, 3900 m alt., 7 Nov 2000, M. Véliz, et al. 10521 (MEXU), 10539 (MEXU). **Municipio Tajumulco**, Volcán Tajumulco, Tuichán, 15.06861111N, 91.87305556W, 3083 m alt., 26 May 2000, N. Gallardo et al. 9075b (MEXU), 7 Sep 2000, M. Véliz et al. 10387 (MEXU), 10394 (MEXU). **MEXICO: Chiapas: Municipio Unión Juárez**, cráter del Volcán Tacaná, [15.09306218N, 92.08369755W], 4000 m alt., 9 Oct 1987, E. Martínez 20870 (MEXU). **Durango: Municipio Suchil**, Reserva de la Biosfera La Michilia, G. Flores s.n. (CIIDIR). **Guerrero: Municipio General Heliodoro Castillo**, cerro Teotepec, [17.46666667N, 100.2166667W], 3200 m alt., 24 Aug 1999, E. Domínguez 866 (MEXU), 11 Apr 1963, J. Rzedowski 16485 (ENCB), 5 Dec 1963, 18147 (ENCB), 10 Aug 1964, 18558 (ENCB); vertiente SO del cerro Teotepec, [17.48333333N, 100.2W], 3400 m alt., 29 Jan 1965, J. Rzedowski and R. McVaugh 213 (ENCB). **Jalisco: Municipio Ciudad Guzmán**, 1 km hacia debajo de La Cabaña, camino a Ciudad Guzmán, 3220 m alt., 3 Feb 1994, Grupo de ordenamiento ecológico s.n. (IBUG). **Municipio Cuautitlán de García Barragán**, cerro de las Capillas, Sierra de Manantlán, [19.55592982N, 104.1476287, 2856 m alt.], 10 Oct 1980, R. Guzmán 895 (MEXU); top of sharp crest of the Sierra de Manantlán Oriental, just E of cerro Las Capillas, along road from La Cumbre to Los Jardines, 19 km due SSE of El Chante, 19.55416667N, 104.15W, 2860 m alt., 7 Jan 1980, H.H. Iltis et al. 2417 (IBUG). **Municipio San Gabriel**, N slopes of Nevado de Colima, [19.60110761N, 103.5808018W, 3000 m alt.], 19 Sep 1980, A.A. Beetle and R. Guzmán M-5400 (IBUG, MEXU), M-6084 (IBUG, MEXU); ladera de exposición N del Nevado de Colima, [19.60110761N, 103.5808018W], 3000 m alt., 10 Nov 1984, R. González 13 (IBUG), 14 Nov 1984, 34 (IBUG), 54 (IBUG); NE slopes of the Nevado de Colima, above Canoa de Leoncito, [19.6167N, 103.65W], 3100–3300 m alt., 13 Sep 1952, R. McVaugh 12902 (US); Parque Nacional Nevado de Colima, near 17.5 km mark from entrance up road to Observatorio Vulcanológico, 19.59138889N, 103.5986111W, 3440 m alt., 23 Sep 2005, P.M. Peterson et al. 19092 (CIIDIR). **Municipio Tonila**, camino El Fresnito–antenas de microondas, Nevado de Colima, 3400 m alt., 7 Feb 1987, S. González et al. 2934 (IBUG). **Municipio Tuxpan**, 28.3 mi SW of Ciudad Guzman, along ridge just near Microondas at Nevado de Colima, 19.566N, 103.617W, 4007 m alt., 6 Oct 2001, P.M. Peterson and O. Rosales 16101 (US). **Municipio Tuxpan**, Volcán de Fuego, 20 Mar 1965, L.M. Villareal s.n. (IBUG). **Without municipality**, Mt. Nevada, 23 Sep 1910, A.S. Hitchcock 7157 (MEXU). **México: Municipio Amecameca**, Iztaccihuatl, S side of mointain, [19.089948N, 98.645973W], 4050 m alt., 1 Aug 1958, J.H. Beaman 1990 (TEX); S side of Iztaccíhuatl, 0.5 km NE of the summit of Pies, [19.089948N, 98.645973W], 4300–4350 m alt., 16 Sep 1958, Beaman 2553 (MEXU, US); La Joya de Alcalican, extremo SW del Iztaccíhuatl, [19.141667N, 98.675W], 3900 m alt., 26 Nov 1978, V. Benhumea 69a (CHAPA), 14 Sep 1976, A. Castellanos s.n. (CHAPA); Contrafuertes de la Joya, Iztaccíhuatl, [19.136596N, 98.650187W], 3820 m alt., 8 Oct 1955, R.T. Clausen s.n. (MEXU); La Joya de Alcalican, extremo SW del Iztaccíhuatl, [19.141667N, 98.675W], 3900 m alt., 27 Mar 1976, J.M. Díaz 3860 (CHAPA); La Joya, 1.5 km al N de Tlalmanalco, [19.141667N, 98.675W], 3900 m alt., 26 Oct 1986, P. Escalera 78 (IBUG); La Joya de Alcalican, extremo SW del Iztaccíhuatl, [19.141667N, 98.675W], 3900 m alt., 26 Nov 1978, A. García 139a (CHAPA); ladera S del Volcán Iztaccíhuatl, 19.13336111N, 98.63980556W, 3995 m alt., 13 Sep 2013, R. Hernández-Cárdenas and L. Arredondo-Amezcua 1342 (IEB), 1366 (IEB); La Joya, Volcán Iztaccíhuatl, 7 km al N de la carretera pavimentada a Amecameca, 13 km al E de Amecameca, [19.141667N, 98.675W], 3920–4000 m alt., 28 Oct 1976, S.D. Koch 76232 (CHAPA, US); carretera al Paso de Cortés, 16 km al E del entronque con la carretera Amecameca-Cuautla, 9 km al ESE de Amecameca, [19.09095877N, 98.68123042W], 3300 m alt., 20 Nov 1976, S.D. Koch 76272 (CHAPA, US); Iztaccíhuatl, falda SW, [19.07592501N, 98.71272144W], 3800 m alt., 1 Dec 1952, E. Matuda 26103 (MEXU, US); Paso de Cortés, Parque Nacional Izta-Popo, [19.08873853N, 98.67164797W], 3445 m alt., 2 Oct 1992, A. Miranda and G. Villegas 646 (MEXU); km 15 carretera Amecameca–Tlamacas, [19.08516405N, 98.68113865W, 3288 m alt.], 2 Oct 1992, A. Miranda and G. Villegas 633 (MEXU); estribaciones del Popocatépetl, 3900 m alt, 2 Dec 1979, R. Rodríguez 124 (CHAPA, CIIDIR). **Municipio Atlautla**, N side of Popocatepetl, 11 Sep 1957, J.H. Beaman 1731 (US); carretera al Paso de Cortés, 13 km al E del entronque con la carretera Amecameca-Cuautla, 10 km al ESE de Amecameca, [19.07692613N, 98.68826854W], 3230 m alt., 5 Nov 1976, S.D. Koch 76251 (CHAPA); campamento Tlamacas, [19.06653N, 98.629409W, 3988 m alt.], 20 Nov 1983, E. López PT-303 (MEXU); Popocatépetl, Cañada del Ventorrillo, [19.04781N, 98.65419W], 4080 m alt., 18 Jan 1985, I. Luna and L. Almeida s.n. (FCME); **Municipio** **Chalco**, Llano Grande, 3150 m alt., 21 Aug 1966, J. Rzedowski 22973 (MEXU). **Municipio Isidro Fabela**, alrededores de la presa Iturbide, 34 km al WNW de Naucalpan (6 km al Sw de Santiago Tlazala, por la carretera), [19.52454734N, 99.46880154W], 3350 m alt., 7 Aug 1977, S.D. Koch 77141 (CHAPA, US). **Municipio Ixtapaluca**, Estación Experimental de Investigación y de Enseñanza de Zoquiapan, km 50 carretera México-Puebla, [19.280108N, 98.671828W], 3400 m alt., 12 Mar 1978, E. Calderón 67 (CHAPA); ladera de San Francisco, 1.5 km al SW de la Estación Forestal Experimental Zoquiapan, [19.26833333N, 98.66722222W], 3345 m alt., 25 Aug 2000, E. Guízar 5097a (MEXU); Estación Experimental de Investigación y de Enseñanza de Zoquiapan, 8 km de Río Frío, lado N del Llano Aculco, [19.29079527N, 98.67281007W], 3250 m alt., 21 Aug 1975, S.D. Koch 75414 (CHAPA); Estación Experimental de Investigación y de Enseñanza de Zoquiapan, 8 km de Río Frío, entronque del camino 3 y 4, [19.28697457N, 98.67034008W], 3240 m alt., 5 Sep 1975, S.D. Koch 75484 (CHAPA); Estación Experimental de Investigación y de Enseñanza de Zoquiapan, 8 km de Río Frío, Llano Aculco, [19.29079527N, 98.67281007W], 3200 m alt., 30 Oct 1975, S.D. Koch 75683 (CHAPA, US), 75692 (US); Estación Experimental de Investigación y de Enseñanza de Zoquiapan, 8 km de Río Frío, [19.280108N, 98.671828W], 3450 m alt., 12 Mar 1978, R. Vega 32 (CHAPA); Estación Experimental de Investigación y de Enseñanza de Zoquiapan, 8 km de Río Frío, camino 4, cerro Tres Cruces, [19.28697457N, 98.67034008W], 3550 m alt., 30 Jul 1978, R. Vega 363 (CHAPA); Estación Experimental de Investigación y de Enseñanza de Zoquiapan, 8 km al S de Río Frío, camino 3, al S de Llano Aculco, [19.29079527N, 98.67281007W], 3400 m alt., 20 Aug 1978, R. Vega 435 (CHAPA). **Municipio Ocoyoacac**, 1 mi S of the village of Ocoyoacac, [19.24838151N, 99.44116306W], 2600 m alt., 17 Oct 1953, E. Sohns 986 (MEXU, US). **Municipio San Lucas del Pulque**, San Lucas del Pulque, [19.11165251N, 100.0267285W], 2266 m alt., 30 Jul 1987, L. Olguin 18 (MEXU). **Municipio San Simón de Guerrero**, 5 km de Buenavista, rumbo a Temascaltepec, 3100 m alt., 15 Jan 1983, E. Manrique et al. 70 (MEXU); 7 km de Buenavista, rumbo a Temascaltepec, 3250 m alt., 29 Aug 1983, E. Manrique et al. 281 (IEB, MEXU); 14 km de Buenavista, rumbo a Temascaltepec, 3010 m alt., 29 Aug 1983, E. Manrique et al. 295 (MEXU). **Municipio Temascaltepec**, Las Cruces, [19.18596N, 99.85251W], 3350 m alt., 21 Sep 1932, G.B. Hinton 1713 (MEXU, US); 1 km después de Cajones, rumbo a Sultepec, [19.04436944N, 99.875W], 3060 m alt., 7 Feb 1984, E. Manrique et al. 660 (MEXU); 27 km SW of Toluca on road to Temaxcaltepic [Temascaltepec], W facing slope, [19.11N, 100W], 2800 m alt., 9 Jul 1964, G. Mick and K.E. Roe 230 (US). **Municipio Texcoco**, Tlaloc, near summit of mountain, [19.48275708N, 98.70691757W], 4100 m alt., 22 Aug 1958, J.H. Beaman 2346 (MEXU, TEX); 26 km de Texcoco, sobre brecha madedera que va hacia Sierra Tláloc, [19.48275708N, 98.70691757W], 3600 m alt., 10 Nov 1975, E. García s.n. (CHAPA); cerro Tláloc, [19.48275708N, 98.70691757W], 3860 m alt., 1 Nov 1984, M. González and J. García 147 (CHAPA); vertiente W del cerro Tláloc, [19.45081577N, 98.71004541W], 3700 m alt., 15 Oct 1985, M. González and P. Vera 179 (CHAPA); cerro Tláloc, 18 km al E de Tequexquinahuac, sobre la brecha maderera a la cima, 17 km al ESE de Texcoco, [19.48275708N, 98.70691757W], 3540 m alt., 26 Oct 1976, S.D. Koch 76229 (CHAPA, US); cerro Tláloc, Sierra Nevada, base del precipicio vertical entre los dos picos en zona alpina, frente al E, 19.4N, 98.71666667W, 3950 m alt., 20 Sep 1981, T. Wendt and T. Atkinson 3429 (CHAPA); cerro Tláloc, Sierra Nevada, cuesta pedregosa en parte inferior occidental, 19.4N, 98.71666667W, 3920 m alt., 20 Sep 1981, T. Wendt and T. Atkinson 3445 (CHAPA). **Municipio Tlalmanalco**, La Ciénega, región de Peñas Cuatas, cerca de la cabeza del Iztaccíhuatl, [19.668889N, 99.328611W], 3650 m alt., 19 Aug 1984, S. Acosta s.n. (IBUG, MEXU); Apitza, Mt. Ixtaccihuatl [Iztaccíhuatl], [19.1833N, 98.65W], 3871 m alt., 27 Jul 1938, E.K. Balls 5132 (US); San Rafael, falda W del Ixtaccihuatl [Iztaccíhuatl], [19.21032338N, 98.74569839W], 2900 m alt., 16 Nov 1952, E. Matuda 27612 (US), 27615 (US), 27619 (MEXU, US). **Municipio Toluca**, Nevado de Toluca, shore of large lake in the crater, [19.1014N, 99.7694W], 4140 m alt., 22 Jul 1958, J.H. Beaman 1895 (MEXU, US); Nevado de Toluca, summit of E rim of crater, [19.1014N, 99.7694W], 4170 m alt., 6 Sep 1958, J.H. Beaman 2483 (US); cráter de la Laguna del Sol, parte alta del Nevado de Toluca, [19.10496084N, 99.75259522W], 4100 m alt., 7 Sep 1982, R. Guzmán 6019a (MEXU); orillas de la Laguna del Sol, dentro del cráter del volcán Nevado de Toluca, [19.108075N, 99.768824], 4200 m alt., 2 Oct 1987, R. Méndez 183 (MEXU); 100 m al S de la laguna "La Luna", del Nevado de Toluca, [19.105556N, 99.751667W], 4240 m alt., 17 Sep 1997, A. Ramírez 766 (MEXU); **Municipio Villa del Carbón**, cerro la Bufa, [19.66649556N, 99.55298776W], 2900 m alt., 22 Nov 1953, E. Matuda 29745 (MEXU, US). **Municipio Zinacantepec**, 3 km al N de Volcán Gordo, [19.13769411, 99.82736448W], 3300 m alt., 29 Sep 1987, J. García 8 (MEXU); entre Raíces y Las Peñas, al S de Toluca, [19.15541999N, 99.82741468W], 3280 m alt., 1 Aug 1981, R. Guzmán 3992 (MEXU); crucero Sultepec–camino al Nevado de Toluca, [19.15229441N, 99.8044558W], 3538 m alt., 7 Sep 1982, R. Guzmán 6005 (MEXU); ascenso al Nevado de Toluca, camino nuevo que entronca con el de Zinancatepec, 4100 m alt., 5 Sep 1958, E. Hernández X. X-10190 (CHAPA, MEXU), X-10191 (CHAPA), X-10192 (US); 600 m al NW de la carretera relieve N del Nevado de Toluca, [19.141667N, 99.775W], 3900 m alt., 26 Oct 1972, A. Hernández S-4 (MEXU); Crucero Agua Blanca, [19.07841941N, 99.84049741W, 3169 m alt.], 26 Oct 1933, G.B. Hinton 4933 (US); camino al cráter del volcán, 3.5 km NW de estación de microondas, 19.13892417N, 99.78963W, 3801 m alt., 22 Oct 2003, A. Ibelles 3 (INEGI); ladera SW del Nevado de Toluca, 4200 m alt., 19 Oct 1985, D. Madrigal and A. González MAGT-485 (FCME); camino al albergue del Nevado de Toluca, [19.120168N, 99.751533W], 3640 m alt., 7 Feb 1984, E. Manrique et al. 650 (MEXU); 2 km antes de caseta de vigilancia, Nevado de Toluca, 19.12638889N, 99.77W, 3850 m alt., 16 Jul 2002, J. Martínez et al. 436 (INEGI); W slopes of Nevado de Toluca, 3700 m alt., 3 Jul 1964, G. Mick and K.E. Roe 193 (US); camino al Nevado, [19.1225N, 99.77888889W], 3200 m alt., 1 Oct 1992, A. Miranda et al. 600 (MEXU); Parque Nacional Nevado de Toluca, 4.6 mi above park gate, S of Nevado Toluca, 19.1426N, 99.7806W, 3789 m alt., 9 Oct 2007, P.M. Peterson et al. 21338 (US); 4.93 km al SE de Ojo de Agua, [19.17861111N, 99.74277778W], 3368 m alt., 11 Nov 1995, A. Ramírez 673 (MEXU); 1.65 km al SW de la comunidad La Ciervita, [19.17916667N, 99.78638889W], 3388 m alt., 12 Nov 1995, A. Ramírez 684 (MEXU); estación meterológica, Nevado de Toluca, [19.120833N, 99.753333W], 4110 m alt., 17 Sep 1997, A. Ramírez 771 (MEXU), 773 (MEXU); W slope of Nevado de Toluca, 2300 m alt., 16 Jul 1954, G.B. Van Schaack 3387 (CHAPA), 3391 (IBUG). **Without municipality**, Izta-Popo National Park at treeline, 8 Jul 1980, A.A. Beetle M-5195 (CHAPA, IBUG, MEXU); Iztaccíhuatl, Falda W, 3200 m alt., W. Boege 943 (MEXU); 12.5 mi E of turnoff to summit of Popocatépetl, 3460 m alt., 18 Oct 1976, J. Brunken and C. Perino 418 (CHAPA); on Mt. Popocatepetl, 7 Dec 1947, D.S. Correll 14310 (US); Entre Las Juntas y Tescaltitlán, al S del Nevado de Toluca, 1 Aug 1981, R. Guzmán, 4008 (MEXU); Nevado de Toluca, 3780 m alt., Sep 1958, Hernández X. X-10173 (CHAPA); Mt. Popocatepetl, 3658 m alt., Aug 1910, A.S. Hitchcock 5991 (US); Nevado de Toluca, 3800 m alt., 16 Jul 2002, J. Martínez et al. 441 (INEGI, MEXU); Acatzingo, cerca de Ozumba, 1900 m alt., 13 Sep 1953, E. Matuda 28951 (MEXU, US); Nevado de Toluca, 3000–4200 m alt., 18 Oct 1953, E. Matuda 29493 (US), 29500 (US), 1 Oct 1992, A. Miranda et al. 603a (MEXU); Cima, 24 Aug 1910, C.R. Orcutt 3789 (US); 1.5 km al E de Corral de Piedra, 2850 m alt., 27 Aug 1975, U. Pantoja s.n. (INEGI); Sierra de Las Cruces, 28 Aug 1892, C.G. Pringle 4219 (US); Nevado de Toluca, 4115 m alt., 7 Sep 1892, C.G. Pringle 5202 (MEXU, US); Ixtaccihuatl [Iztaccíhuatl], 3658–4267 m alt., 1903, C.A. Purpus 224 (US), 229 (US), 1643 (US); Nevada [Nevado] de Toluca, 3353–3962 m alt., 16 Oct 1903, J.N. Rose and J.H. Painter 8016 (US); Popocatepetl, 1100 m alt., Aug 1901, J.N. Rose and R.H. Hay 6296 (US); Nevado, 3800 m alt., 17 Sep 1930, M. Saint-Pierre 908 (US). **Mexico City: Alcaldía Cuajimalpa**, Puerto Las Cruces, [19.28971944N, 99.34563889W], 3220 m alt., 13 Aug 1985, M. Álvarez 9 (IEB, MEXU); Desierto de los Leones, 23 Sep 1938, E. Lyonnet 2601 (US), 1 Jan 1952, E. Matuda 25924 (MEXU, US). **Alcaldía Magdalena Contreras**, Cañada de Vista Alegre, [19.3211N, 99.2209W], 2600 m alt., 2 Dec 1951, E. Matuda 25796 (MEXU), 25812 (MEXU, US), 25820 (MEXU); Contreras, Cañada, [19.3N, 99.2833W], 2700 m alt., 1930, M. Saint-Pierre 913 (US). **Alcaldía Milpa Alta**, faldas del volcán El Cilcuayo, [19.103N, 98.988W], 3250 m alt., 17 Nov 1992, R. Giles 31 (MEXU). **Alcaldía Tlalpan**, volcán Pelado, [19.151N, 99.2171W], 3100 m alt., 29 Nov 1986, G. Campos et al. PEL-NE-31-31 (FCME); Parque Nacional Cumbres del Ajusco, cordillera central, ruta que sube al Pico del Águila, [19.21275782N, 99.26823297W], 3745 m alt., 21 Jul 2014, A. Rincón and C. Gómez 4060 (MEXU); volcán Pelado, [19.151N, 99.2171W], 340 m alt., R. Romero and G. Campos SW-34 (FCME [*]); al S del Xitle, [19.24328N, 99.221413W, 3000 m alt.], 30 Oct 1962, J. Rzedowski 2072 (MEXU); volcán Pelado, [19.151N, 99.2171W], 3370 m alt., 8 Aug 1987, Sandoval 240 (MEXU), s.n. (MEXU). **Michoacán: Municipio Angangueo**, camino al vivero Sierra Chincua, 2980 m alt., 19 Jan 1988, M. Mejía 2980 (IEB); alrededores del Llano de las Papas, [19.65618889N, 100.2717278], 3200 m alt., 9 Oct 1988, J. Rzedowski 47404 (CHAPA, CIIDIR, IEB, MEXU); Sierra Chincua, 3200 m alt., 30 Oct 2005, M.A. Salinas and J. Martínez 546 (MEXU). **Municipio Hidalgo**, ladera W de la cima del cerro San Andrés, 19.805N, 100.598055W, 3568 m alt., 7 Aug 2010, G. Aguilar et al. 50 (XAL); ladera N de la cima del cerro San Andrés, alrededor de las antenas, 19.80584167N, 100.5960111W, 3600 m alt., 6 Oct 2010, G. Aguilar and S. Zamudio 177 (MEXU). **Municipio Huiramba**, parte alta del cerro Burro, [19.43503889N, 101.5121083W], 3300 m alt., 23 Nov 1986, J. Rzedowski 41921 (ENCB, IEB), 3108 (CHAPA, IEB, MEXU); **Municipio Morelia**, parte alta del cerro del Águila, [19.62259044N, 101.3636366W], 3300 m alt., 24 Aug 1980, E. García et al. 3108 (MEXU). **Municipio Tancítaro**, Mt. Tancitaro, 3048, 22 Jul 1941, W.C. Leavenworth 1112 (US). **Municipio Zinapécuaro,** 1 km al E de Agua Fría, 2900, 1 Jul 1985, J. Almazán et al. 323 (IEB); cerro San Andrés, [19.80527778N, 100.5972222W], 3400 m alt., 13 Sep 1991, E. Pérez-Cálix 2482 (IEB). **Morelos: Municipio Huitzilac**, 2.88 km lineales del NE del puente Paso Morelos, 19.08839722N, 99.18872778W, 3061 m alt., 20 Nov 2012, L. Hernández 68 (HUMO), 28 Aug 2012, F. Hinterholzer 230 (HUMO); 55 km SE of Mexico City, [19.08489002N, 99.31190521W], 3200 m alt., 14 Jul 1942, J.N. Weaver 792 (US). **Municipio Oaxtepec**, Oaxtepec, [18.90219847N, 98.96287658W], 1333 m alt., 7 Dec 1952, F.G. Harking 660 (US). **Without municipality**, Volcán Las Palomas, NO de Morelos, 22 Nov 2007, T. Amezcua et al. 60 (MEXU). **Oaxaca: Municipio Teposcolula**, cerro El Garabatal, al NNW de San Pedro Nopala, [17.81304114N, 97.55596651W], 2610 m alt., 9 Sep 1990, J.G. Sánchez-Ken 128 (MEXU). **Puebla: Municipio Cholula**, La Ventana, lado Poniente, Santiago Xalitzintla, [19.09166667N, 98.34W], 3200–3300 m alt., 2 Dec 1987, M. Tlapa and C.Ubirna 1608 (MEXU). **Municipio San Nicolás de los Ranchos**, SE Iztaccíhuatl, [19.12916319N, 98.61736513W], 3835 m alt., 25 Nov 1993, A.M. Cleef et al. 17 (MEXU); 8 km de Xalitzintla rumbo a México por el Paso de Cortés, [19.07957114N, 98.56612932W], 2900 m alt., 31 Jul 1981, S. Morales 3 (MEXU); extremo S del volcán Iztaccíhuatl, cerca de la repetidora de televisión, [19.105N, 98.6214W], 3850 m alt., 22 Nov 1964, J. Rzedowski 19154 (MEXU). **Municipio San Salvador el Verde**, Ladera E del Iztaccihuatl, cerca de San Agustín, W de Texmelucan, [19.233333N, 98.606111W], 1650 m alt., 7 Oct 1968, W. Boege 242 (MEXU). **Municipio Tlachichuca,** alpine zone of Pico de Orizaba (Volcan Citlaltepetl), Jagged rock outcrop just SE of base of cone of peak ca. several hundred feet below permanent snow line, area with caves and shrine, ca. 1 km SE of cabin, [19.033058N, 97.272847W], 4267 m alt., 1 Aug 1977, T. Reeves et al. R-5818 (ASU). **Without municipality**, Ixtaccihuatl [Iztaccíhuatl], Oct 1905, C.A. Purpus 1643 (F); km 15 carretera Juchitán–Puebla, 11 Aug 1976, J.J. Soto s.n. (IBUG). **Tlaxcala: Municipio Chiautempan**, ladera E de la Malinche, [19.24297169N, 97.9789338W], 3600 m alt., 8 Oct 90, E. Acosta 3999 (MEXU), 4012 (MEXU); ladera N de La Malinche, [19.23705301N, 98.01935192W], 3980 m alt., 8 Oct 1986, L. Aragón et al. 9 (MEXU); 200 m de la estación de microondas rumbo a Tlaxcala, [19.258333N, 98.0325W], 3000 m alt., 5 Sep 1981, S. Contreras 101 (MEXU), 597 (MEXU). **Municipio Huamantla**, Volcán La Malinche, 4200 m alt., 4 Nov 1988, R. Acosta 2459 (CIB, MEXU); Malinche, N side of the mountain, [19.23705301N, 98.01935192W], 4200 m alt., 10 Aug 1958, J.H. Beaman 2263 (MEXU); Malinche, 2 Nov 1965, W. Boege 10 (MEXU); 10 km sobre la desviación al albergue del volcán La Malinche, carretera a Teacalco, [19.278456N, 98.042307W], 3550 m alt., 2 Aug 1983, P. Guerrero et al. 619 (MEXU); volcán La Malinche [19.258333N, 98.0325W], 3950 m alt., 2 Aug 1983, P. Guerrero et al. 624 (MEXU); Parque Nacional de La Malinche, 3500 m alt., 17 Oct 1992, E. Oviedo 55 (MEXU); 200 m después del fin del bosque, parte alta de La Malinche, [19.24027778N, 98.03W], 3910 m alt., 7 Oct 1993, J. Rosado 101 (MEXU), 102 (MEXU), 103 (MEXU); N slope of the cerro Matlalcueyetl, [19.23705301N, 98.01935192W], 3503 m alt., 26 Oct 1953, E. Sohns 665 (MEXU, US). **Municipio San Francisco Tetlanohcan**, 5 km al SE del albergue Malintzi, [19.25260492N, 98.03697747W], 3670 m alt., 15 Sep 2004, A. Mora et al. 315 (FCME). **Veracruz: Municipio Ayahualulco**, entronque del camino a la toma de agua, entre El Triunfo–Los Laureles, [19.45N, 97.18333333W], 3000 m alt., 14 Nov 1995, J. Becerra et al. 290 (XAL), 22 Nov 1995, 354 (XAL), 12 Dec 1995, 691 (XAL). **Municipio Calcahualco**, NE side of Pico de Orizaba, [19.04304N, 97.255242W], 4250–4450, 16 Aug 1958, J.H. Beaman 2274 (MEXU, TEX); La Cuchilla, camino al Pico de Orizaba, por Coscomatepec, [19.06728503N, 97.19152382W], 3160 m alt., 22 Jul 1982, R. Guzmán et al. 5841 (MEXU); 1 km al SO de Jacal, [19.140278N, 97.130555W], 3100 m alt., 26 Apr 1985, J.L. Martínez 99 (XAL). **Municipio Coscomatepec,** camino al Pico de Orizaba, por Coscomatepec, [19.05844254N, 97.14998176W], 2400 m alt., 22 Jul 1982, R. Guzmán et al. 5852 (MEXU). **Municipio Ixhuacán de los Reyes**, Los Laureles, alrededores, [19.43333333N, 97.16666667W], 2500 m alt., 22 Dec 1995, J. Becerra et al. 888 (XAL); faldas del Cofre de Perote, [19.45N, 97.166667W], 3900 m alt., 13 Dec 1979, J.I. Calzada 5707 (MEXU). **Municipio La Perla**, Pico de Orizaba, NE side of mountain, 4250–4450 m alt., 16 Aug 1958, J.H. Beaman 2274 (US); SE del volcán Pico de Orizaba, 19.00241667N, 97.27030556W, 4377 m alt., 5 Jun 2014, R. Hernández 1717 (MEXU); camino Potrero Nuevo–Las Cabañas de Manuel, [19.04555556N, 97.20305556W], 3360 m alt., 13 Nov 1998, F. Lorea and C. Durán 5817 (XAL); Mt. Orizaba, Mar 1908, C.A. Purpus 3014 (US); Pic d’Orizaba, 4200 m alt., Oct 1906, H. Ross 1263 (US); Mt. Orizaba, 4267–4572 m alt., 26 Feb 1892, J.G. Smith 595 (US). **Municipio Perote**, Cofre de Perote, summit of mountain, [19.494444N, 97.147953W], 4192 m alt., 6 Aug 1958, J.H. Beaman 2152 (TEX); Cofre de Perote, E side of mountain, [19.4833N, 97.09W], 1965 m alt., 6 Aug 1958, J.H. Beaman 2182 (US); faldas del Cofre de Perote, [19.483333N, 97.166667W], 3900 m alt., 13 Nov 1979, J.I. Calzada 5707 (IBUG, TEX, XAL); summit of Cofre de Perote, [19.495N, 97.1486W], 4140 m alt., S.J. Darbyshire and M. González 4799 (US); Cofre de Perote, 4150 m alt., 27 Sep 1973, C. Delgadillo and J. Dorantes 3081 (XAL); Cofre de Perote, 50 m al S de la estación de TV, [19.49305556N, 97.14861111], 4170 m alt., 10 Sep 1992, B.V. Hernández 70 (CIB, XAL); NW sloples of Volcán Perote, [19.49251978N, 97.14994892W], 8 Oct 1978, H.H. Iltis et al. 873 (MEXU); 10 km SE of Perote along road to top of Volcán Cofre de Perote, 2 km by road, below poblado Alberjan, [19.491667N, 97.203611W], 2800 m alt., 8 Oct 1978, H.H. Iltis et al. 873 (XAL); ca. 15 Km SE of Perote, on steep slopes along rd near top NW slope of Volcán Cofre de Perote, 19.5N, 97.2W, 4192 m alt., 8 Oct 1978, Iltis, H.H. et al. 905 (CHAPA, TEX, XAL), 916 (XAL), 919 (IBUG, MEXU); Cofre de Perote, 4000 m alt., 20 Jan 1977, W. Márquez 922 (MEXU, TEX, XAL); por la vereda de Los Altos de La Laguna Tilapa, Parque Nacional Cofre de Perote, 3300 m alt., 6 Jul 1983, F. Narave and F. Vázquez 770 (MEXU); Cofre de Perote, 8.4 km al S de El Conejo por el camino a las torres, 19.49645N, 97.15245W, 3993 m alt., 26 Aug 2012, Y. Ramírez-Amezcua et al. 2170 (MEXU); 9 km al S de El Conejo, Cima Cofre de Perote, Instalaciones de Antenas TV Azteca, 19.49175N, 97.14822222W, 4158 m alt., 26 Oct 2010, V. Santos et al. 4 (INEGI, MEXU), 108 (XAL). **Municipio Xico**, W side of Cofre de Perote, [19.495978N, 97.114905W], 2182 m alt., 6 Aug 1958, J.H. Beaman 2182 (MEXU).

***Agrostis turrialbae***

**GUATEMALA. Huehuetenango:** **Municipio Chiantla**, Aldea San Nicolás Chiantla, [15.45330246N, 91.40226796W], 3160 m alt., 1 Dec 1976, D.N. Smith 457 (F). **Municipio San Mateo Ixtatán**, cerro Cananá, between Nucapuxlac and Cananá, Sierra de los Cuchumatanes, [15.8719N, 91.4203W], 2500–2800 m alt., 18 Jul 1942, J.A. Steyermark 49026 (F). **MEXICO.** **Chiapas: Municipio Chamula**, slope near Paraje Cruston, [16.75N, 92.57W], 2500 m alt., 14 Nov 1980, D.E. Breedlove and B.M. Bartholomew 55496 (MO). **Municipio Motozintla**, near summit of cerro Mozotal., [15.419722N, 92.336667W], 2750 m alt., 24 Nov 1981, D.E. Breedlove and B.M. Bartholomew 55846 (MO). **Mexico: Municipio Amecameca**, Joya de Alcalican, extremo SW del Iztaccíhuatl, [19.152778N, 98.673333W], 3900 m alt., 26 Nov 1978, J. Rzedowski, 36013 (IEB, XAL); La Joya de Alcalican, pies de Iztaccíhuatl, [19.141667N, 98.675W], 3950 m alt., 23 Nov 1975, M.E. Vargas s.n. (CHAPA). **Municipio Ixtapaluca**, cima del cerro Telapón, 19.371N, 98.72W, 4079 m alt., 12 Jul 2013, R. Hernández-Cárdenas and L. Arredondo-Amezcua 1256 (IEB); Estación Experimental de Investigación y de Enseñanza de Zoquiapan, km 50 de la autopista México–Puebla, Llano de San Miguel, [19.28980287N, 98.67285299W], 3350 m alt., 26 Aug 1978, I. Sánchez 2226 (CHAPA); Estación Experimental de Investigación y de Enseñanza de Zoquiapan, 8 km al S de Río Frío, Llano Aculco, [19.29079527N, 98.67281007W], 3230 m alt., 26 Jul 1978, R. Vega 464 (CHAPA). **Municipio Tlalmanalco**, faldas del Volcán Iztaccíhuatl, espinazo que cierra el término E de la cañada La Joya, 13 km al E de Amecameca, [19.141667N, 98.675W], 4050 m alt., 25 Nov 1976, S.D. Koch 76296 (CHAPA); vertiente NW del Iztaccíhuatl, en la región de Peñas Cuatas, [19.668889N, 99.328611W], 4000 m alt., 30 Dec 1965, J. Rzedowski 21791 (MEXU). **Municipio Zinacantepec**, Nevado de Toluca, on N side of mountain, 9 Sep 1957, J.H. Beaman 1707 (US); cráter del Nevado de Toluca, [19.108075N, 99.768824W], 4240 m alt., Sep 1958, E. Hernández X. s.n (CHAPA); 750 m de la estación meterológica, Nevado de Toluca, [19.120833N, 99.753333W], 4110 m alt., 17 Sep 1997, A. Ramírez 769 (MEXU). **Without municipality**, Acatzingo, cerca de Ozumba, 1900 m alt., 13 Sep 1953, E. Matuda 28950 (MEXU). **Querétaro: Municipio Cadereyta**, alrededores de El Doctor, [20.87513889N, 99.65602778W], 2600 m alt., 11 Oct 1988, J. Rzedowski 45086 (IEB). **Municipio Landa**, El Madroño, por la carretera a Pinal de Amoles, [21.28506944N, 99.14247778W], 1600 m alt., 24 Aug 1982, R. Guzmán 5957 (MEXU); Lobo, [21.29275N, 99.11930833W], 1600 m alt., 24 Aug 1982, R. Guzmán 5961 (MEXU). **Veracruz: Municipio Coscomatepec**, camino al Pico de Orizaba, por Coscomatepec, [19.05844254N, 97.14998176W], 2400 m alt., 22 Jul 1982, R. Guzmán 5853 (MEXU).
